# Polydatin ameliorates early brain injury after subarachnoid hemorrhage through up-regulating SIRT1 to suppress endoplasmic reticulum stress

**DOI:** 10.3389/fphar.2024.1450238

**Published:** 2024-09-04

**Authors:** Yuwei Han, Guangzhi Hao, Song Han, Tingzhun Zhu, Yushu Dong, Ligang Chen, Xinyu Yang, Xiaoming Li, Hai Jin, Guobiao Liang

**Affiliations:** Department of Neurosurgery, General Hospital of Northern Theater Command, Shenyang, Liaoning, China

**Keywords:** Polydatin, subarachnoid hemorrhage, endoplasmic reticulum stress, SIRT1, early brain injury

## Abstract

**Objective:**

This study aims to investigate the inhibitory effect of Polydatin (PD) on endoplasmic reticulum (ER) stress following subarachnoid hemorrhage (SAH) and to elucidate the underlying mechanisms.

**Methods:**

A standard intravascular puncture model was established to mimic SAH in mice. Neurological functions were assessed using neurological scoring, Grip test, and Morris water maze. Brain edema and Evans blue extravasation were measured to evaluate blood-brain barrier permeability. Western blot and quantitative real-time polymerase chain reaction (PCR) analyses were performed to examine protein and mRNA expressions related to ER stress. Terminal deoxynucleotidyl transferase-mediated dUTP nick end labeling (TUNEL) staining was used to detect cell apoptosis, and transmission electron microscopy was used to observe the ultrastructure of the endoplasmic reticulum.

**Results:**

The results indicated that PD significantly reduced brain edema and Evans blue extravasation after SAH, improving neurological function. Compared to the SAH group, the expression levels of ER stress-related proteins including glucose-regulated protein 78 (GRP78), phosphorylated protein kinase R-like endoplasmic reticulum kinase (p-PERK), phosphorylated eukaryotic initiation factor 2α (p-eIF2α), activating transcription factor 4 (ATF4), and C/EBP homologous protein (CHOP), were significantly lower in the PD-treated group. Moreover, PD significantly enhances the protein expression of Sirtuin 1 (SIRT1). Validation with sh-SIRT1 confirmed the critical role of SIRT1 in ER stress, with PD’s inhibitory effect on ER stress being dependent on SIRT1 expression. Additionally, PD attenuated ER stress-mediated neuronal apoptosis and SAH-induced ferroptosis through upregulation of SIRT1.

**Conclusion:**

PD alleviates ER stress following SAH by upregulating SIRT1 expression, thereby mitigating early brain injury. The protective effects of PD are mediated through SIRT1, which inhibits ER stress and reduces neuronal apoptosis and ferroptosis.

## Graphical Abstract

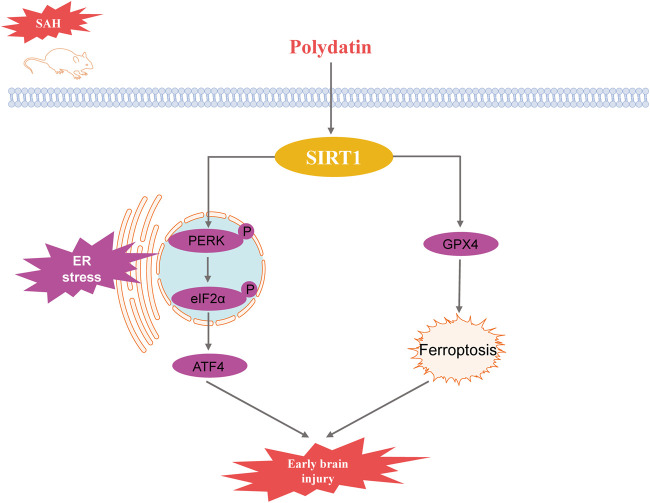

## 1 Introduction

Subarachnoid hemorrhage (SAH) is a neurological disorder characterized by high morbidity and mortality, primarily caused by ruptured aneurysms (Muehlschlegel, 2018). Early brain injury (EBI) following SAH significantly contributes to poor patient outcomes (Dou et al., 2017). Therefore, the alleviation of EBI is crucial for improving postoperative prognosis in SAH patients.

Endoplasmic reticulum (ER) stress-mediated apoptosis plays an important role in the pathology of EBI after SAH (Chen et al., 2020; Tian et al., 2020; Xu et al., 2018; Xu et al., 2019). ER is the largest organelle responsible for the translation, folding and translocation of membrane or secretory proteins (Almanza et al., 2019). Sustained or excessive ER stress can induce activation of apoptotic signaling pathways, which involve three main signalling pathways: C-EBP homologous protein (CHOP), Jun N-terminal kinase (JNK) and Caspase-12. CHOP is an ER-specific stress protein whose expression increases with the onset of ER stress. CHOP ultimately leads to apoptosis by inhibiting the expression of glucose-regulated protein 78 (GRP78) and the anti-apoptotic gene B-cell lymphoma 2 (Bcl-2). In addition, CHOP can also induce apoptosis through the Bax pathway, an apoptosis-related gene (Tabas and Ron, 2011).

Sirtuin 1 (SIRT1) has been shown to be effective in alleviating ER stress (Melhem et al., 2016; Zhang et al., 2020). Upregulation of SIRT1 inhibited ER stress, improved osteoarthritis (Feng, Chen, Pengcheng, Zhang and Wang, 2019) and reduced apoptosis in rats with chronic obstructive pulmonary disease model (Zhang et al., 2020). SIRT1 is a histone deacetylase that is involved in important physiopathological processes such as oxidative stress, inflammation, immune response and apoptosis (Donmez, 2012; Vellimana et al., 2018). In our previous study, we found that inhibition of SIRT1 expression induced inflammation and exacerbated EBI disease after SAH (Han et al., 2021). However, the role of SIRT1 in regulating endoplasmic reticulum stress in SAH has not been reported.

**FIGURE 1:**
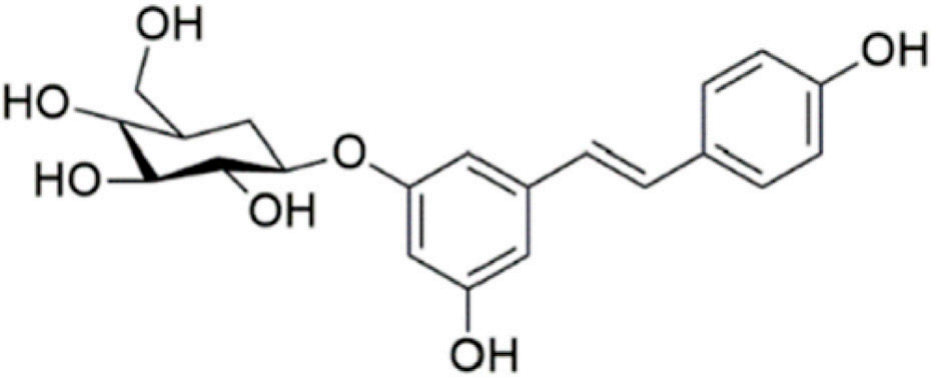
Chemical structural formula of PD.

Polydatin (3,4′,5-trihydroxy-stilbene-3-β-D-glucoside, PD, Figure 1) is a phenolic compound isolated from Polygonum cuspidatum Siebold & Zucc. It has demonstrated stronger antioxidant activity and better water solubility compared to resveratrol (Henry-Vitrac et al., 2006; Henry et al., 2005). PD has garnered attention for its potential therapeutic benefits in various neurological conditions. Research indicates that PD exerts significant neuroprotective effects across several models of neurological disorders. In cerebral ischemia, PD has been shown to mitigate neuronal damage and improve functional outcomes, likely through its antioxidant and anti-inflammatory properties (Shah et al., 2019; Tang and Tan, 2019). Similarly, in traumatic brain injury, PD reduces oxidative stress and neuronal apoptosis, contributing to improved recovery (Li et al., 2019). In the context of spinal cord injury, PD has been reported to enhance functional recovery and reduce secondary damage by modulating oxidative stress and inflammatory responses (Lv et al., 2019a; Lv et al., 2019b). Moreover, PD’s efficacy extends to neurodegenerative diseases. For instance, in Alzheimer’s disease, PD has shown potential in reducing amyloid-beta accumulation and improving cognitive function (Tang, 2021). In Parkinson’s disease, PD appears to protect dopaminergic neurons from degeneration and improve motor function (Huang et al., 2018). However, the effects and mechanisms of PD on SAH remain unexplored. Therefore, this study aims to investigate the efficacy and pharmacological mechanisms of PD in SAH.

## 2 Materials and methods

### 2.1 Animals and SAH models

All experimental protocols adhered to the National Institutes of Health Guide for the Care and Use of Laboratory Animals (NIH Publications No.8023, Revised 1996). Male C57BL/6 mice (20–25 g) were obtained from the Department of Experimental Animals at the General Hospital of Northern Theater Command (Shenyang, China). In accordance with ARRIVE guidelines, the mice were maintained on a 12-h light/dark cycle with *ad libitum* access to food and water. Following a previous study (Han et al., 2022), mice were anesthetized via intraperitoneal injection of 1% sodium pentobarbital (40 mg/kg). The left carotid artery, external carotid artery (ECA), and internal carotid artery (ICA) were exposed. A 4–0 monofilament nylon suture was introduced into the ICA through the ECA. The suture was slowly withdrawn to induce SAH. Sham-operated mice underwent the same procedure without the arterial puncture.

### 2.2 Experimental grouping and drug administration

**FIGURE 2:**
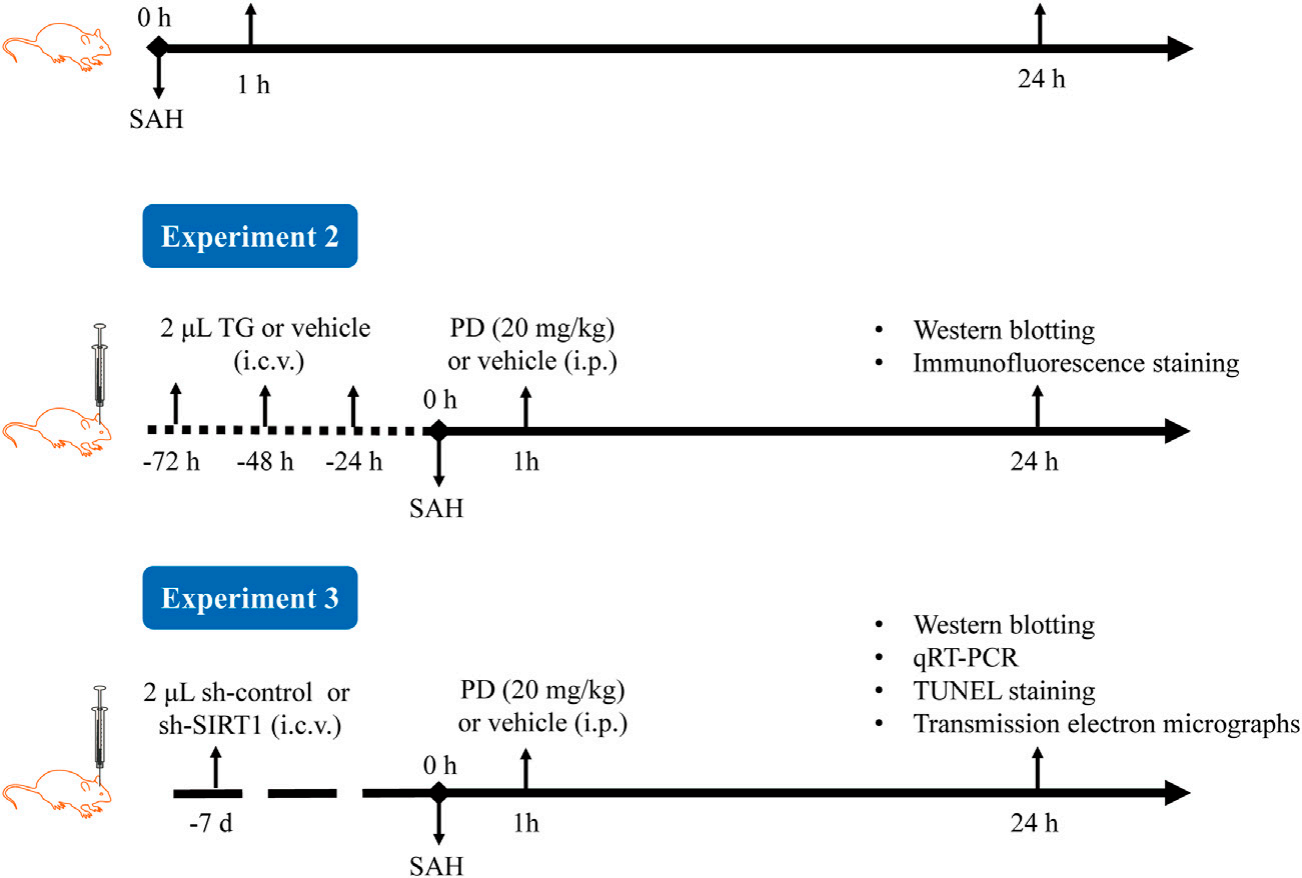
Experimental procedure diagram.

The experimental procedure is shown in Figure 2. In Experiment 1, based on previous research, we selected three doses of PD for intraperitoneal injection: 10, 20, and 30 mg/kg (Bian et al., 2022; Li et al., 2019; Sun et al., 2014). The mice were randomly divided into six groups: sham, SAH, SAH + vehicle, SAH + PD10, SAH + PD20, and SAH + PD30. PD (Selleckchem, USA) was prepared in saline containing 1% DMSO. PD or vehicle was administered intraperitoneally 1 h after SAH induction. Each experiment has six mice per group.

In Experiment 2, mice were randomly assigned to six groups: sham + vehicle, sham + Thapsigargin (TG), SAH + vehicle, SAH + TG, SAH + PD20, and SAH + PD20 + TG. TG, an ER inducer (MedChem Express, US), was prepared at 0.5 mg/mL in saline containing 1% DMSO. Following the previous study (Purkayastha et al., 2011), mice received intracerebroventricular injections of 2 μL TG or an equal volume of vehicle for three consecutive days, followed by SAH induction. PD (20 mg/kg) or vehicle was administered intraperitoneally 1 h after SAH induction.

In Experiment 3, 72 mice were randomly divided into six groups: sham, SAH, SAH + sh-control, SAH + sh-SIRT1, SAH + PD, and SAH + sh-SIRT1 + PD.

### 2.3 Neurological tests

Neurological functions were assessed using an improved Garcia score and Grip test. The Garcia scoring system evaluates body proprioception, spontaneous activity, limb symmetry, climbing, forelimb extension, and response to vibratory stimuli. Higher scores indicate better neurological function. The grip test (Denorme et al., 2016), involved placing mice on a 50 cm long string stretched between two vertical supports, elevated 40 cm above a horizontal surface. The Morris water maze test assessed spatial learning and memory. The Morris Water Maze consists of a circular pool (diameter: 200 cm; height: 60 cm) filled with opaque water. A submerged platform (10 cm diameter) is fixed in one location. Mice are trained over 4 days, with two trials per day. Each trial starts by placing the mouse in one of four quadrants of the pool. The time taken to locate and climb onto the platform (escape latency), swimming path, and distance traveled are recorded.

### 2.4 Brain water content

Mice were euthanized with an overdose of pentobarbital. Brain tissue was collected and weighed immediately (wet weight), then dried in an oven at 105°C for 72 h (dry weight). Brain water content was calculated using the formula: (wet weight-dry weight)/(wet weight) ×100%.

### 2.5 Evans blue extravasation

Evans blue dye (2%, 5 mL/kg; Sigma, USA) was injected into the left femoral vein and allowed to circulate for 1 h. After intracardiac perfusion with saline, brains were collected, weighed, and immersed in formamide (10 mL/g, Sinopharm Chemical Reagent Co., Ltd, China) at 60°C for 24 h. Absorbance of the supernatant was measured at 620 nm.

### 2.6 Lentivirus transfection

sh-control or sh-SIRT1 (Hanbio, Shanghai, China) was injected into the lateral ventricles of mice 7 days before SAH surgery. Using a Hamilton microsyringe (10 µL), 2 µL of sh-SIRT1 lentivirus (1 × 10^^^9 TU/mL) or sh-control lentivirus (1 × 10^^^9 TU/mL) was injected into the left lateral ventricle at a rate of 0.2 μL/min. Coordinates relative to bregma were: anteroposterior, - 0.3 mm; mediolateral, ±1.0 mm; dorsoventral, - 2.5 mm. The needle was left in place for 5 min post-injection to allow diffusion and prevent backflow. The scalp was sutured, and mice were allowed to recover on a heating pad until regaining consciousness.

### 2.7 Western blot

The left cerebral cortex was lysed in RIPA buffer (Beyotime Institute of Biotechnology, China). Protein (40 μg per sample) was separated by SDS-PAGE and transferred to PVDF membranes (Millipore, USA). Membranes were blocked in 5% BSA at room temperature for 1 h and incubated overnight at 4°C with primary antibodies against GRP78, ATF4, CHOP, cleaved caspase-3 (CC-3), Bcl-2-associated X protein (Bax), Bcl2, and β-actin (1:200, Santa Cruz, USA), p-PERK, PERK, p-eIF2α, eIF2α, and ferritin heavy chain 1 (FTH1) (1:800, Cell Signaling Technology, USA), and SIRT1, HO-1, and glutathione peroxidase 4 (GPX4) (1:1000, Abcam, UK). Secondary antibodies (1:10,000, Santa Cruz, USA) were incubated at room temperature for 1 h. Protein detection was performed using the ECL Plus chemiluminescence reagent kit (Beyotime Institute of Biotechnology, China) and visualized with the Tanon Imaging System (Tanon, China).

### 2.8 Quantitative real-time polymerase chain reaction (qRT-PCR)

qRT-PCR was performed to analyze SIRT1 mRNA levels. Total RNA was extracted using an RNA isolation kit (Beyotime Institute of Biotechnology, China) and reverse-transcribed into cDNA using a First Strand cDNA synthesis kit (Vazyme Biotech Co., Ltd, Nanjing, China). Real-time PCR was conducted with SYBR Green Supermix (BIO-RAD, US). Primer sequences were: mouse SIRT1 forward, 5′-CGG​CTA​CCG​AGG​TCC​ATA​TAC-3’; reverse, 5′-ACA​ATC​TGC​CAC​AGC​GTC​AT-3’; mouse GAPDH forward, 5′-CCC​TTA​AGA​GGG​ATG​CTG​CC-3’; reverse, 5′-ACT​GTG​CCG​TTG​AAT​TTG​CC-3’. Amplification conditions were 1 cycle at 95°C for 2 min followed by 39 cycles at 95°C for 15 s and 60°C for 15 s mRNA expression was normalized to GAPDH using the 2^−ΔΔCT^ method, where ΔCT = CT_SIRT1_ - CT_GAPDH_ and ΔΔCT = ΔCT_treatment_ - ΔCT_sham_.

### 2.9 Terminal deoxynucleotidyl transferase-mediated dUTP nick end labeling (TUNEL) staining

Brains were fixed in 4% paraformaldehyde for 24 h, then placed in 30% sucrose solution for 2 days. Sections (4 μm thick) were incubated with primary antibody Neun (1: 50, Abcam, USA) at 4°C overnight, followed by digestion with 20 μg/mL protease K (Beyotime Institute of Biotechnology, China) at 37°C for 30 min. TUNEL reaction mixture was applied at 37°C for 1 h. Sections were observed using a fluorescence microscope (Nikon, Japan) at ×400 magnification.

### 2.10 Transmission electron micrographs

Brains were fixed in 4% paraformaldehyde overnight at 4°C, washed with 0.1 M imidazole-HCl buffer (pH 7.2), and fixed in 2.5% glutaraldehyde in 0.1 M imidazole buffer. Sections were stained with 2% uranyl acetate and lead citrate. Grids were examined using a HT7800 transmission electron microscope (Hitachi High Technologies, Dallas, TX) at 80 kV.

### 2.11 Statistical analysis

Results are presented as mean ± SD. Data were analyzed using GraphPad Prism software (Version 8.0, GraphPad Software Inc., CA). Significance between groups was determined using one-way analysis of variance (ANOVA) for comparisons involving three or more groups, or Student’s two-tailed *t*-test or Dunnett’s test where appropriate. Statistical significance was set at *p <* 0.05.

## 3 Results

### 3.1 PD alleviates EBI after SAH

**FIGURE 3:**
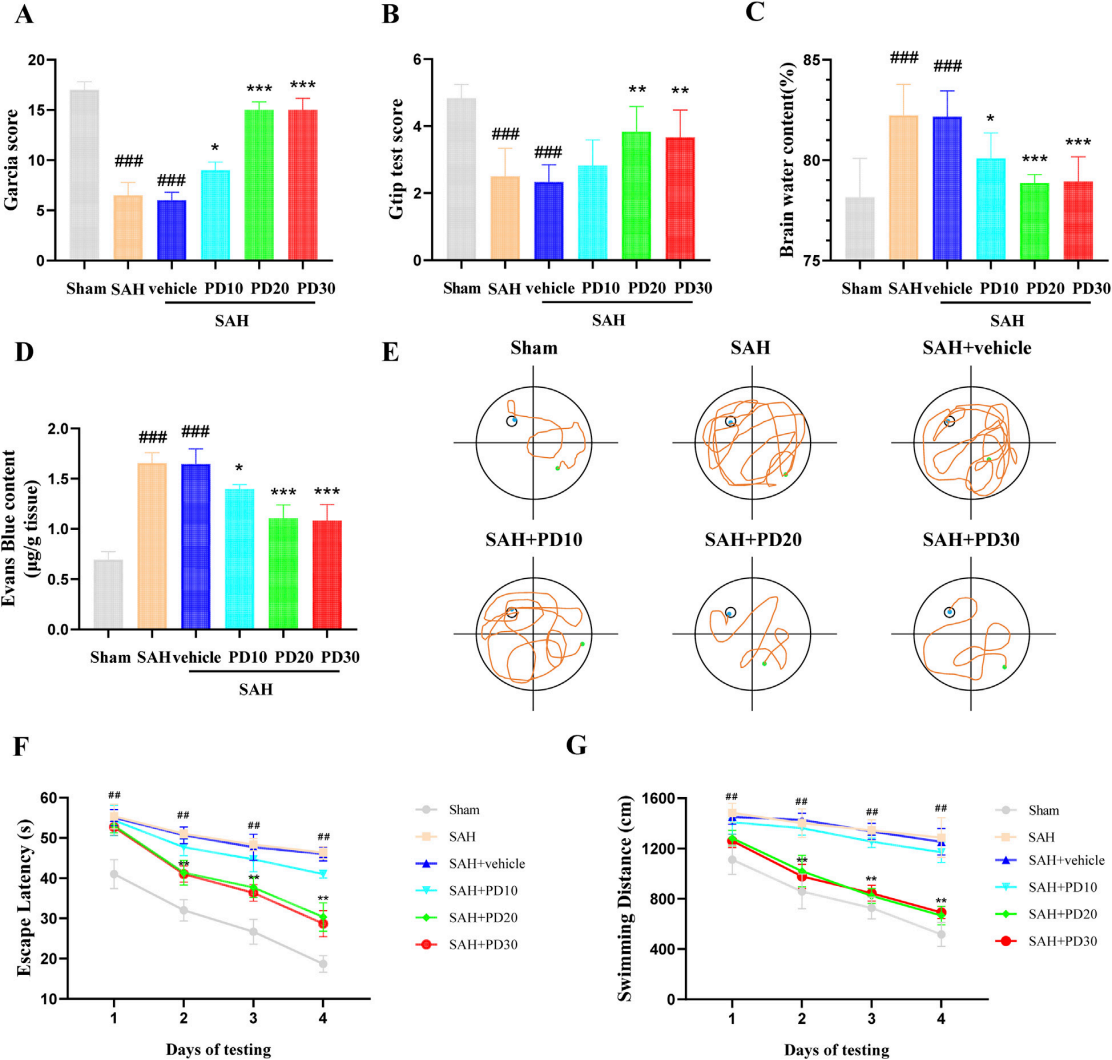
Effects of PD on EBI 24 h after SAH. **(A)** Garcia score; **(B)** Brain water content; **(C)** Evans blue exudation; **(D)** Gtip test score; **(E)** Representative tracing images from the Morris water maze test; **(F–G)** Escape latencies and swimming distance over 4 days. Data were presented as mean ± SD (n = 6). Compared with the sham group, ^*###*^ *P <* 0.001, ^*##*^ *P <* 0.01; Compared with the SAH + vehicle group, ^***^ *P <* 0.001, ^**^ *P <* 0.01, ^*^ *P <* 0.05.

Compared with the sham group, the Garcia scores and Grip test score in the SAH group and the SAH + vehicle group were significantly decreased, while the Garcia scores and Grip test score in the medium dose (20 mg/kg) and high dose (30 mg/kg) PD group increased significantly (*p <* 0.001, Figures 3A, B). The brain water content and Evans blue exudation were higher than those in sham group (*p <* 0.001, Figures 3C, D). The administration of PD was significantly reduced brain water content and Evans blue exudation (*p <* 0.05, Figures 3C, D). The Morris water maze test (Figure 3E) revealed that PD treatment (20 mg/kg and 30 mg/kg) significantly reduced the escape latency to the platform (Figure 3F) and the swimming distance (Figure 3G). There was no significant difference in the above data between the medium dose group and the high dose group of PD (*p >* 0.05). Based on the experimental results above, we selected the moderate dose of PD (20 mg/kg) for all subsequent experiments.

### 3.2 PD inhibits ER stress after SAH

**FIGURE 4:**
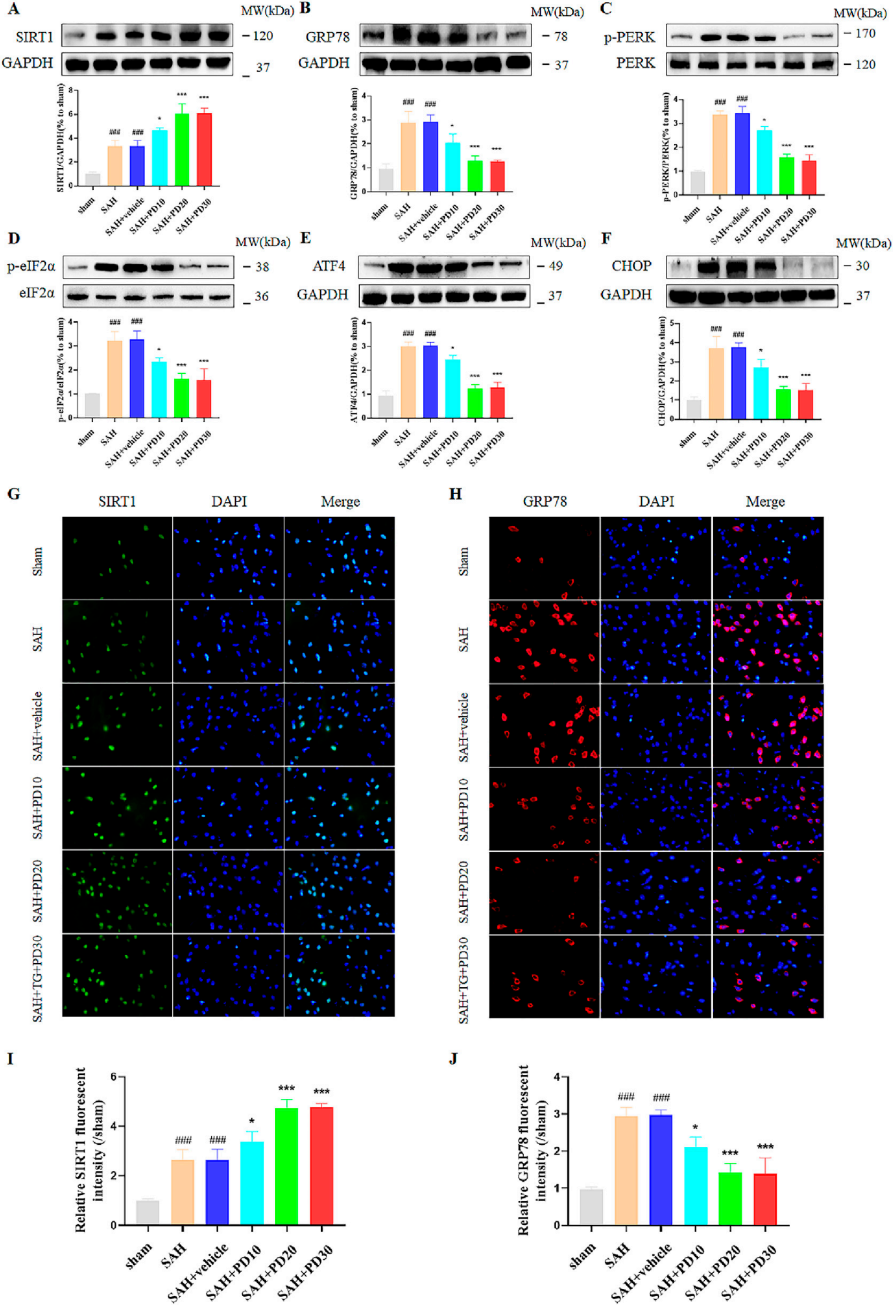
Effects of different concentrations of PD on ER stress. The protein expression of SIRT1 **(A)**, GRP78 **(B)**, p-PERK **(C)**, p-eIF2α **(D)**, ATF4 **(E)**, and CHOP **(F)**; The immunofluorescence staining of SIRT1 **(G)** and GRP78 **(H)**; Quantitative analysis of fluorescence intensity **(I, J)**. Data were presented as mean ± SD (*n* = 6). Compared to sham group, ^*###*^ *P <* 0.001; Compared with the SAH + vehicle group, ^***^ *P <* 0.001, ^*^ *P <* 0.05.

Compared with the sham group, the SIRT1 expression in SAH group was significantly increased, and the expression of SIRT1 in the medium and high dose PD group increased significantly (*p <* 0.001, Figure 4A). There was no significant difference between the medium dose group and the high dose group of PD (*p >* 0.05). Compared with the sham group, the expression of ER stress related proteins GRP78, p-PERK, p-eIF2α, ATF4 and CHOP was significantly increased in the SAH group, which suggested that ER stress did occur after SAH (*p <* 0.001, Figures 4B–F). Compared with the SAH group, the administration of the medium dose group and the high dose of PD significantly decreased the expression of ER stress-related proteins. There was no significant difference between the medium dose group and the high dose group of PD (*p >* 0.05). Immunofluorescence staining experiments also showed similar results, with a significant increase in the immunofluorescence intensity of SIRT1 and GRP78 following SAH. Administration of PD (20 and 30 mg/kg) significantly reduced the immunofluorescence intensity of SIRT1 and GRP78 (Figures 4G–J). These results indicate that PD can upregulate SIRT1 expression and inhibit ER stress. Due to the significant inhibitory effect of PD at 20 mg/kg on ER stress, we confirmed the use of 20 mg/kg PD for subsequent experiments.

**FIGURE 5:**
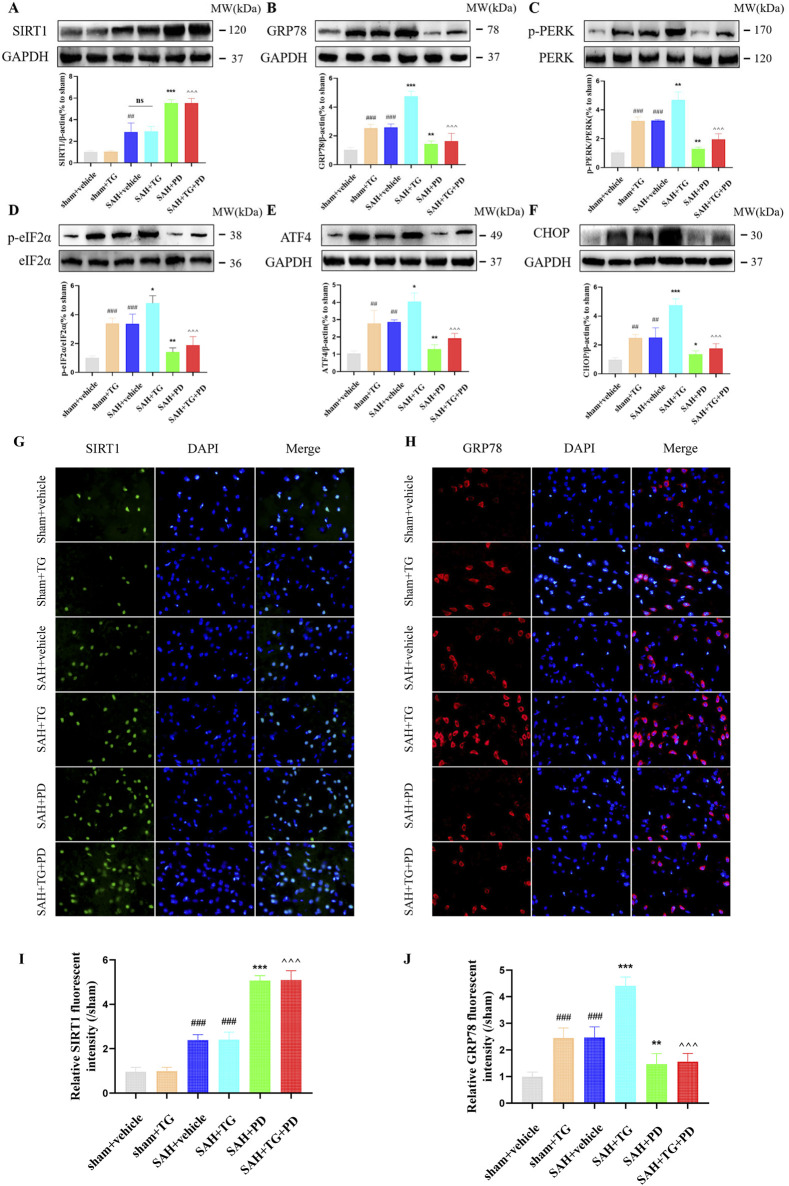
Effects of PD on ER stress after SAH. The protein expression of SIRT1 **(A)**, GRP78 **(B)**, p-PERK **(C)**, p-eIF2α **(D)**, ATF4 **(E)**, and CHOP **(F)**; The immunofluorescence staining of SIRT1 **(G)** and GRP78 **(H)**; Quantitative analysis of fluorescence intensity **(I, J)**. Data were presented as mean ± SD (n = 6). Compared to sham + vehicle group, ^*###*^ *P <* 0.001, ^*##*^ *P <* 0.01; Compared with the SAH + vehicle group, ^***^ *P <* 0.001, ^**^ *P <* 0.01, ^*^ *P <* 0.05; Compared to the SAH + TG group, ^*^^^*^ *P <* 0.001.

PD can alleviate EBI after SAH, we want to know whether ER stress is involved in this process. To test this, we used ER activator (TG) as evidence. Compared with the sham group, the expression of ER stress related proteins GRP78, p-PERK, p-eIF2α, ATF4 and CHOP was significantly increased in the sham + TG group and SAH + vehicle group, and the expression levels of these proteins were similar between the two groups, which suggested that ER stress did occur after SAH (*p <* 0.001, Figures 5A–F). Compared with the SAH + vehicle group and the SAH + TG group, the administration of PD (in the SAH + PD group and in the SAH + TG + PD group) significantly decreased the expression of ER stress-related proteins (*p <* 0.001, Figures 5A–F). The immunofluorescence intensity of SIRT1 and GRP78 was significantly increased in the sham + TG group and SAH + vehicle group. Compared with the SAH + vehicle group and the SAH + TG group, the administration of PD (in the SAH + PD group and in the SAH + TG + PD group) significantly increased the immunofluorescence intensity of SIRT1 and decreased the GRP78 fluorescence intensity (*p <* 0.01, Figures 5G–J). The immunofluorescence staining for SIRT1 and GRP78 showed results consistent with those of the Western blot analysis (Figures 5G–J).

### 3.3 PD inhibits ER stress by up-regulating SIRT1 after SAH

**FIGURE 6:**
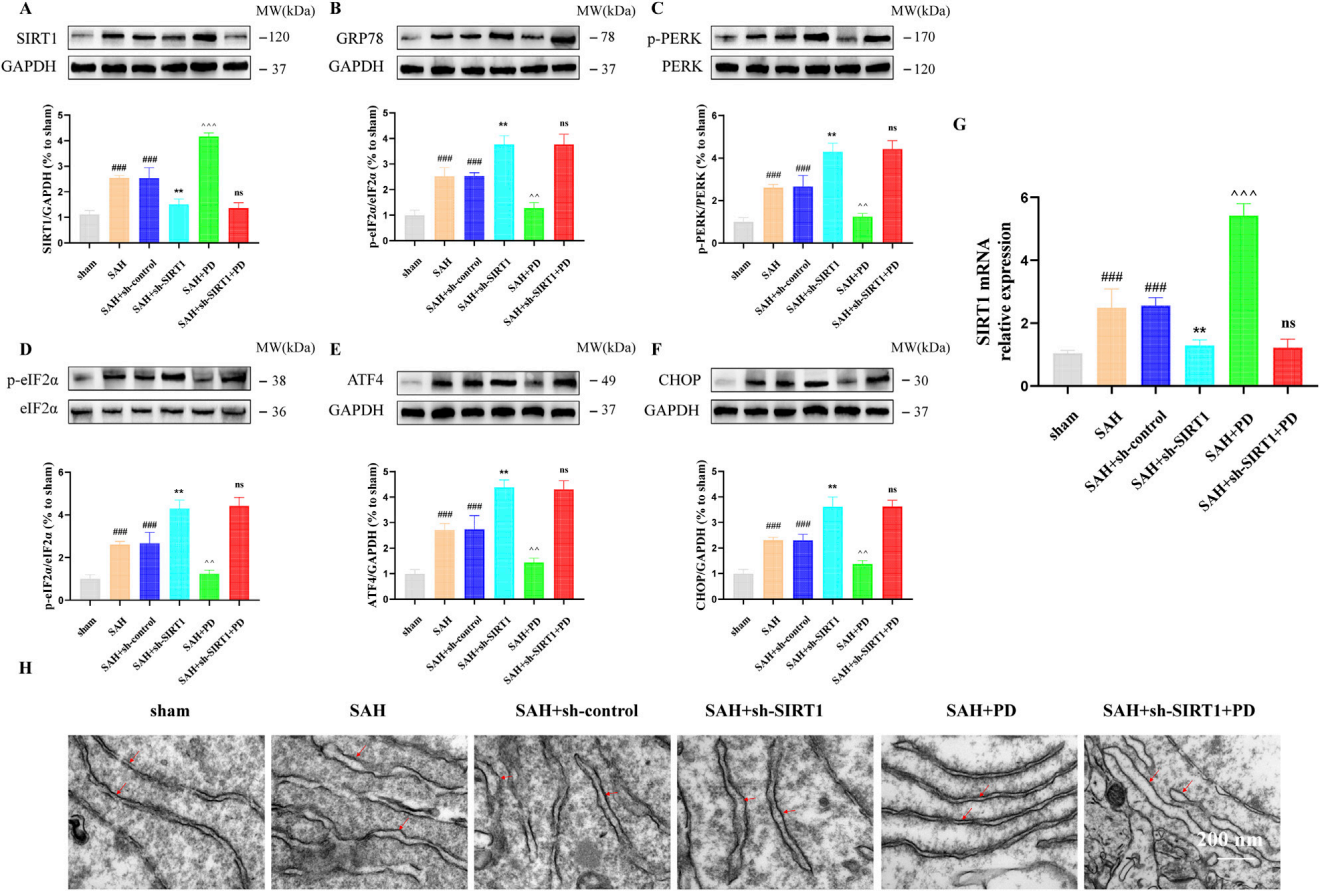
Effect of SIRT1 on ER stress after SAH. The protein expression of SIRT1 **(A)**, GRP78 **(B)**, p-PERK **(C)**, p-eIF2α **(D)**, ATF4 **(E)**, and CHOP **(F)**; SIRT1 mRNA level **(G)**; **(H)** Representative images of transmission electron micrographs of ER (Bar = 200 nm, Magnification 40, 000 ×). Data were presented as mean SD (n = 6). Compared to sham group, ^*###*^ *P <* 0.001; Compared to the SAH + sh-control group, ^**^ *P <* 0.01; Compared to the SAH group, ^*^^^*^ *P <* 0.001, ^*^^*^ *P <* 0.01; Compared to the SAH + sh-SIRT1 group, ns, no significant difference.

As shown in Figure 5, in sham group, SAH group and SAH + PD group, the expression of SIRT1 in each group had no significant difference between TG administration and no administration (*p >* 0.05). These results suggest that TG has no effect on SIRT1 expression, and SIRT1 is the upstream regulator of ER stress pathway. In order to explain that SIRT1 is involved in and mediates the ER stress after SAH, we use sh-SIRT1 to confirm it. Compared with the SAH + sh-control group, the expressions of ER stress-related proteins in SAH + sh-SIRT1 group were significantly increased, suggesting that knockdown of SIRT1 aggravate ER stress response after SAH, and SIRT1 was involved in ER stress after SAH (*p <* 0.001, Figures 6A–F). There was no significant difference in the expression levels of ER stress-related proteins between the SAH + sh-SIRT1 group and the SAH + sh-SIRT1 + PD group (*p >* 0.05, Figures 6A–F), suggesting that the effects of PD were reversed by SIRT1 inhibition.

Under the same grouping we detected the mRNA of SIRT1. As shown in Figure 6G, the mRNA level of SIRT1 was significantly elevated when SAH occurred, compared with the sham group. PD was able to significantly increase the mRNA expression of SIRT1. However, when sh-SIRT1 inhibited the expression of SIRT1 mRNA, PD could no longer increase the expression of SIRT1 mRNA. As shown in Figure 6H, the ER was abnormally swollen and the ER volume was significantly increased in the SAH model group compared with the sham group. The degree of ER abnormally swollen was more pronounced in the SAH + sh-SIRT1 group than in the SAH group. This suggests that inhibiting SIRT1 expression can aggravate ER stress after SAH. However, when PD was used in the SAH model group, the morphology of the ER structure normalised. When both sh-SIRT1 and PD were used in the SAH model group, the ER morphostructure was again abnormally swollen. The above experimental results indicate that the effect of PD on ER stress depends on the expression of SIRT1.

### 3.4 PD inhibits ER stress-mediated neuronal apoptosis and SAH-induced ferroptosis by up-regulating SIRT1

**FIGURE 7:**
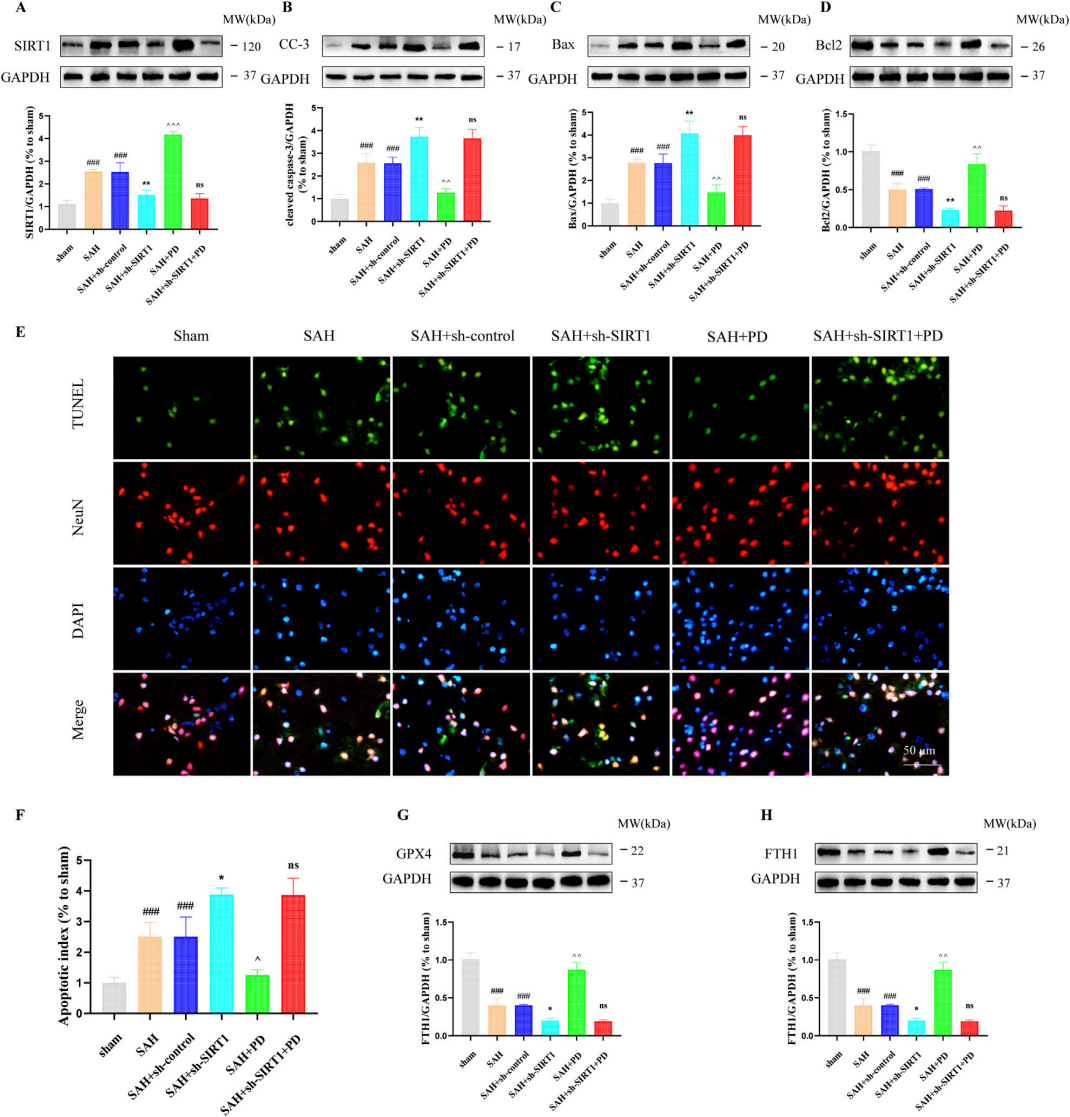
Effect of PD on ER stress-mediated neuronal apoptosis. The protein expression of SIRT1 **(A)**, cleaved caspase-3 **(B)**, Bax **(C)**, Bcl2 **(D)**, TUNEL and NeuN immunofluorescence staining **(E)**, quantitative analysis of fluorescence intensity **(F)**; The protein expression of GPX4 **(G)** and FTH1 **(H)**. Data were presented as mean SD (n = 6). Compared with the sham group, ^*###*^ *P <* 0.001; Compared to the SAH + sh-control group, ^**^ *P <* 0.01, ^*^ *P <* 0.05; Compared to the SAH group, ^*^^^*^ *P <* 0.001, ^*^^*^ *P <* 0.01, ^*^*^ *P <* 0.05; Compared to the SAH + sh-SIRT1 group, ns, no significant difference.

In addition, we also evaluated the ER stress-mediated apoptosis after SAH. Western blotting results showed that compared with the SAH group, PD could decrease the expression of cleaved caspase-3 and Bax, increased the Bcl2 expression. However, cleaved caspase-3 (CC-3) and Bax expression was significantly increased and Bcl2 expression was significantly decreased in the SAH + sh-SIRT1 group. There was no significant difference in the expression levels of apoptosis-related proteins between the SAH + sh-SIRT1group and the SAH + sh-SIRT1 + PD group (*p >* 0.05, Figures 7A–D), suggesting that effect of PD on the expression of apoptosis-related proteins were reversed by SIRT1 inhibition. In addition, compared to the SAH group, PD could increase the number of TUNEL-positive cells in neurons (*p <* 0.01, Figures 7E, F). However, administration of sh-SIRT1 increased the number of TUNEL-positive cells in neurons (*p <* 0.05, Figures 7E, F). There was no significant difference in the number of TUNEL-positive cells between the SAH + sh-SIRT1group and the SAH + sh-SIRT1 + PD group (*p >* 0.05, Figures 7E, F). These results suggested that the effect of PD on apoptosis depends on the expression of SIRT1. Additionally, we found that the levels of anti-ferroptosis related proteins (GPX4 and FTH1) significantly decreased after SAH. PD significantly increased the protein expression levels of GPX4 and FTH1 (Figures 7G, H). Compared to the SAH group, the protein expression levels of GPX4 and FTH1 were significantly reduced in the SAH + sh-SIRT1 group. There was no significant difference in the protein expression levels of GPX4 and FTH1 between the SAH + sh-SIRT1 group and the SAH + sh-SIRT1+PD group. This indicates that PD cannot increase the expression of anti-ferroptosis related proteins after SIRT1 expression is inhibited.

## 4 Discussion

PD is a polyphenolic compound derived from resveratrol combined with glucose, exhibiting stronger antioxidant activity and stability compared to resveratrol. Our study elucidates that PD significantly ameliorates early brain injury following subarachnoid hemorrhage through the upregulation of SIRT1, leading to the suppression of endoplasmic reticulum stress. This finding is pivotal as it not only confirms the neuroprotective effects of PD but also expands our understanding of the underlying mechanisms.

SIRT1, a NAD + -dependent deacetylase, has been extensively studied for its role in cellular stress responses (Yang et al., 2022). Our study demonstrated that PD significantly increases SIRT1 expression in the brain following SAH. SIRT1 activation leads to the activation of several downstream targets, including PGC-1α, which is involved in mitochondrial biogenesis and function (Tang, 2016; Zhao et al., 2021). This activation is crucial for maintaining mitochondrial integrity and function, which are often compromised in brain injury conditions (Zhao et al., 2021).

Moreover, SIRT1 modulates the activity of transcription factors such as NF-κB and p53, reducing inflammation and promoting cell survival (Zhang et al., 2011). The study underscores the role of SIRT1 in enhancing mitochondrial function and reducing oxidative stress (Tripathi et al., 2022), aligning with our findings that SIRT1 activation by PD confers neuroprotection through multiple pathways.

ER stress is a significant contributor to neuronal apoptosis and dysfunction following SAH (Zhang et al., 2023). Our study found that PD reduces markers of ER stress, such as GRP78 and CHOP, through the upregulation of SIRT1. This suppression of ER stress is critical for reducing apoptosis and promoting neuronal survival (Cao, 2015; Kaneko et al., 2017; Rana, 2020; Tabas and Ron, 2011). The study by Tripathi et al. highlights that antioxidants can mitigate ER stress (Tripathi etal., 2023), supporting our findings that PD, as a potent antioxidant, plays a crucial role in reducing ER stress and subsequent neuronal damage.

The interplay between SIRT1 and ER stress pathways involves multiple mechanisms. SIRT1 activation leads to the reduction of eIF2α and ATF4 activity, thereby diminishing the unfolded protein response (UPR) (Luo et al., 2023). This reduction in UPR markers indicates a decrease in ER stress, promoting cell survival and function (Merighi and Lossi, 2022). Our study contributes to the understanding of how targeting SIRT1 can modulate ER stress responses, offering potential therapeutic strategies for brain injuries.

Ferroptosis, a form of regulated cell death characterized by iron accumulation and lipid peroxidation, has been increasingly recognized in the pathology of brain injuries (Costa et al., 2023). Our study explored the potential involvement of ferroptosis in EBI following SAH. We observed that PD treatment reduced the expression of ferroptosis markers such as ACSL4 and GPX4, suggesting that PD may exert its neuroprotective effects by inhibiting ferroptosis. The suppression of ferroptosis by PD could be linked to its antioxidant properties and its ability to upregulate SIRT1. SIRT1 has been shown to regulate iron metabolism and reduce oxidative stress, which are critical factors in ferroptosis (Dang et al., 2022; Li et al., 2021). The results indicate that antioxidants play a role in mitigating ferroptosis and reducing neuronal damage (Singh et la., 2021), which is consistent with our findings that PD can inhibit ferroptosis and protect against early brain injury.

Our findings align with and extend the existing research on the neuroprotective effects of antioxidants and the role of SIRT1 in brain injuries. Furthermore, the role of SIRT1 in neuroprotection has been well-documented. Our study adds to this body of knowledge by demonstrating that PD upregulates SIRT1 to suppress ER stress and ferroptosis, offering a comprehensive mechanism for its neuroprotective effects. While our study provides significant insights into the mechanisms of PD’s neuroprotective effects, several areas warrant further investigation. Future research should explore the long-term effects of PD treatment in SAH models to assess its potential for clinical application. Additionally, studies should investigate the effects of PD in other models of brain injury, such as traumatic brain injury (TBI) and ischemic stroke, to determine the broader applicability of PD as a neuroprotective agent. Exploring the feasibility of PD’s clinical application involves examining its pharmacokinetics, optimal dosing, and potential side effects in human subjects. Clinical trials are necessary to evaluate the safety and efficacy of PD in patients with SAH and other brain injuries.

Moreover, investigating the interplay between SIRT1 and other signaling pathways could uncover new therapeutic targets for brain injury. For example, understanding how SIRT1 interacts with autophagy pathways, mTOR signaling, and other stress response mechanisms could provide deeper insights into the comprehensive neuroprotective effects of PD.

## 5 Conclusion

In conclusion, our study demonstrates that PD ameliorates EBI after SAH by up-regulating SIRT1, leading to the suppression of ER stress and ferroptosis. These findings highlight the multifaceted neuroprotective effects of PD and underscore its potential as a therapeutic agent for SAH. Future research should focus on exploring the long-term effects of PD, its clinical feasibility, and the broader applicability of its neuroprotective mechanisms in various models of brain injury.

## Data Availability

The original contributions presented in the study are included in the article/supplementary material, further inquiries can be directed to the corresponding authors.
