# Corrigendum: Polydatin ameliorates early brain injury after subarachnoid hemorrhage through up-regulating SIRT1 to suppress endoplasmic reticulum stress

**DOI:** 10.3389/fphar.2024.1537295

**Published:** 2025-01-29

**Authors:** Yuwei Han, Guangzhi Hao, Song Han, Tingzhun Zhu, Yushu Dong, Ligang Chen, Xinyu Yang, Xiaoming Li, Hai Jin, Guobiao Liang

**Affiliations:** General Hospital of Northern Theater Command, Shenyang, China

**Keywords:** polydatin, subarachnoid hemorrhage, endoplasmic reticulum stress, SIRT1, early brain injury

In the published article, there was an error in the SAH and SAH + PD10 group of Figure 4H as published. During the preparation of the figure panels, the images for the SAH and SAH + PD10 groups were mistakenly replaced with images from other groups. The corrected Figure 4 and its caption appear below.

**FIGURE 4:**
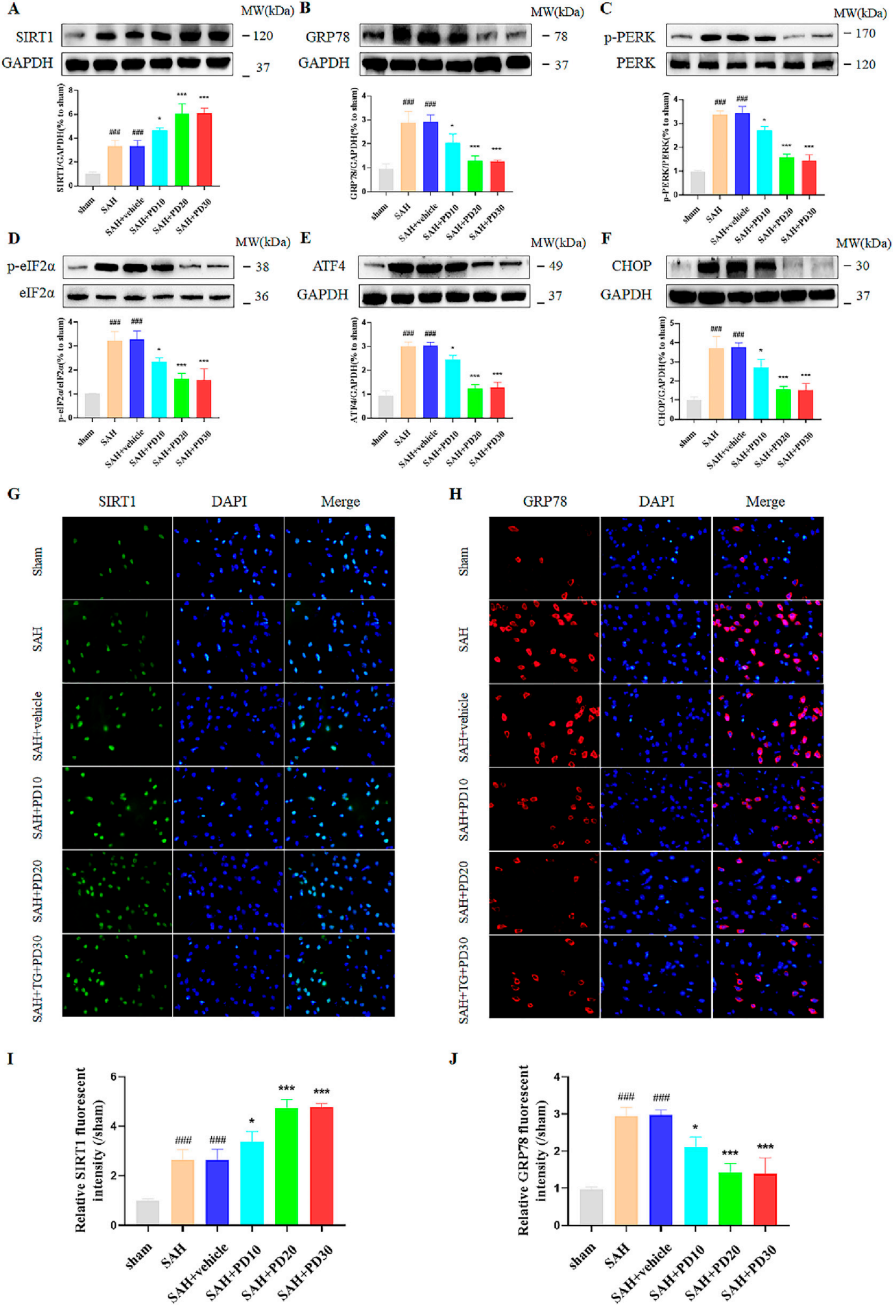
Effects of different concentrations of PD on ER stress. The protein expression of SIRT1 **(A)**, GRP78 **(B)**, p-PERK **(C)**, p-eIF2α **(D)**, ATF4 **(E)**, and CHOP **(F)**; The immunofluorescence staining of SIRT1 **(G)** and GRP78 **(H)**; Quantitative analysis of fluorescence intensity **(I, J)**. Data were presented as mean ± SD (*n* = 6). Compared to sham group, ^###^ *P* < 0.001; Compared with the SAH + vehicle group, ***P < 0.001, *P < 0.05.

Additionally, the previously published Supplementary file “Data Sheet 2” is being updated with a new file that includes protein bands for protein bands for ATF4, CHOP, GRP78, eIF2α, and p-PERK.

The authors apologize for these errors and state that these do not change the scientific conclusions of the article in any way. The original article has been updated.

