# Supplementary figures and images for: Polydatin ameliorates early brain injury after subarachnoid hemorrhage through up-regulating SIRT1 to suppress endoplasmic reticulum stress (part 1 of 2)
Source: Front Pharmacol. 2024 Sep 4;15:1450238. doi: 10.3389/fphar.2024.1450238 (PMC11408241; doi:10.3389/fphar.2024.1450238)

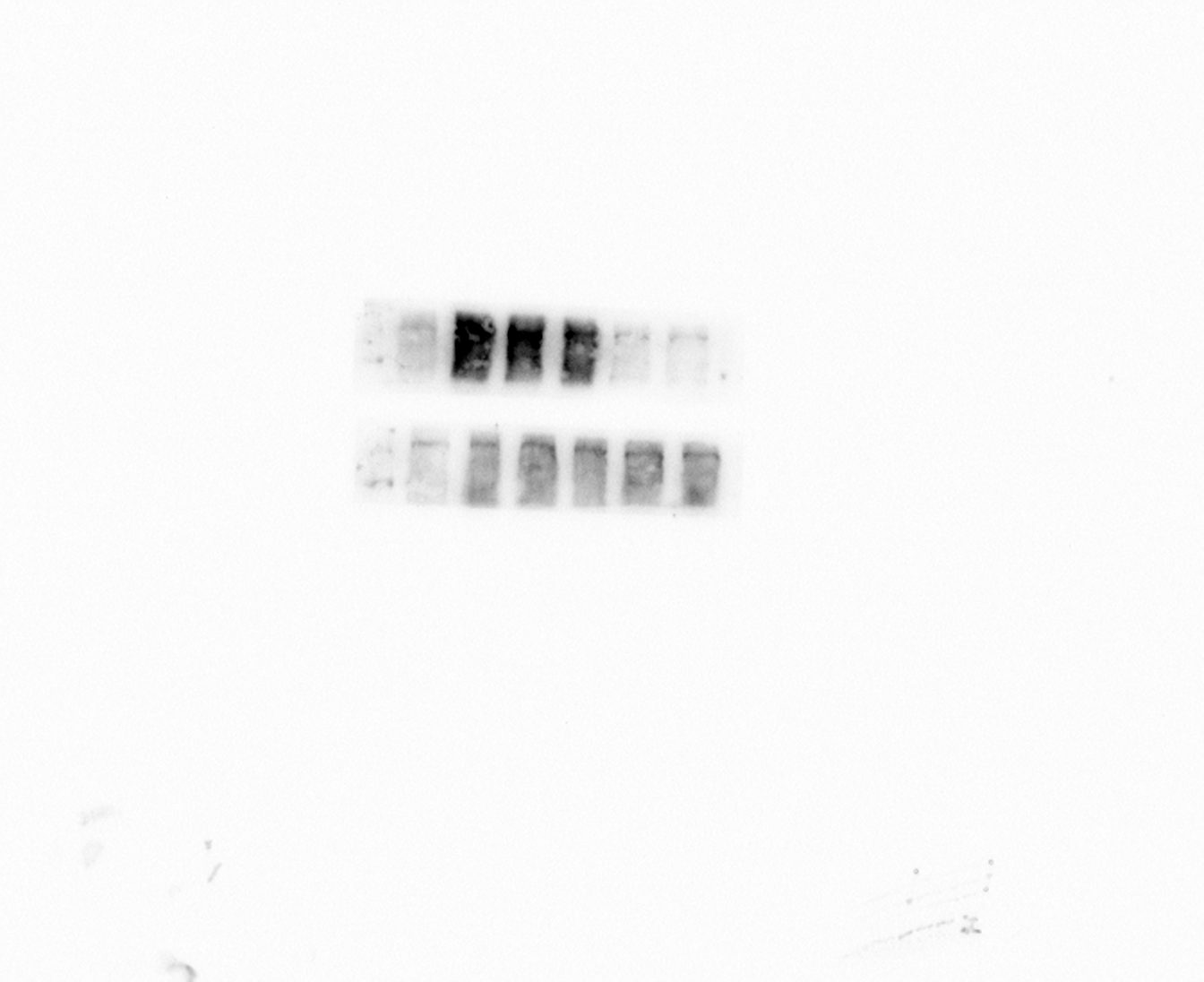

Supplement: Supplementary file 1 [file DataSheet1.ZIP › data/2022.8.30/atf4/10s.Tif]

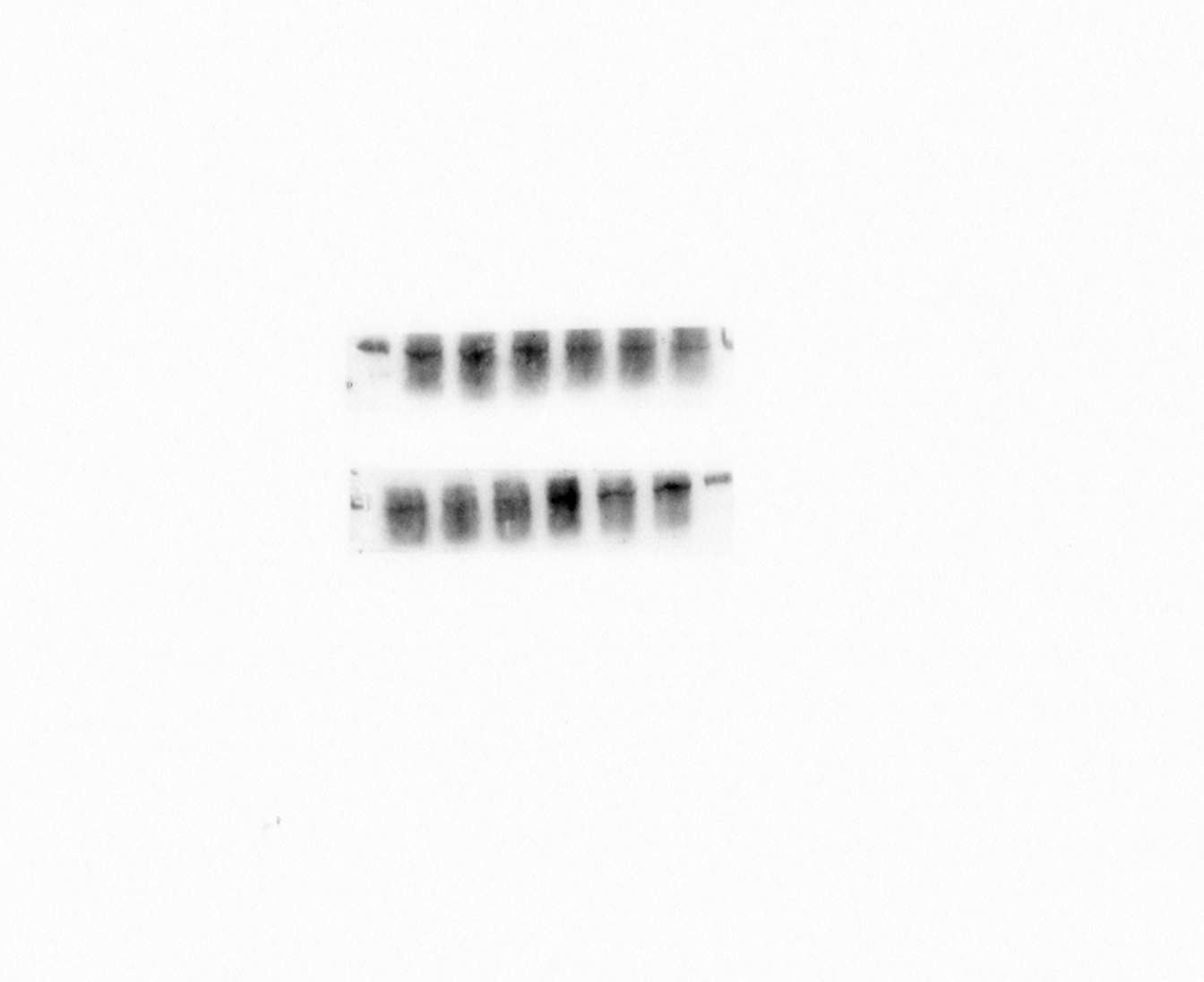

Supplement: Supplementary file 1 [file DataSheet1.ZIP › data/2022.8.30/chop/10s.Tif]

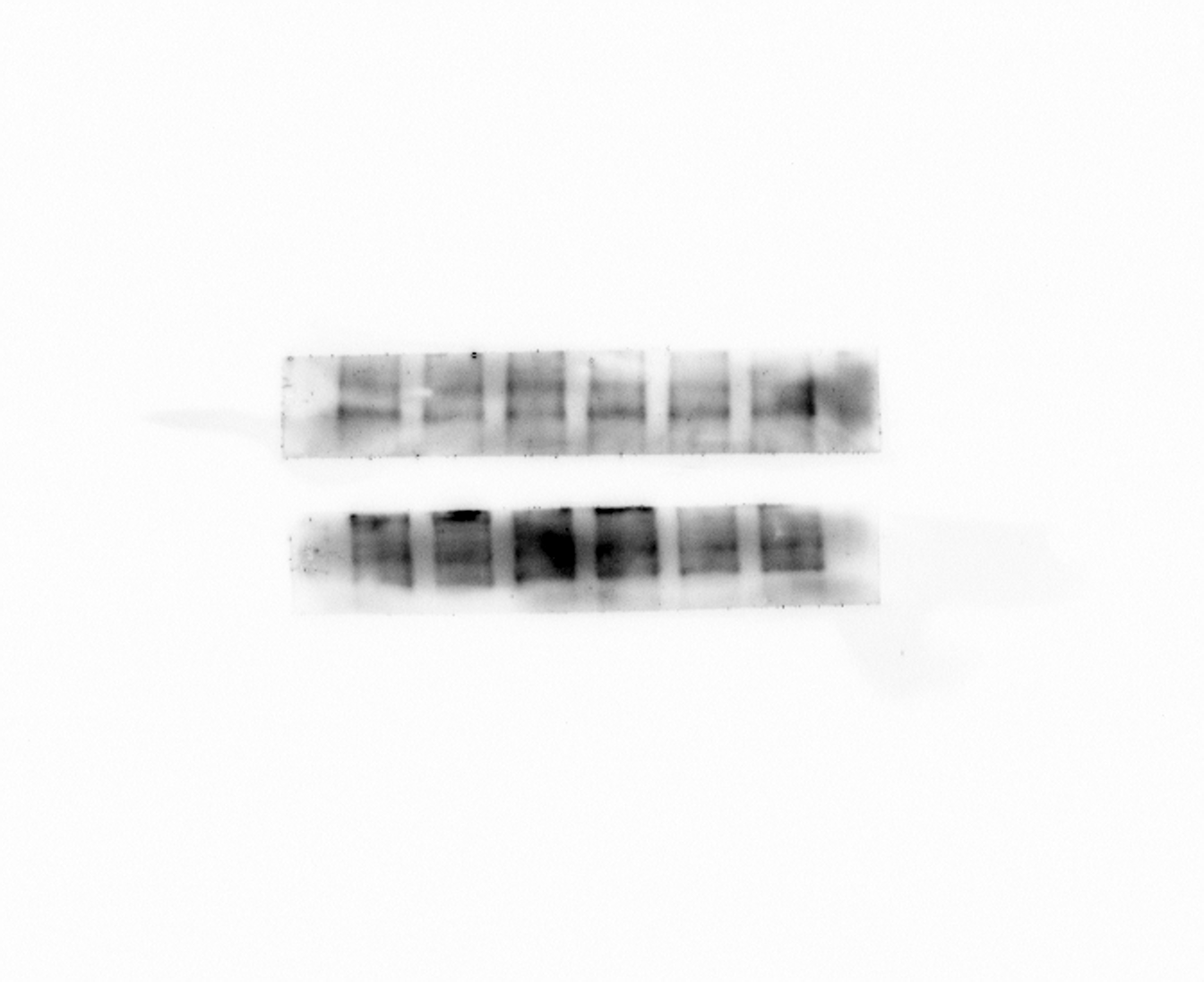

Supplement: Supplementary file 1 [file DataSheet1.ZIP › data/2022.8.30/gapdh/1.Tif]

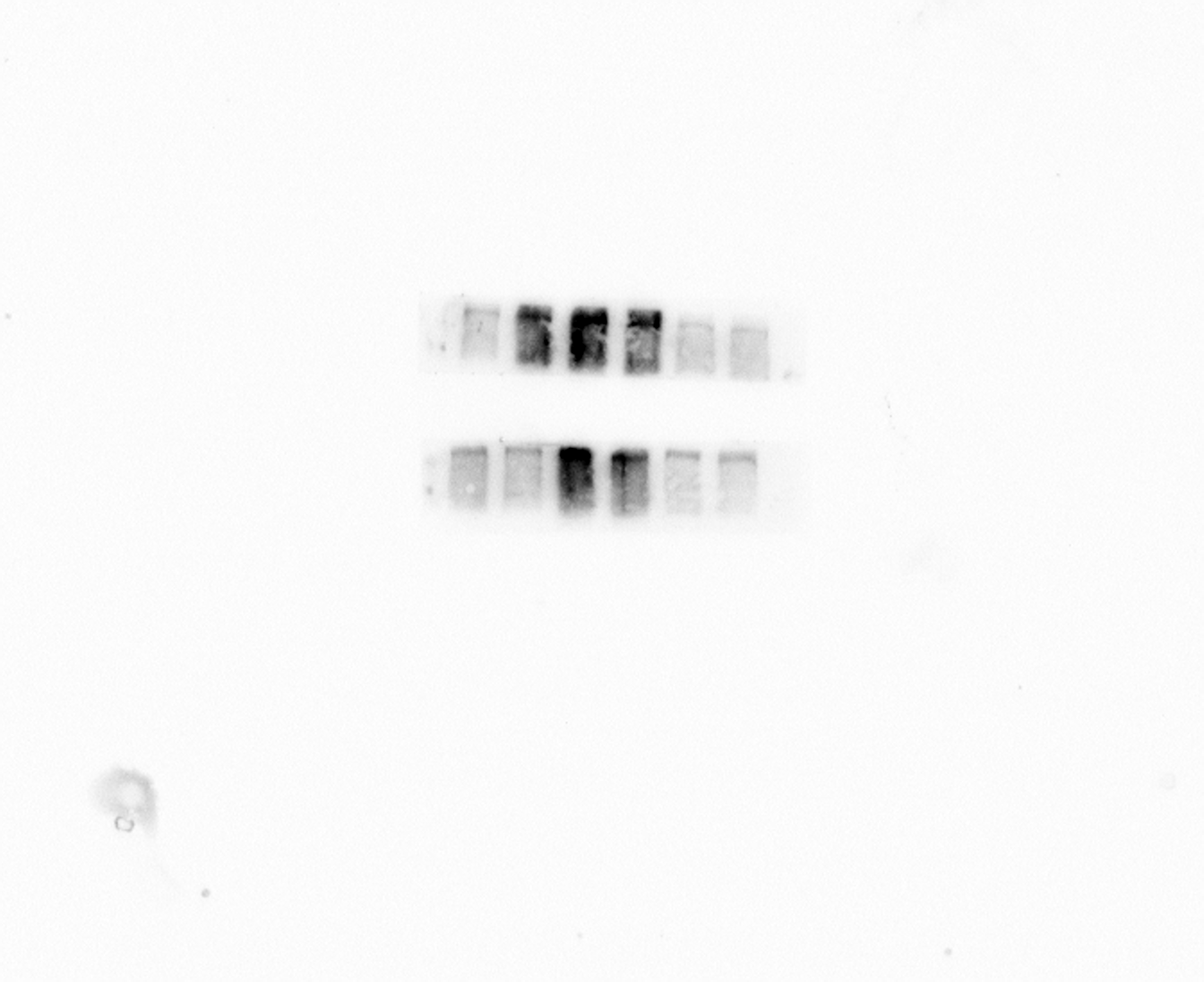

Supplement: Supplementary file 1 [file DataSheet1.ZIP › data/2022.8.30/GRP78/10S.Tif]

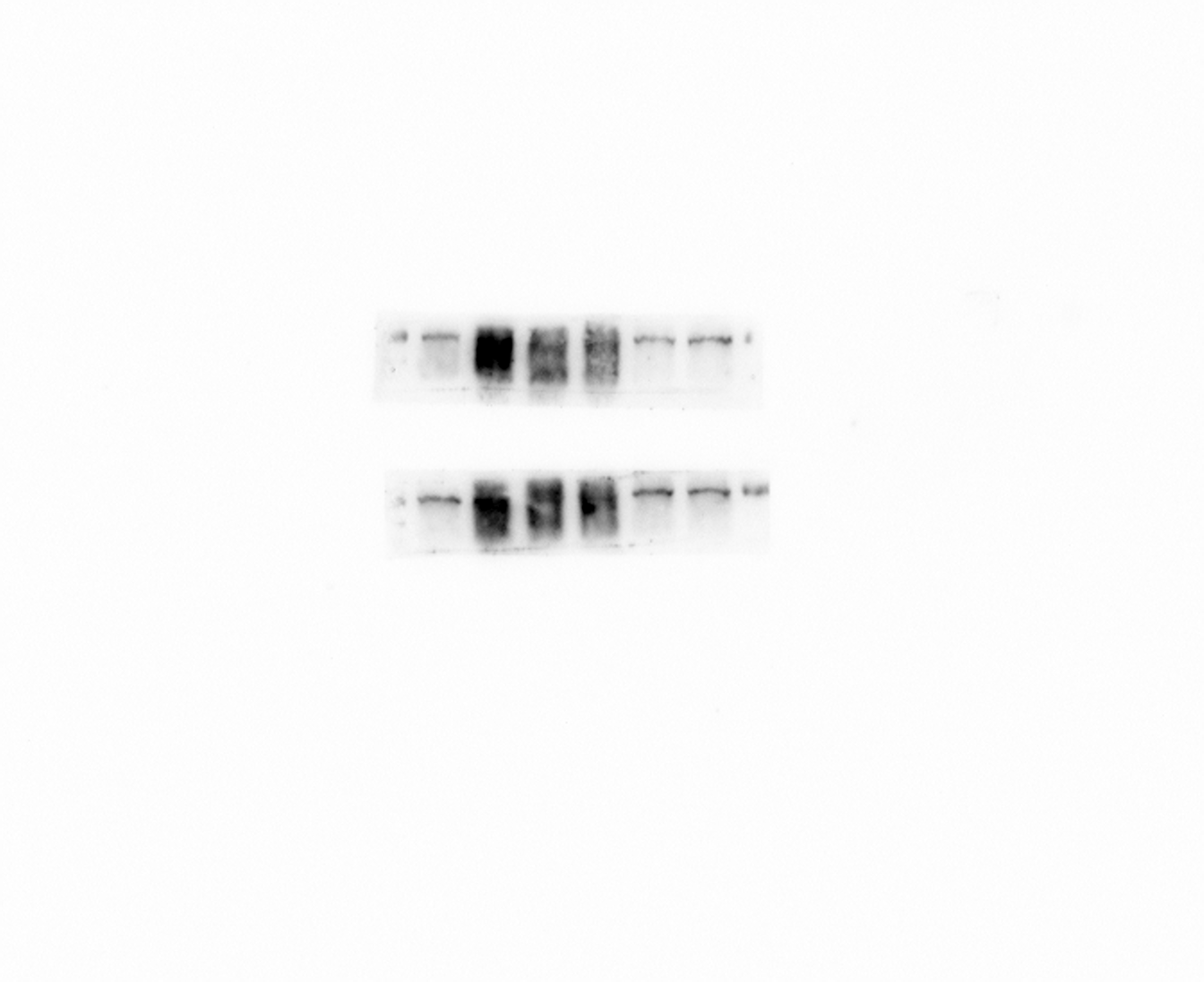

Supplement: Supplementary file 1 [file DataSheet1.ZIP › data/2022.8.30/P=PERK/30S.Tif]

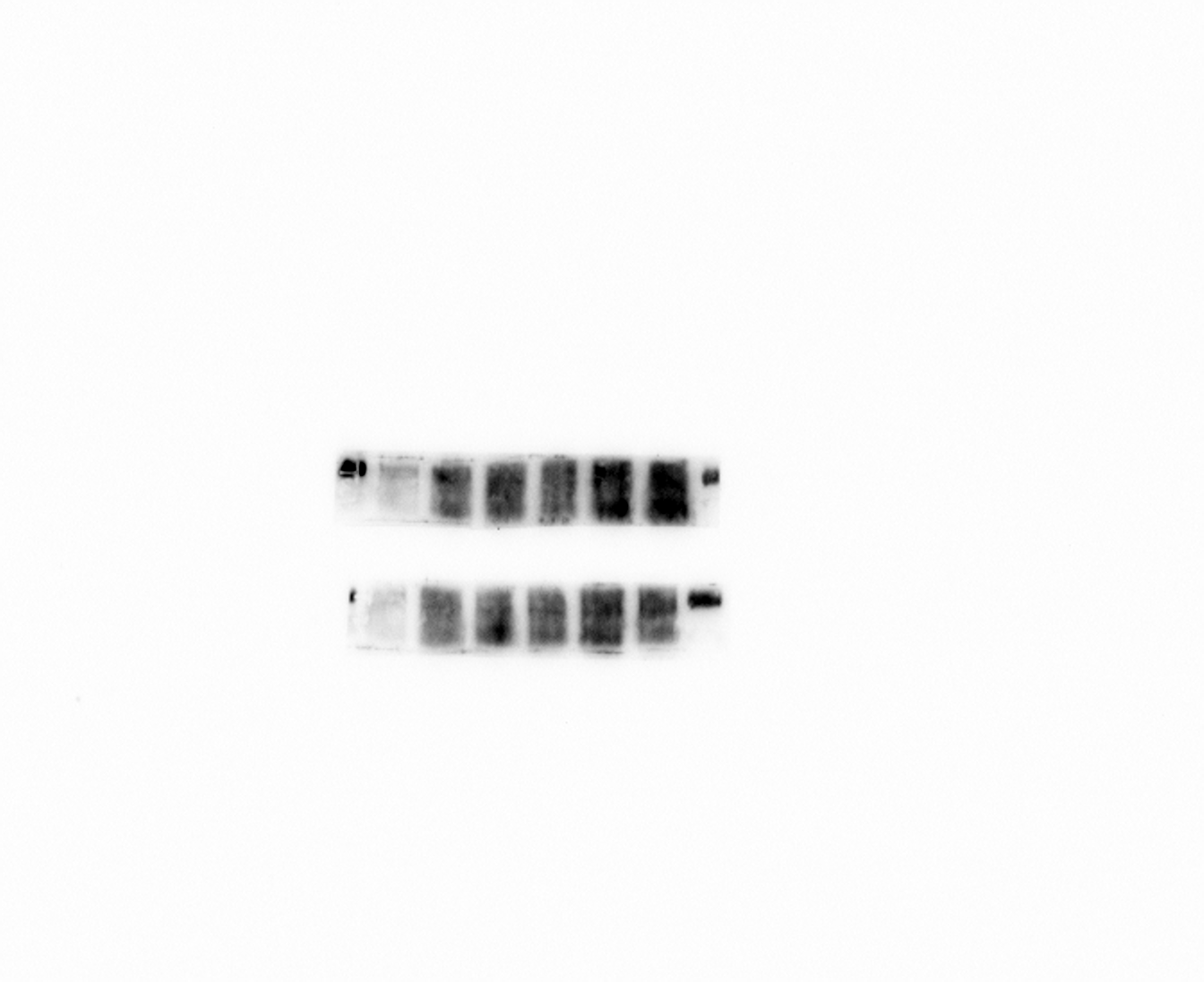

Supplement: Supplementary file 1 [file DataSheet1.ZIP › data/2022.8.30/sirt1/10s.Tif]

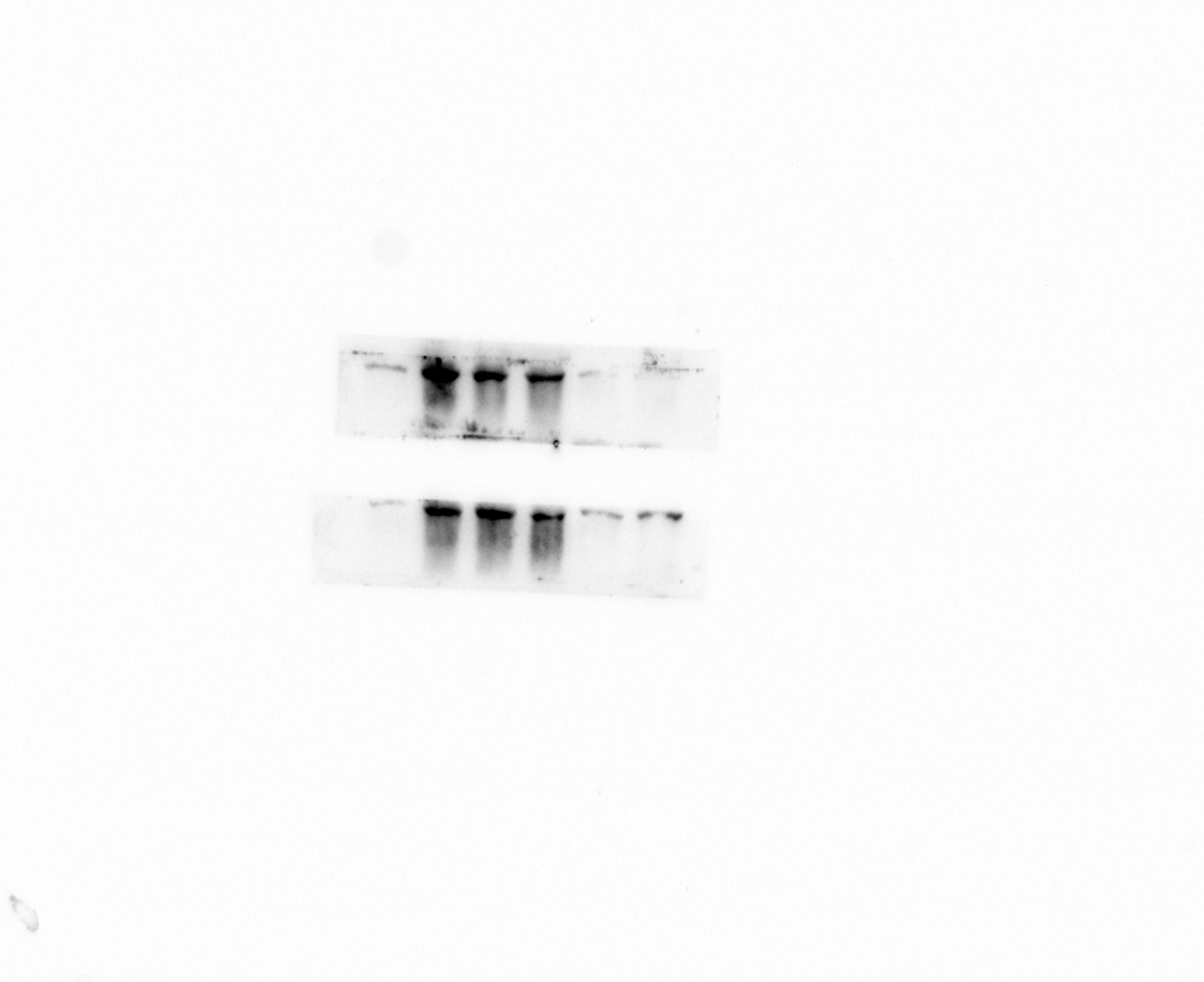

Supplement: Supplementary file 1 [file DataSheet1.ZIP › data/2022.8.30/sirt1/30s.Tif]

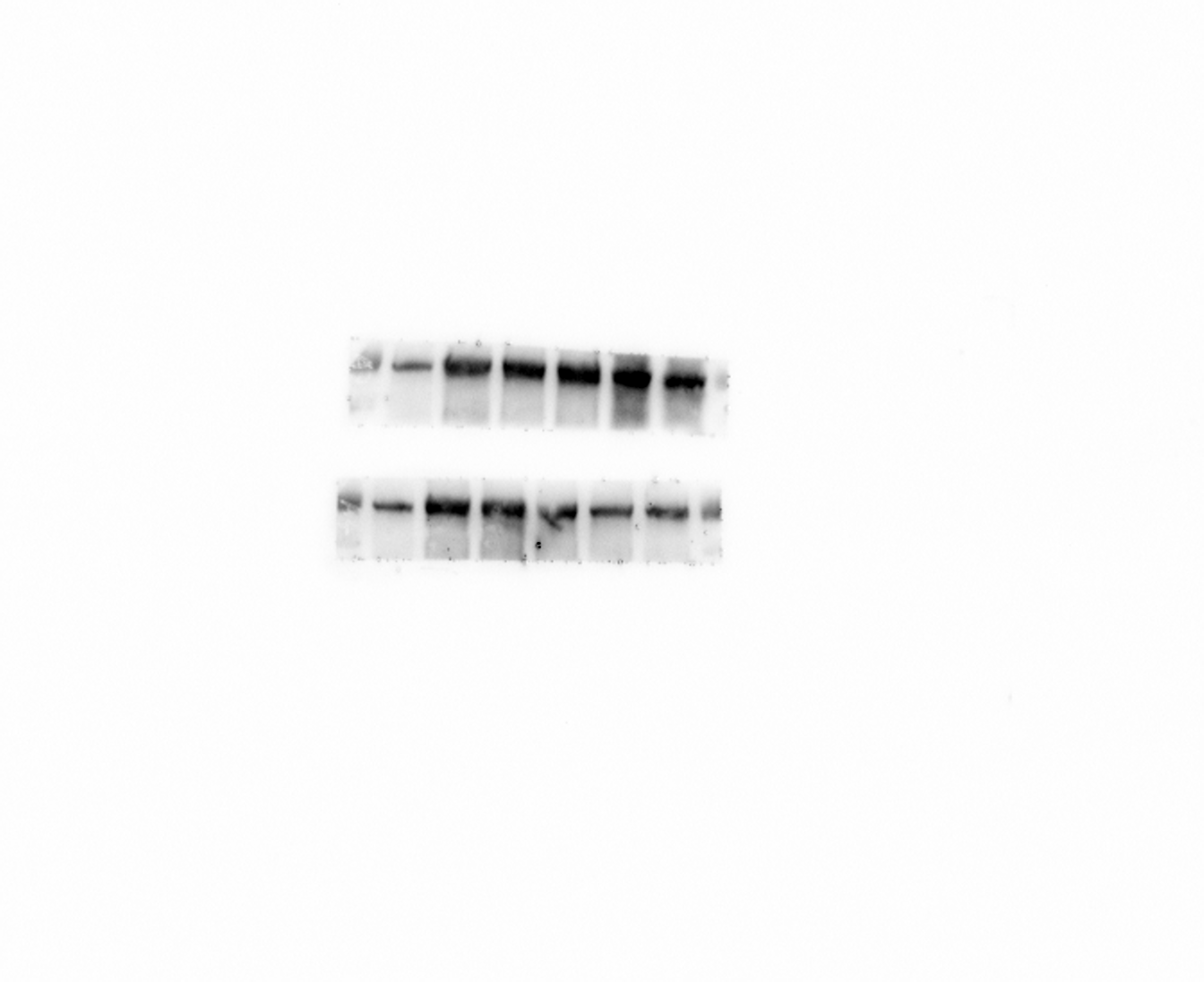

Supplement: Supplementary file 1 [file DataSheet1.ZIP › data/2022.9.15/CHOP/10S.1.Tif]

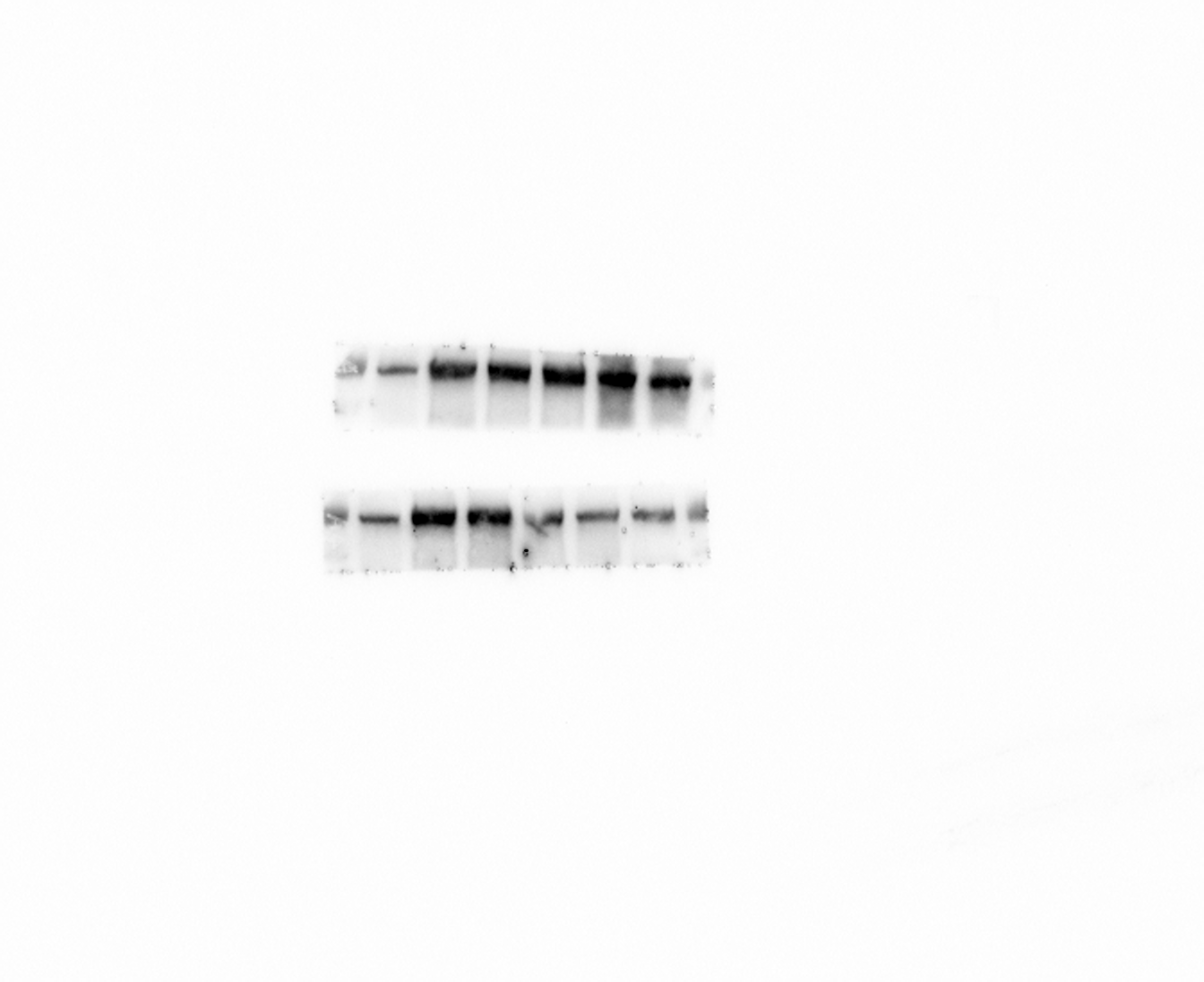

Supplement: Supplementary file 1 [file DataSheet1.ZIP › data/2022.9.15/CHOP/10S.Tif]

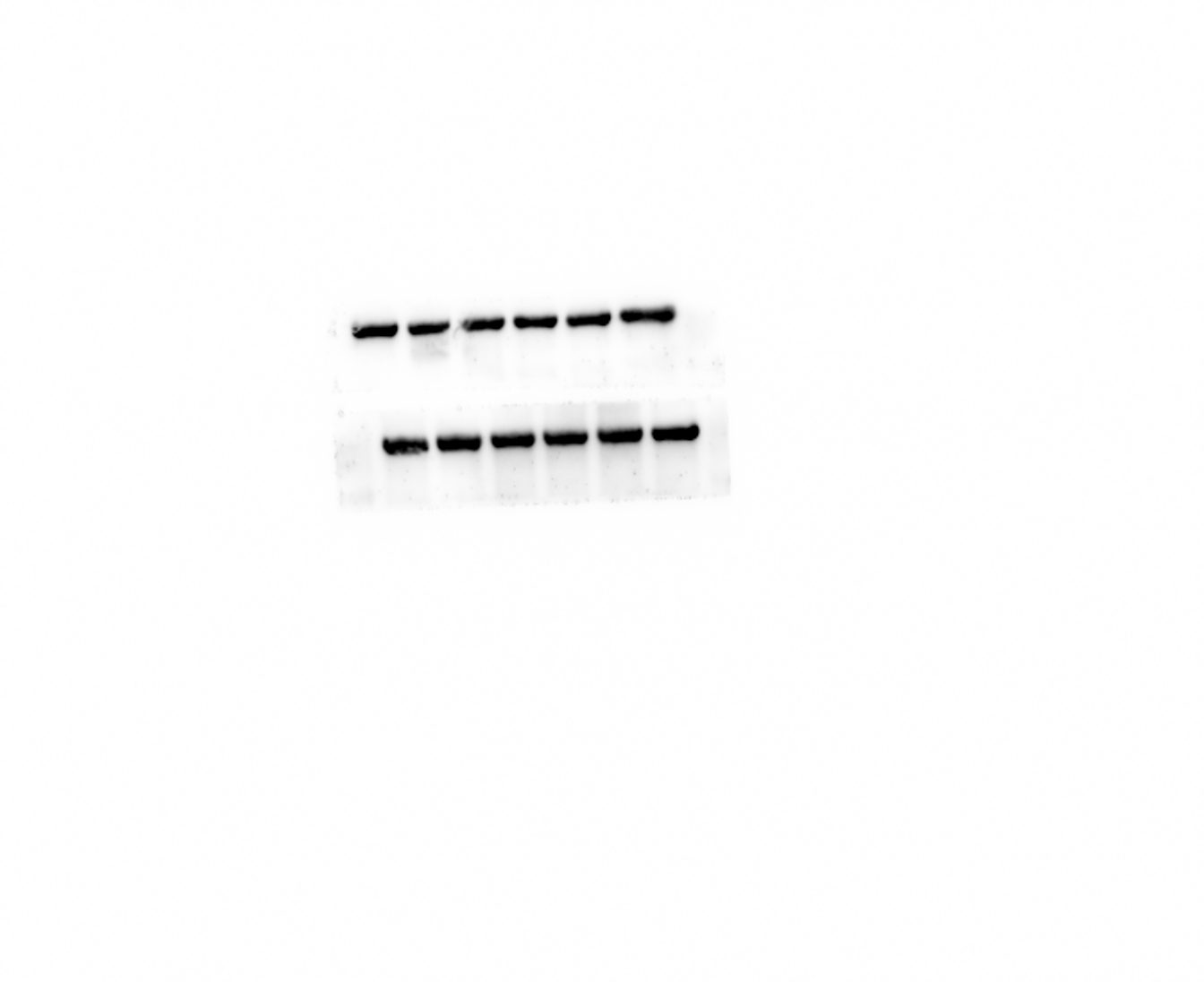

Supplement: Supplementary file 1 [file DataSheet1.ZIP › data/2022.9.15/GAPDH/10s.1.Tif]

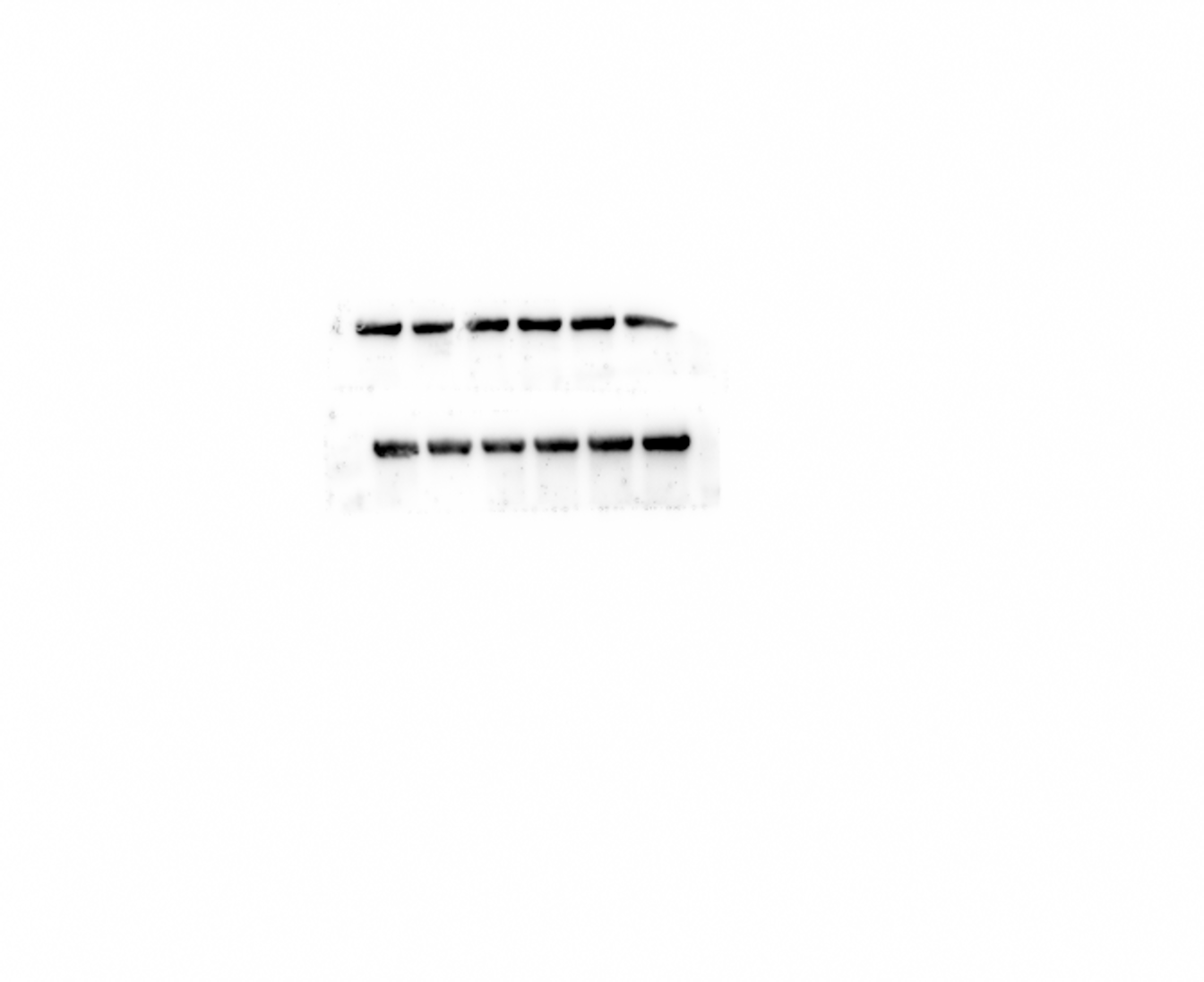

Supplement: Supplementary file 1 [file DataSheet1.ZIP › data/2022.9.15/GAPDH/10s.Tif]

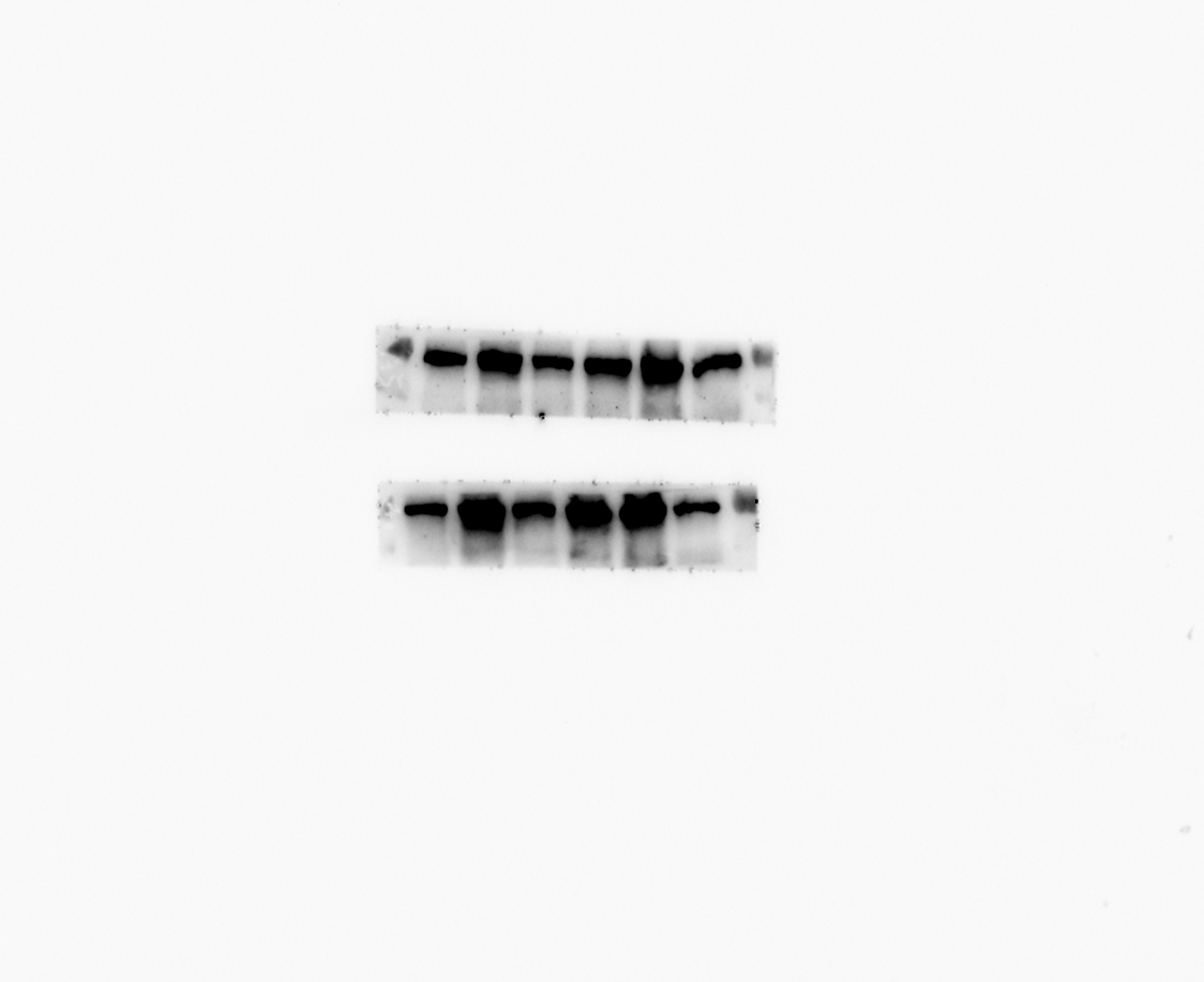

Supplement: Supplementary file 1 [file DataSheet1.ZIP › data/2022.9.15/GRP78/10S.Tif]

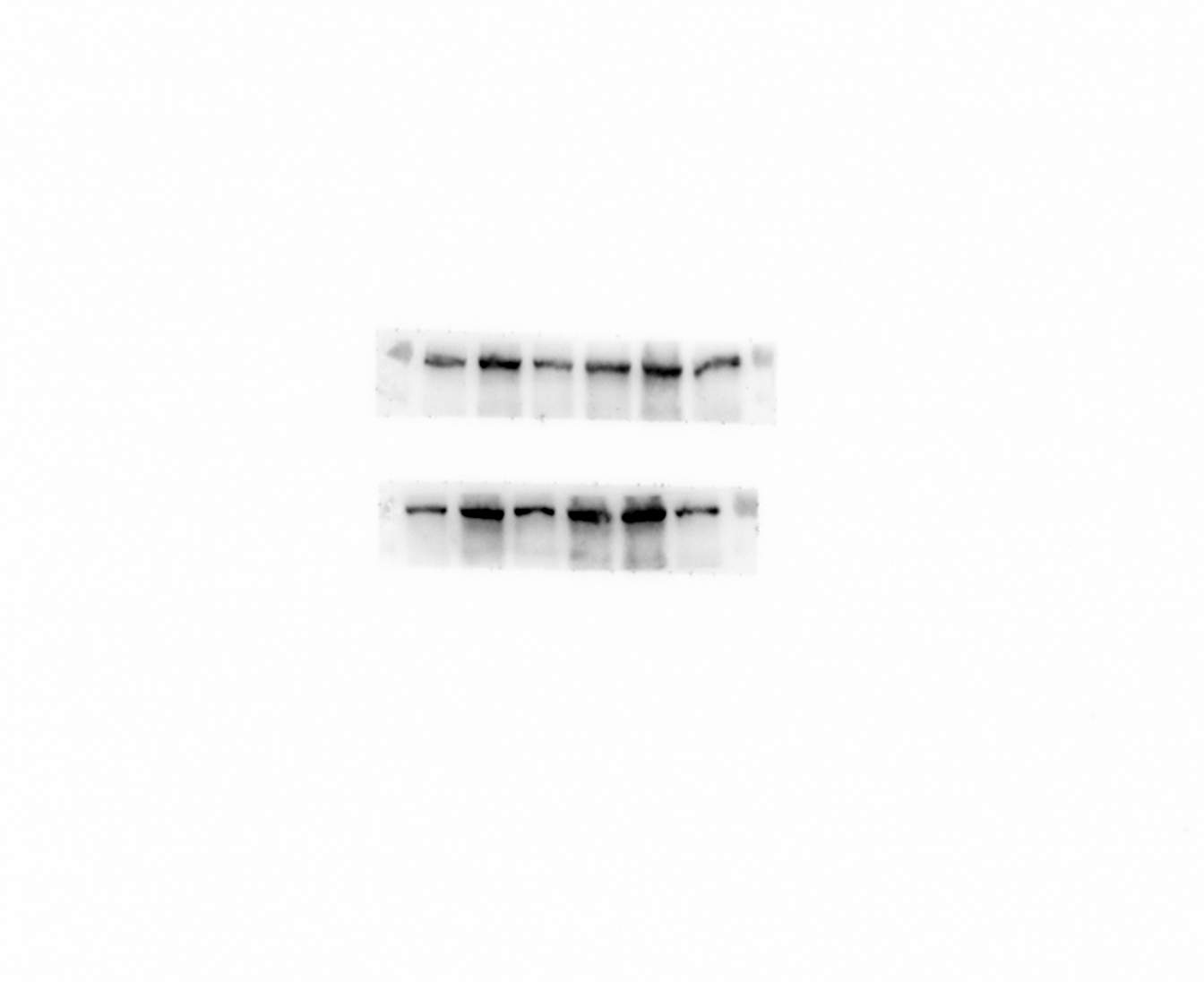

Supplement: Supplementary file 1 [file DataSheet1.ZIP › data/2022.9.15/GRP78/5S.Tif]

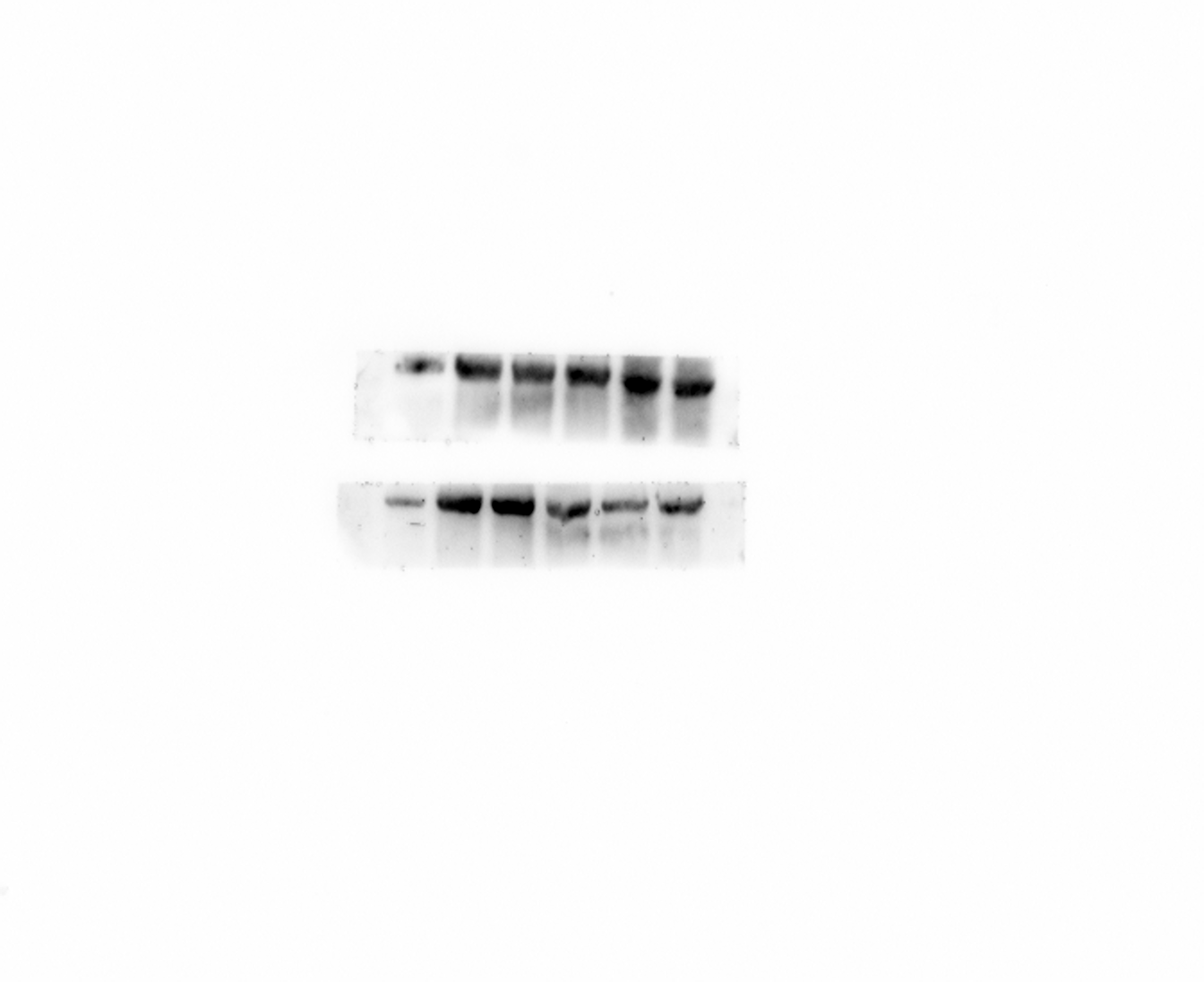

Supplement: Supplementary file 1 [file DataSheet1.ZIP › data/2022.9.15/p-eif/10s.1.Tif]

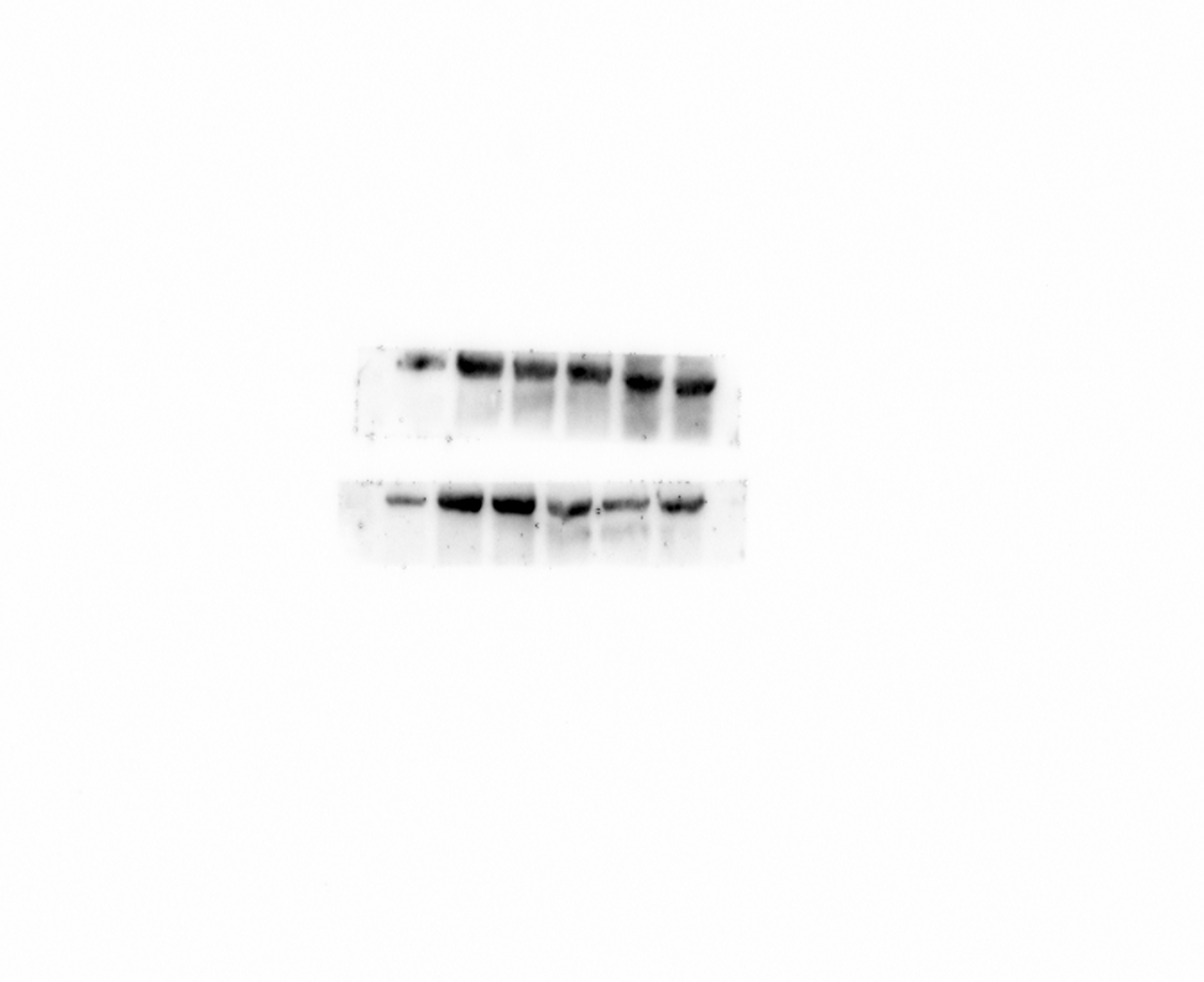

Supplement: Supplementary file 1 [file DataSheet1.ZIP › data/2022.9.15/p-eif/10s.Tif]

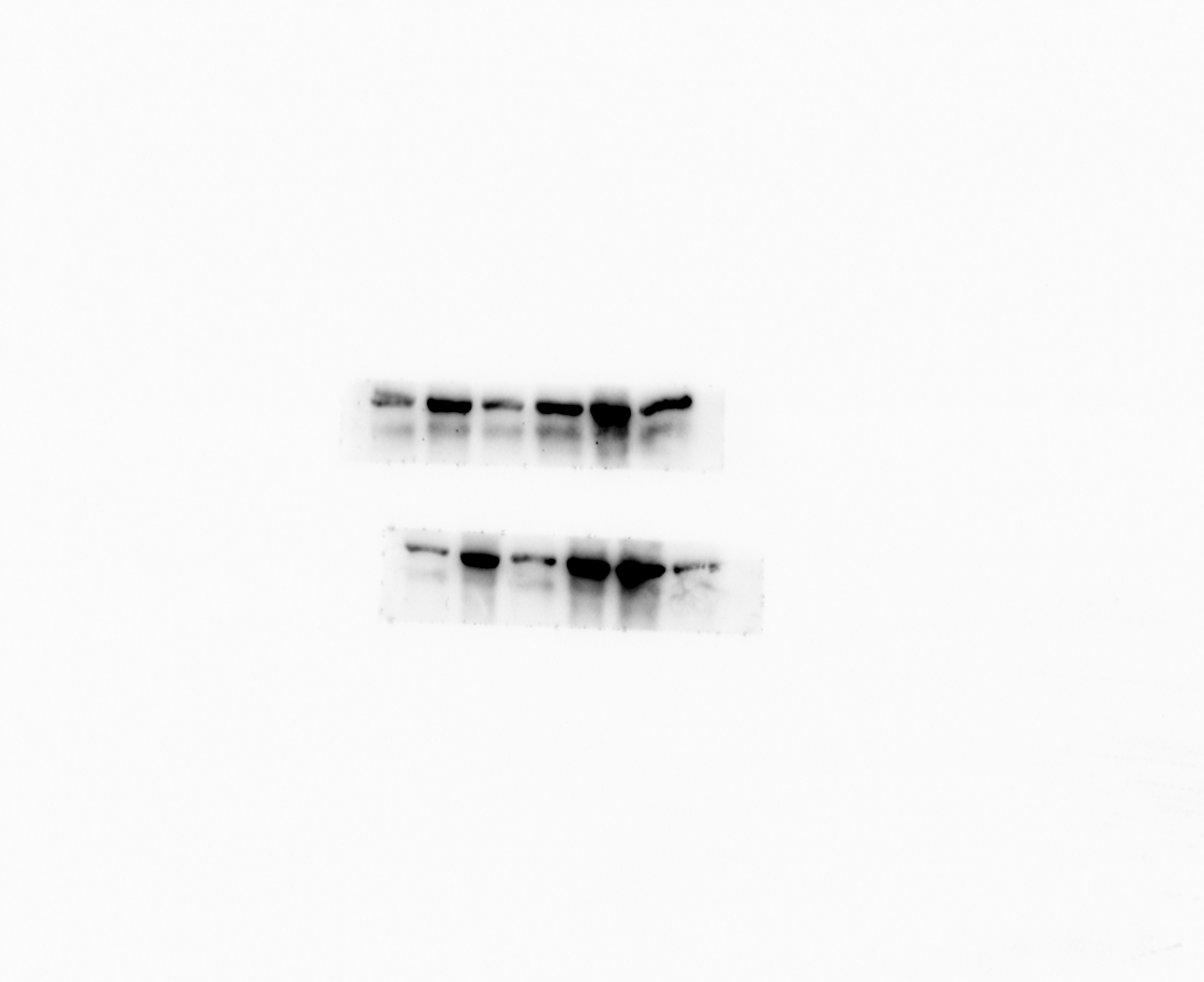

Supplement: Supplementary file 1 [file DataSheet1.ZIP › data/2022.9.15/p-perk/10s.Tif]

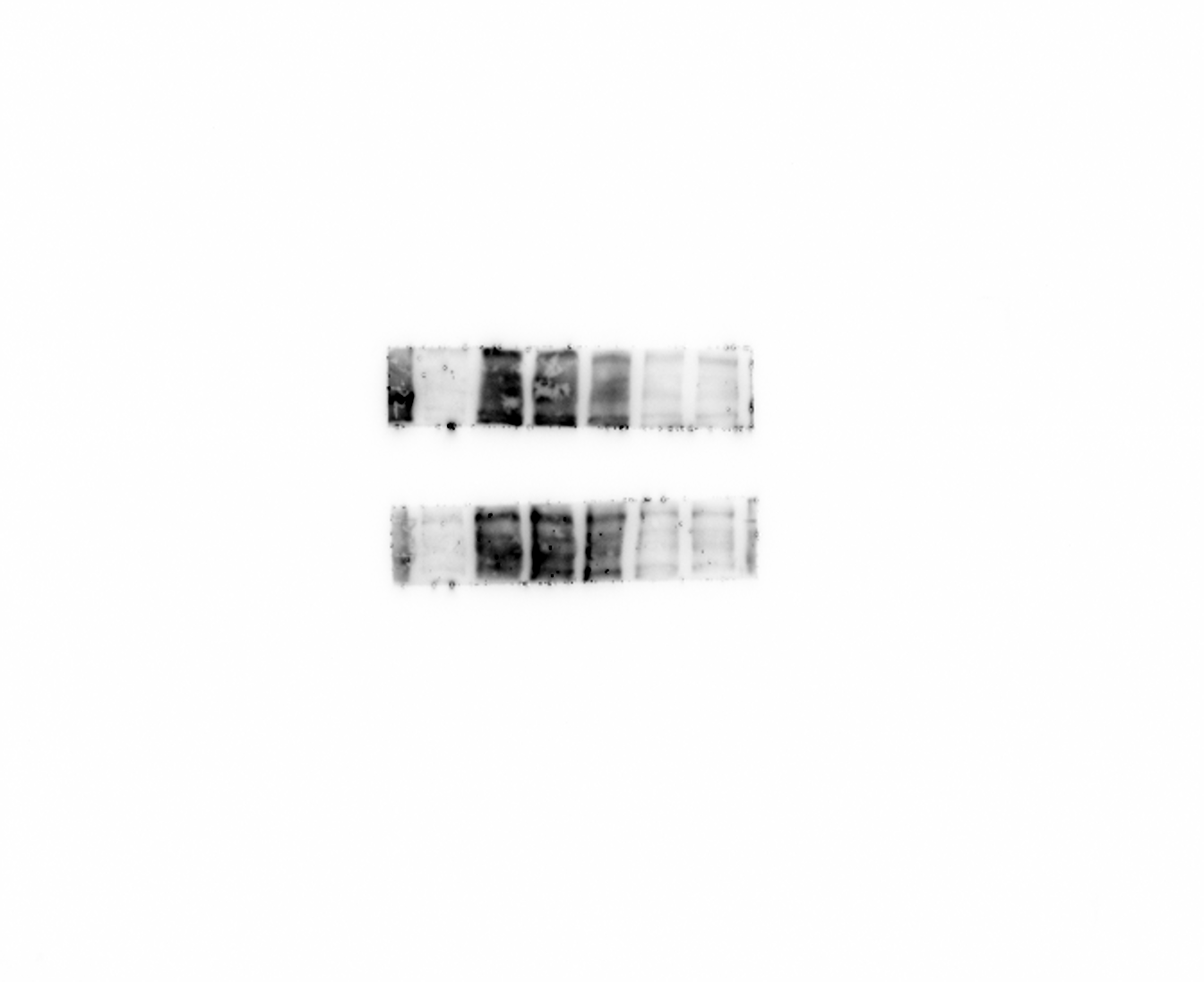

Supplement: Supplementary file 1 [file DataSheet1.ZIP › data/2022.9.2/chop/30s.Tif]

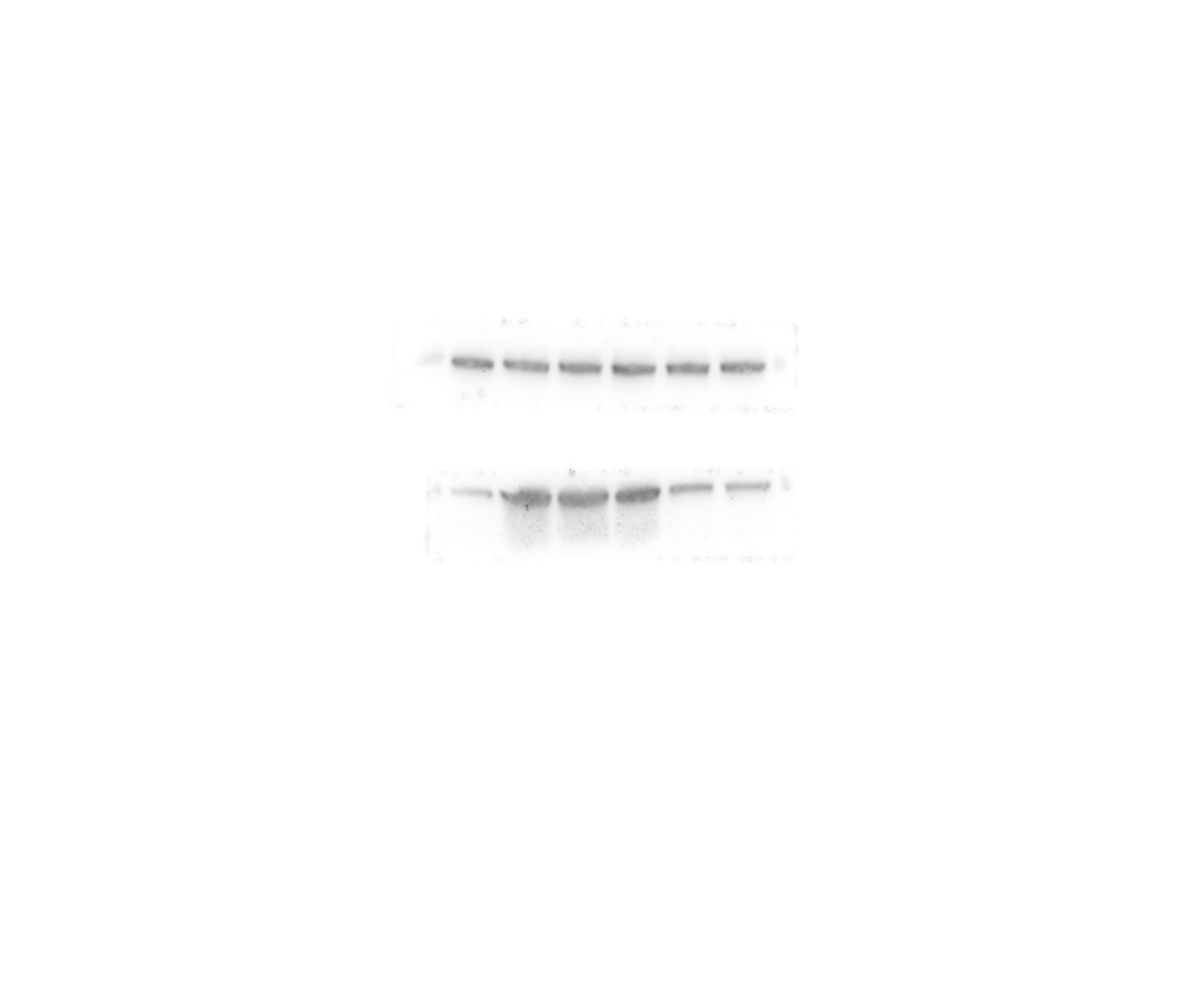

Supplement: Supplementary file 1 [file DataSheet1.ZIP › data/2022.9.2/GAPDH ATF4/10S.Tif]

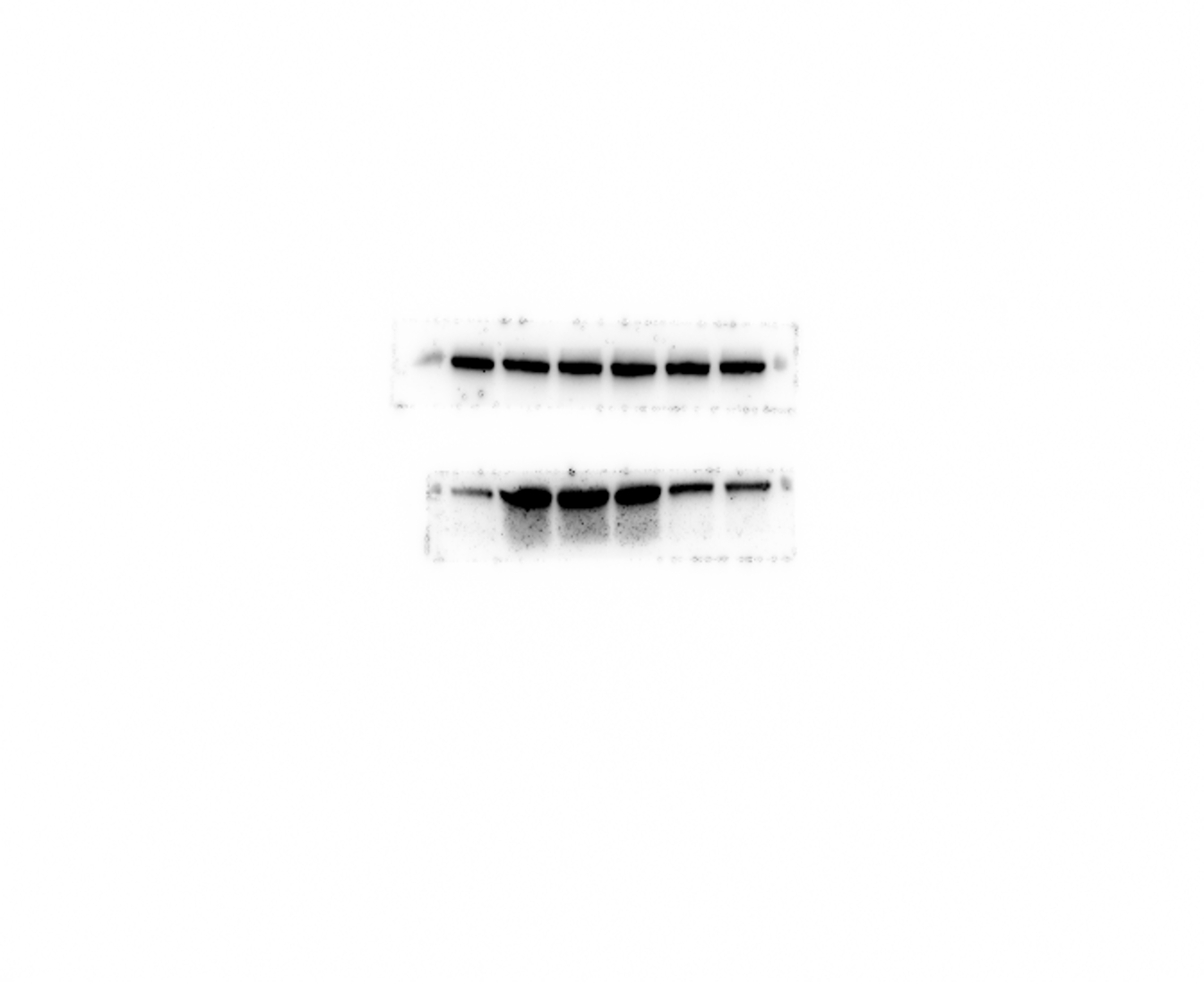

Supplement: Supplementary file 1 [file DataSheet1.ZIP › data/2022.9.2/GAPDH ATF4/30S.Tif]

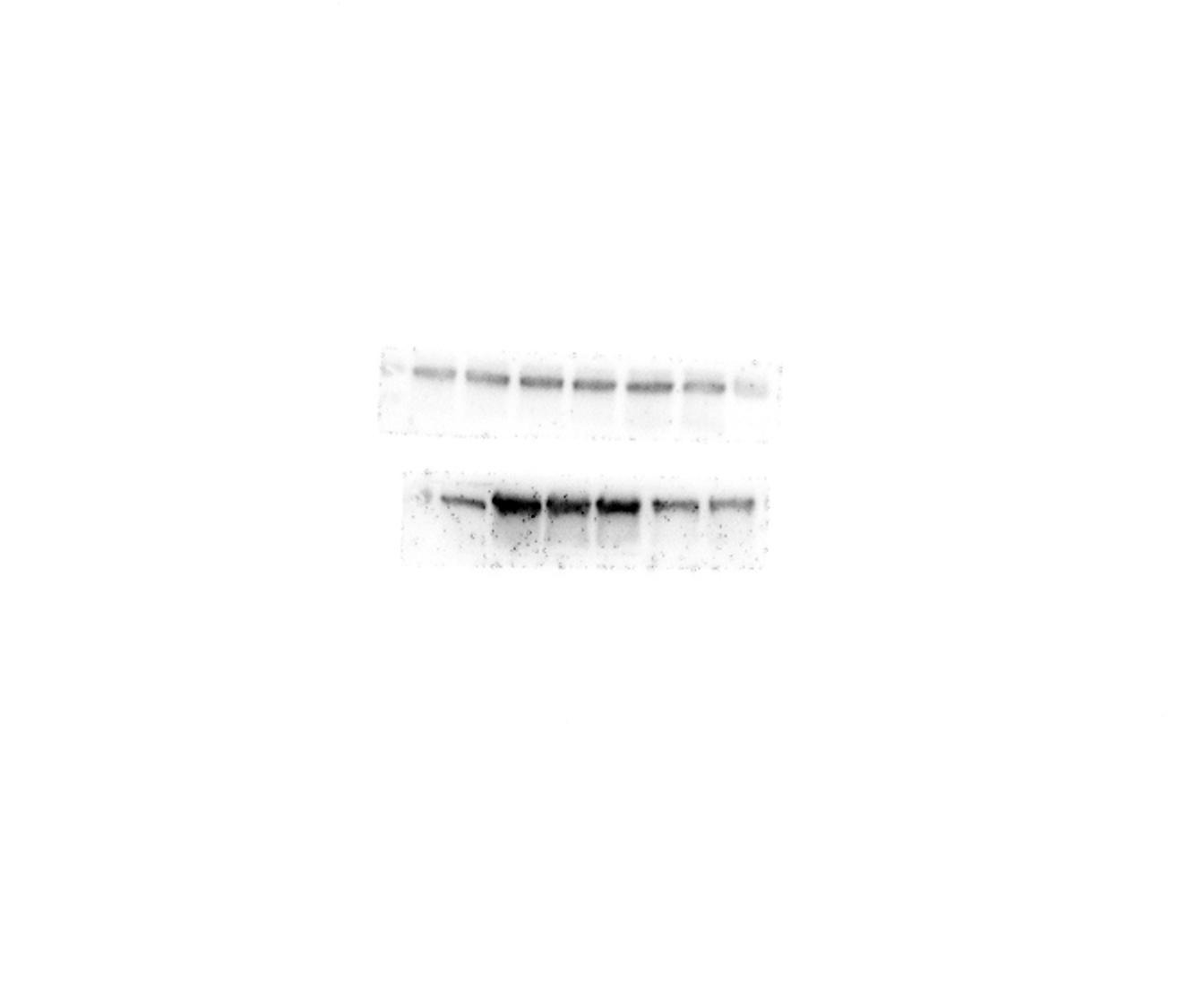

Supplement: Supplementary file 1 [file DataSheet1.ZIP › data/2022.9.2/GAPDH cd/3s.Tif]

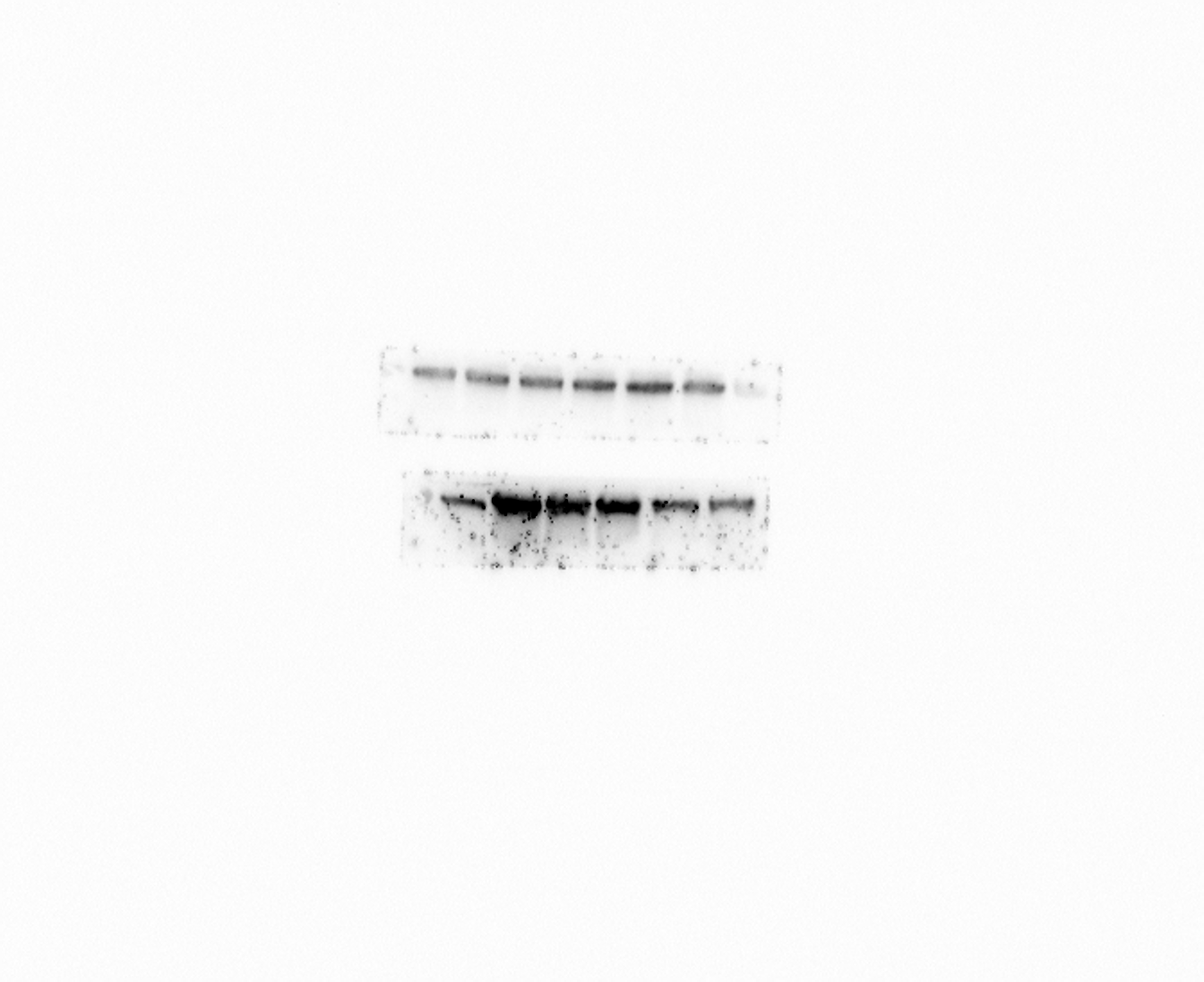

Supplement: Supplementary file 1 [file DataSheet1.ZIP › data/2022.9.2/GAPDH cd/5s.Tif]

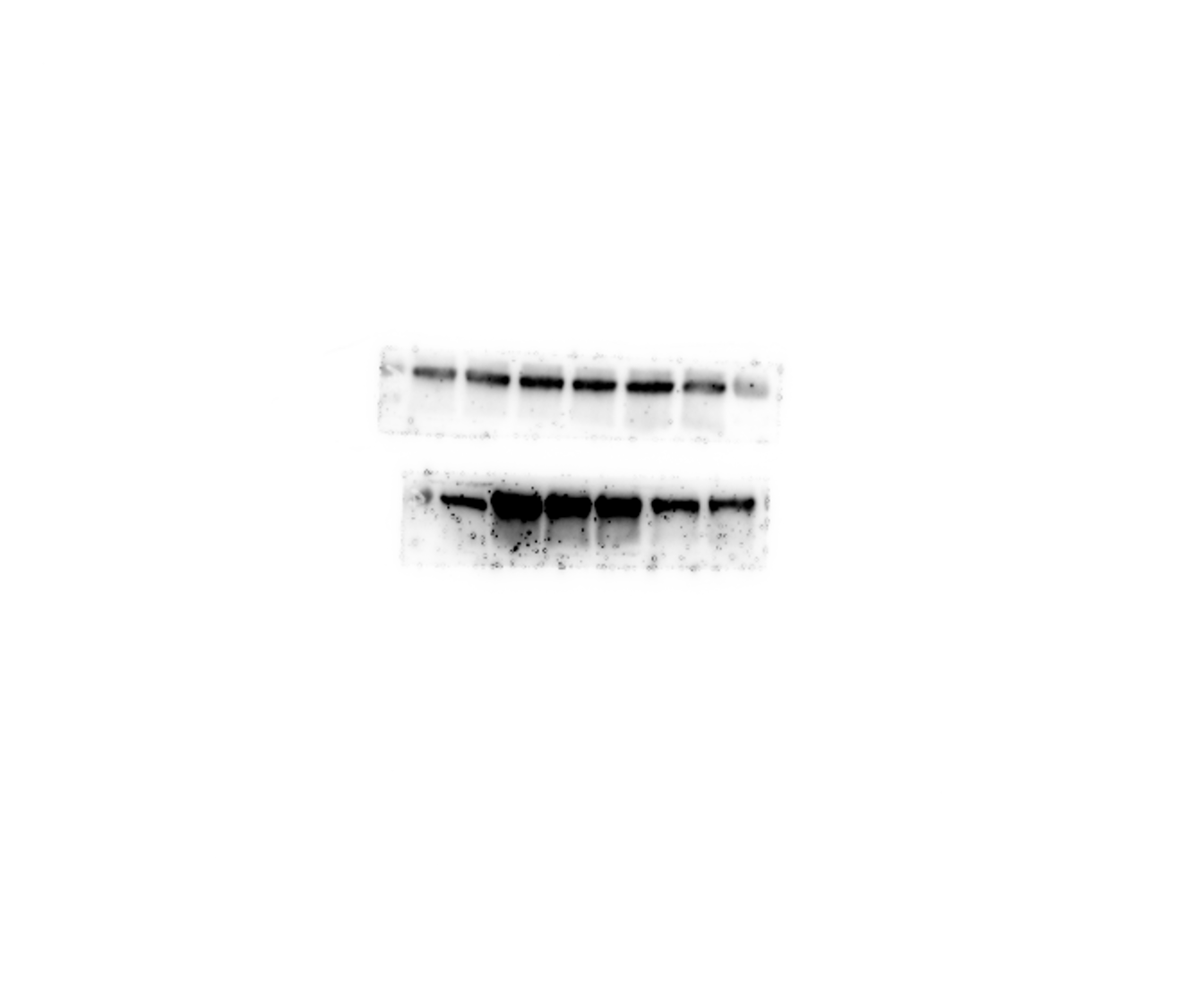

Supplement: Supplementary file 1 [file DataSheet1.ZIP › data/2022.9.2/GAPDH cd/80s.Tif]

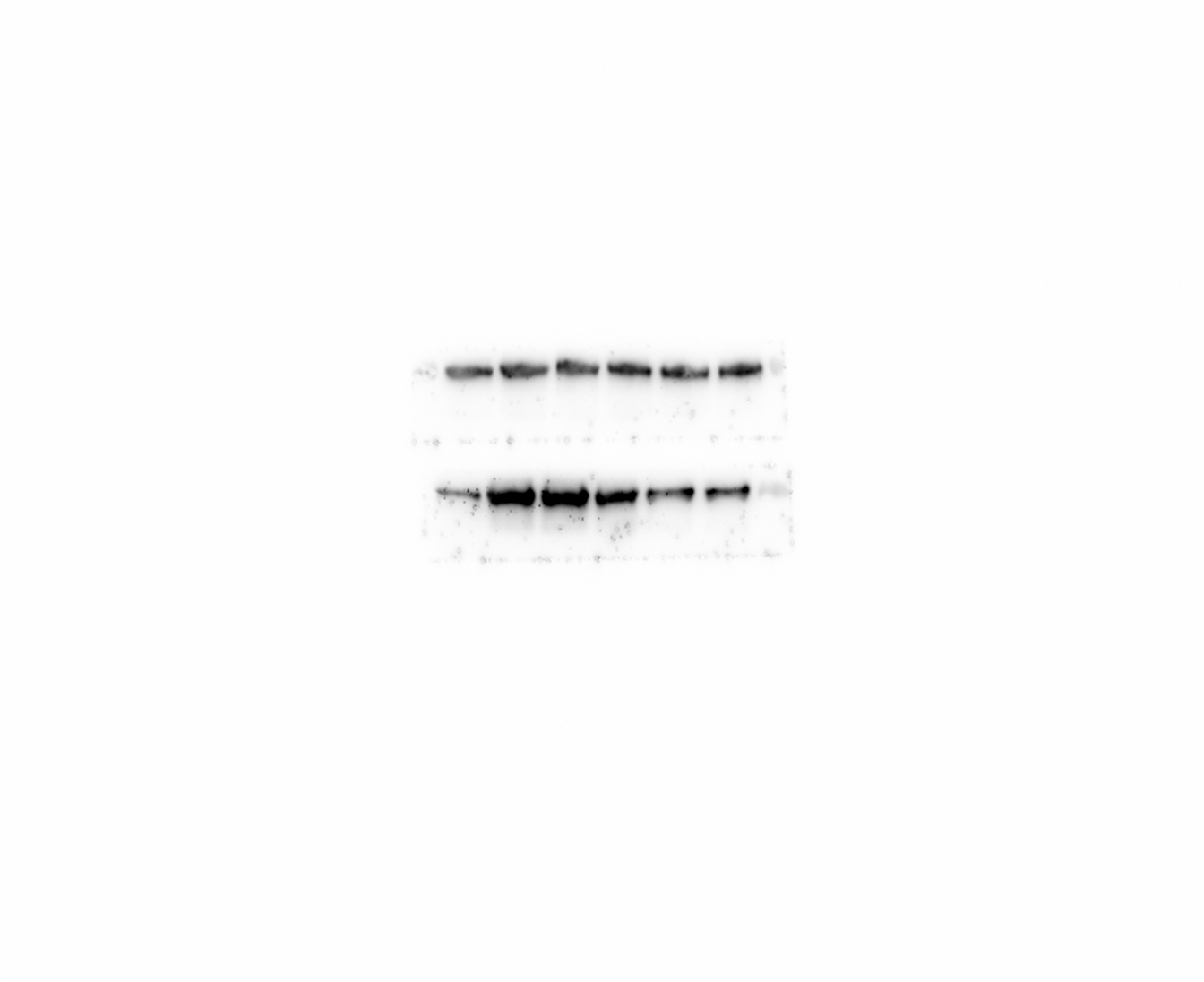

Supplement: Supplementary file 1 [file DataSheet1.ZIP › data/2022.9.2/GAPDH P-PERK/10S.Tif]

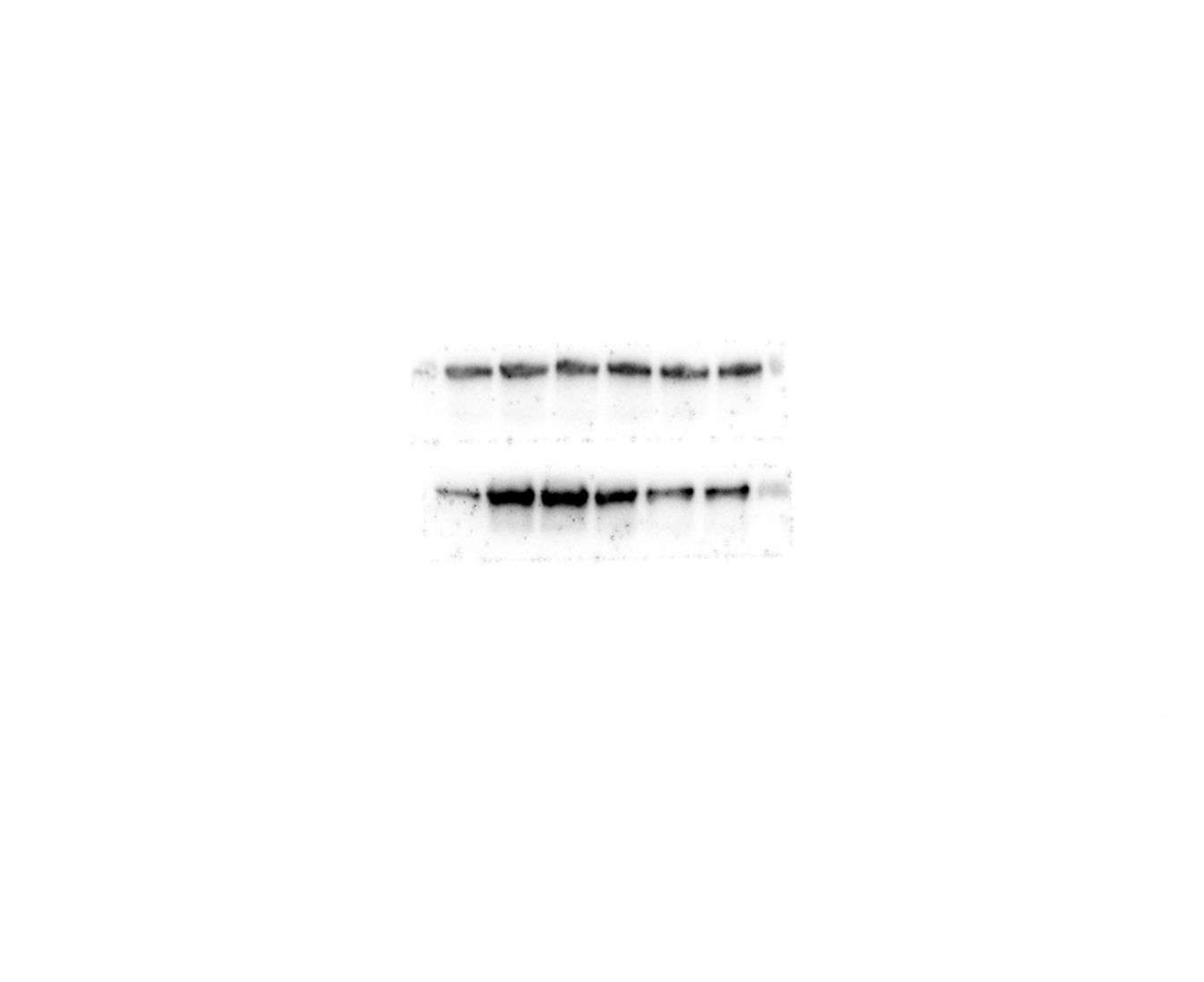

Supplement: Supplementary file 1 [file DataSheet1.ZIP › data/2022.9.2/GAPDH P-PERK/3S.Tif]

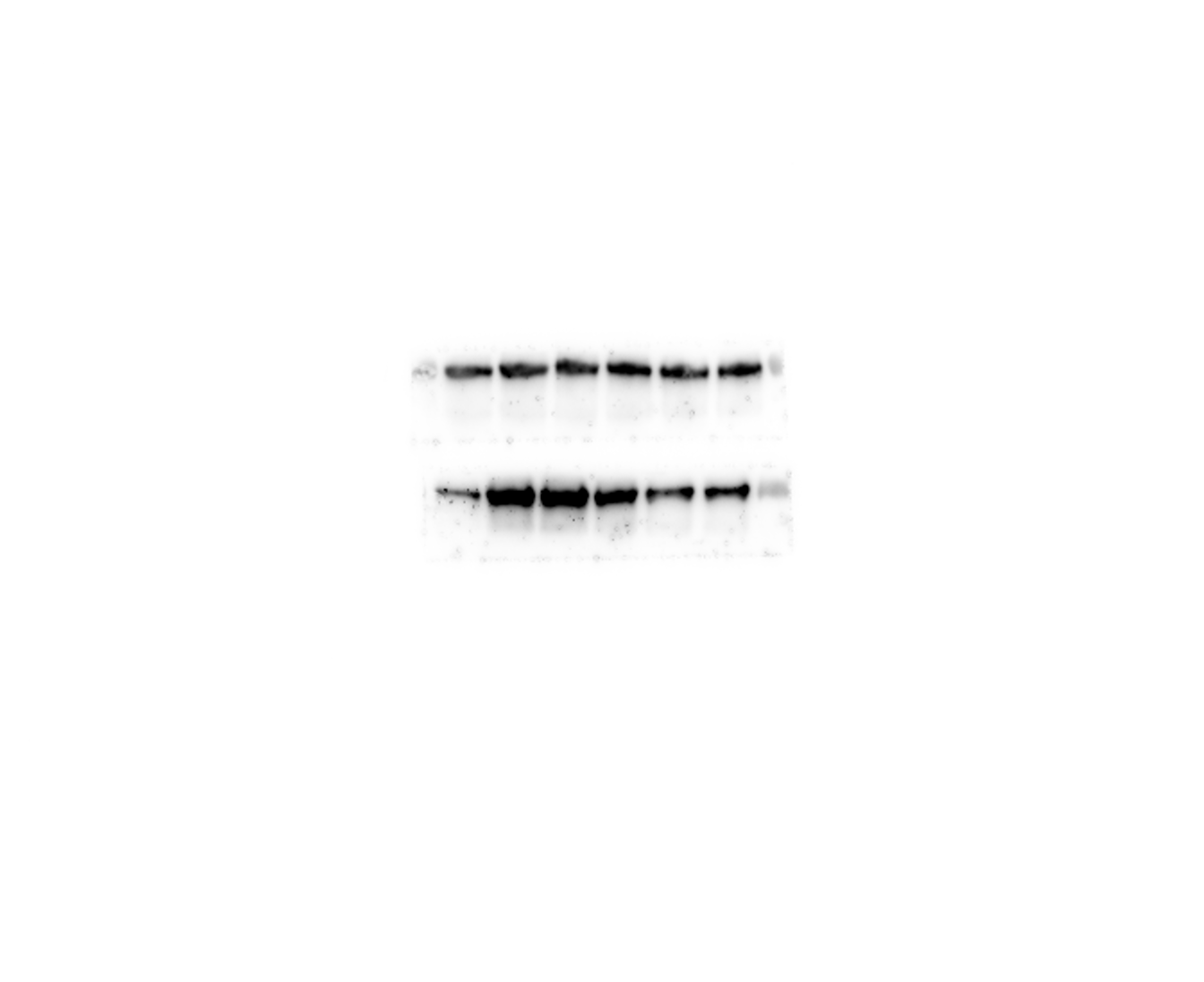

Supplement: Supplementary file 1 [file DataSheet1.ZIP › data/2022.9.2/GAPDH P-PERK/40S.Tif]

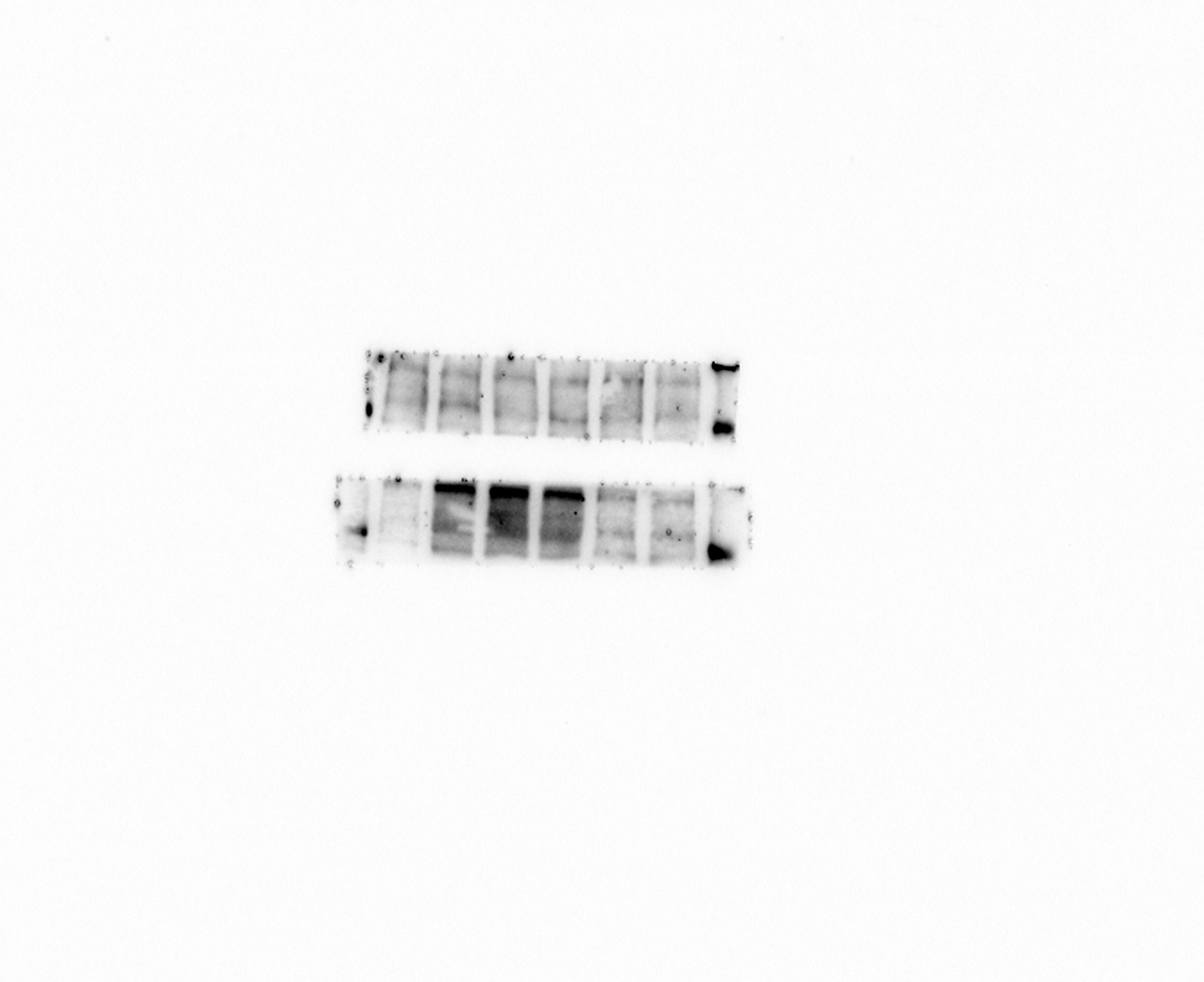

Supplement: Supplementary file 1 [file DataSheet1.ZIP › data/2022.9.2/grp78/10s.Tif]

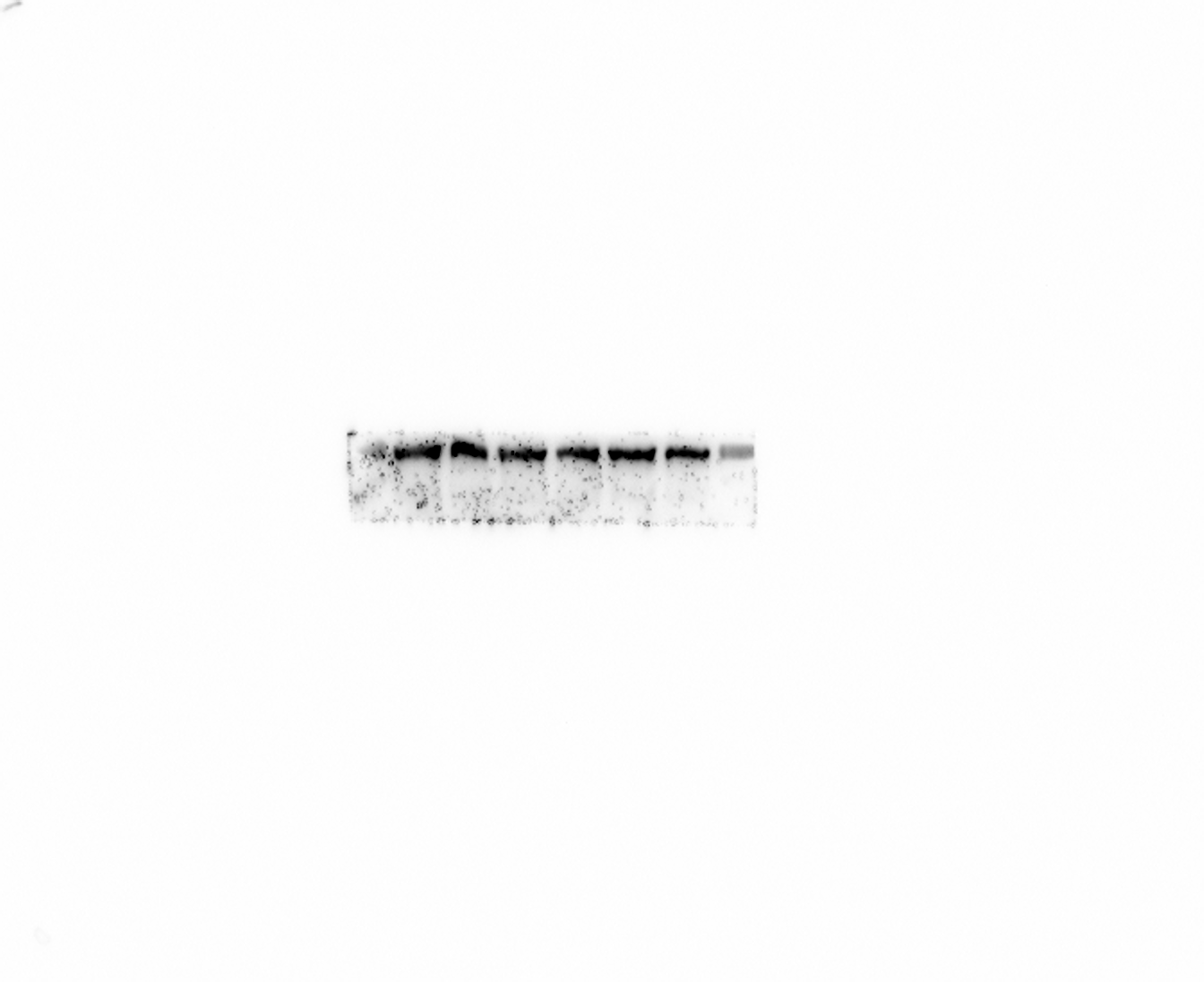

Supplement: Supplementary file 1 [file DataSheet1.ZIP › data/2022.9.2/perk/10s.Tif]

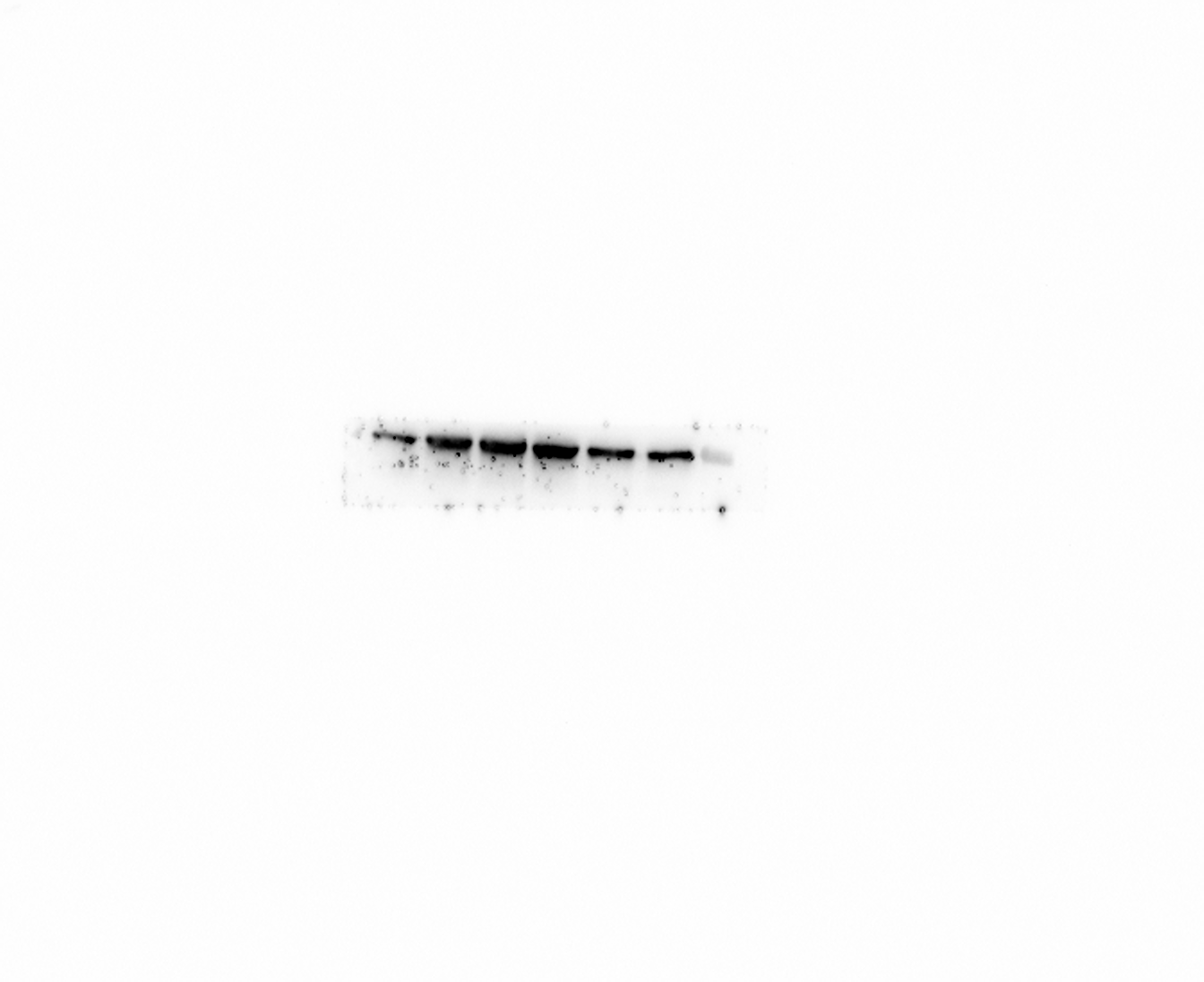

Supplement: Supplementary file 1 [file DataSheet1.ZIP › data/2022.9.2/tnf-a/10s.Tif]

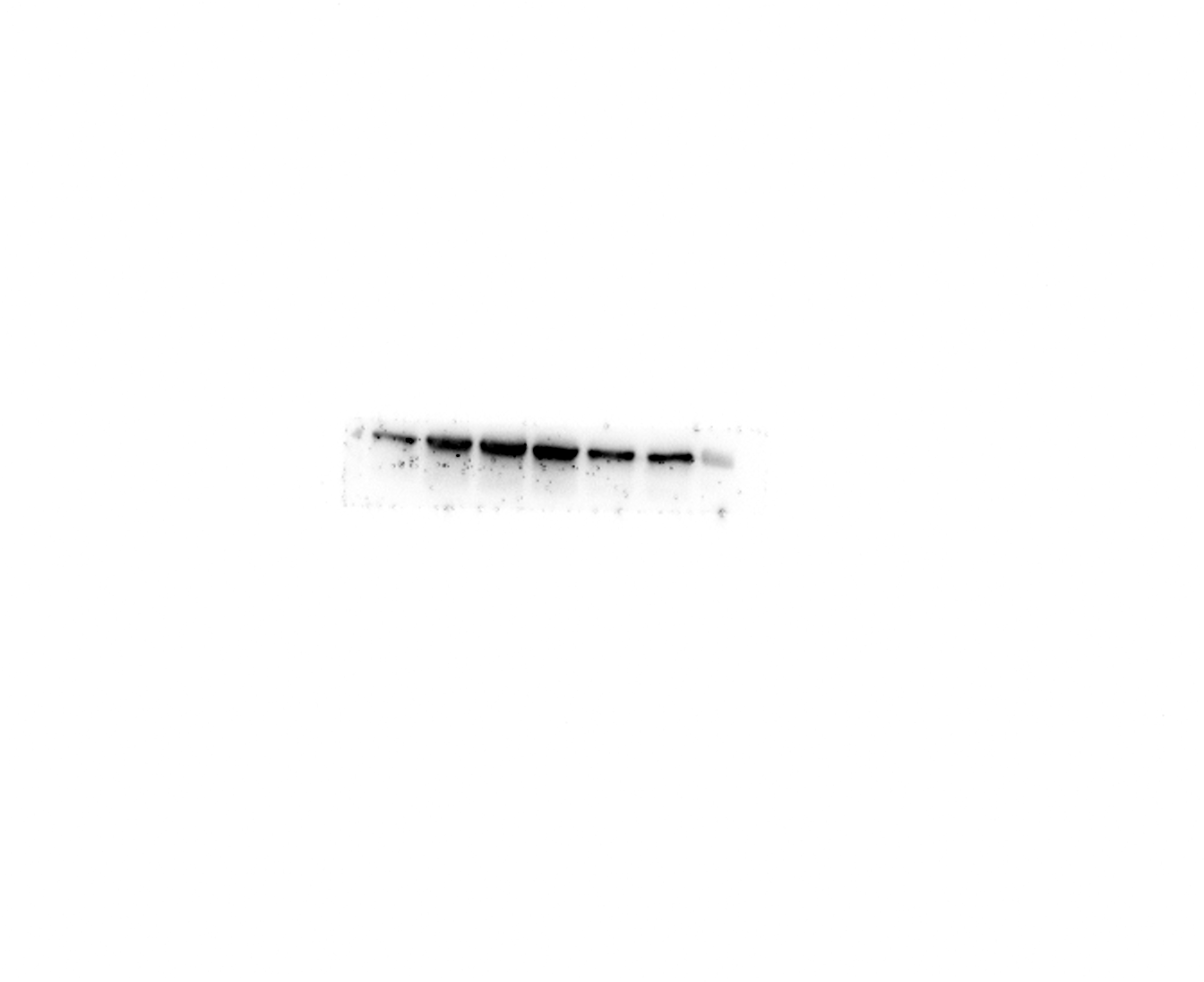

Supplement: Supplementary file 1 [file DataSheet1.ZIP › data/2022.9.2/tnf-a/5s.Tif]

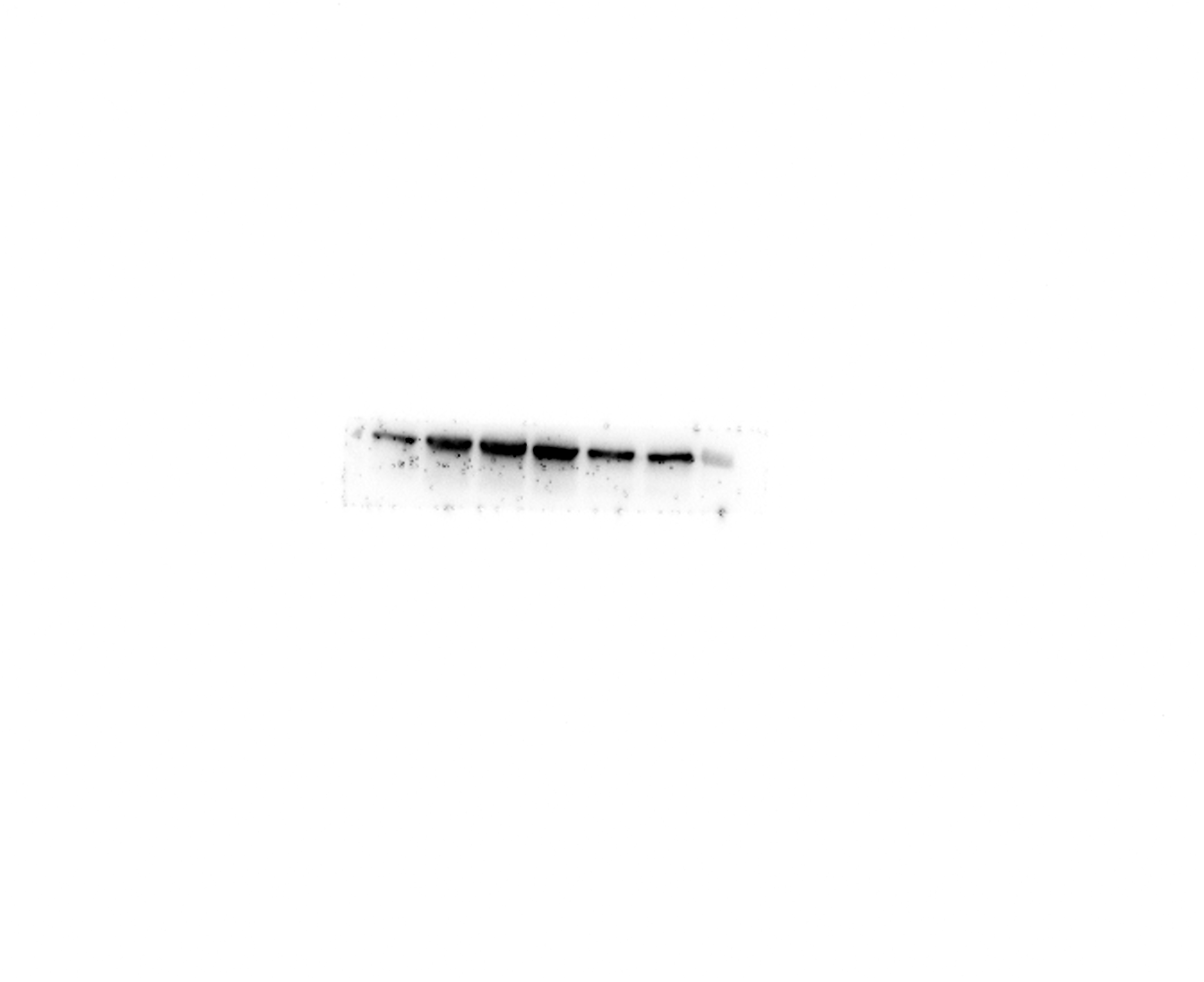

Supplement: Supplementary file 1 [file DataSheet1.ZIP › data/2022.9.2/tnf-a/6s.Tif]

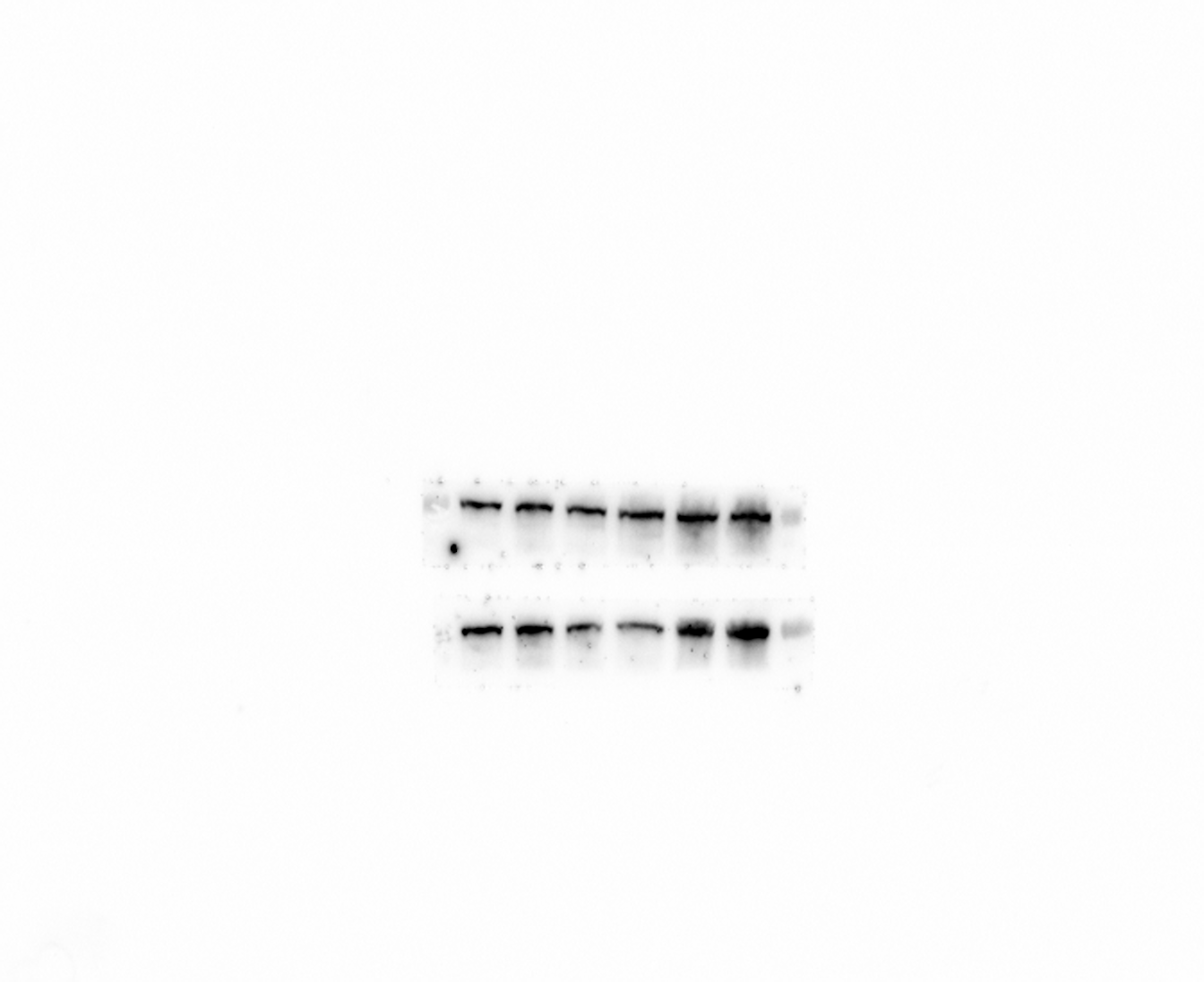

Supplement: Supplementary file 1 [file DataSheet1.ZIP › data/2022.9.6/ARG1/10S.Tif]

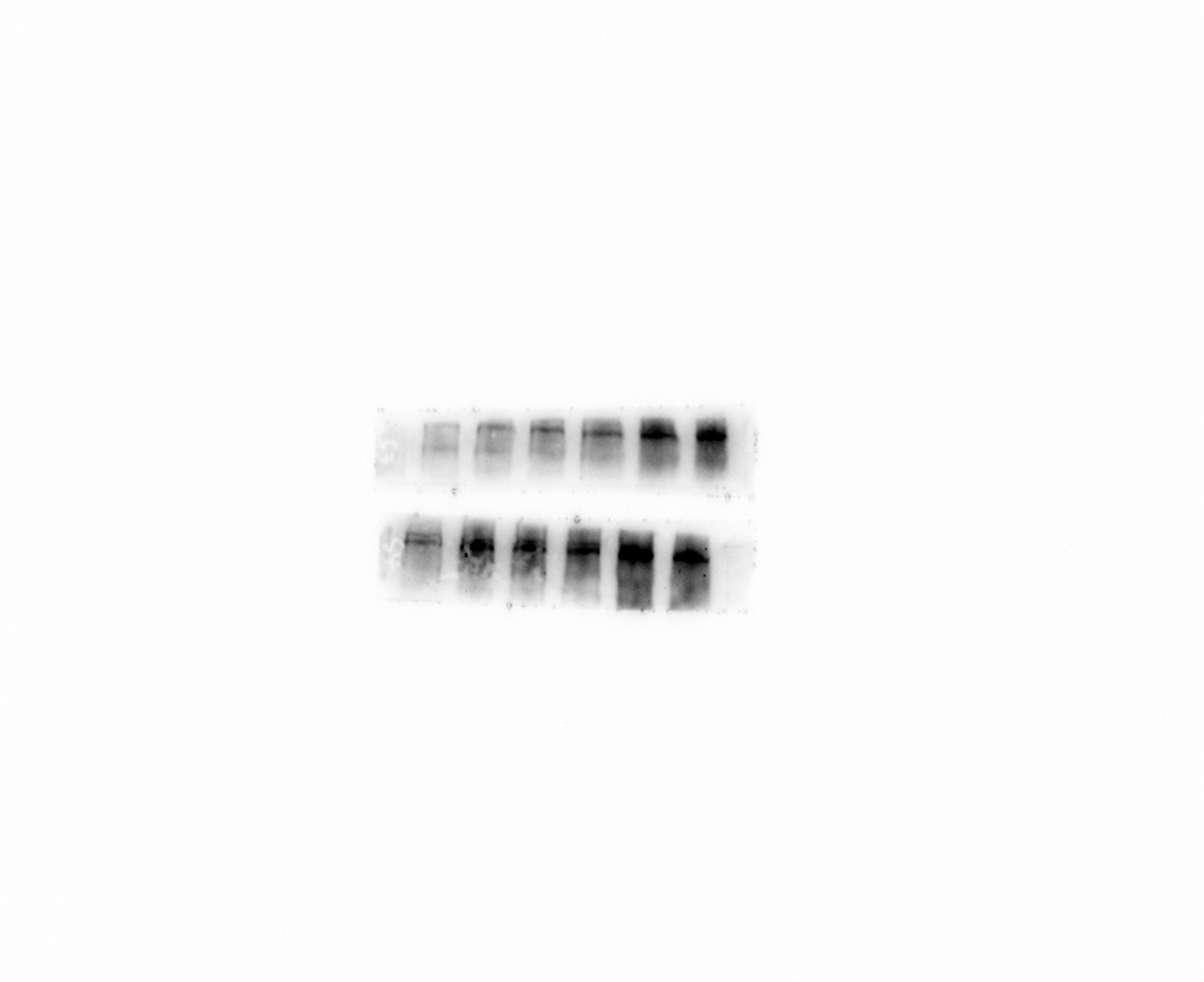

Supplement: Supplementary file 1 [file DataSheet1.ZIP › data/2022.9.6/CD206/10S.Tif]

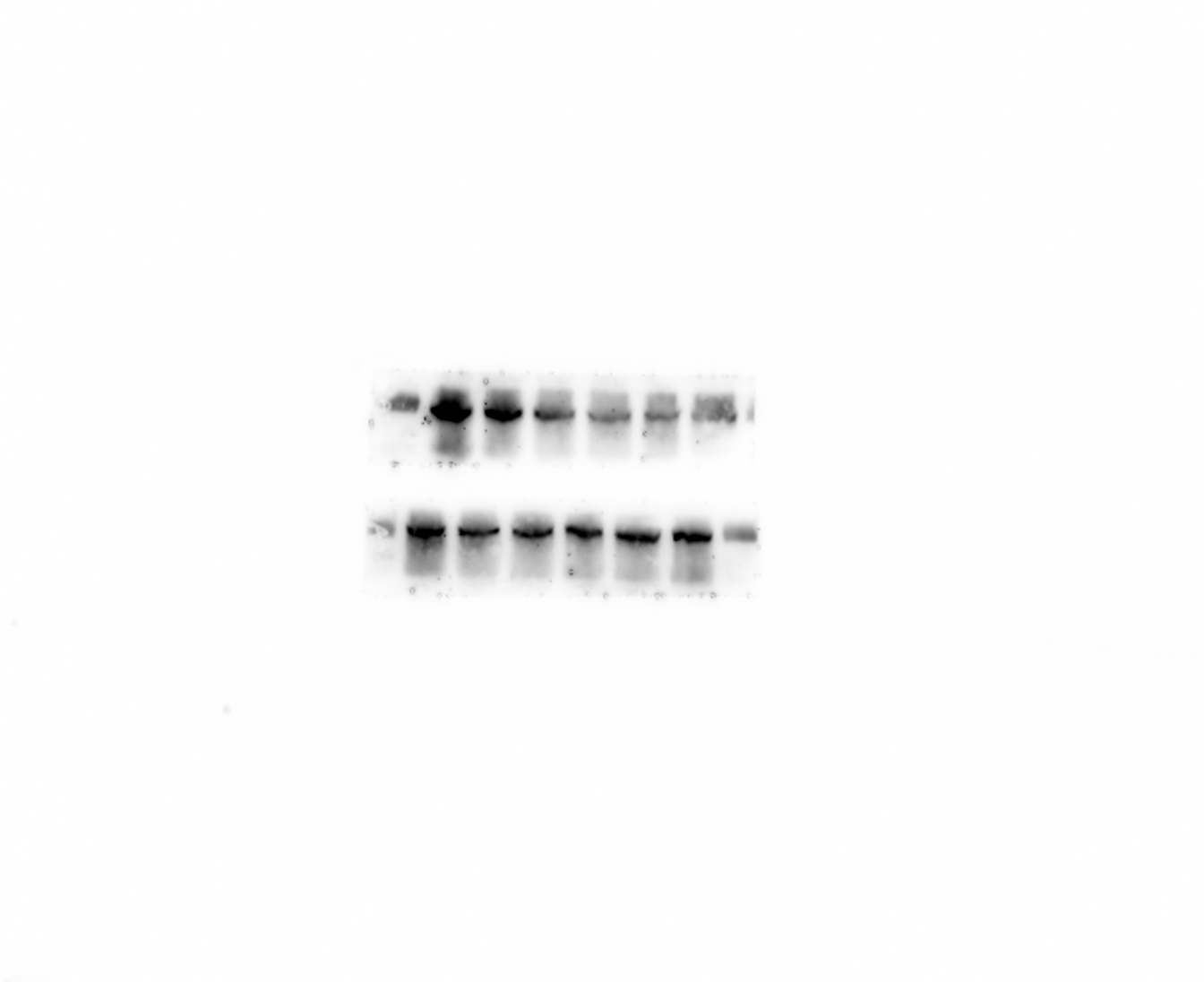

Supplement: Supplementary file 1 [file DataSheet1.ZIP › data/2022.9.6/EIF/60S.Tif]

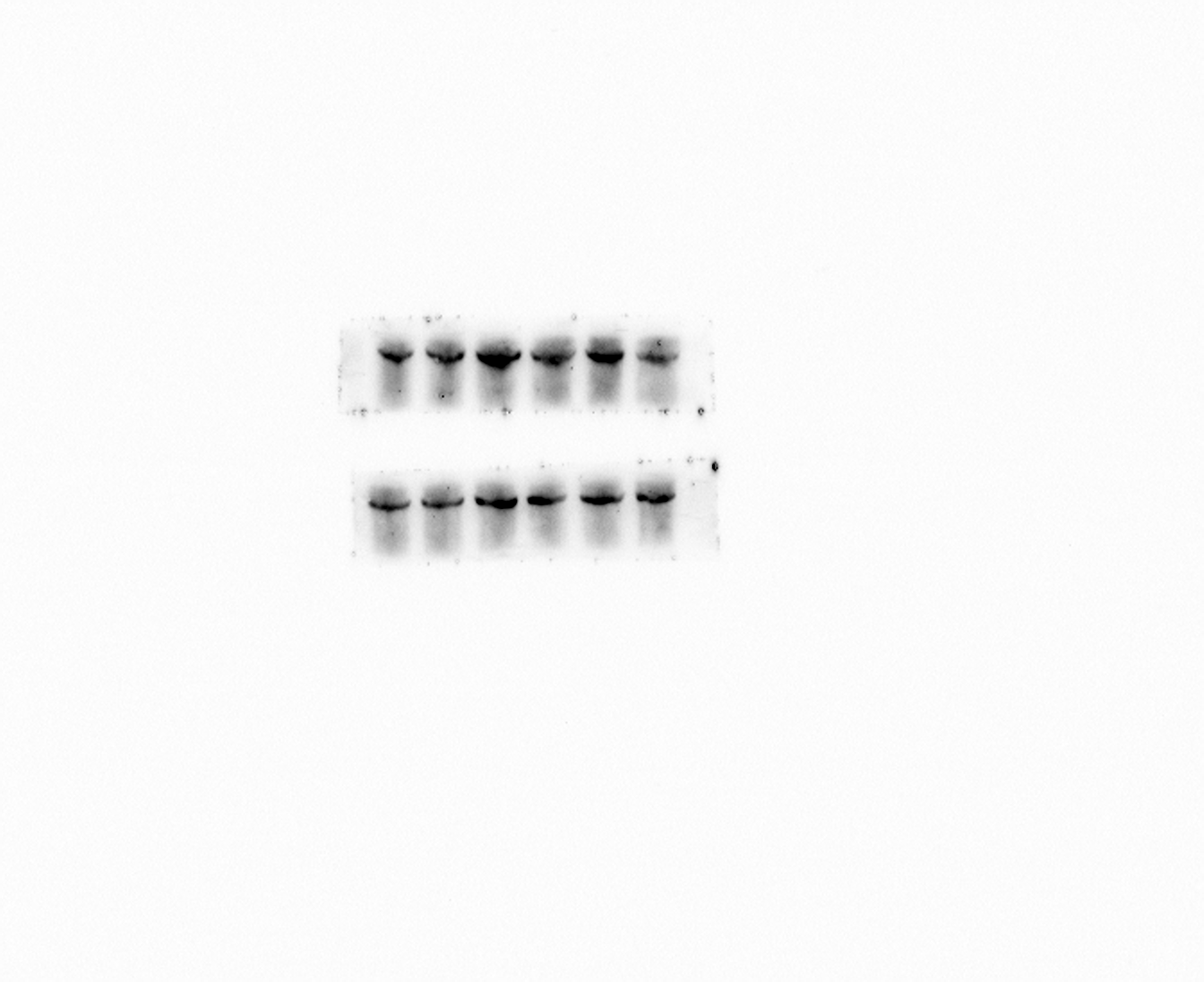

Supplement: Supplementary file 1 [file DataSheet1.ZIP › data/2022.9.6/GAPDH/10S.Tif]

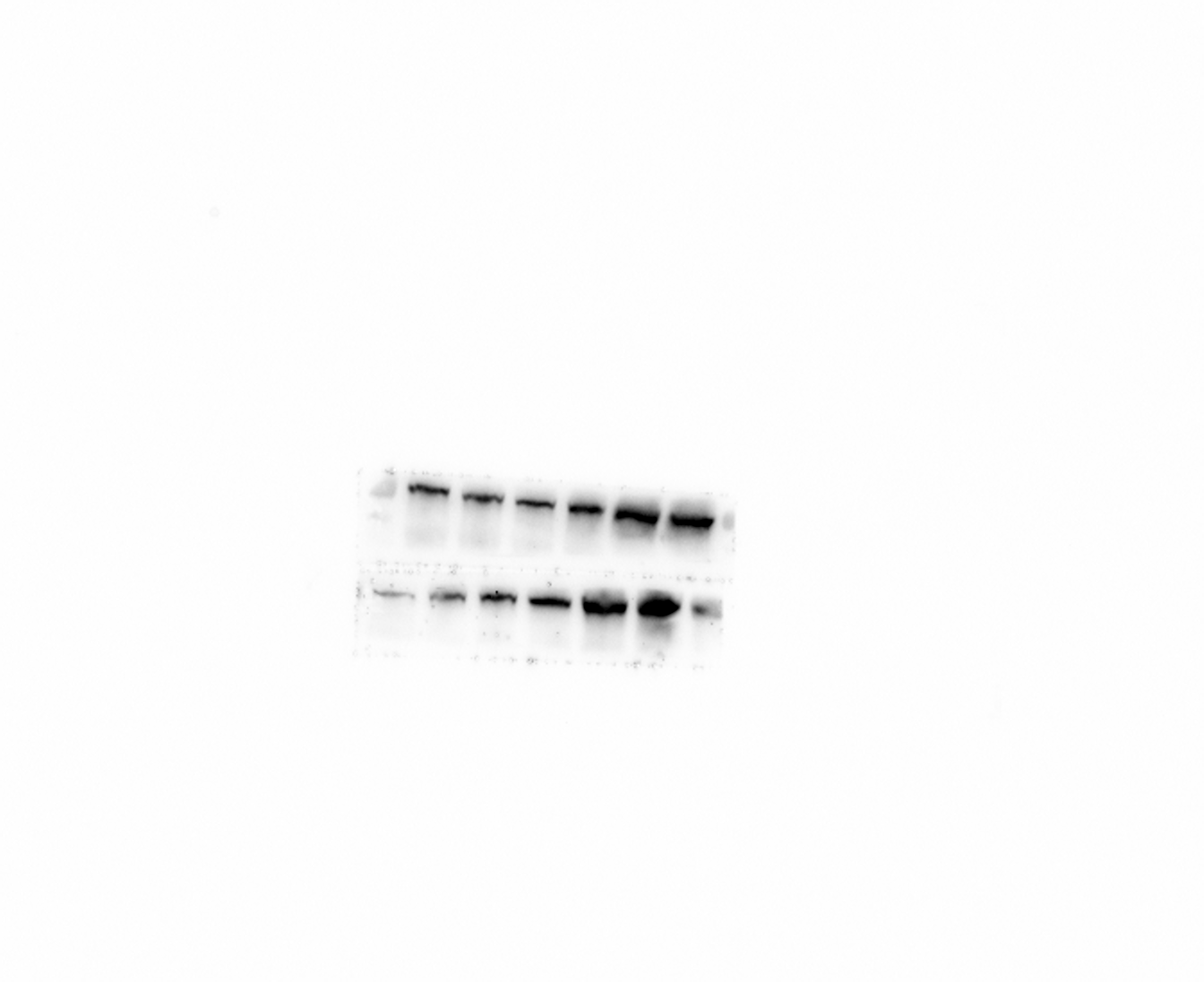

Supplement: Supplementary file 1 [file DataSheet1.ZIP › data/2022.9.6/IL-1B/10S.Tif]

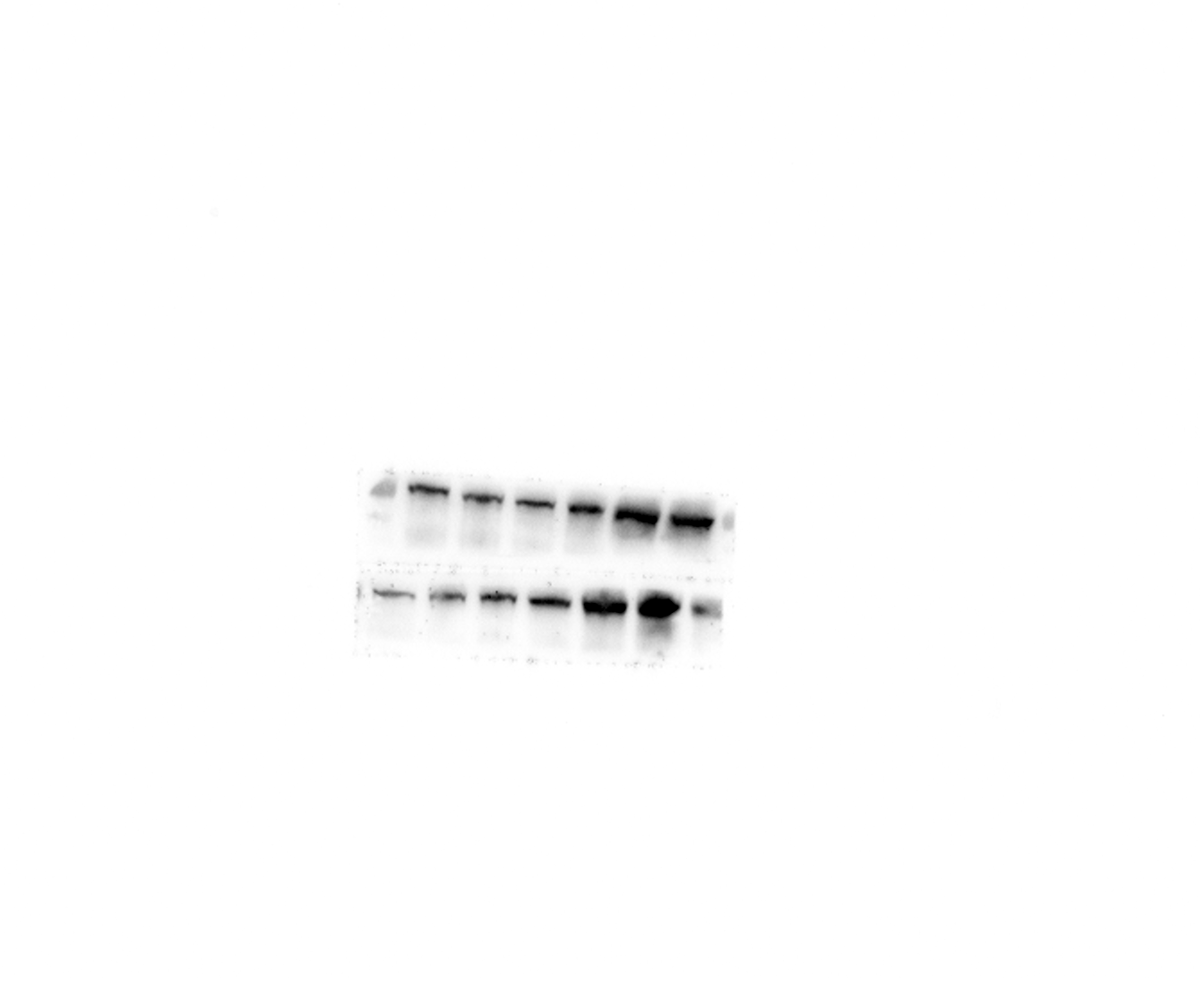

Supplement: Supplementary file 1 [file DataSheet1.ZIP › data/2022.9.6/IL-1B/6S.Tif]

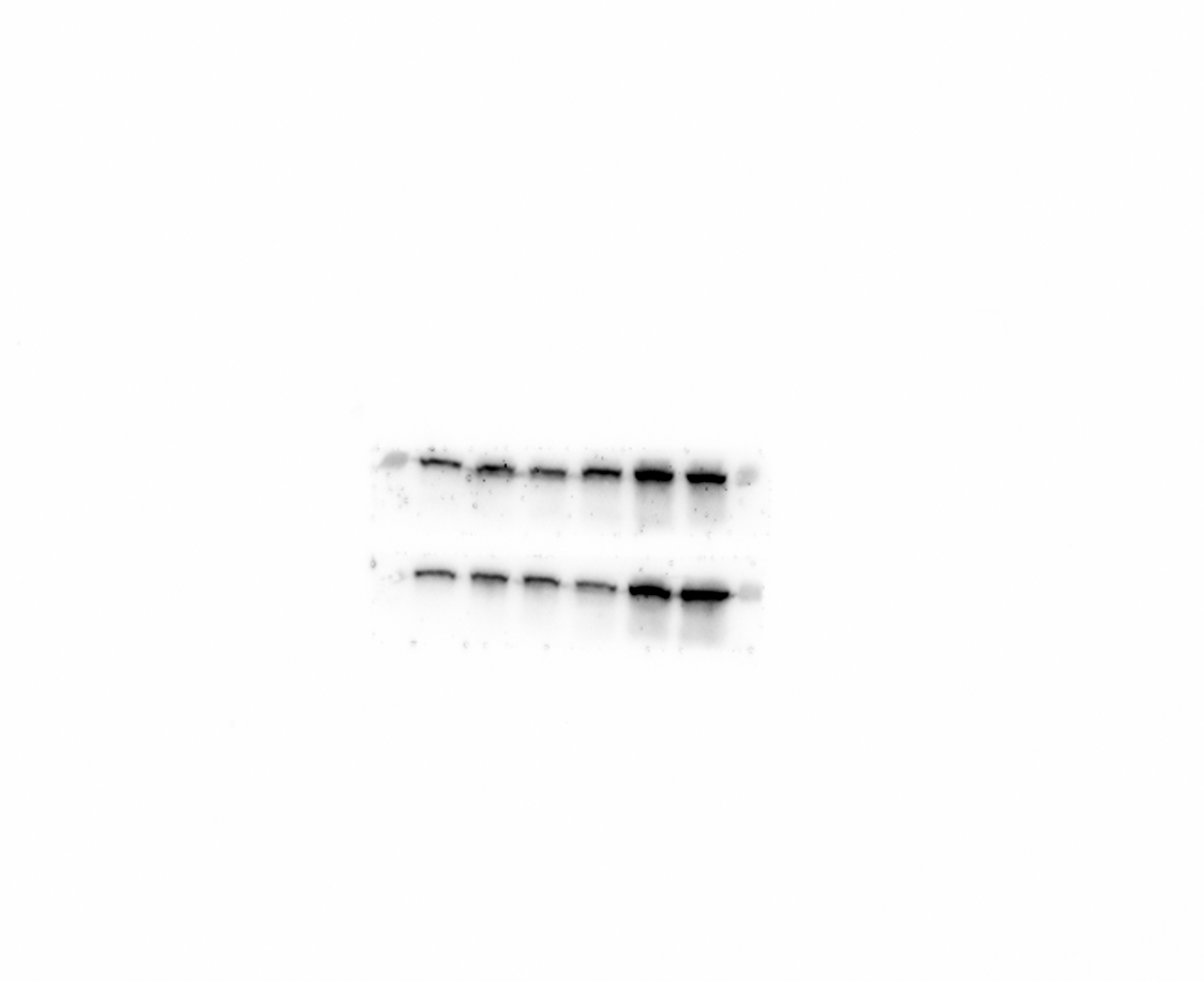

Supplement: Supplementary file 1 [file DataSheet1.ZIP › data/2022.9.6/IL6/10S.Tif]

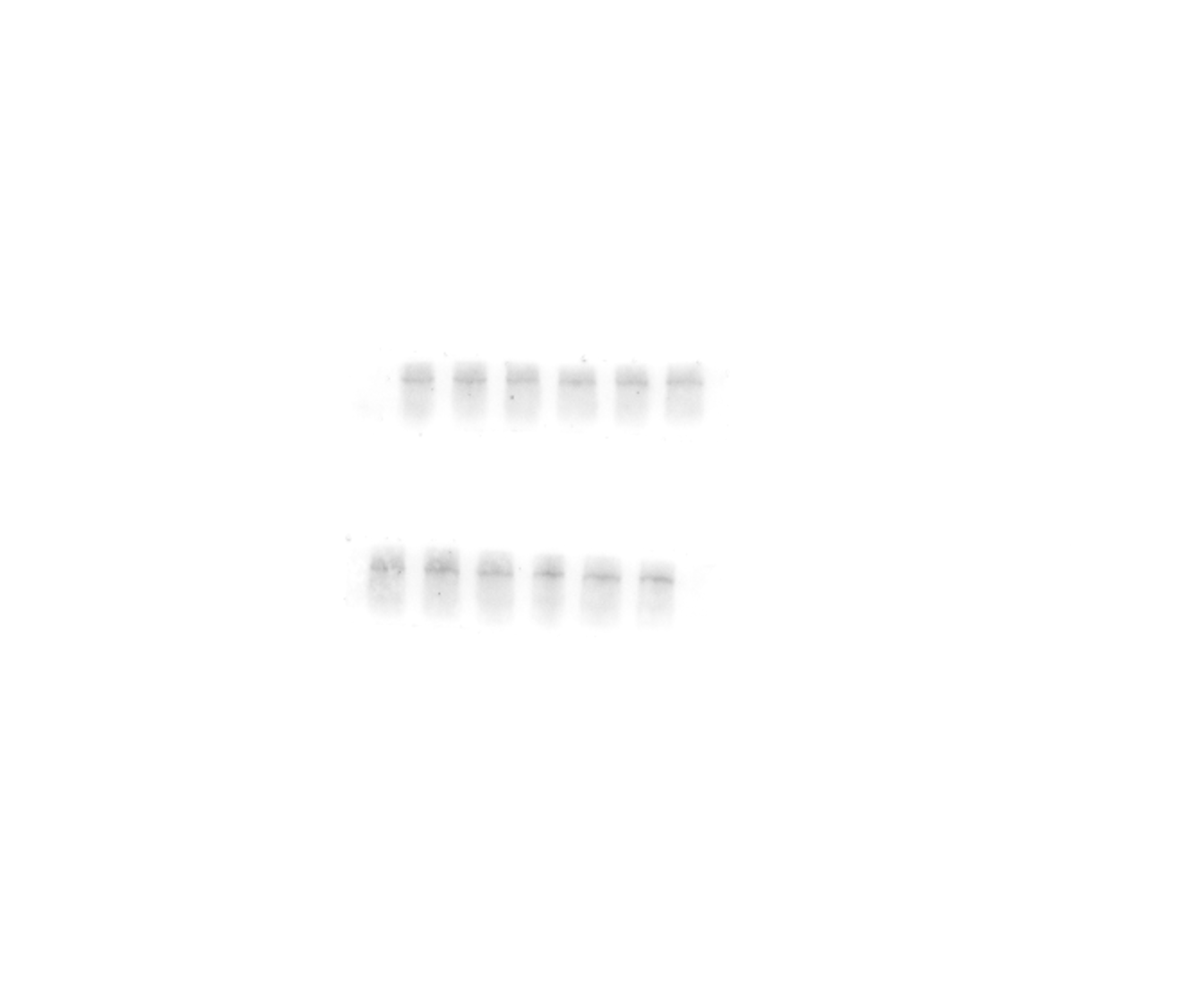

Supplement: Supplementary file 1 [file DataSheet1.ZIP › data/2022.9.6/PERK/3S.Tif]

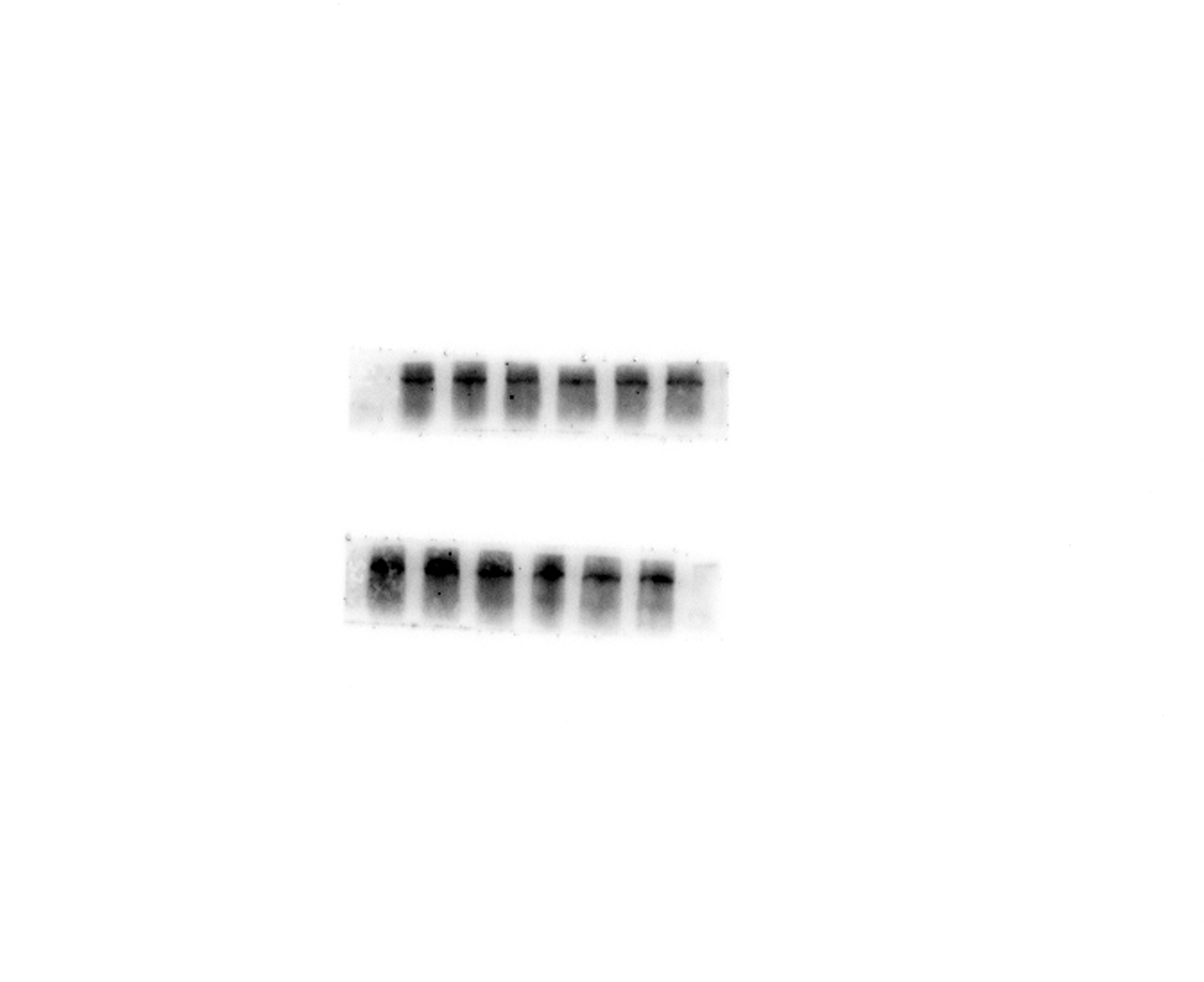

Supplement: Supplementary file 1 [file DataSheet1.ZIP › data/2022.9.6/PERK/4S.Tif]

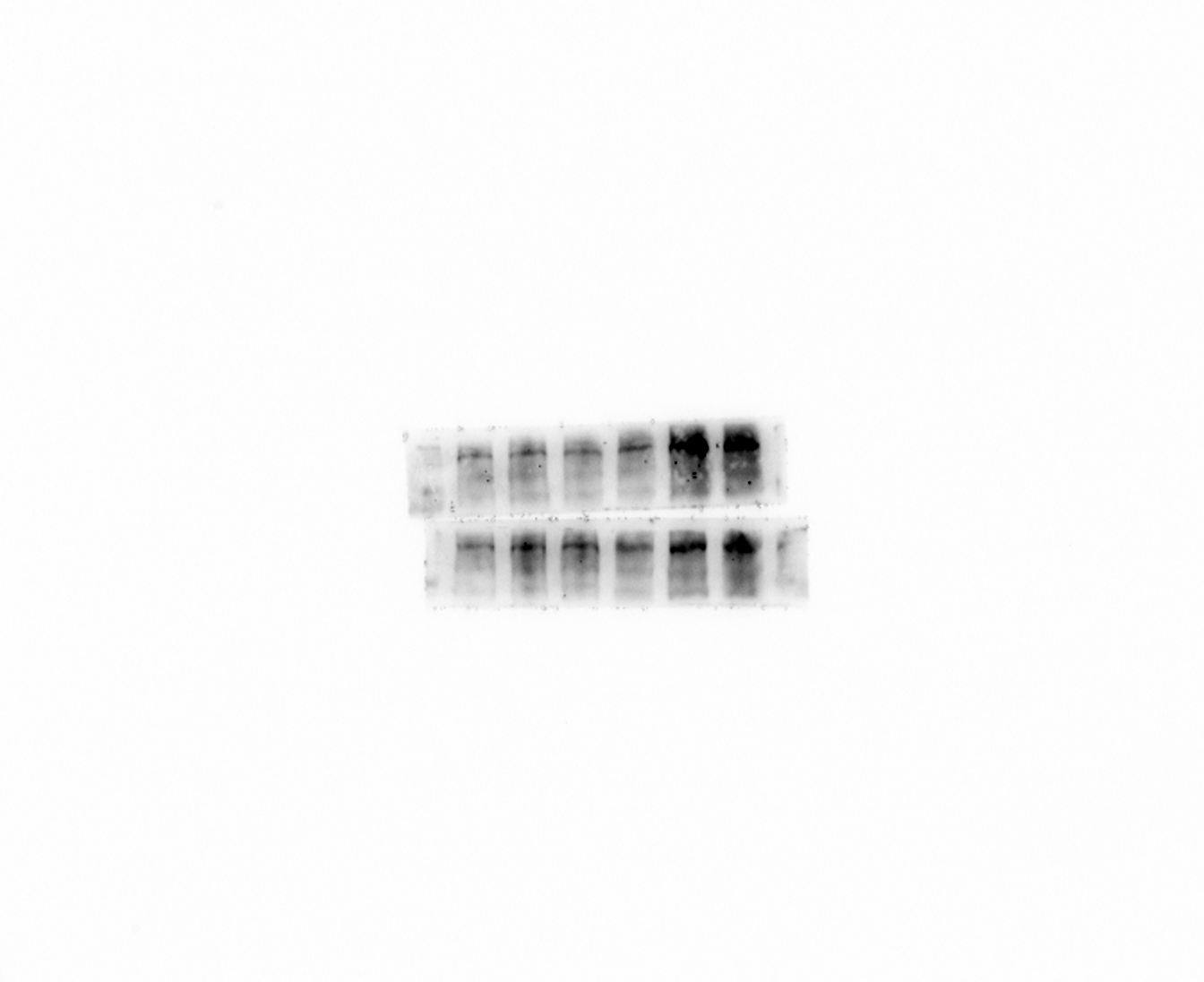

Supplement: Supplementary file 1 [file DataSheet1.ZIP › data/2022.9.6/P-PERK/10S.Tif]

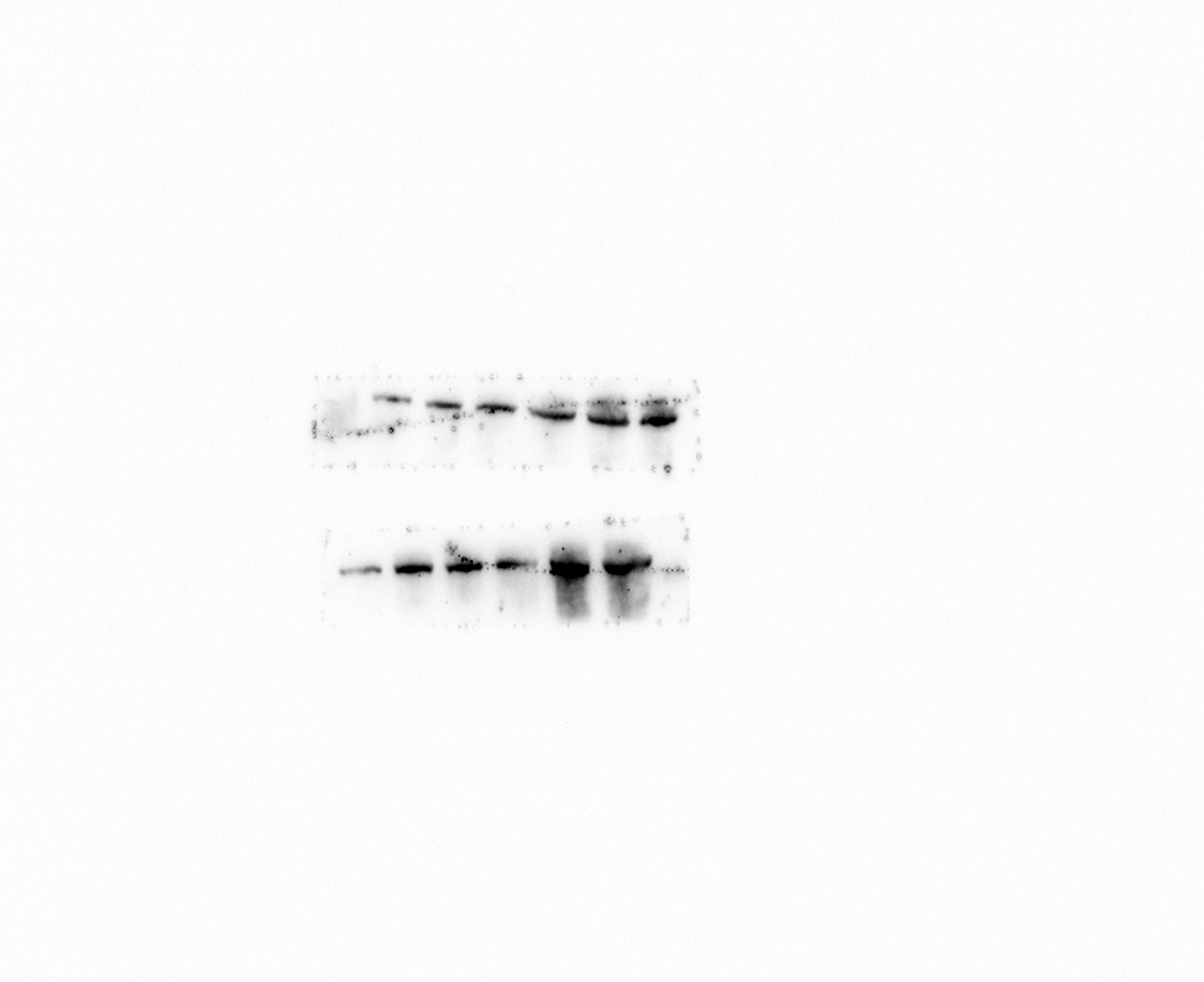

Supplement: Supplementary file 1 [file DataSheet1.ZIP › data/2022.9.6/SIRT1/10S.Tif]

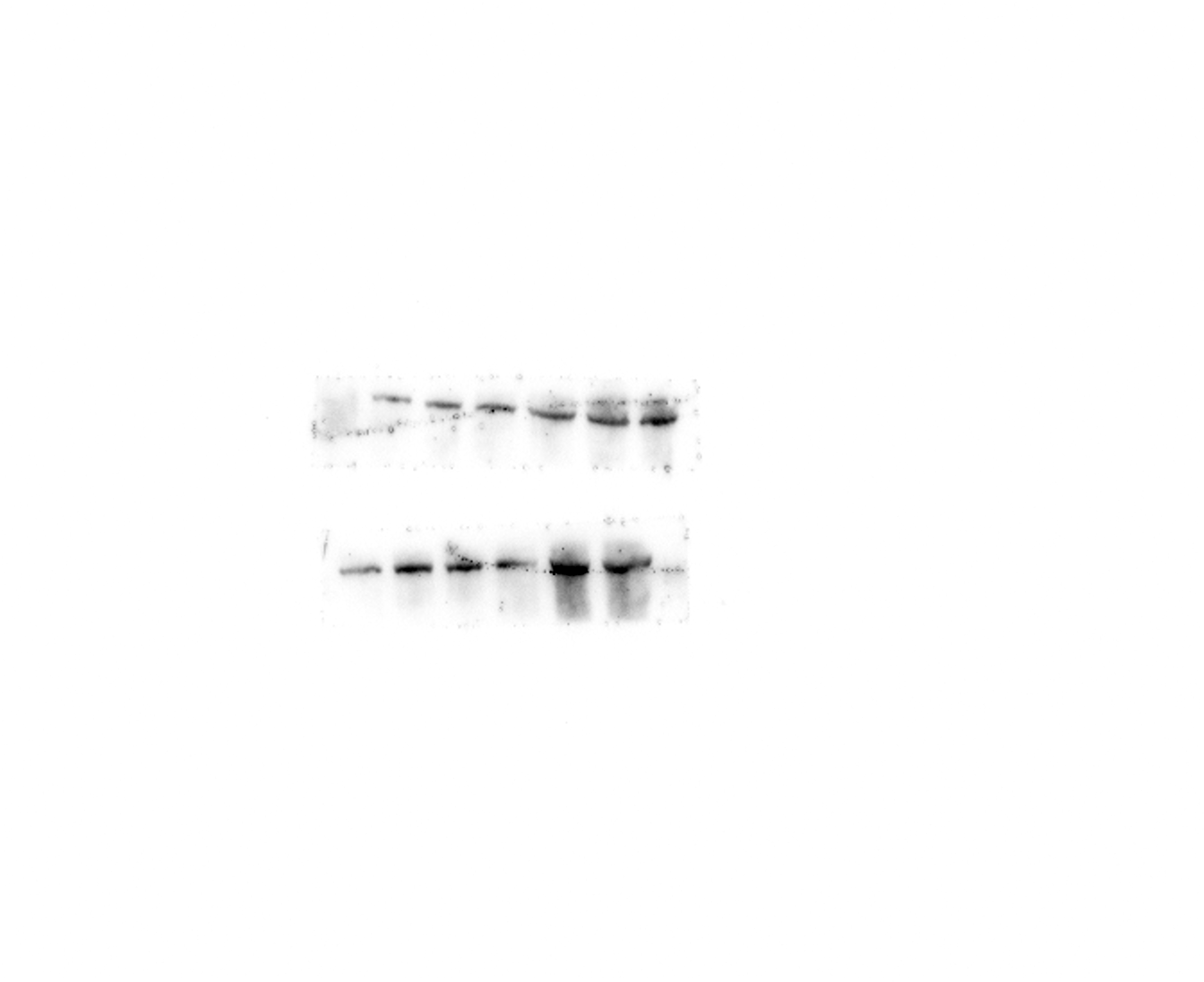

Supplement: Supplementary file 1 [file DataSheet1.ZIP › data/2022.9.6/SIRT1/8S.Tif]

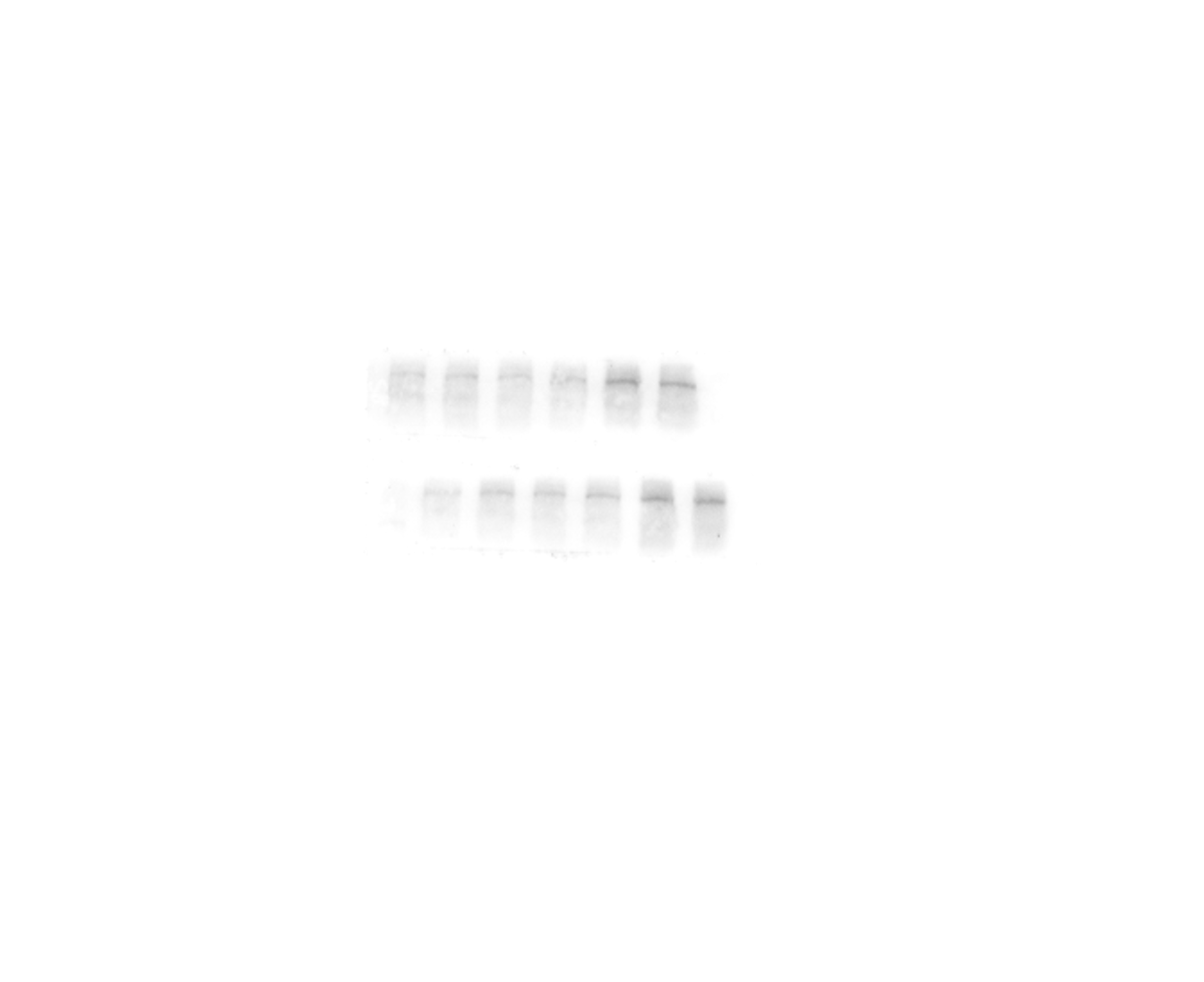

Supplement: Supplementary file 1 [file DataSheet1.ZIP › data/2022.9.6/TNF-A/10S.Tif]

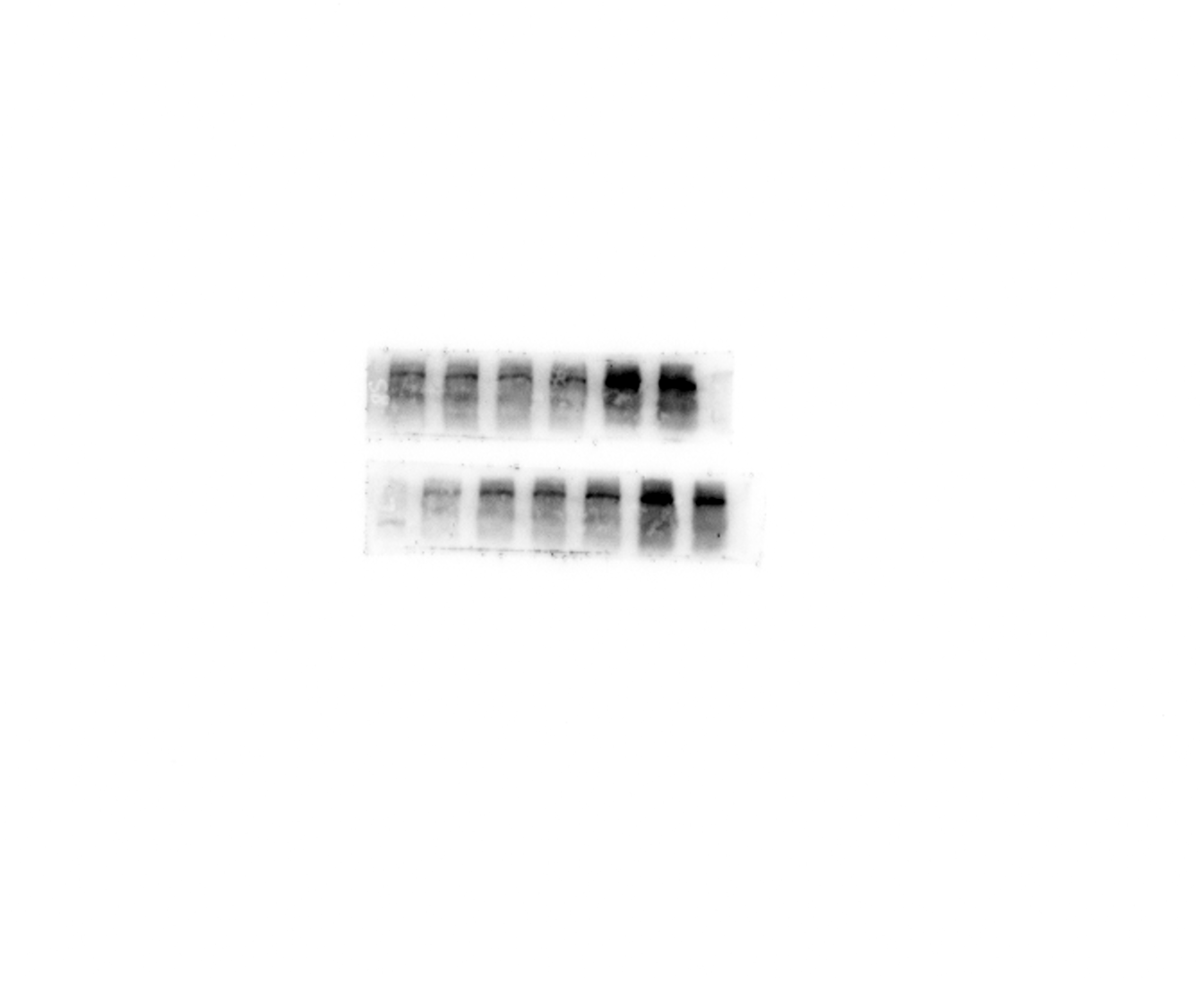

Supplement: Supplementary file 1 [file DataSheet1.ZIP › data/2022.9.6/TNF-A/6S.Tif]

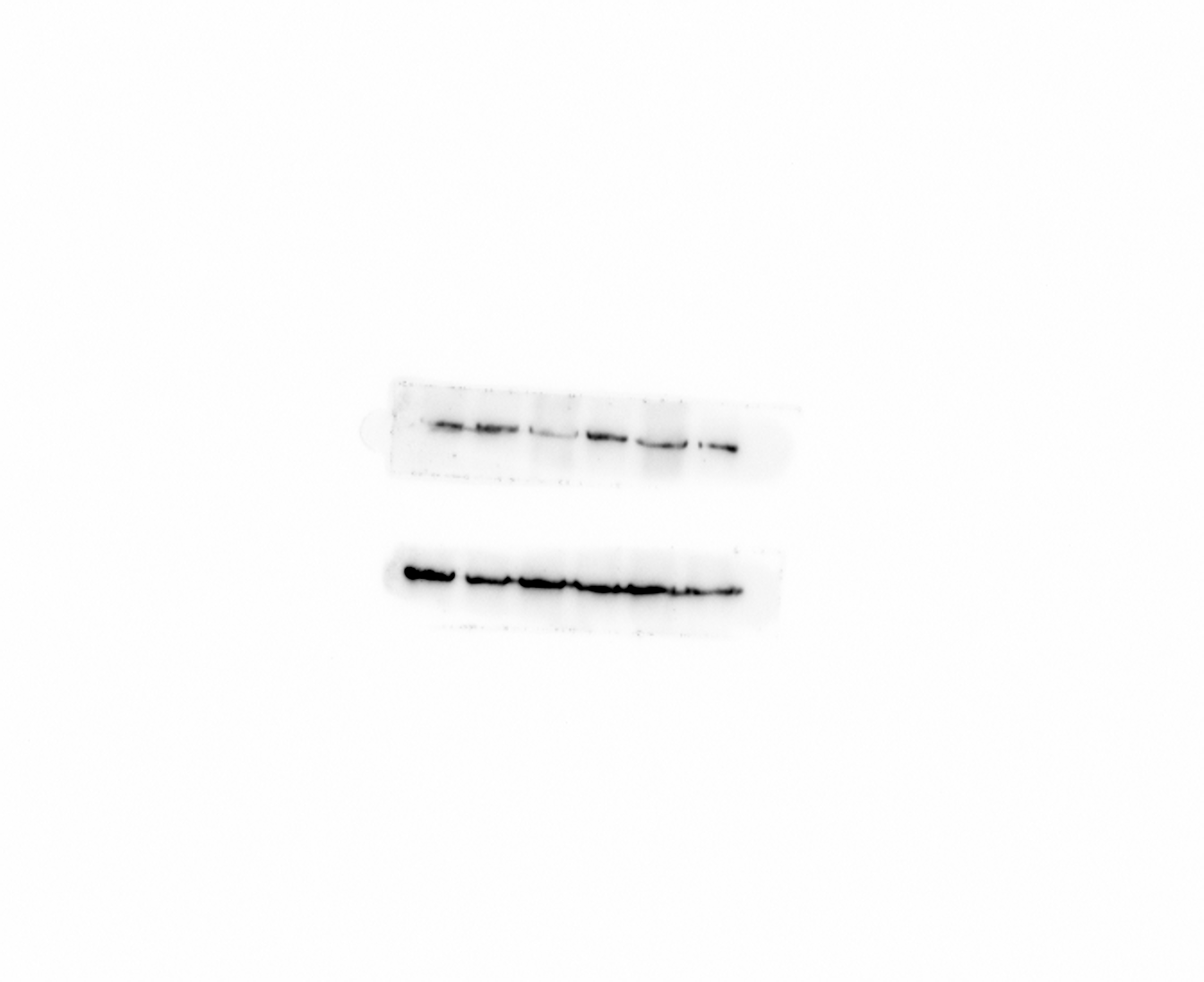

Supplement: Supplementary file 2 [file DataSheet2.ZIP › data1/2022.8.12/ATF4/30S.Tif]

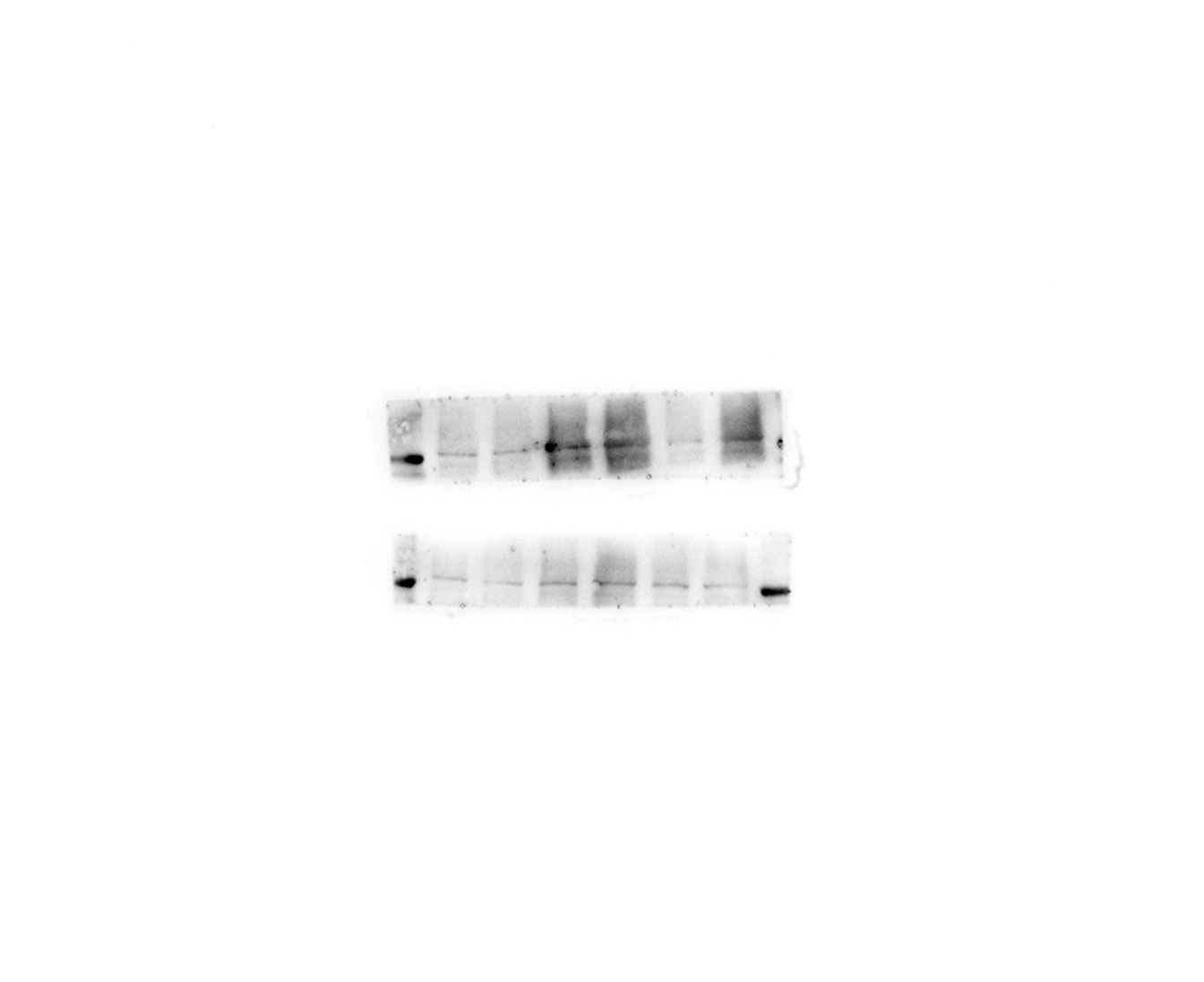

Supplement: Supplementary file 2 [file DataSheet2.ZIP › data1/2022.8.12/EIF2A/20S.Tif]

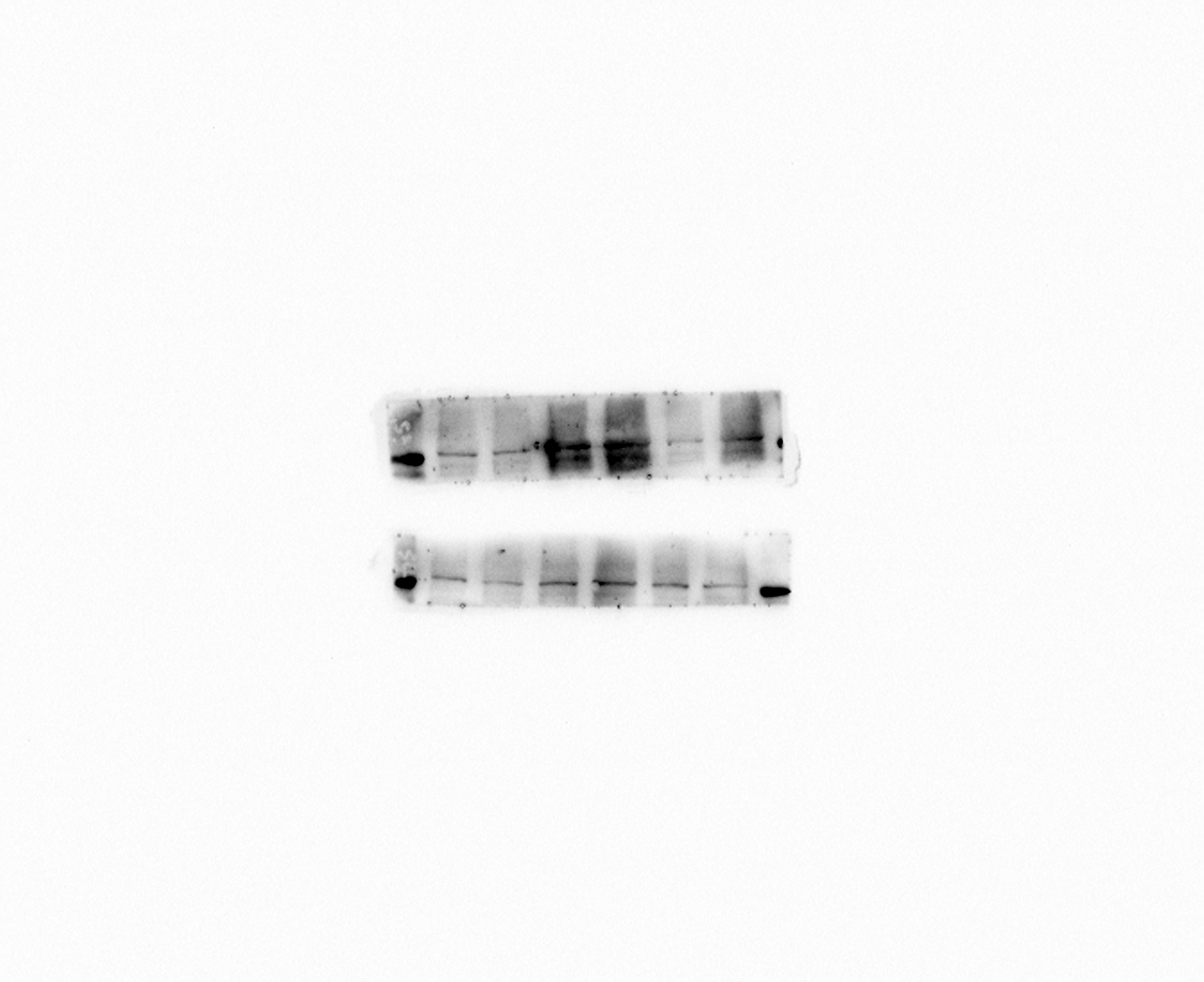

Supplement: Supplementary file 2 [file DataSheet2.ZIP › data1/2022.8.12/EIF2A/30S.Tif]

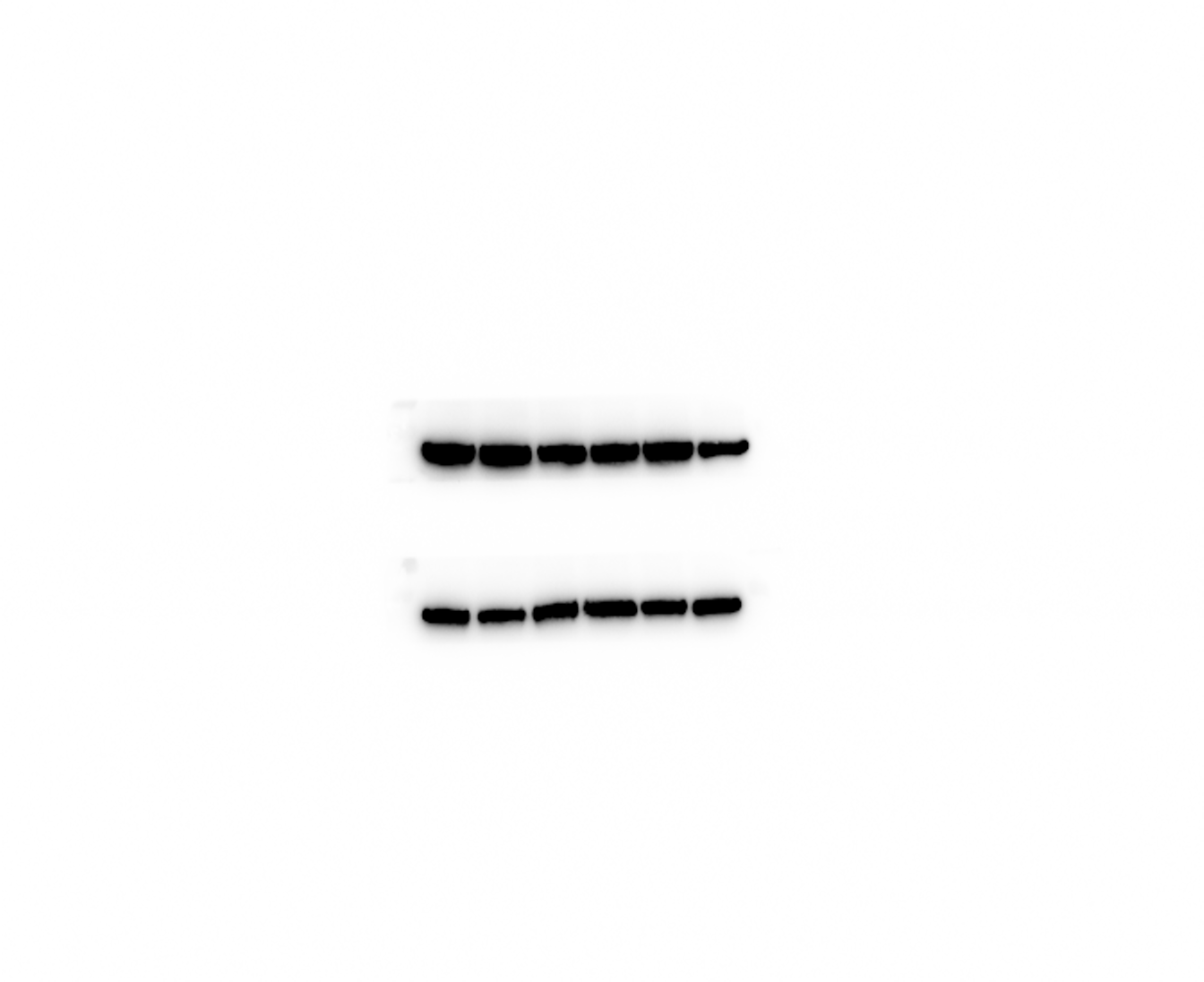

Supplement: Supplementary file 2 [file DataSheet2.ZIP › data1/2022.8.12/GAPDH/1S.Tif]

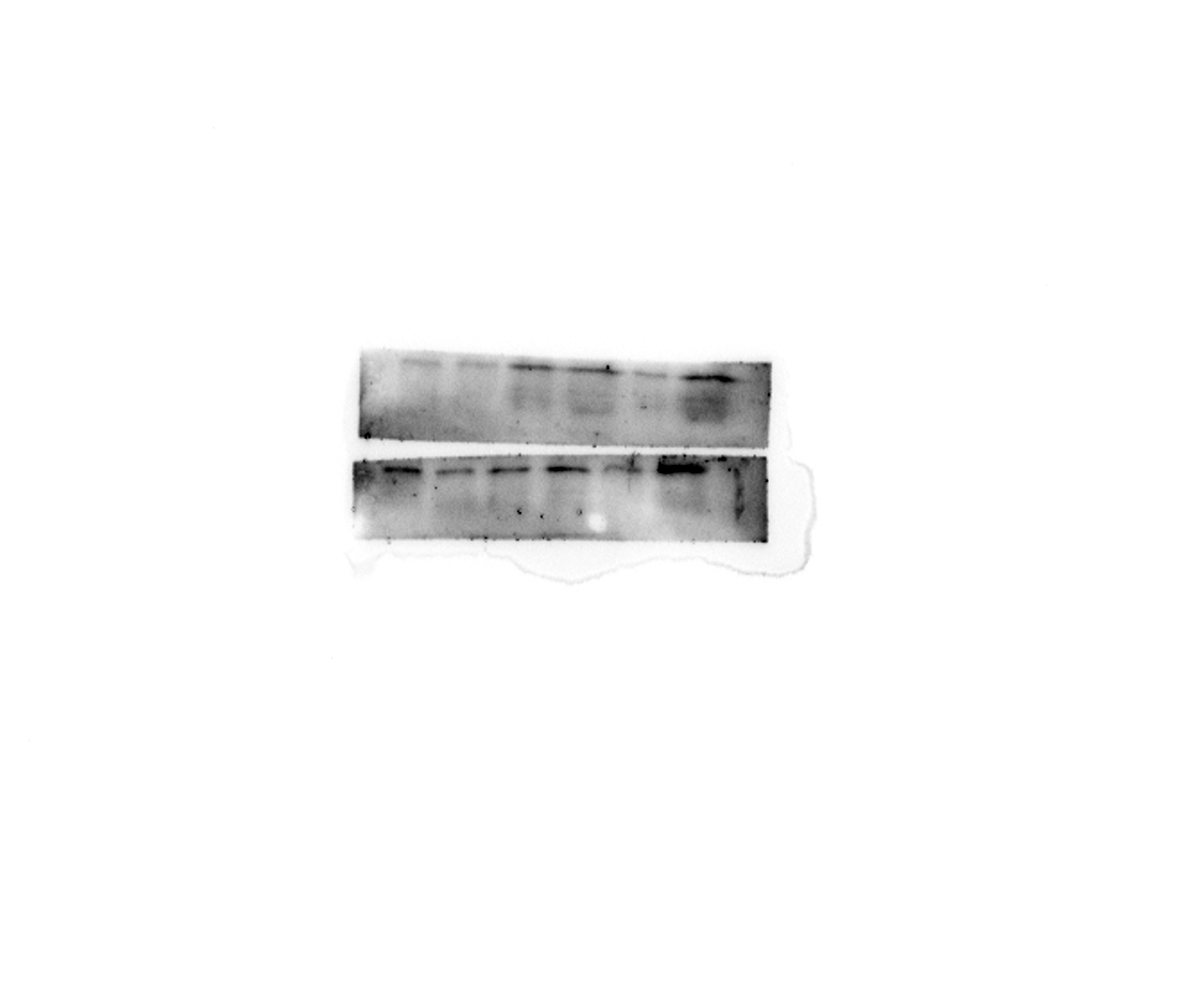

Supplement: Supplementary file 2 [file DataSheet2.ZIP › data1/2022.8.12/P-EIF2A/10S.Tif]

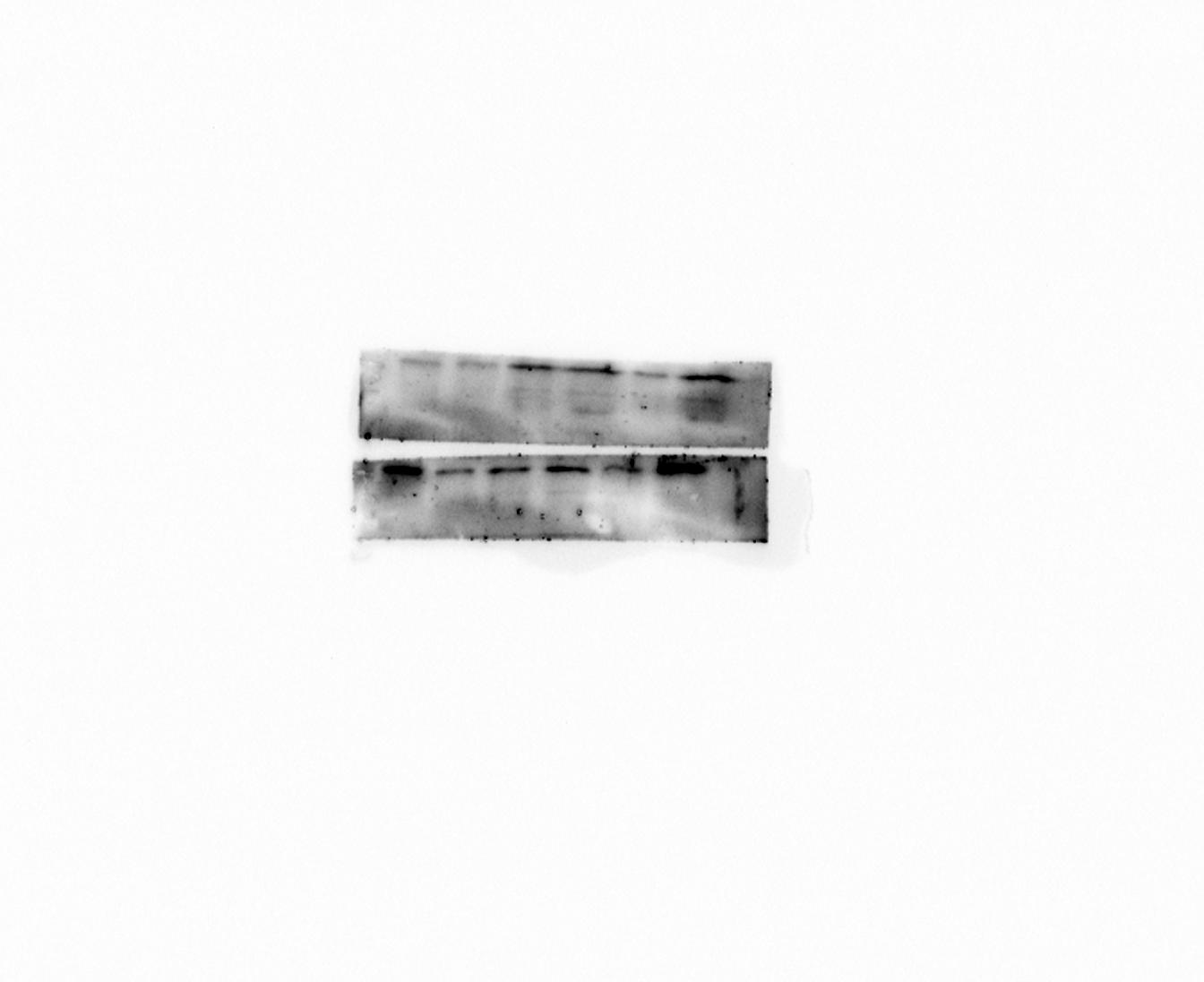

Supplement: Supplementary file 2 [file DataSheet2.ZIP › data1/2022.8.12/P-EIF2A/30S.Tif]

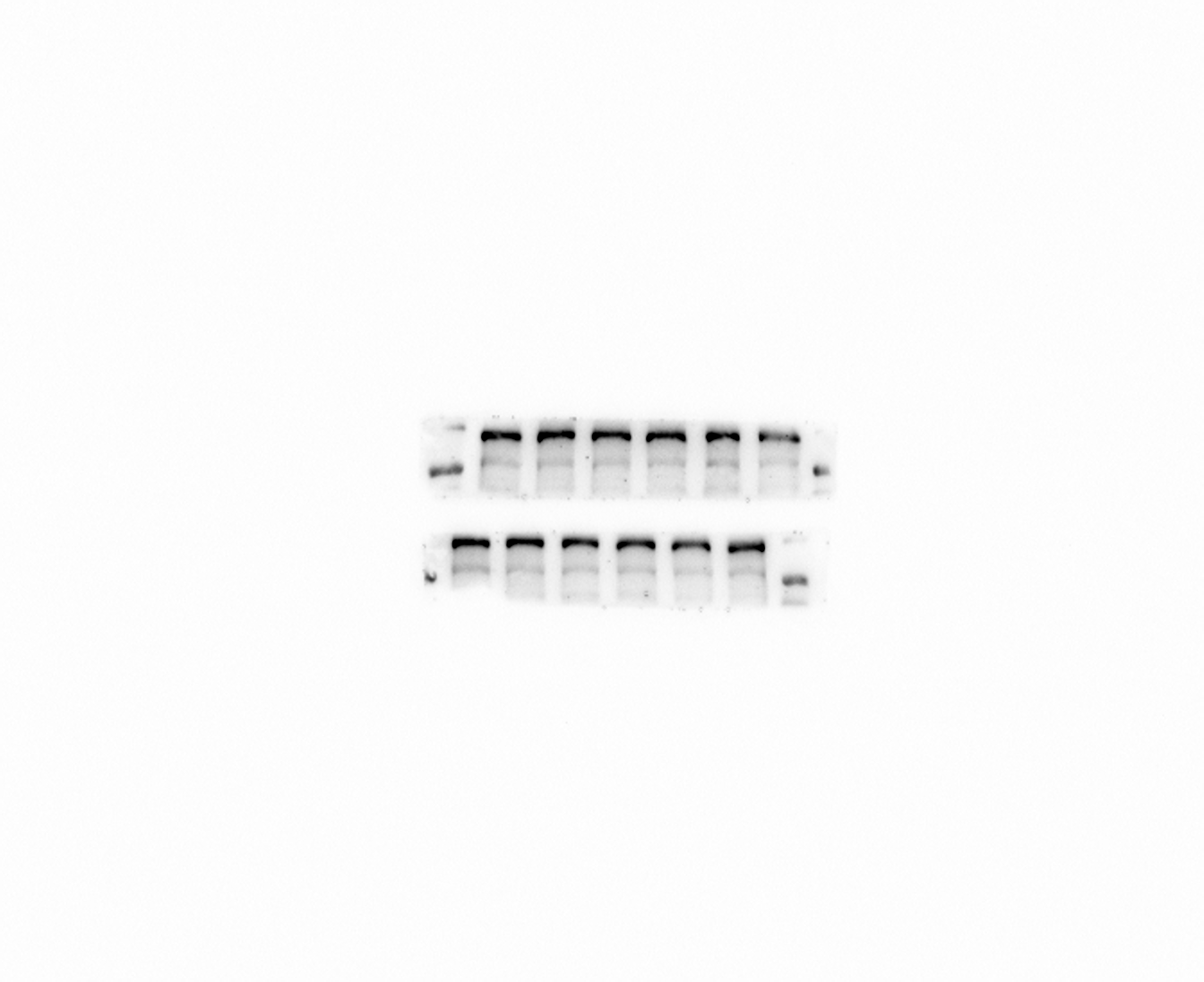

Supplement: Supplementary file 2 [file DataSheet2.ZIP › data1/2022.8.12/PERK/10S.Tif]

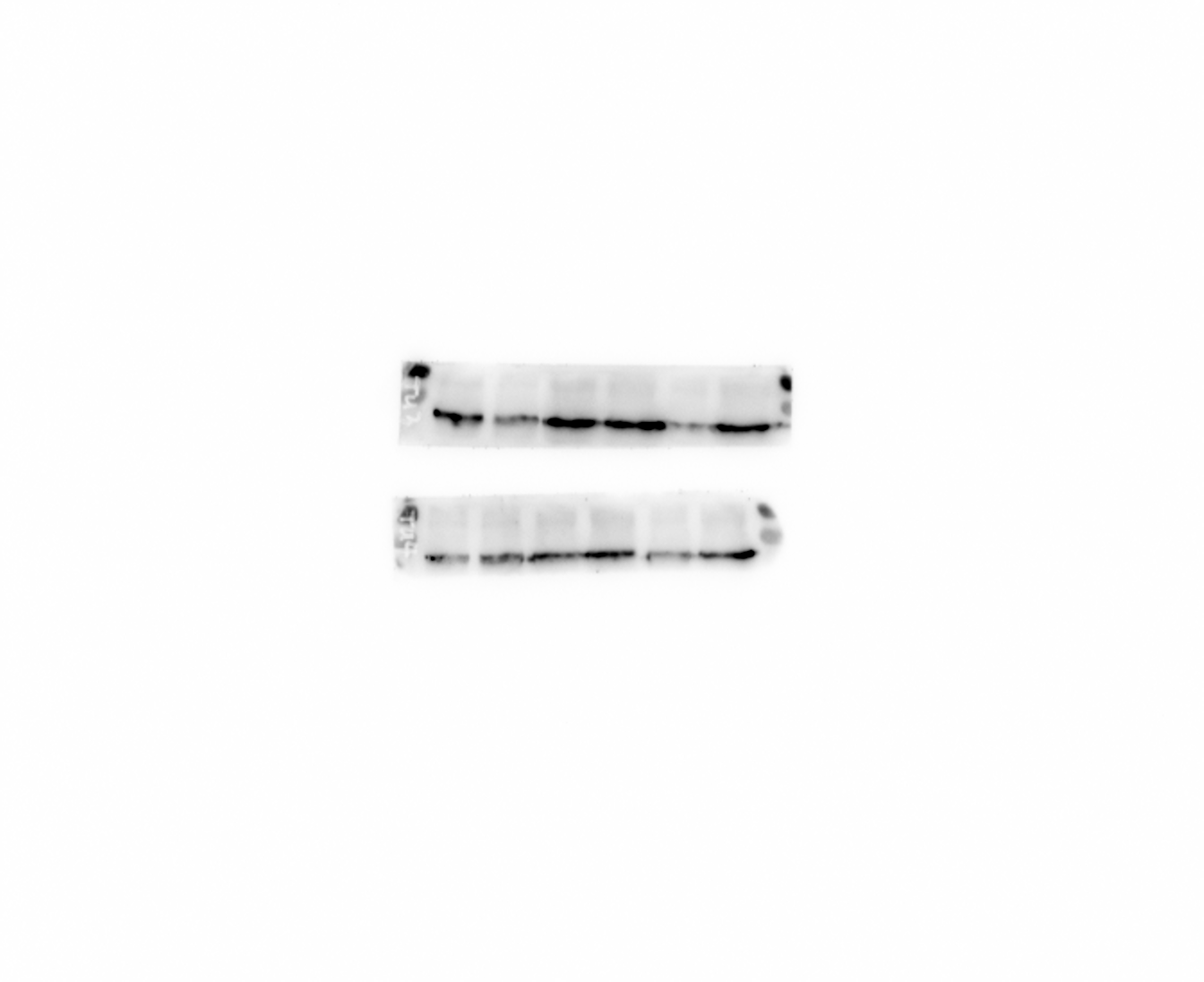

Supplement: Supplementary file 2 [file DataSheet2.ZIP › data1/2022.8.12/P-PERK/10S.Tif]

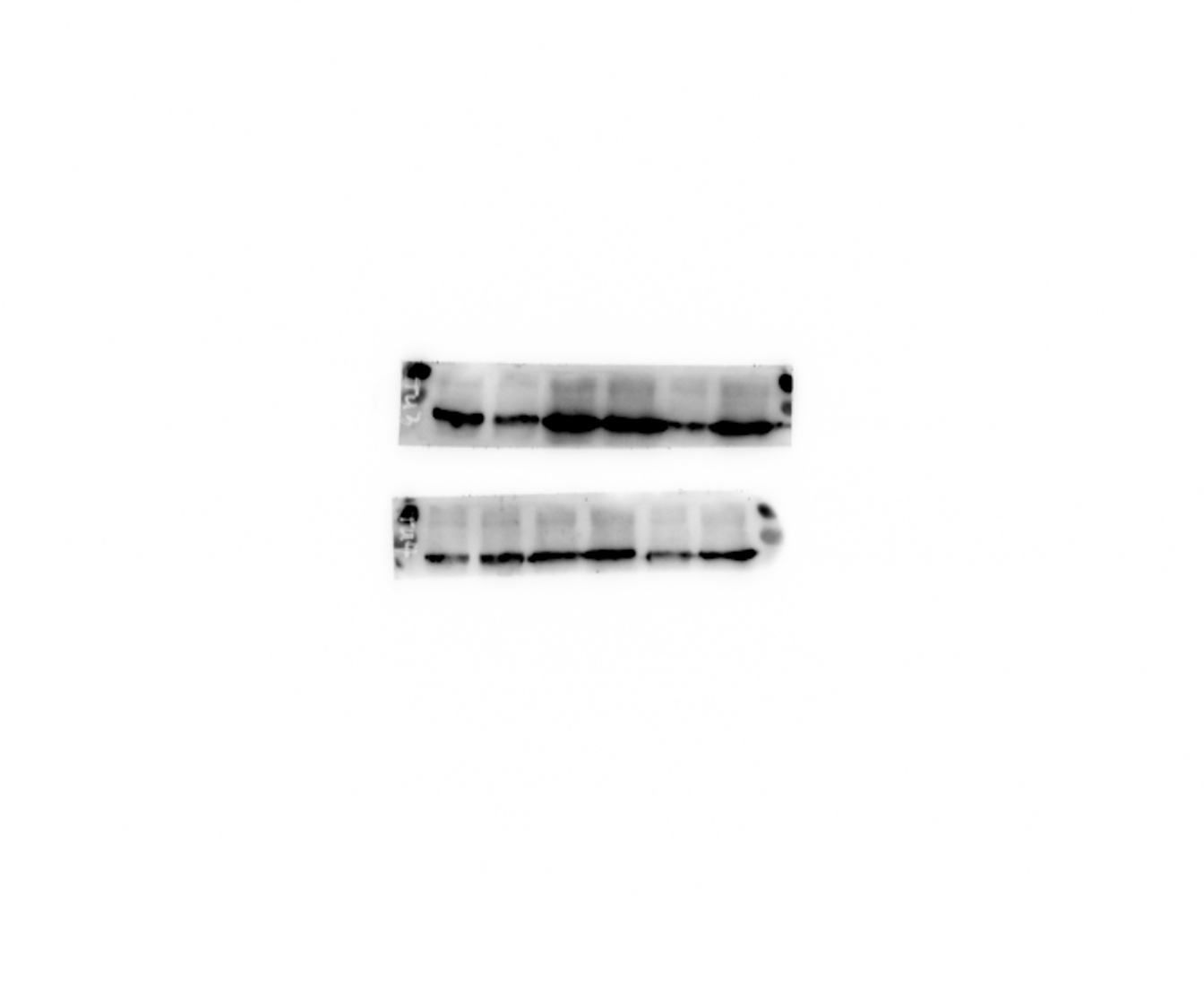

Supplement: Supplementary file 2 [file DataSheet2.ZIP › data1/2022.8.12/P-PERK/30S.Tif]

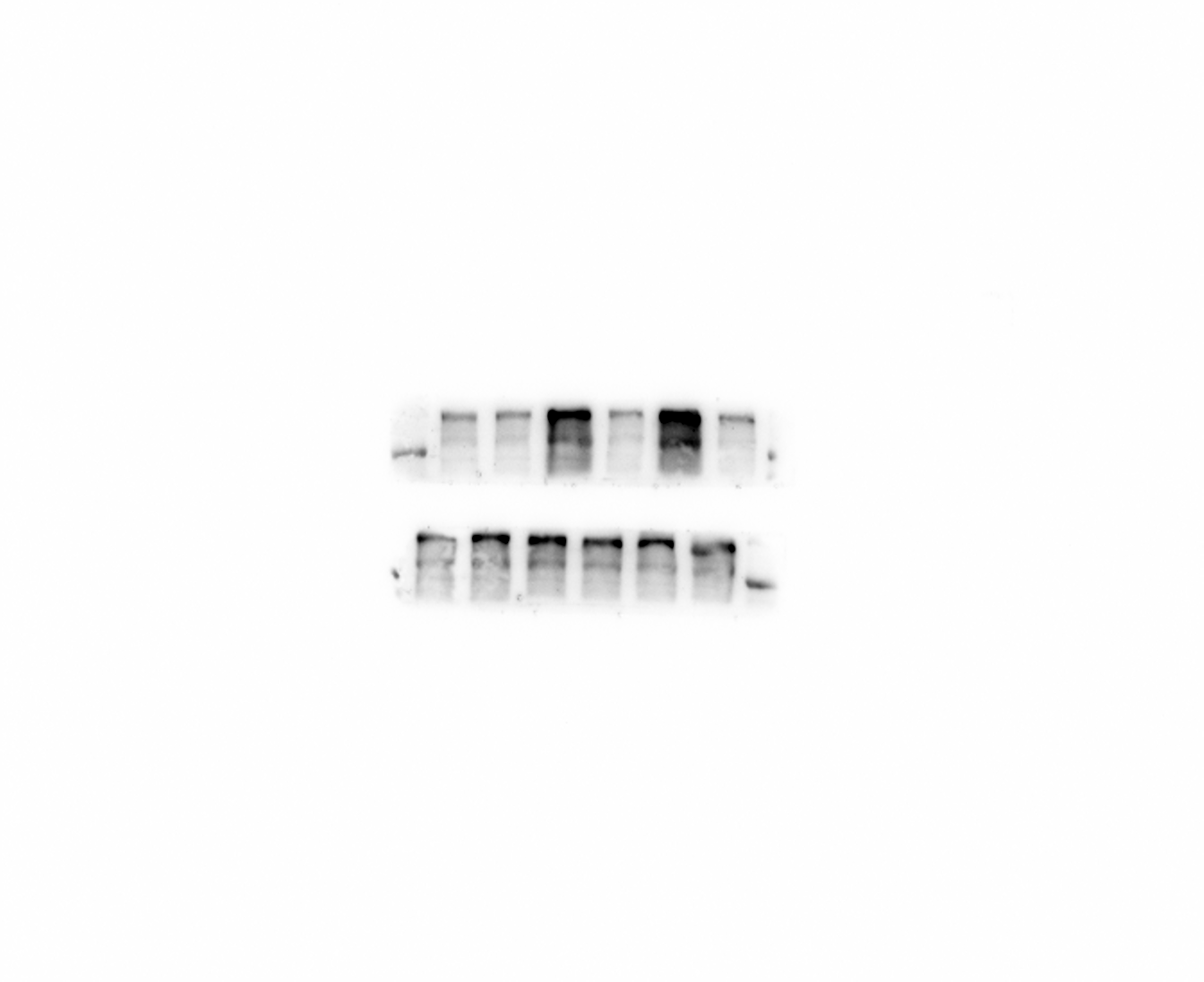

Supplement: Supplementary file 2 [file DataSheet2.ZIP › data1/2022.8.12/SIRT1 2/10S.Tif]

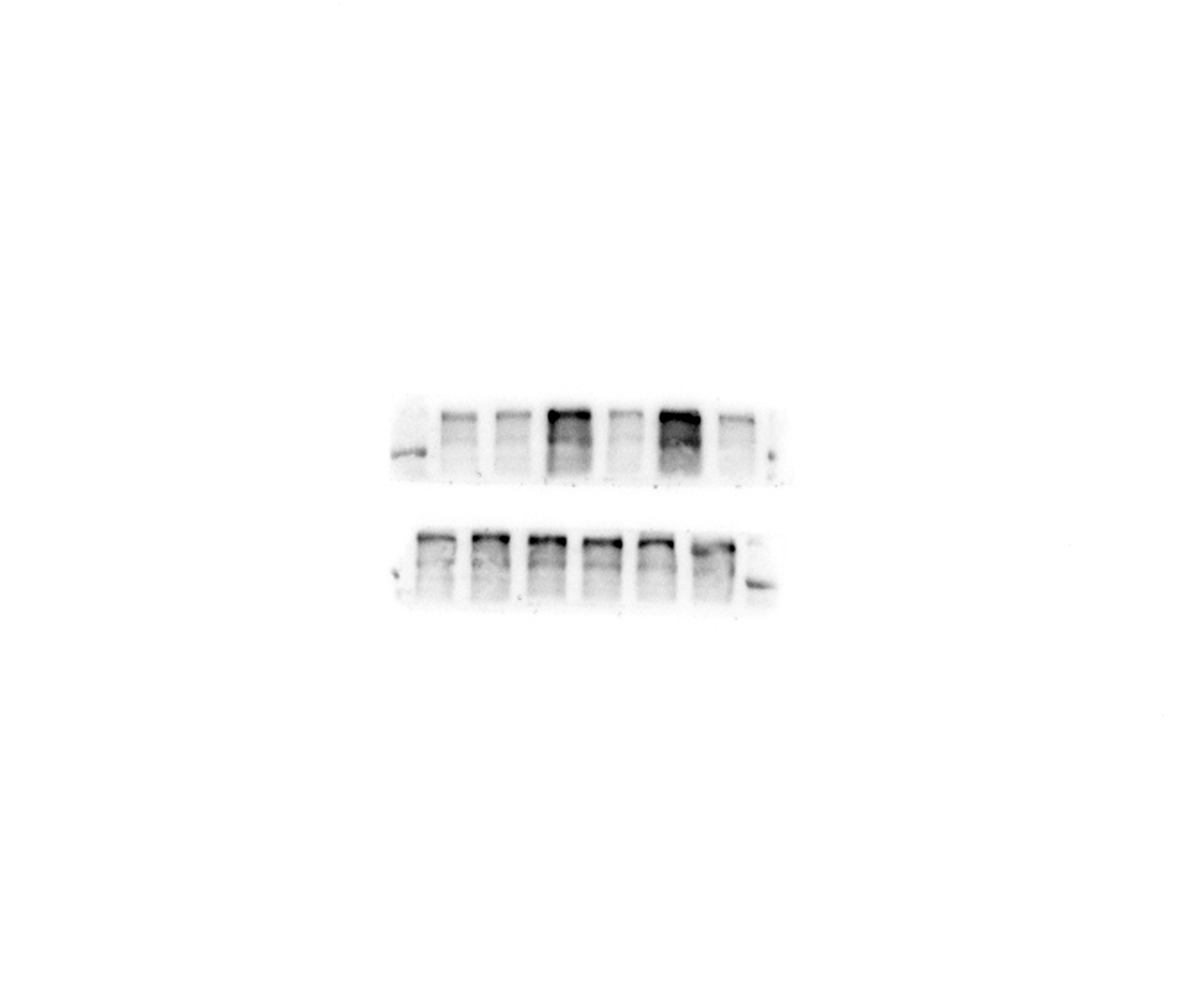

Supplement: Supplementary file 2 [file DataSheet2.ZIP › data1/2022.8.12/SIRT1 2/3S.Tif]

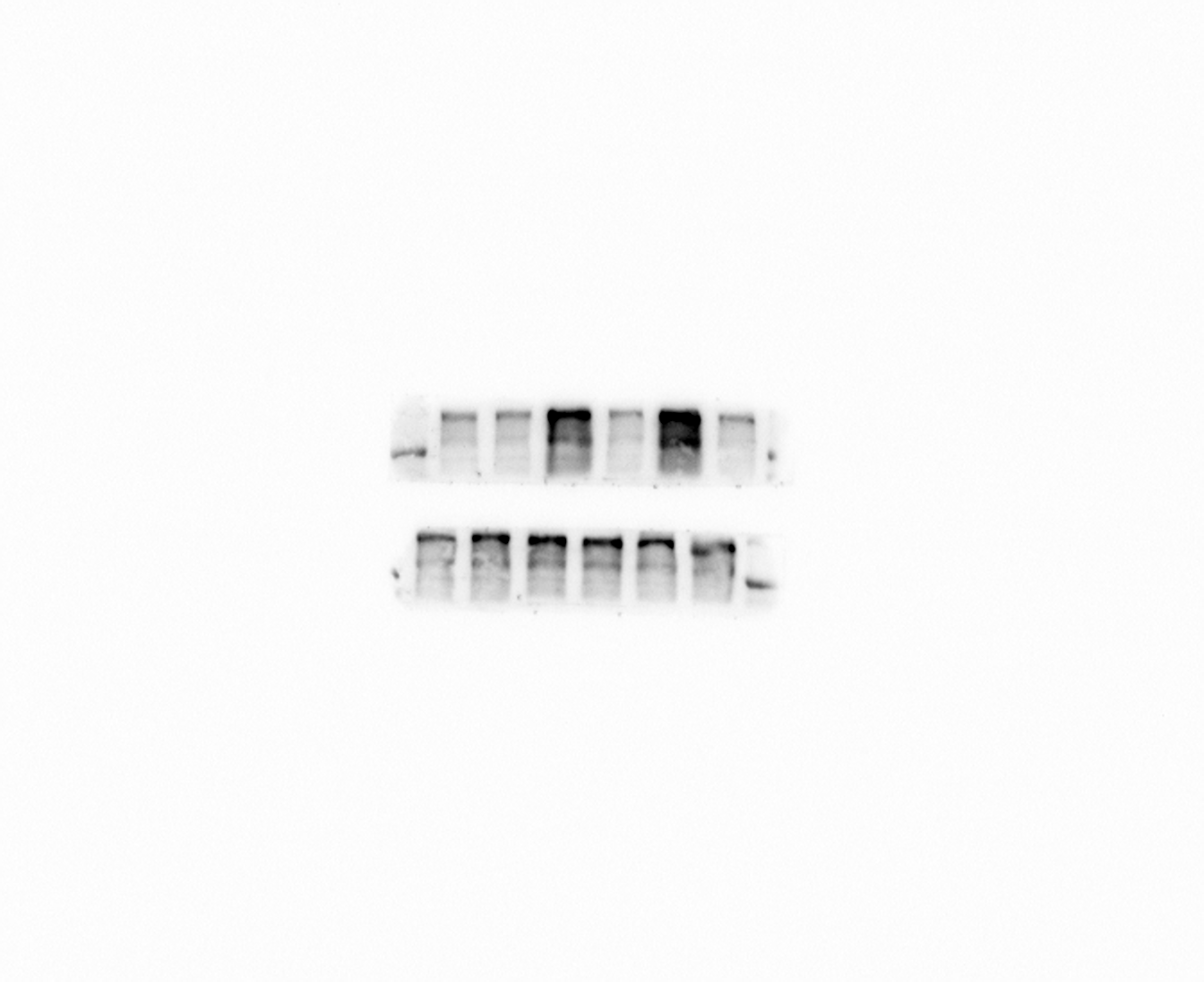

Supplement: Supplementary file 2 [file DataSheet2.ZIP › data1/2022.8.12/SIRT1 2/5S.Tif]

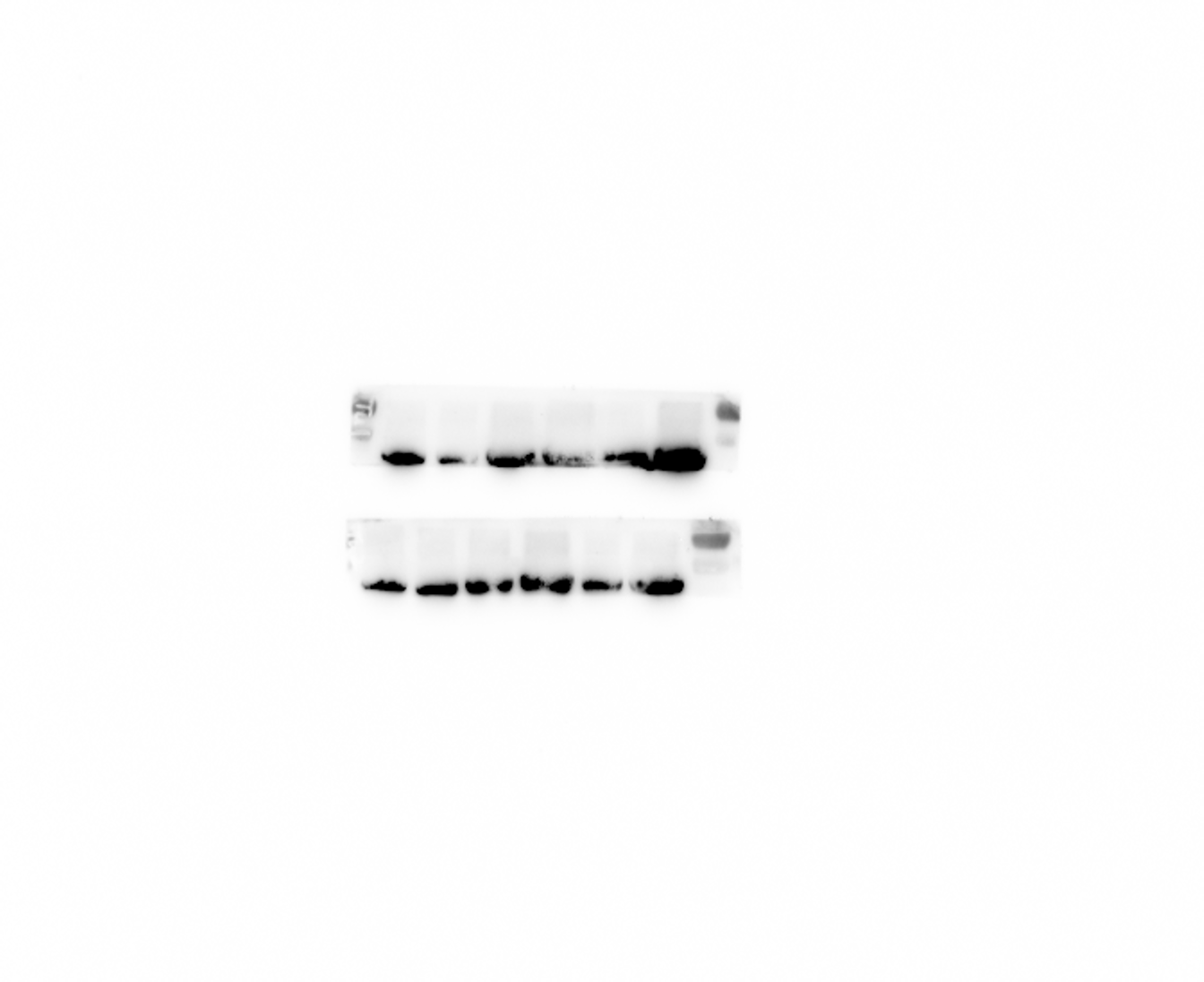

Supplement: Supplementary file 2 [file DataSheet2.ZIP › data1/2022.8.12/SIRT1/10S.Tif]

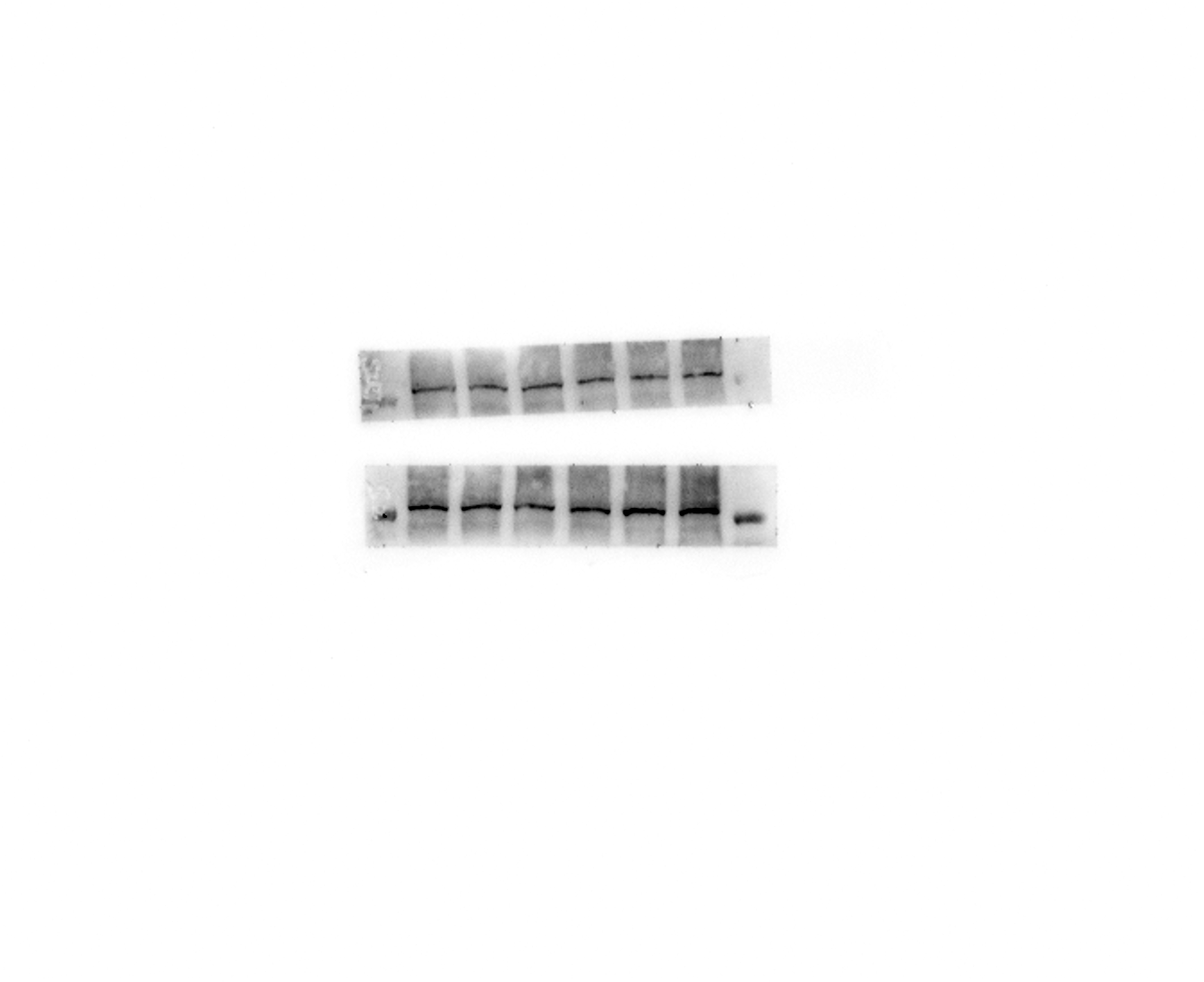

Supplement: Supplementary file 2 [file DataSheet2.ZIP › data1/2022.8.16/EIF2A/20S.Tif]

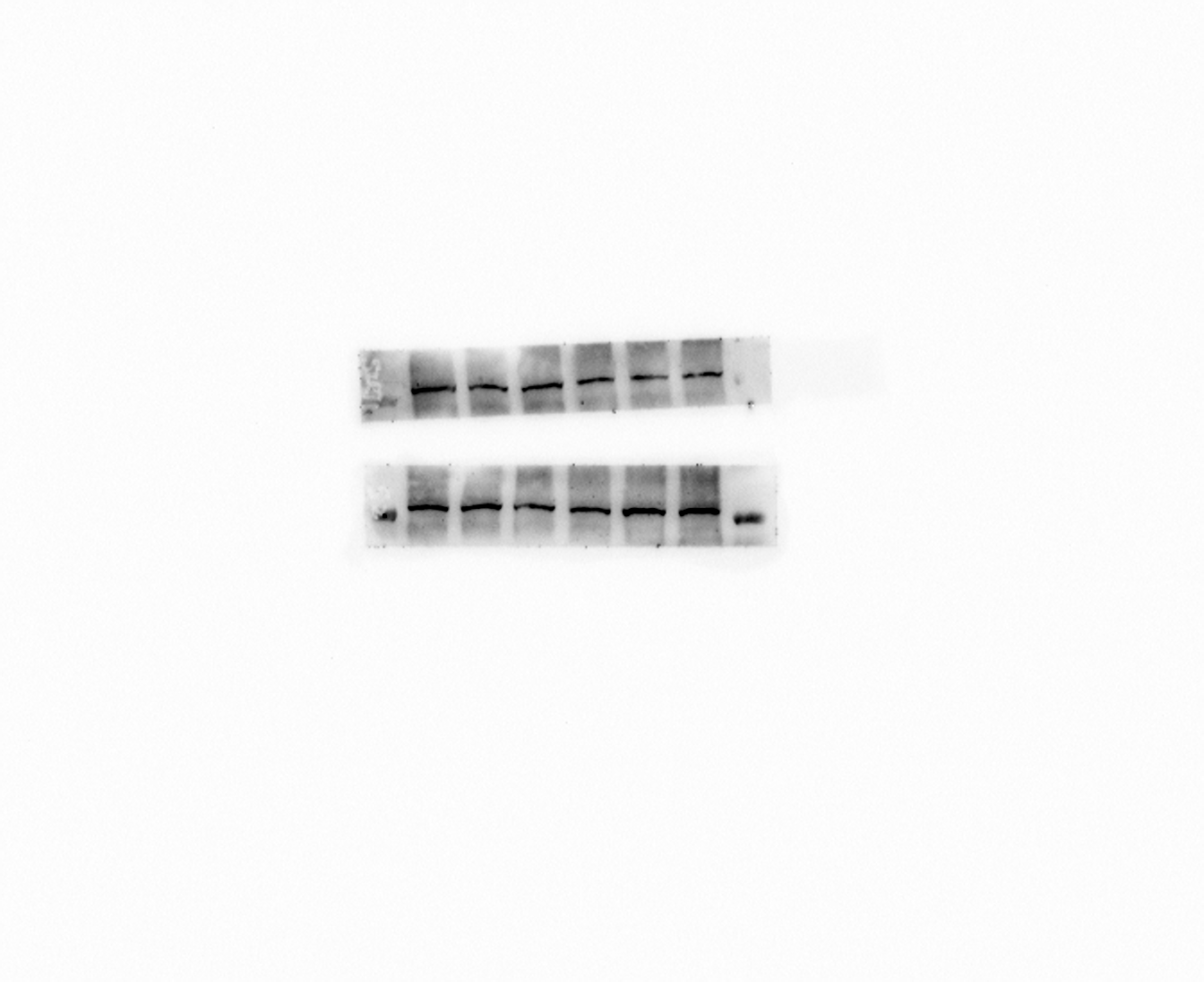

Supplement: Supplementary file 2 [file DataSheet2.ZIP › data1/2022.8.16/EIF2A/30S.Tif]

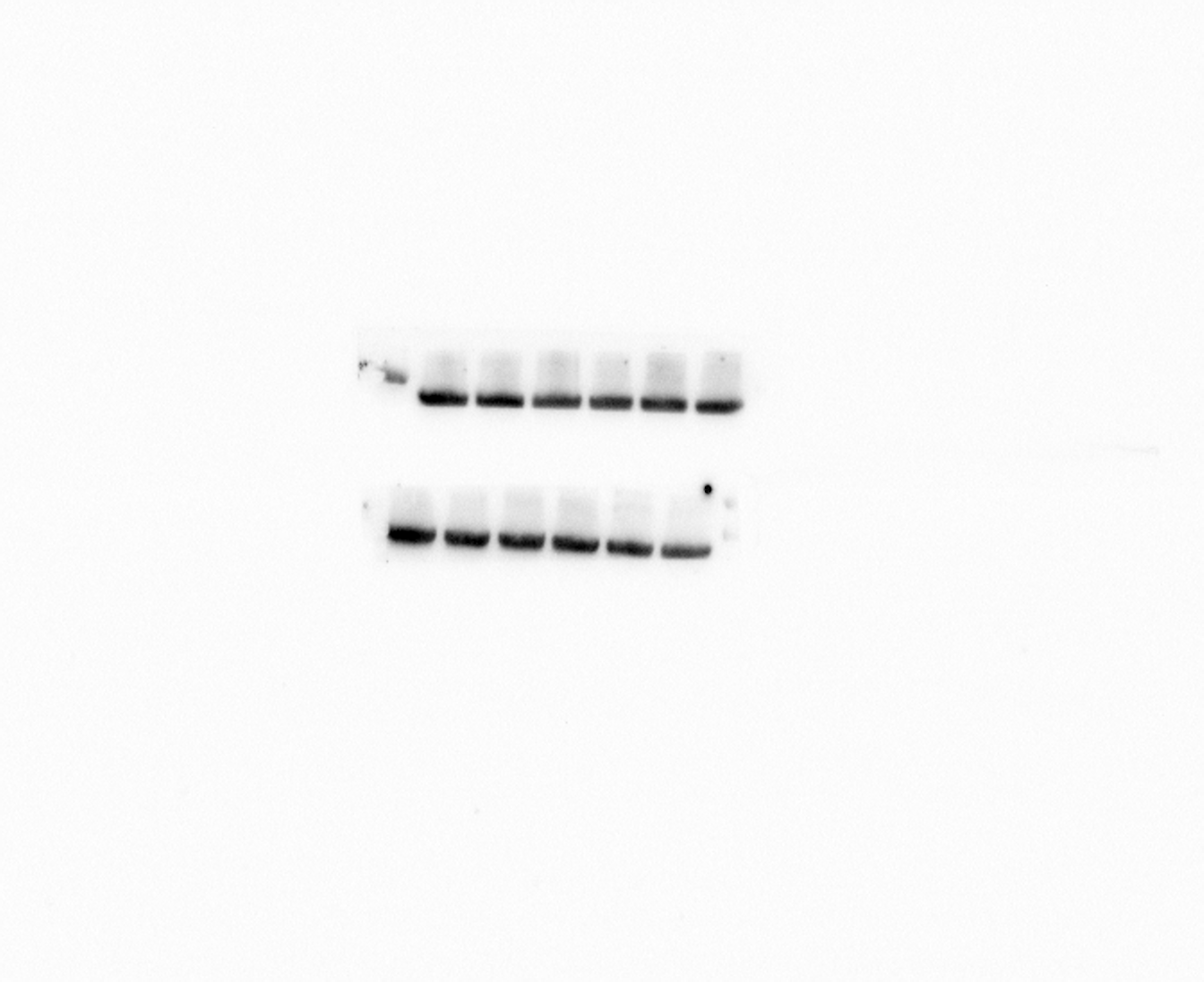

Supplement: Supplementary file 2 [file DataSheet2.ZIP › data1/2022.8.16/GAPDH/tubulin.1.2.10s.Tif]

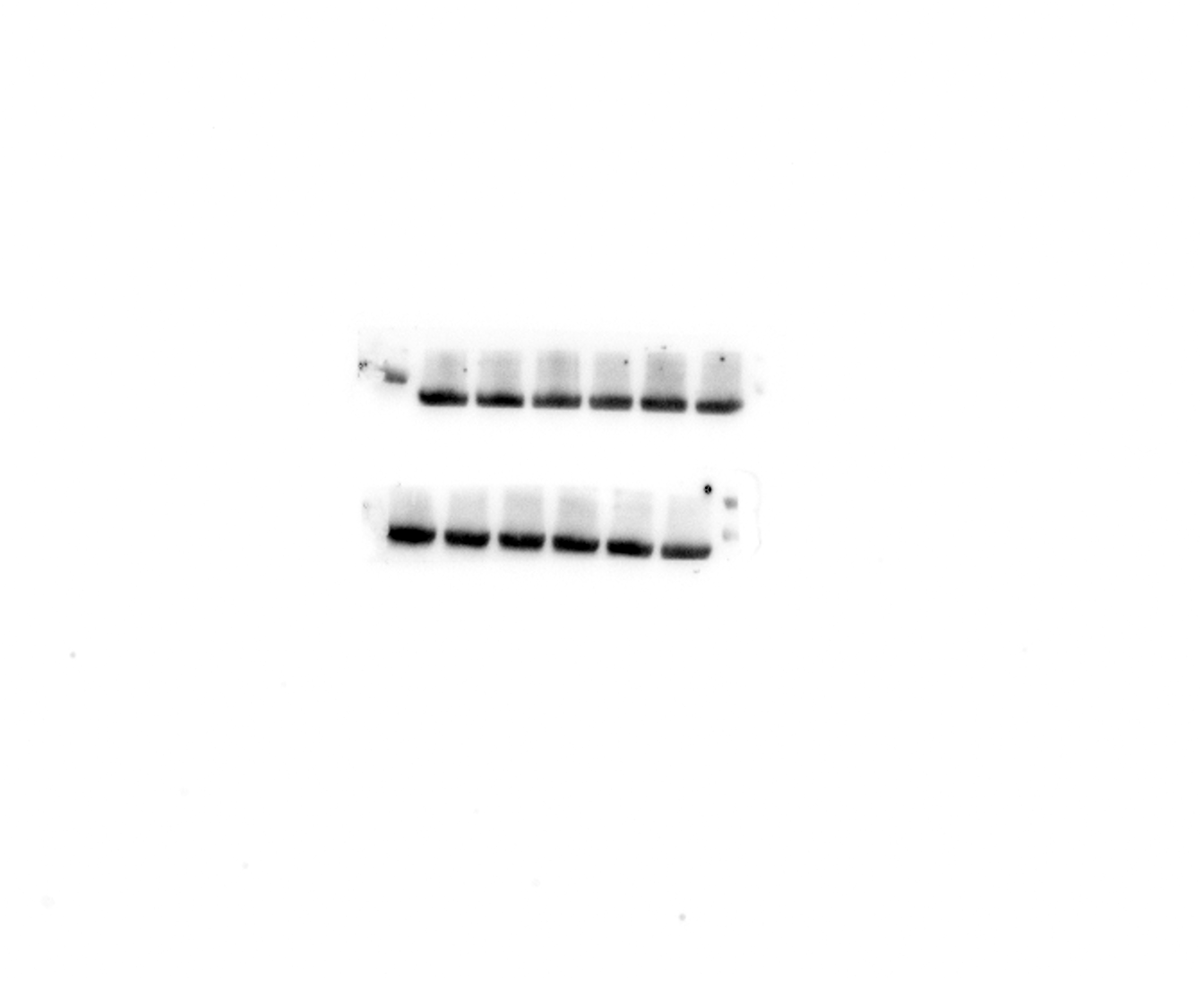

Supplement: Supplementary file 2 [file DataSheet2.ZIP › data1/2022.8.16/GAPDH/tubulin.1.2.15s.Tif]

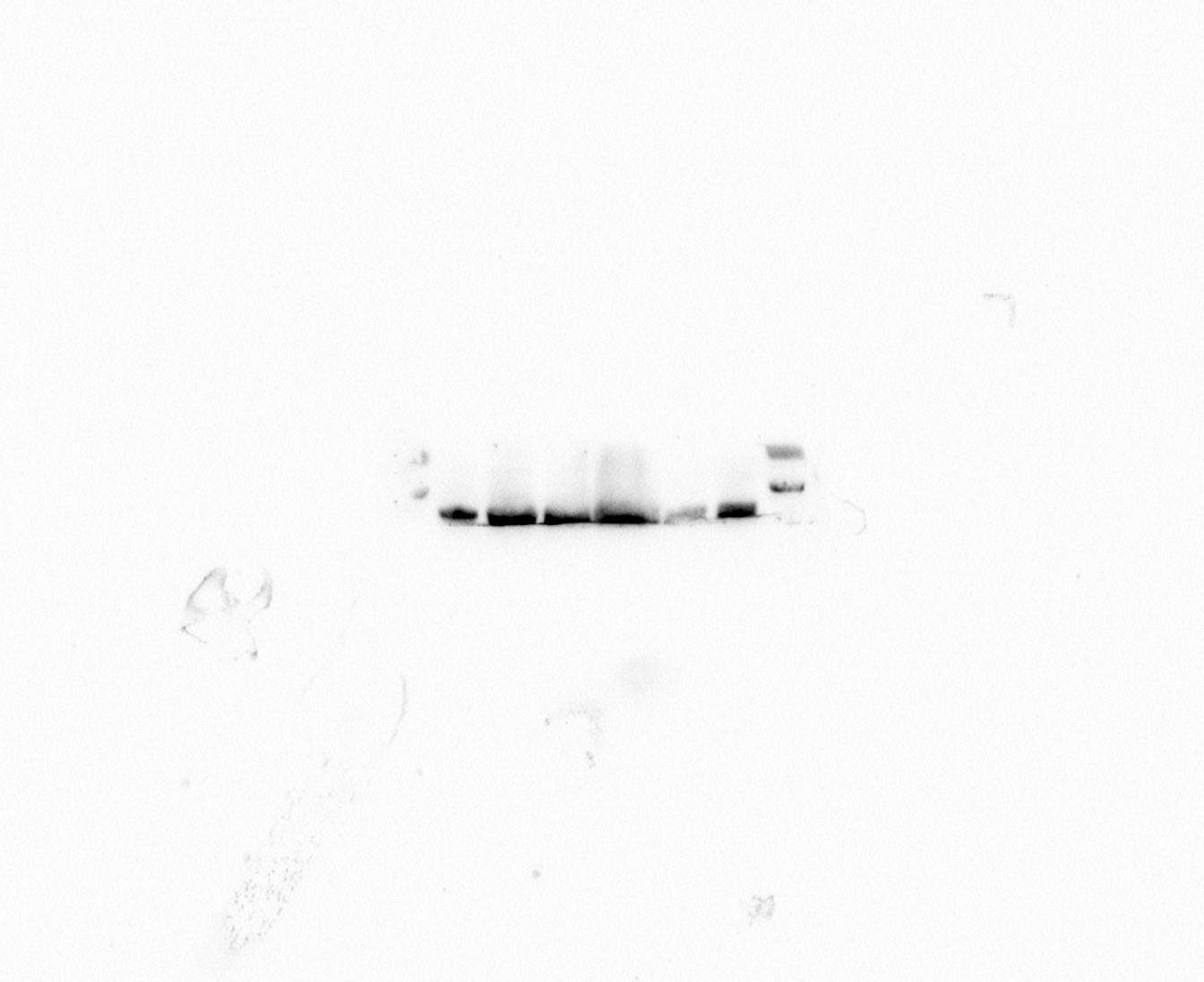

Supplement: Supplementary file 2 [file DataSheet2.ZIP › data1/2022.8.16/GAPDH/tubulin.2.Tif]

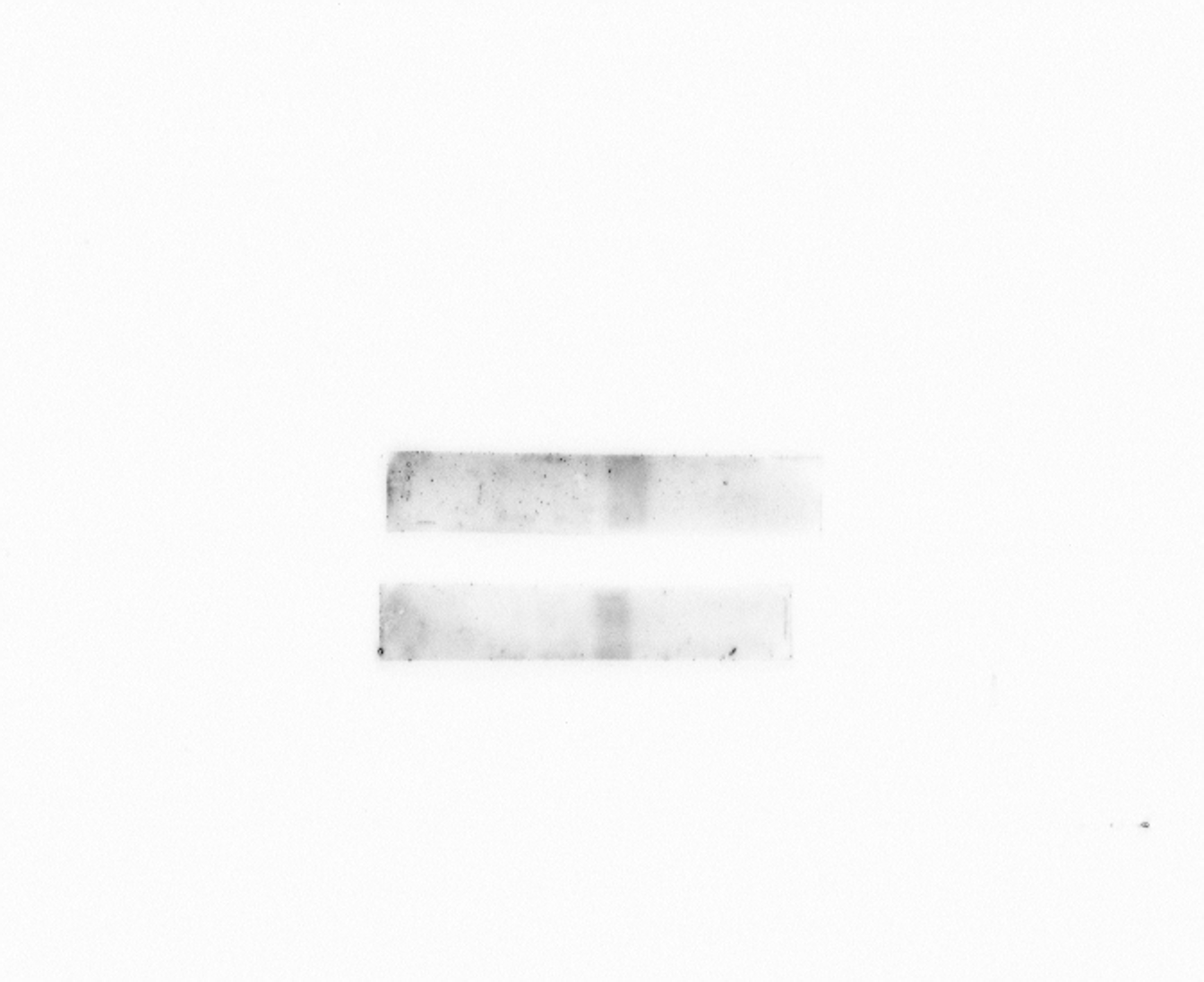

Supplement: Supplementary file 2 [file DataSheet2.ZIP › data1/2022.8.16/GRP78/10S.Tif]

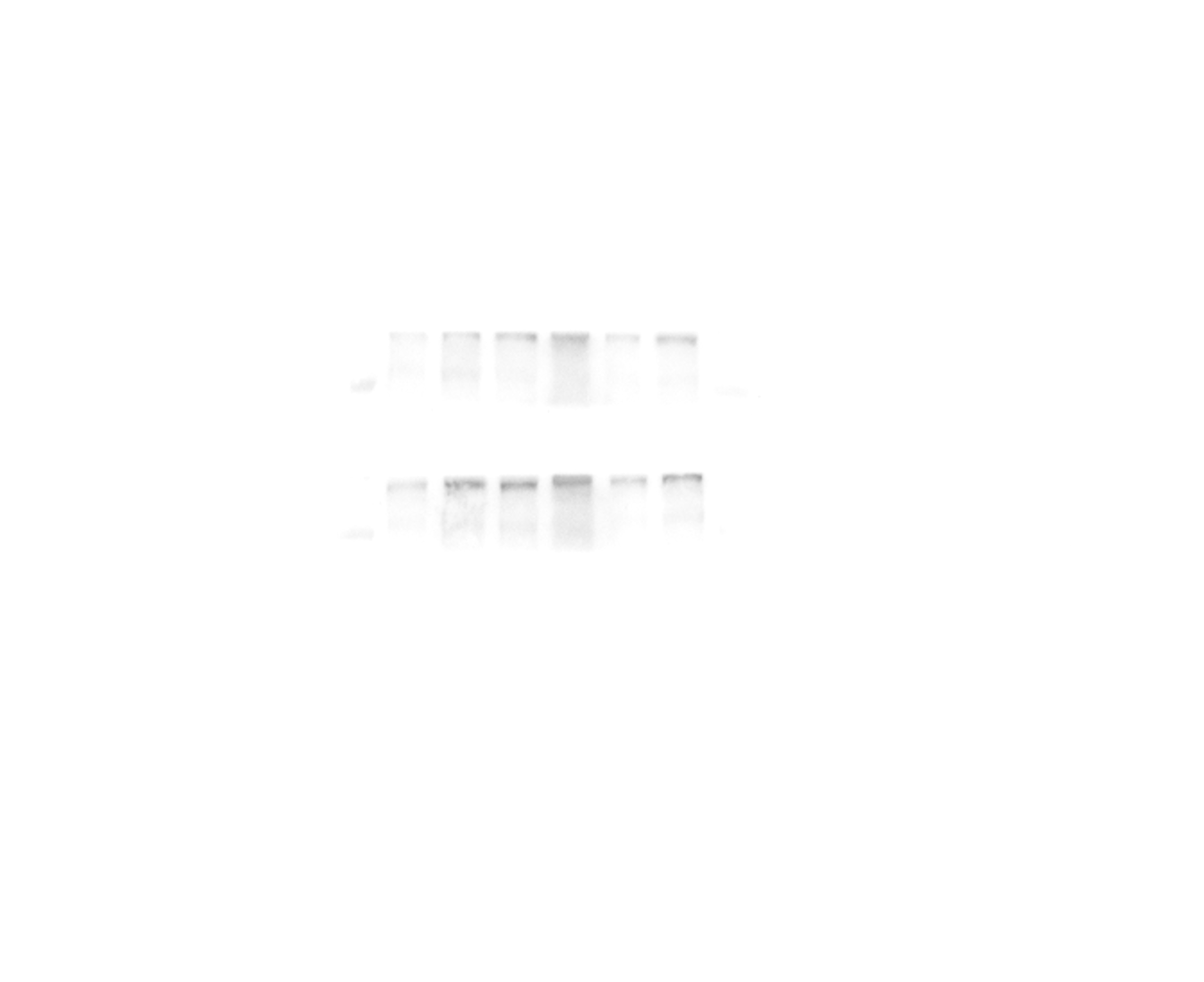

Supplement: Supplementary file 2 [file DataSheet2.ZIP › data1/2022.8.16/P-EIF2A/10S.Tif]

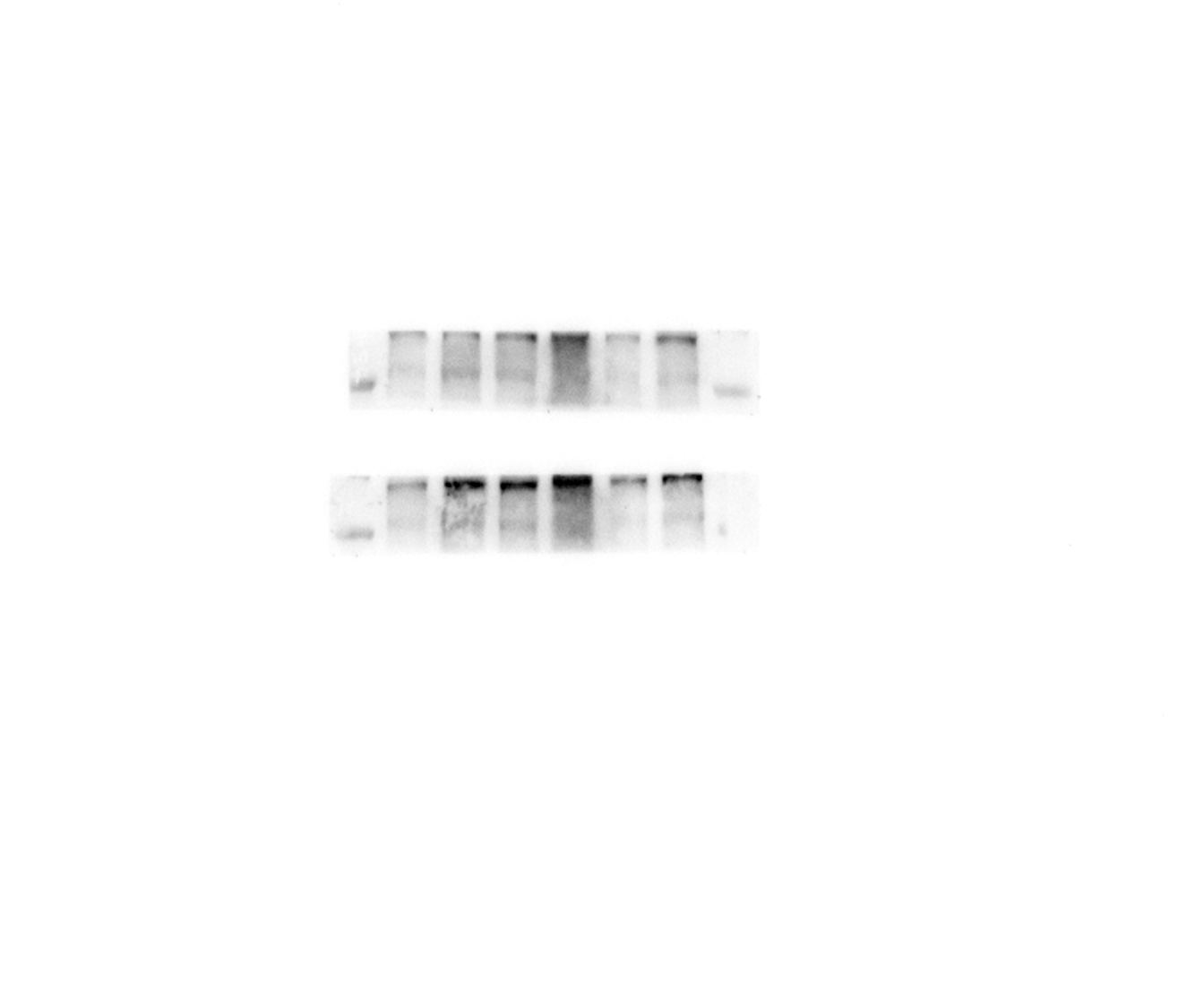

Supplement: Supplementary file 2 [file DataSheet2.ZIP › data1/2022.8.16/P-EIF2A/4S.Tif]

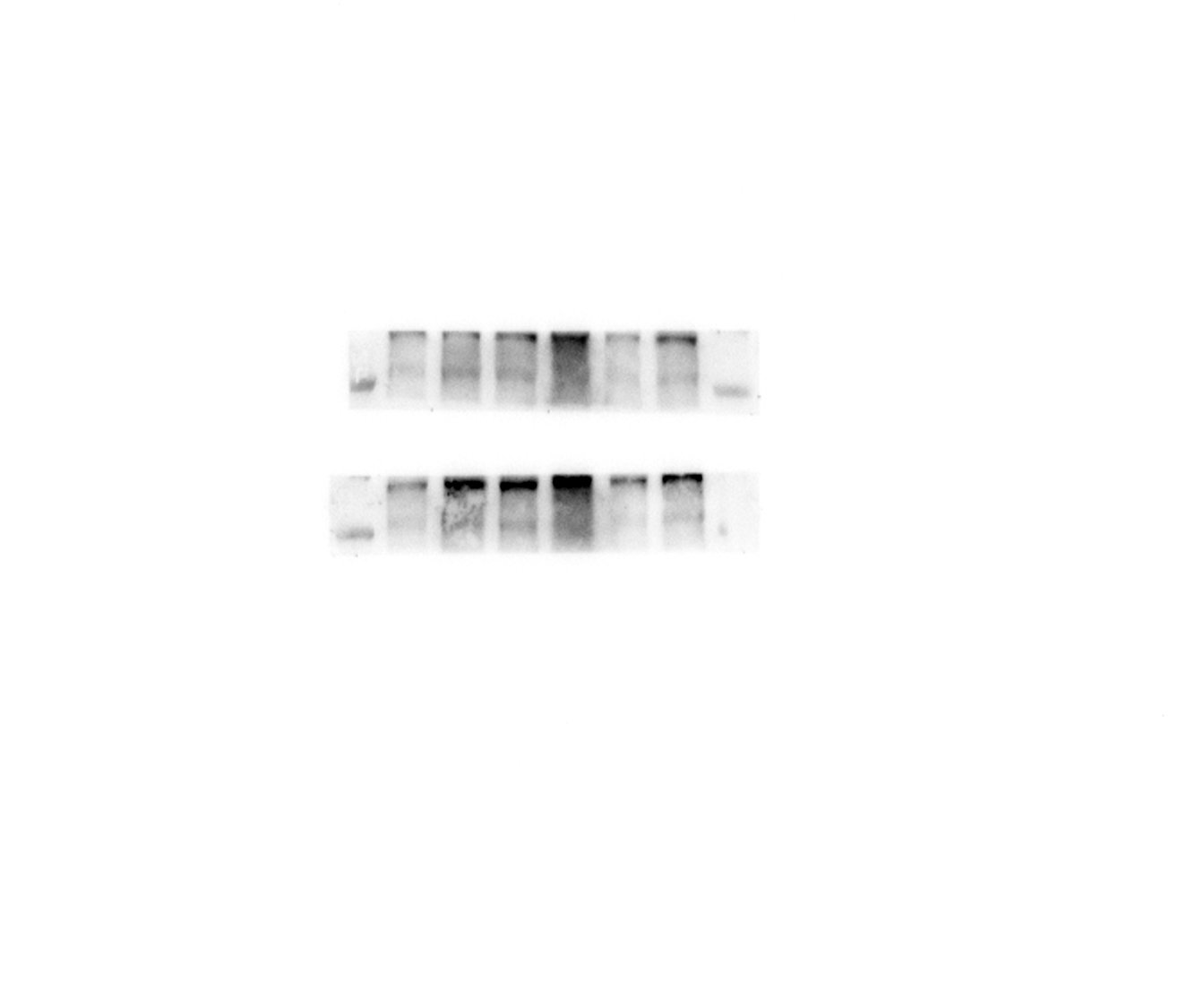

Supplement: Supplementary file 2 [file DataSheet2.ZIP › data1/2022.8.16/P-EIF2A/5S.Tif]

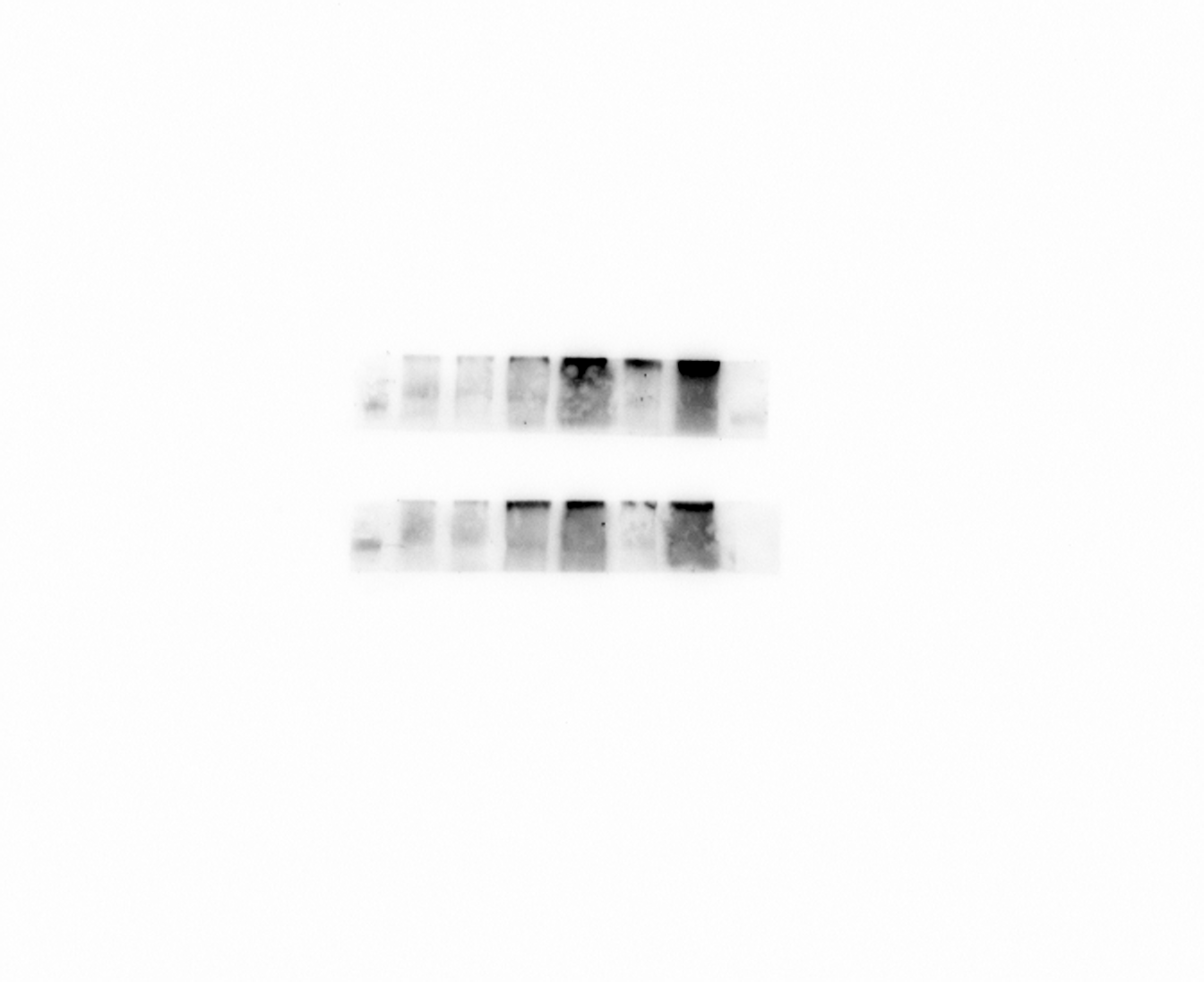

Supplement: Supplementary file 2 [file DataSheet2.ZIP › data1/2022.8.16/P-PERK/10S.Tif]

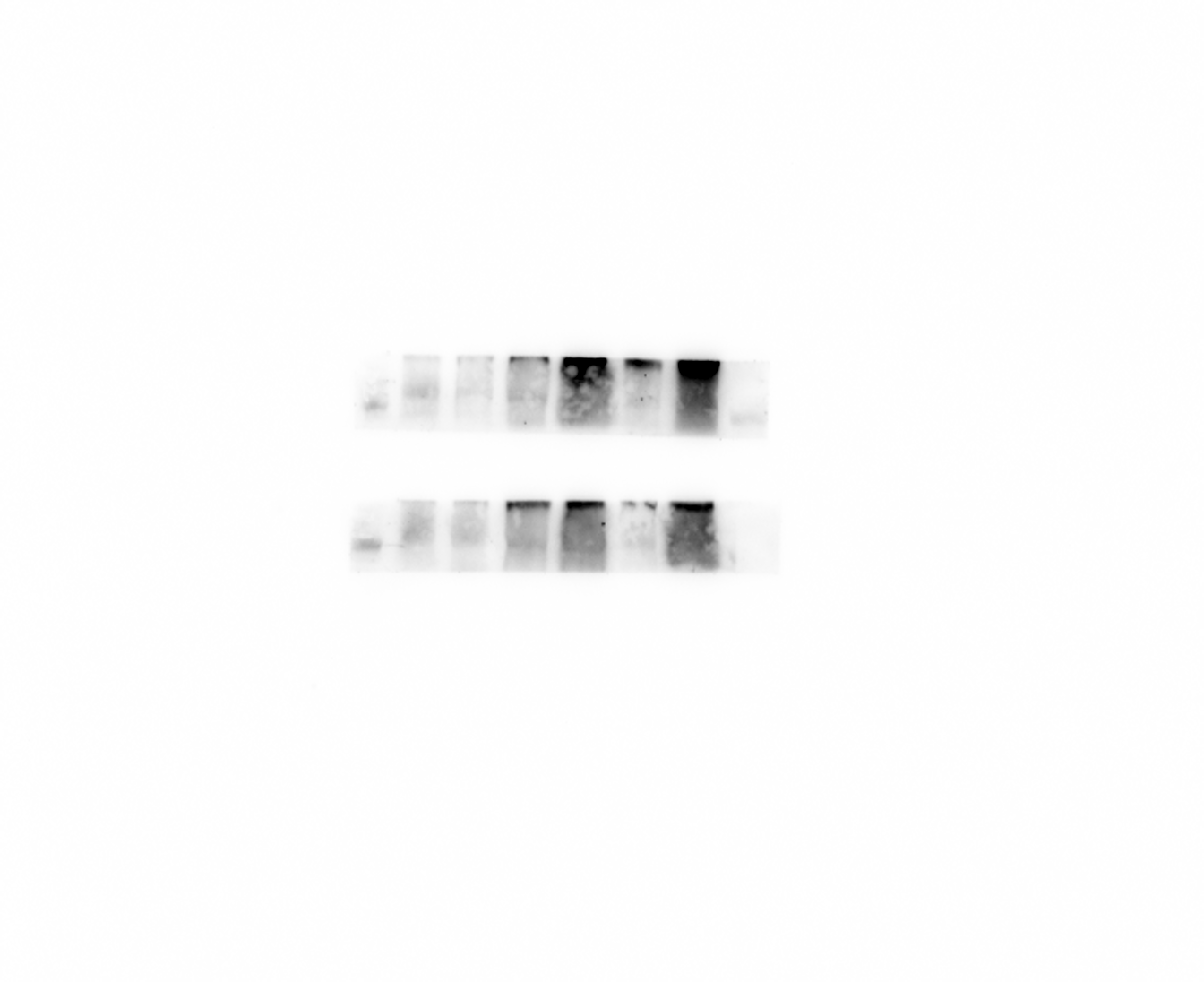

Supplement: Supplementary file 2 [file DataSheet2.ZIP › data1/2022.8.16/P-PERK/30S.Tif]

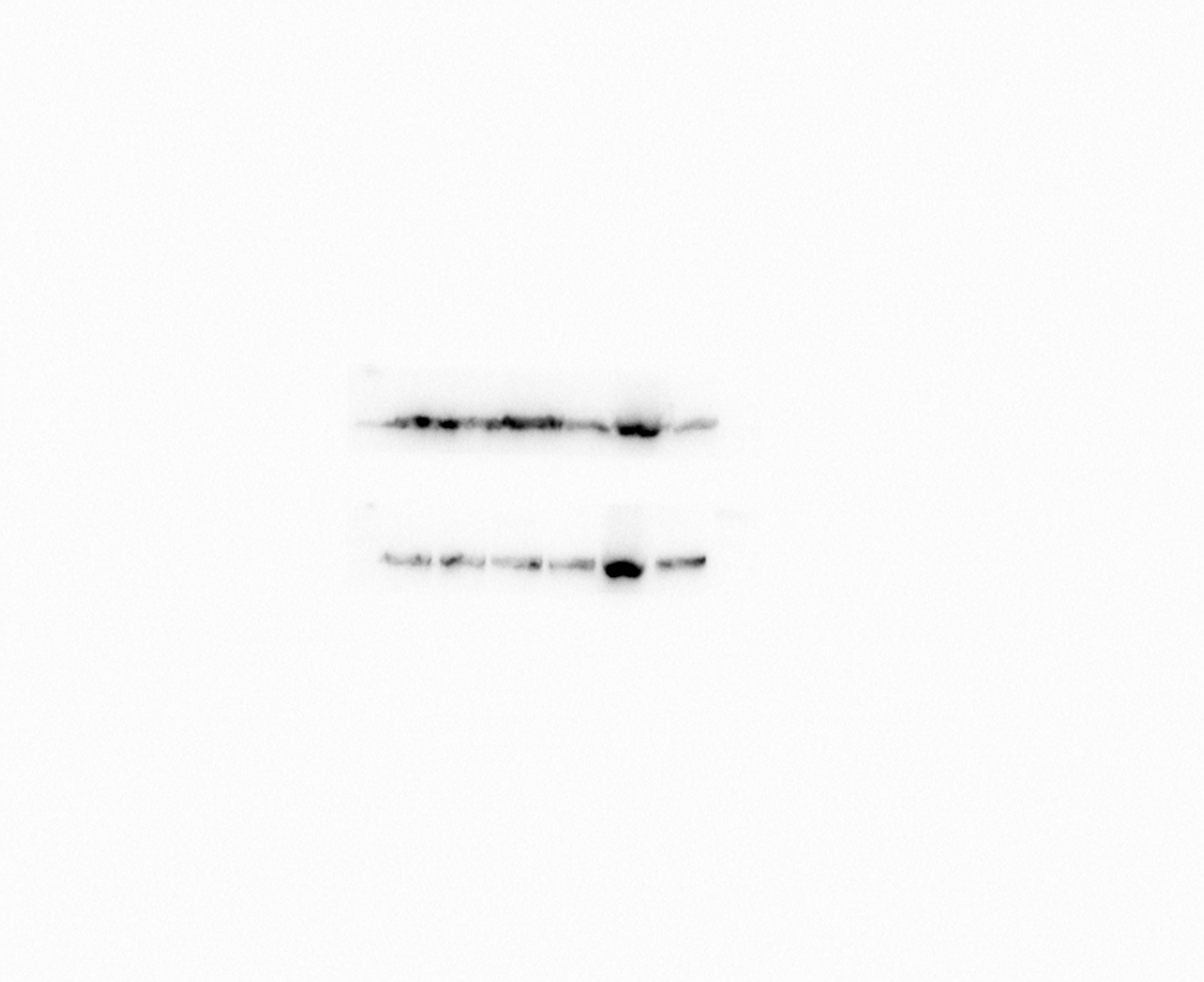

Supplement: Supplementary file 2 [file DataSheet2.ZIP › data1/2022.8.16/sirt1/0.5s.Tif]

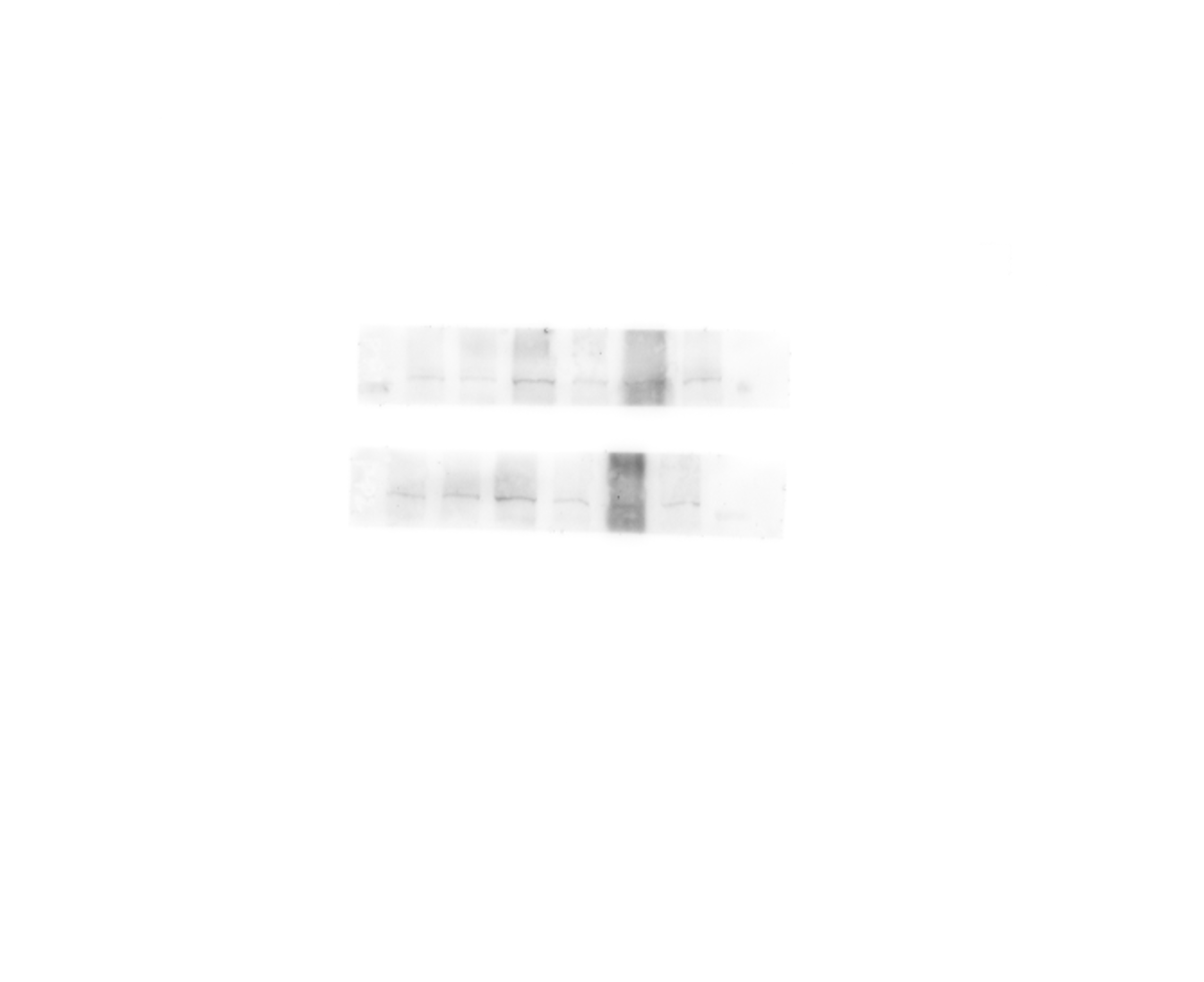

Supplement: Supplementary file 2 [file DataSheet2.ZIP › data1/2022.8.16/sirt1/30S.Tif]

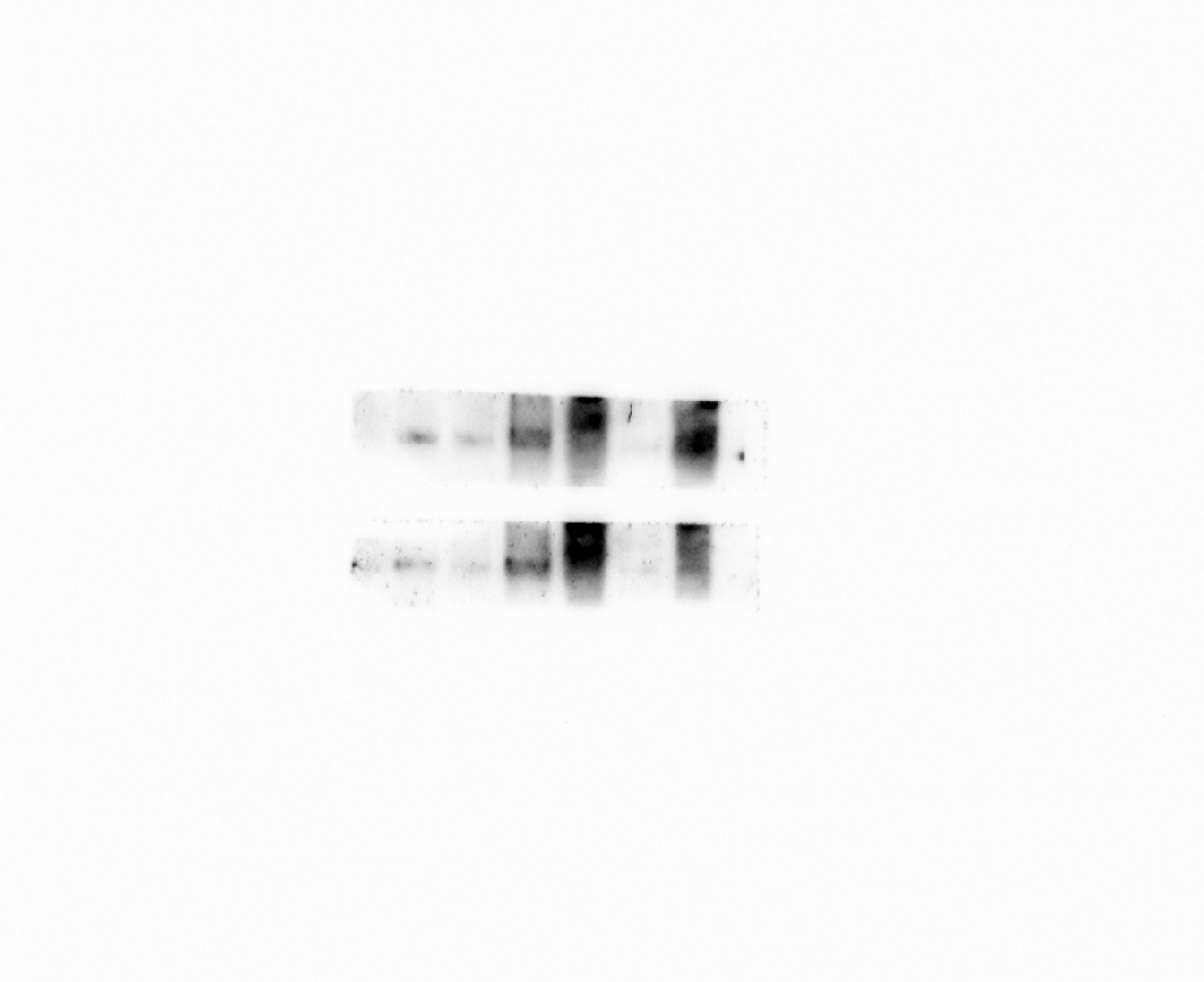

Supplement: Supplementary file 2 [file DataSheet2.ZIP › data1/2022.8.18/atf4/10s.Tif]

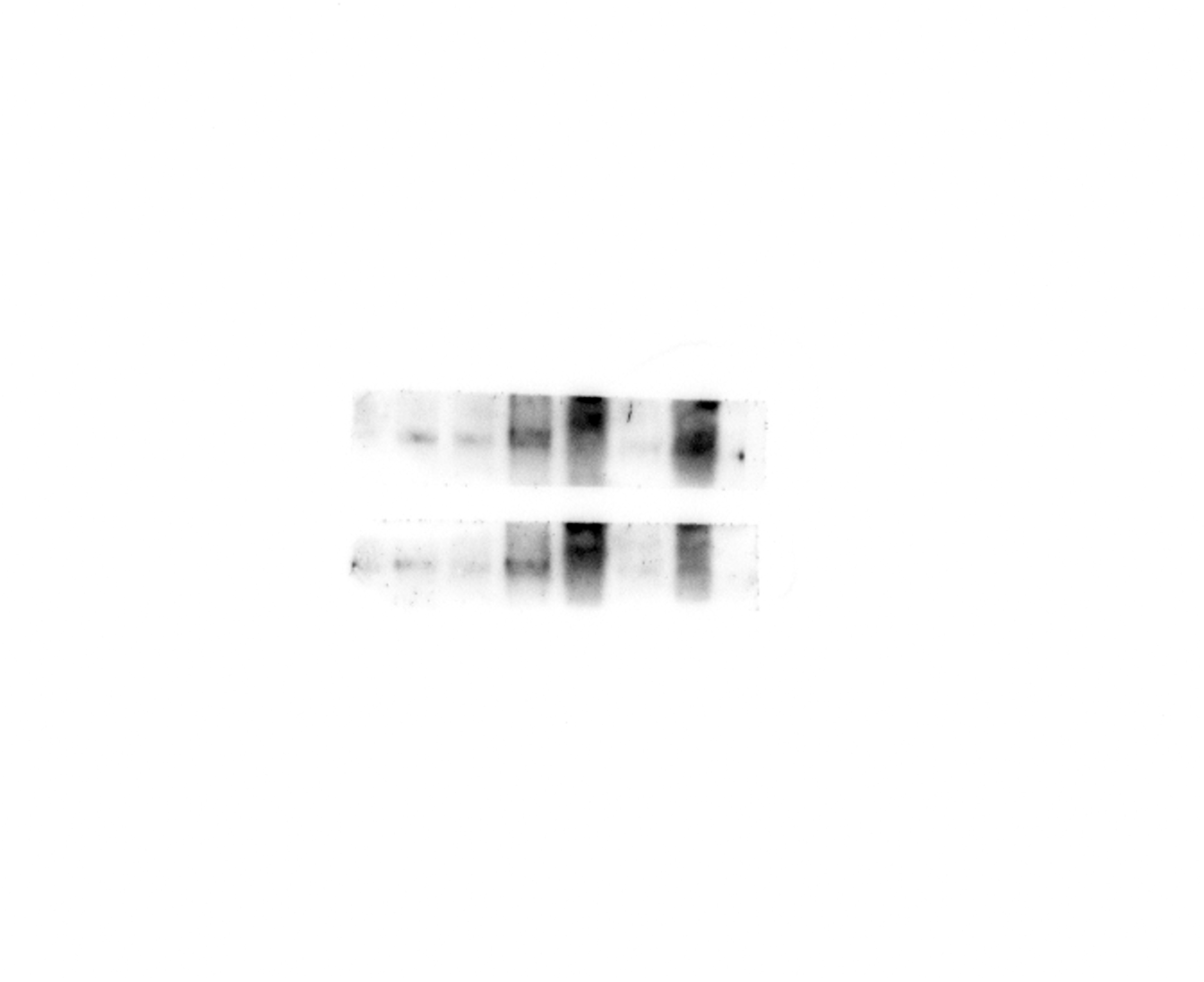

Supplement: Supplementary file 2 [file DataSheet2.ZIP › data1/2022.8.18/atf4/7s.Tif]

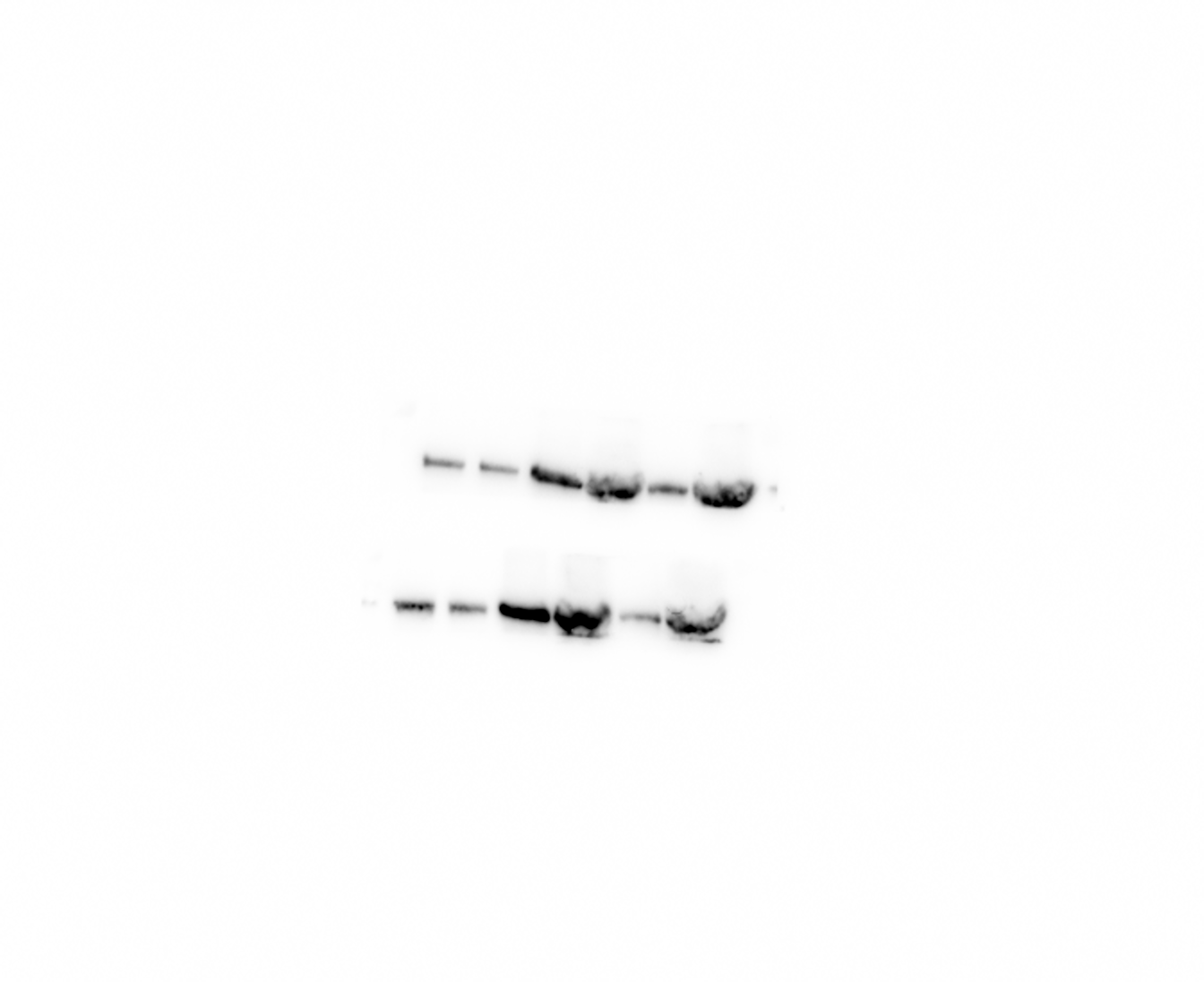

Supplement: Supplementary file 2 [file DataSheet2.ZIP › data1/2022.8.18/chop/10s.Tif]

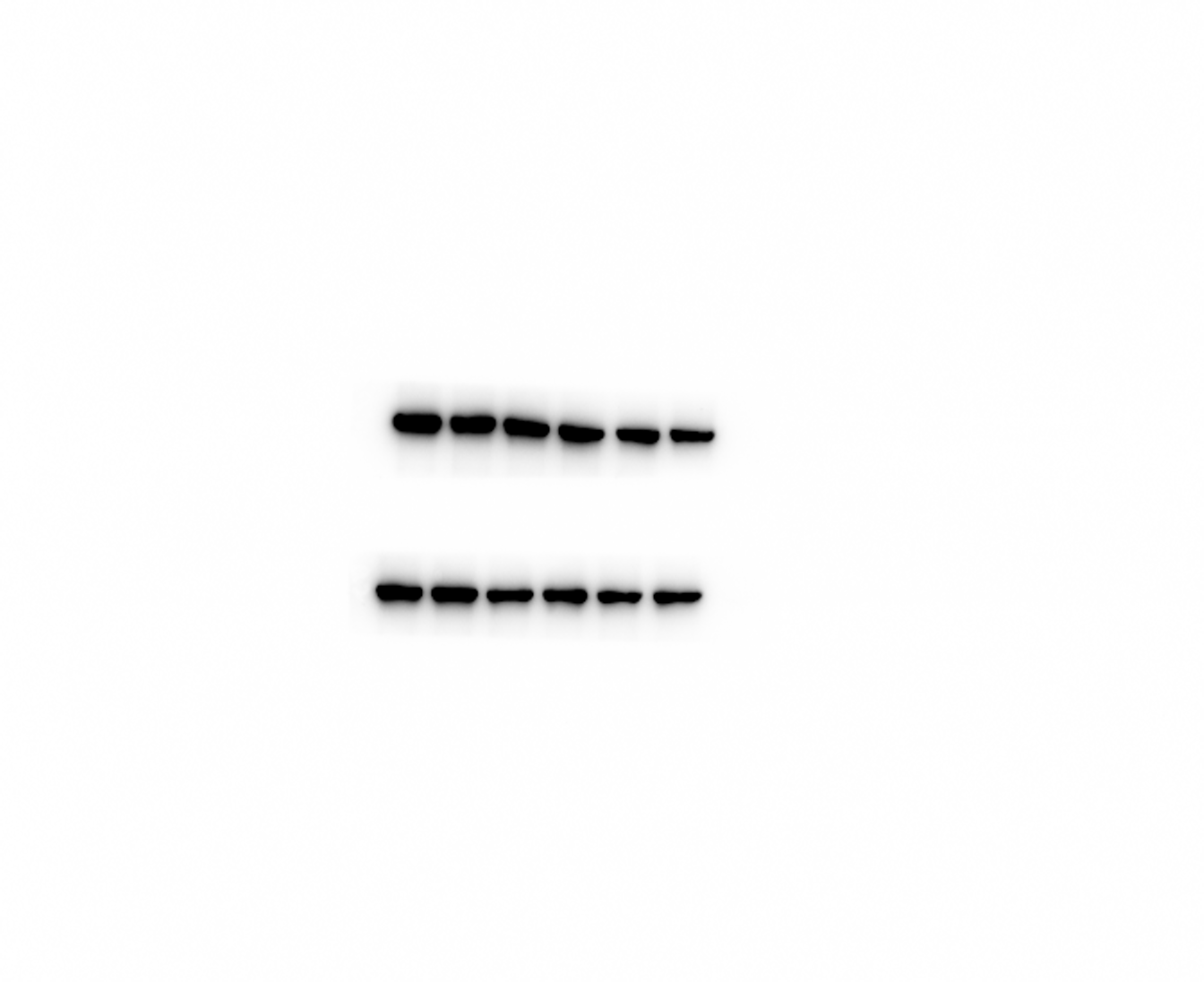

Supplement: Supplementary file 2 [file DataSheet2.ZIP › data1/2022.8.18/GAPDH/10s.Tif]

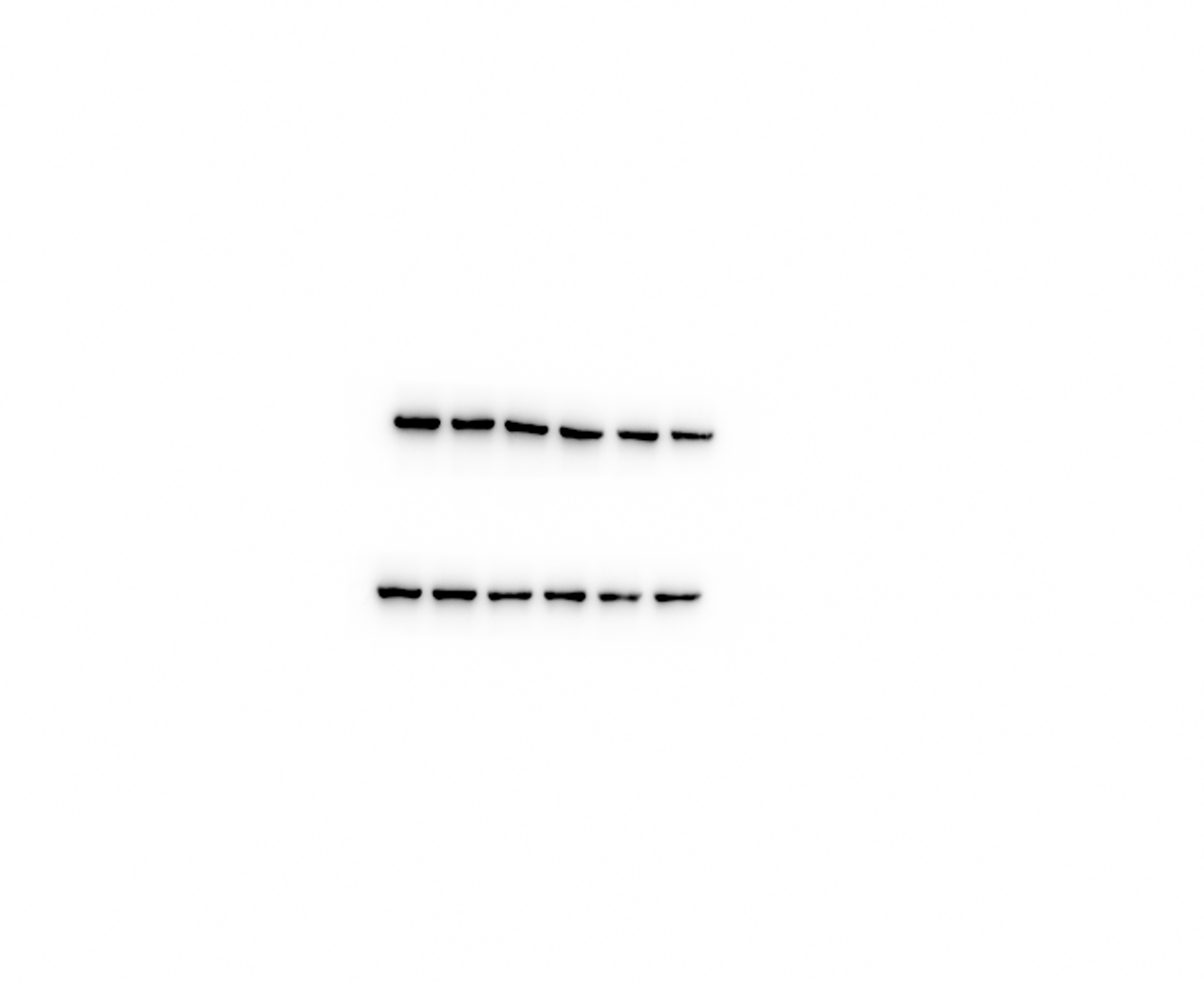

Supplement: Supplementary file 2 [file DataSheet2.ZIP › data1/2022.8.18/GAPDH/5s.Tif]

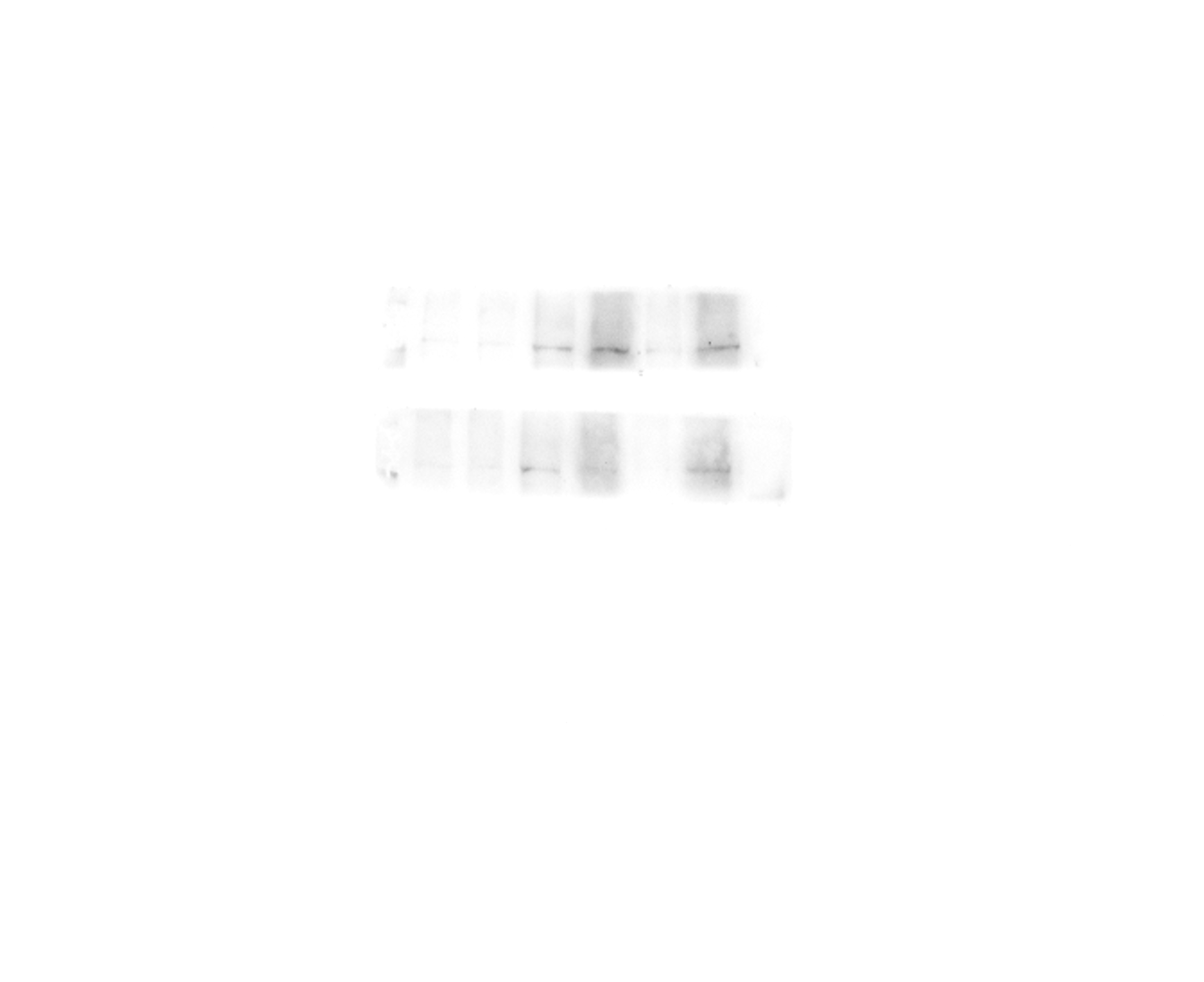

Supplement: Supplementary file 2 [file DataSheet2.ZIP › data1/2022.8.18/GRP78.2/10S.Tif]

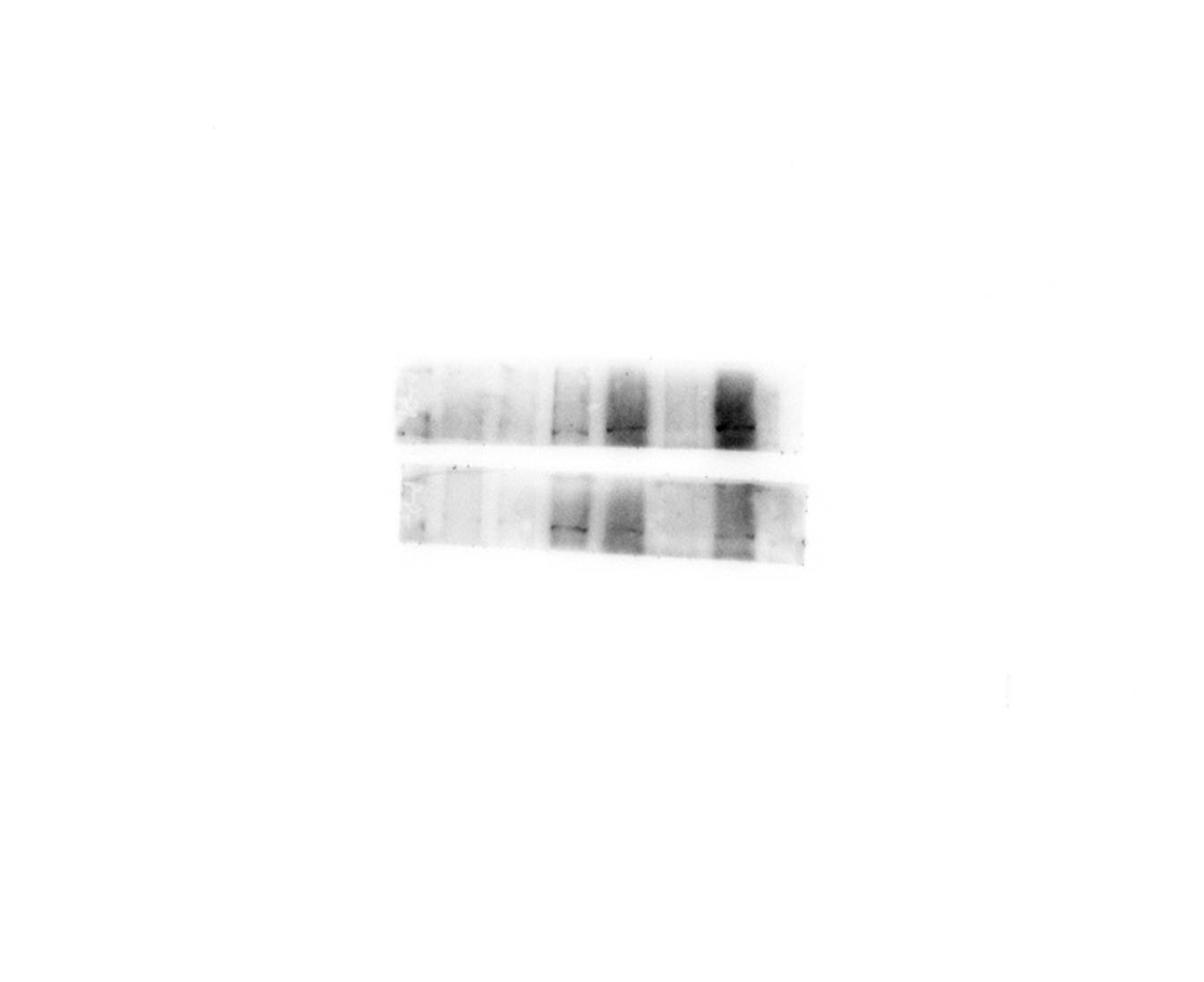

Supplement: Supplementary file 2 [file DataSheet2.ZIP › data1/2022.8.18/GRP78.2/15.Tif]

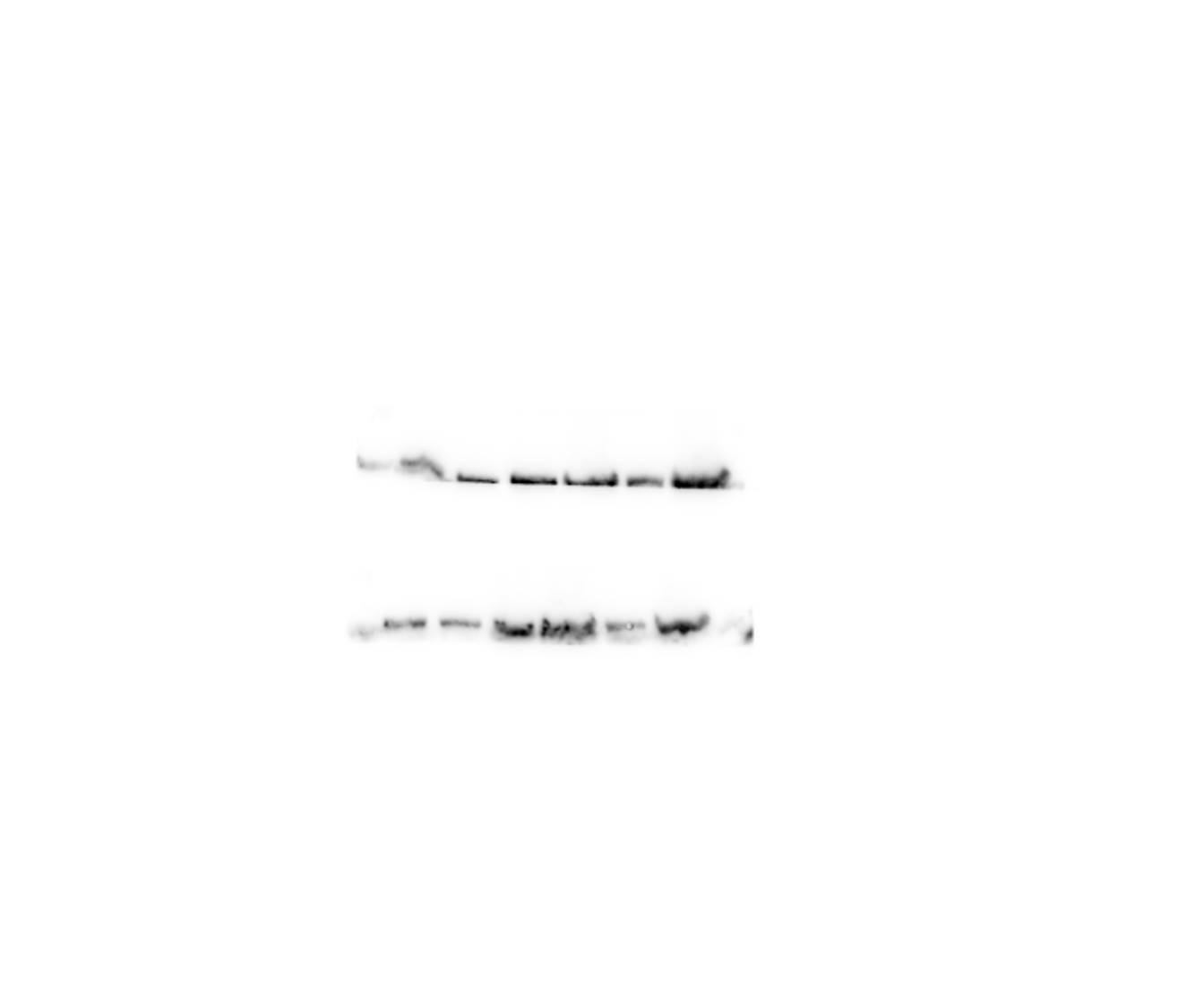

Supplement: Supplementary file 2 [file DataSheet2.ZIP › data1/2022.8.18/grp78/5s.Tif]

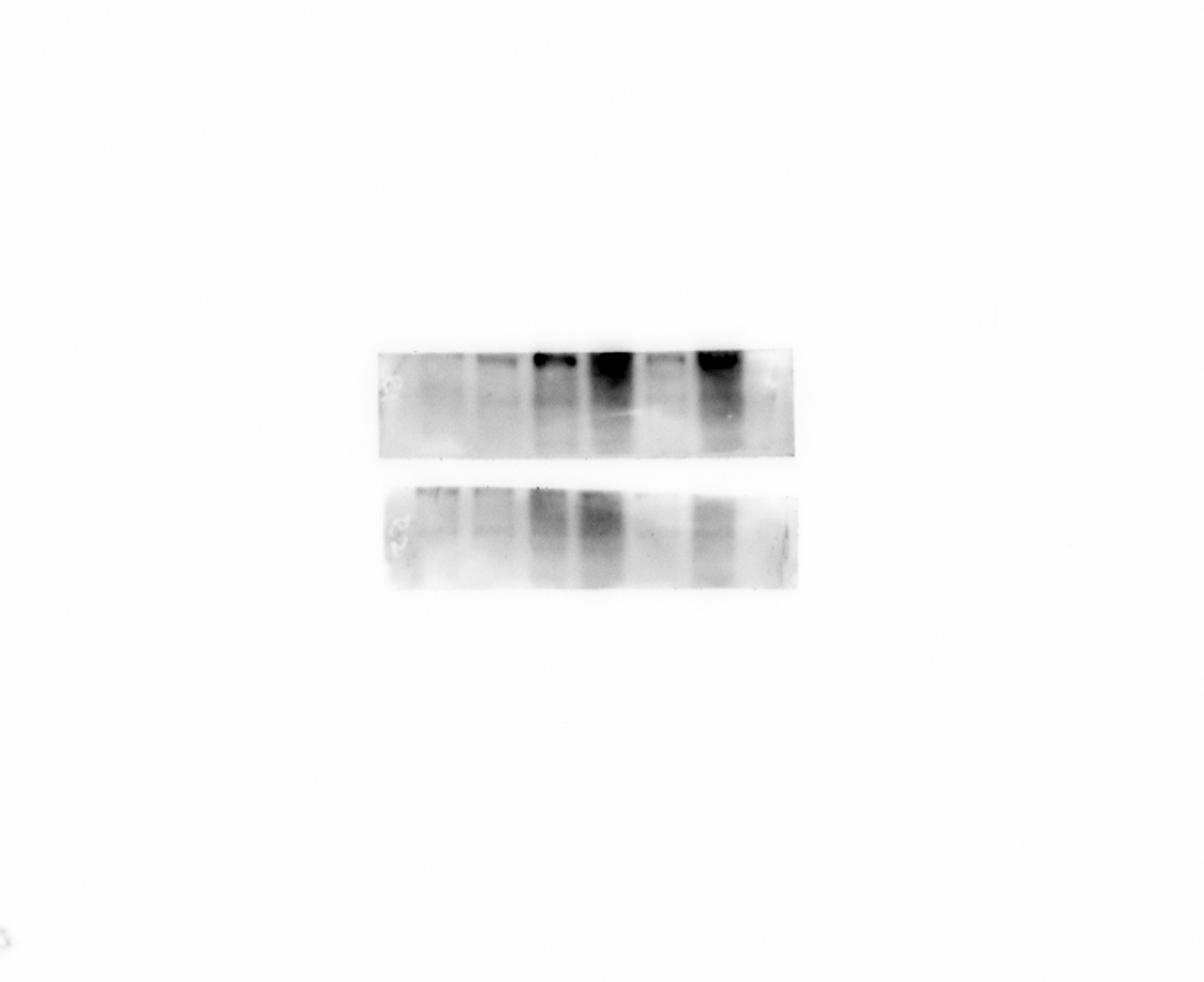

Supplement: Supplementary file 2 [file DataSheet2.ZIP › data1/2022.8.18/P-EIF2A/10S.Tif]

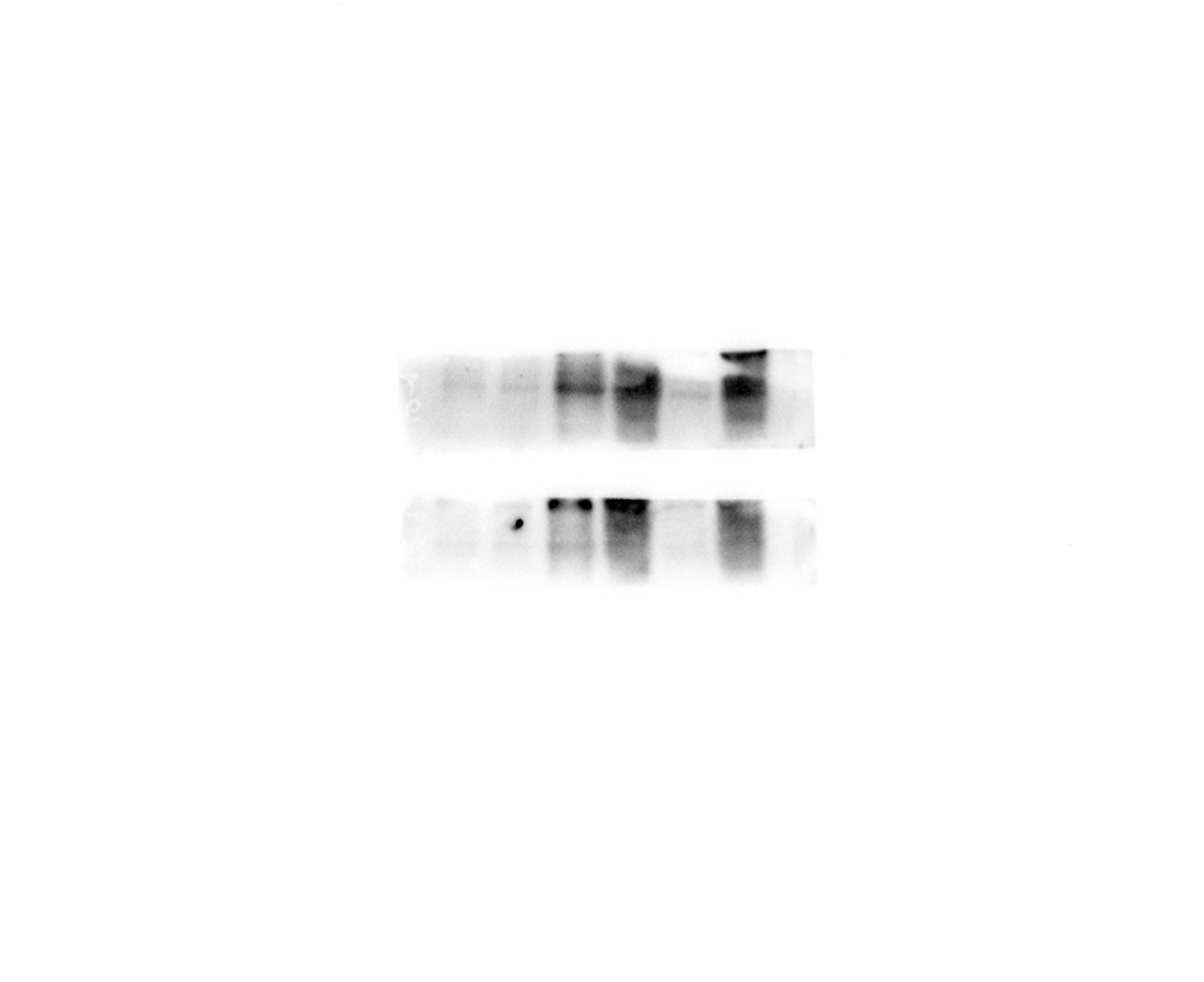

Supplement: Supplementary file 2 [file DataSheet2.ZIP › data1/2022.8.18/p-eif2a2/3s.Tif]

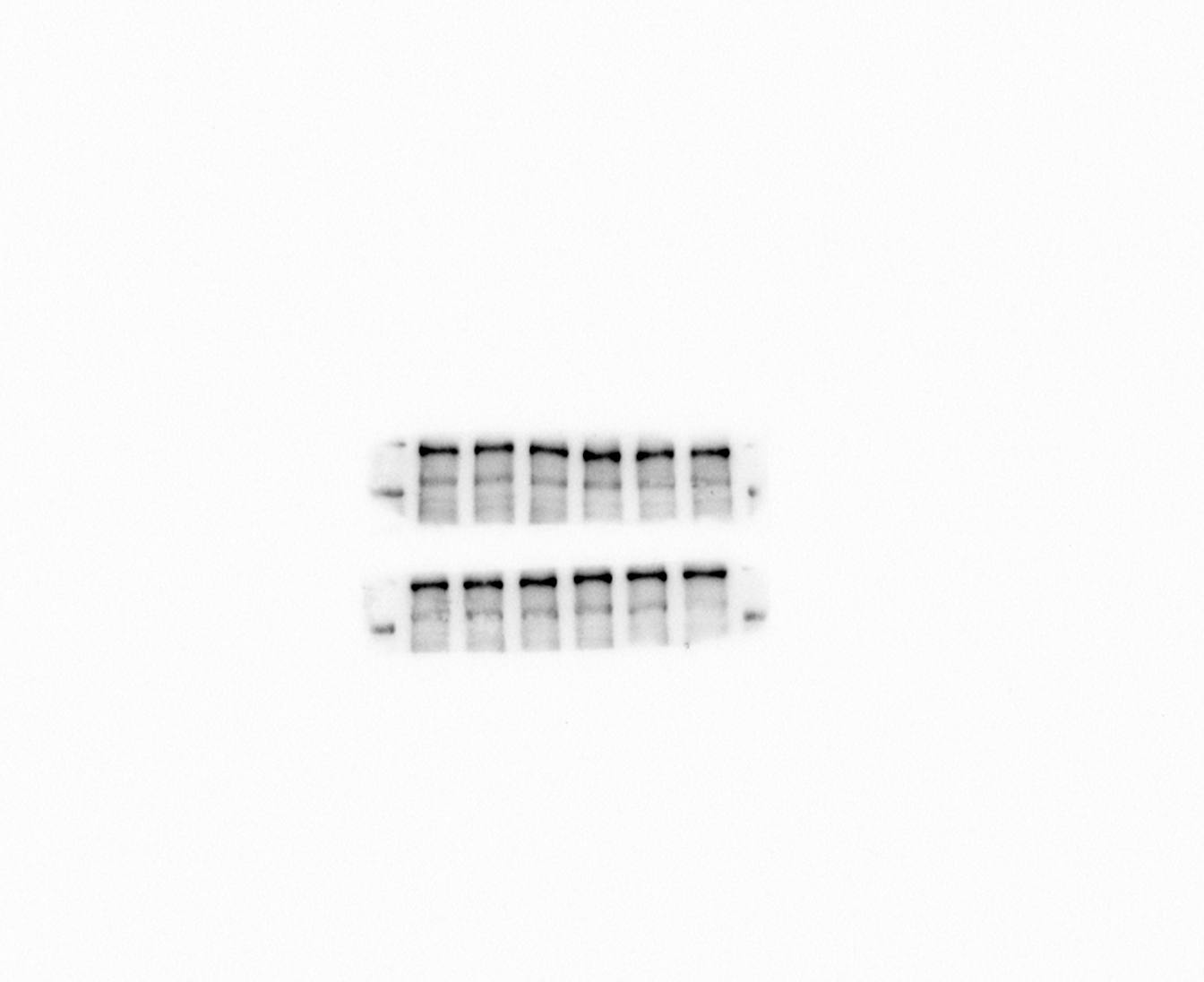

Supplement: Supplementary file 2 [file DataSheet2.ZIP › data1/2022.8.18/perk/10s.Tif]

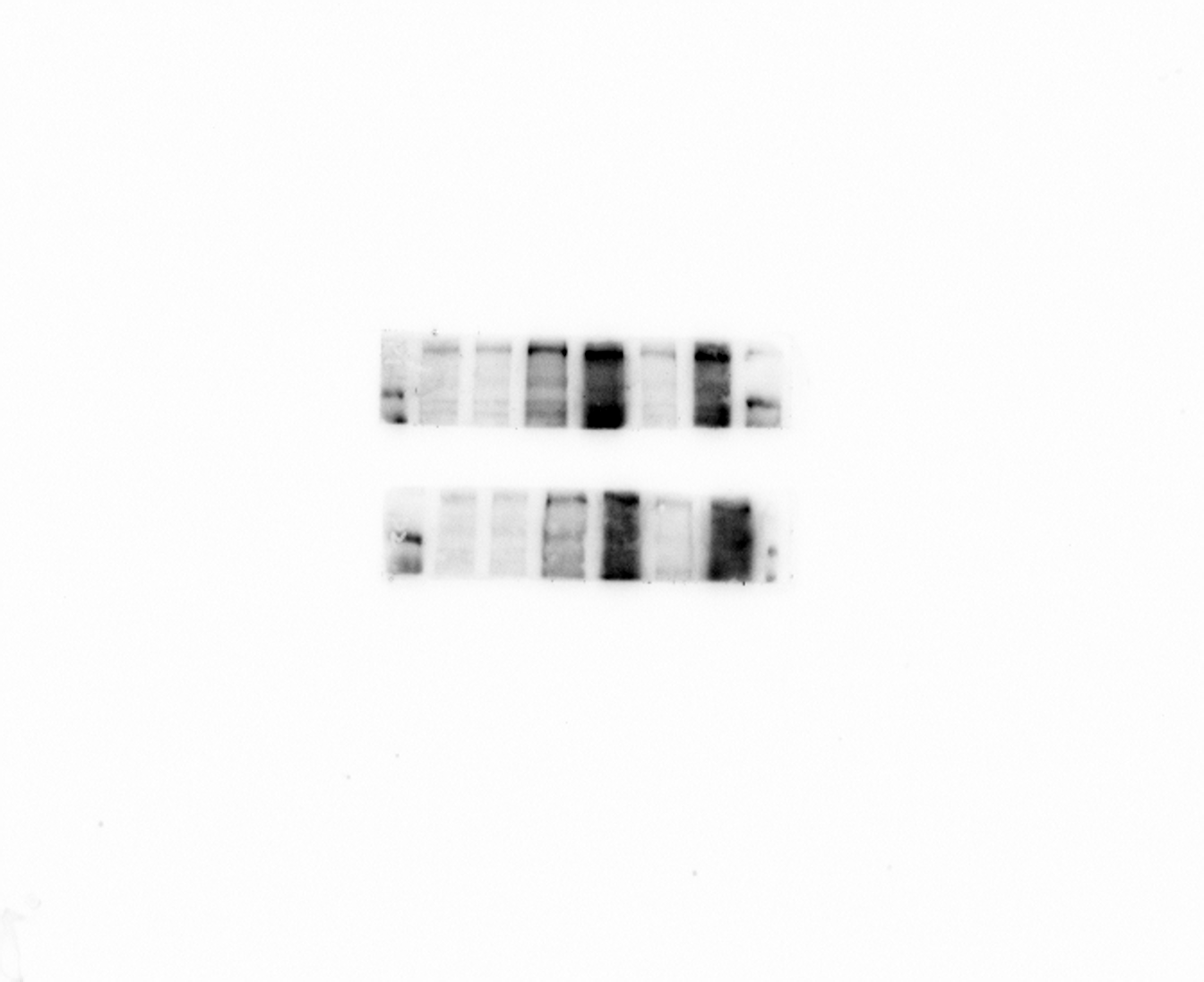

Supplement: Supplementary file 2 [file DataSheet2.ZIP › data1/2022.8.18/p-PERK/10S.Tif]

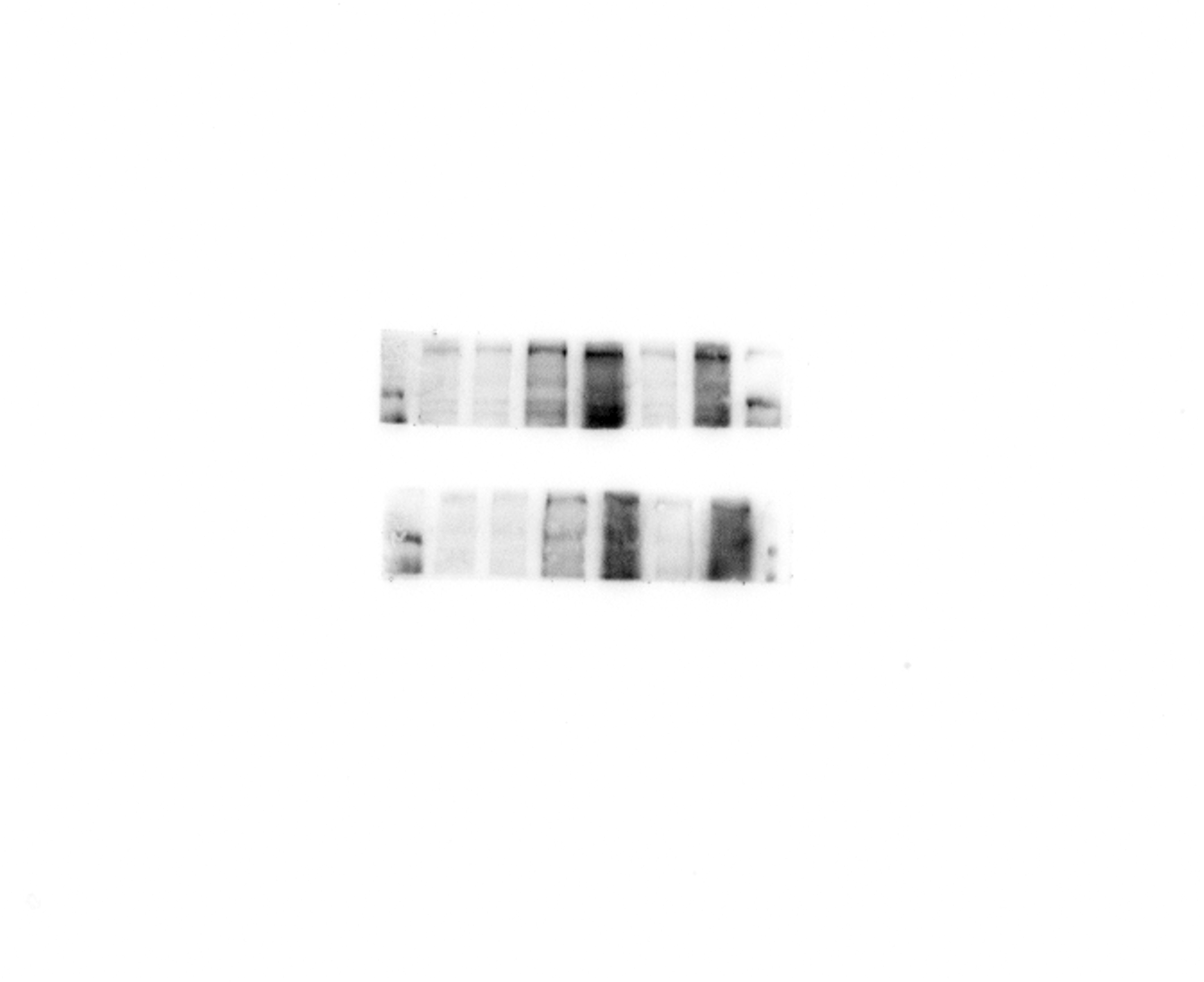

Supplement: Supplementary file 2 [file DataSheet2.ZIP › data1/2022.8.18/p-PERK/6S.Tif]

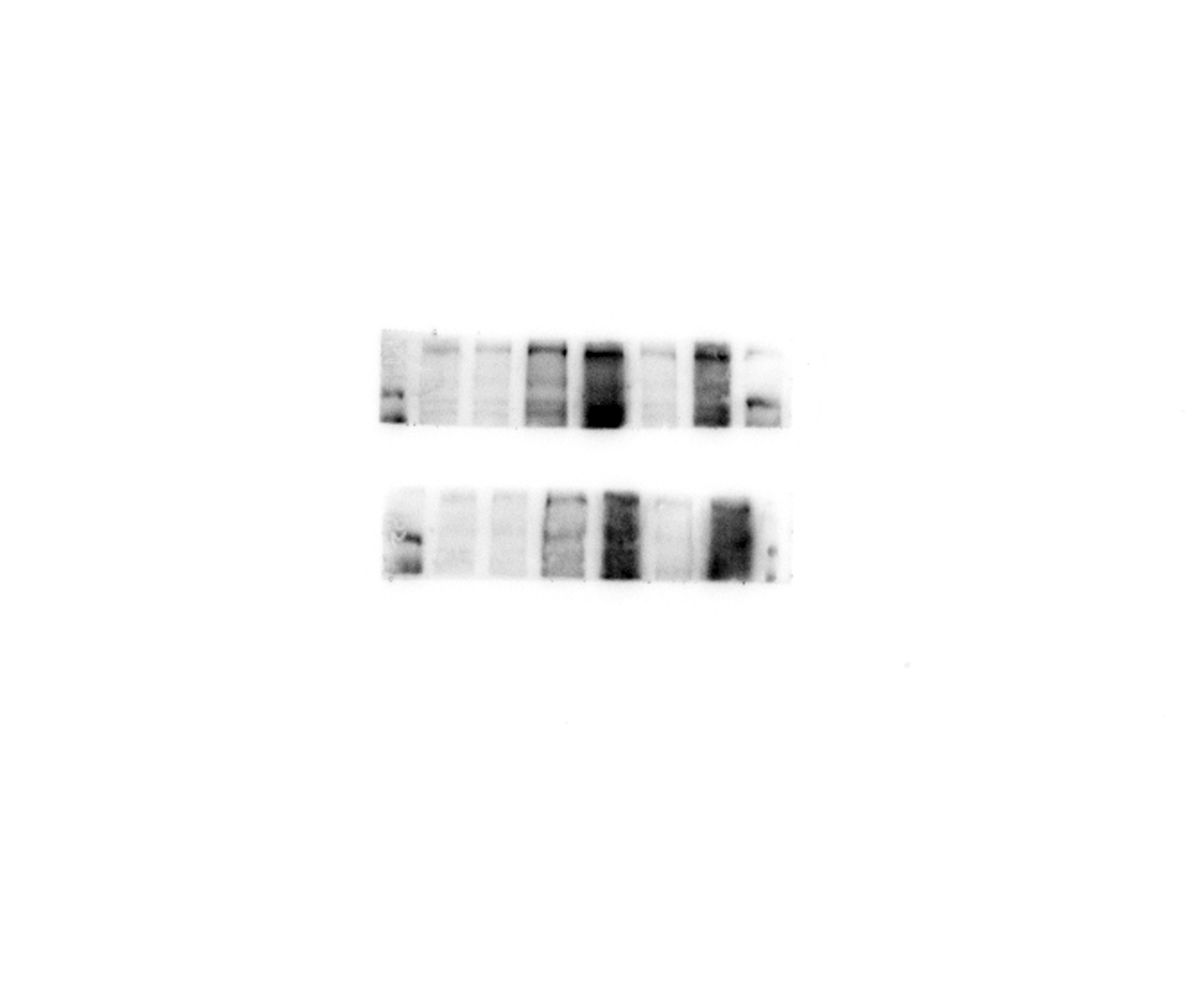

Supplement: Supplementary file 2 [file DataSheet2.ZIP › data1/2022.8.18/p-PERK/7S.Tif]

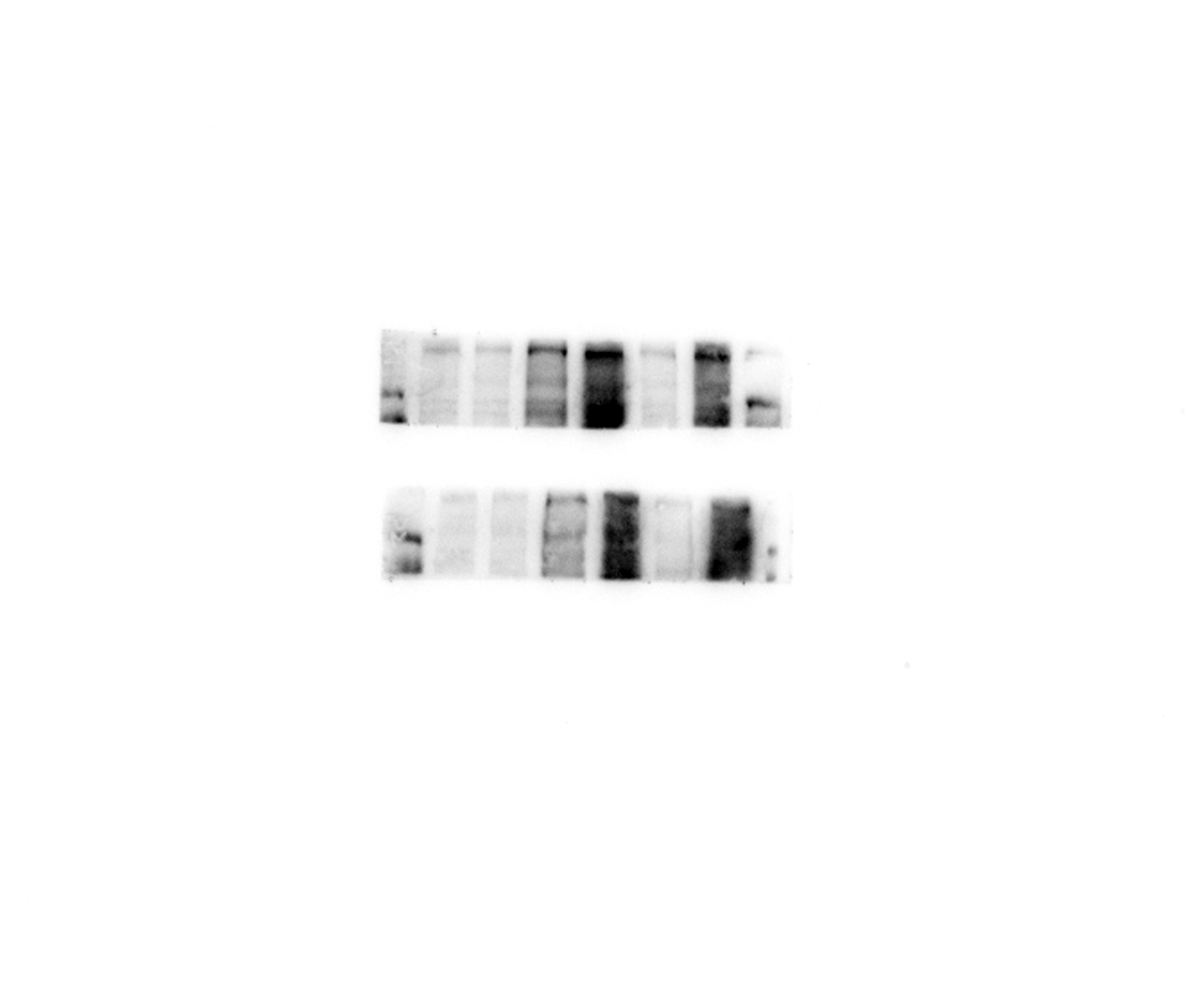

Supplement: Supplementary file 2 [file DataSheet2.ZIP › data1/2022.8.18/p-PERK/8S.Tif]

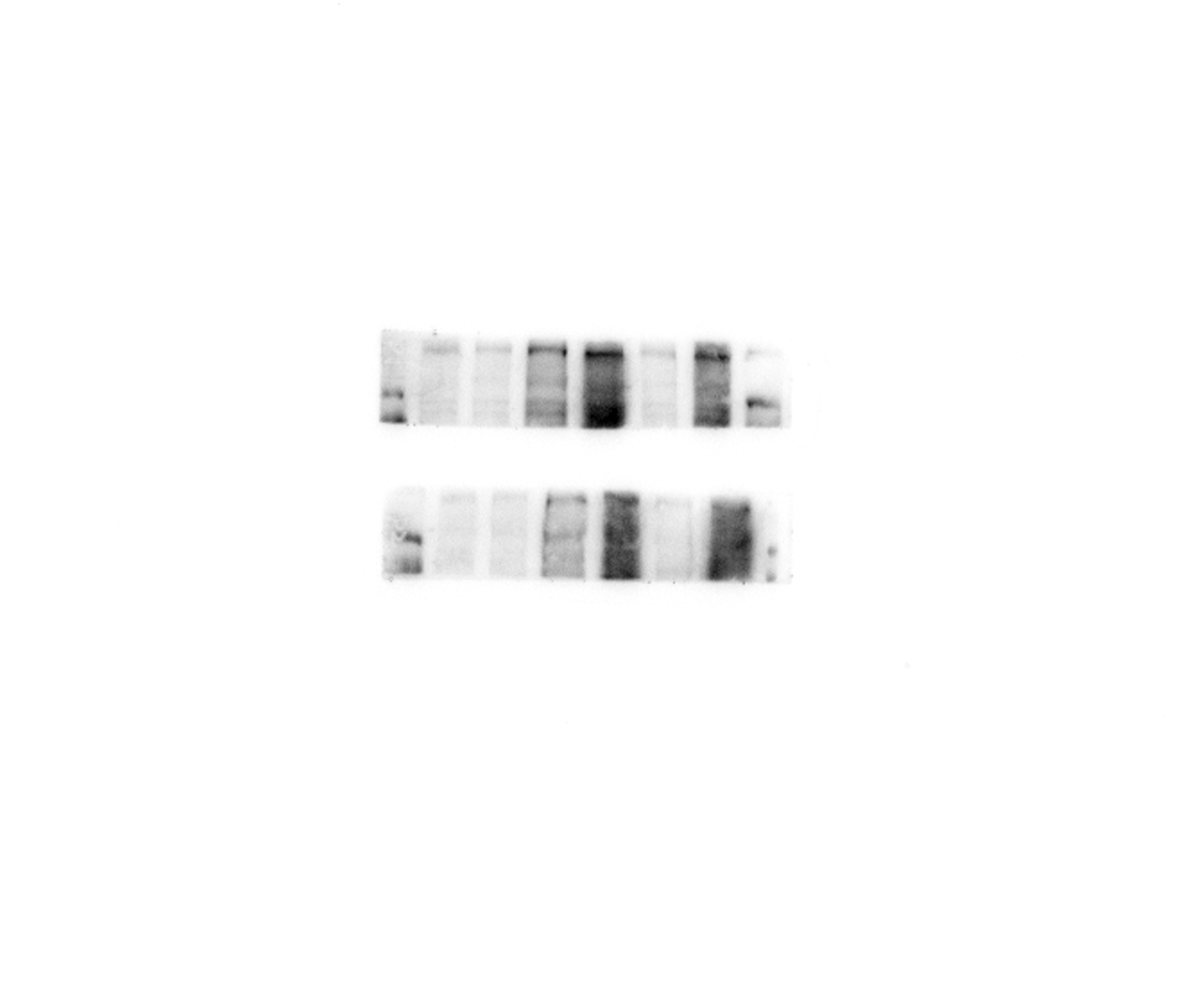

Supplement: Supplementary file 2 [file DataSheet2.ZIP › data1/2022.8.18/p-PERK/9S.Tif]

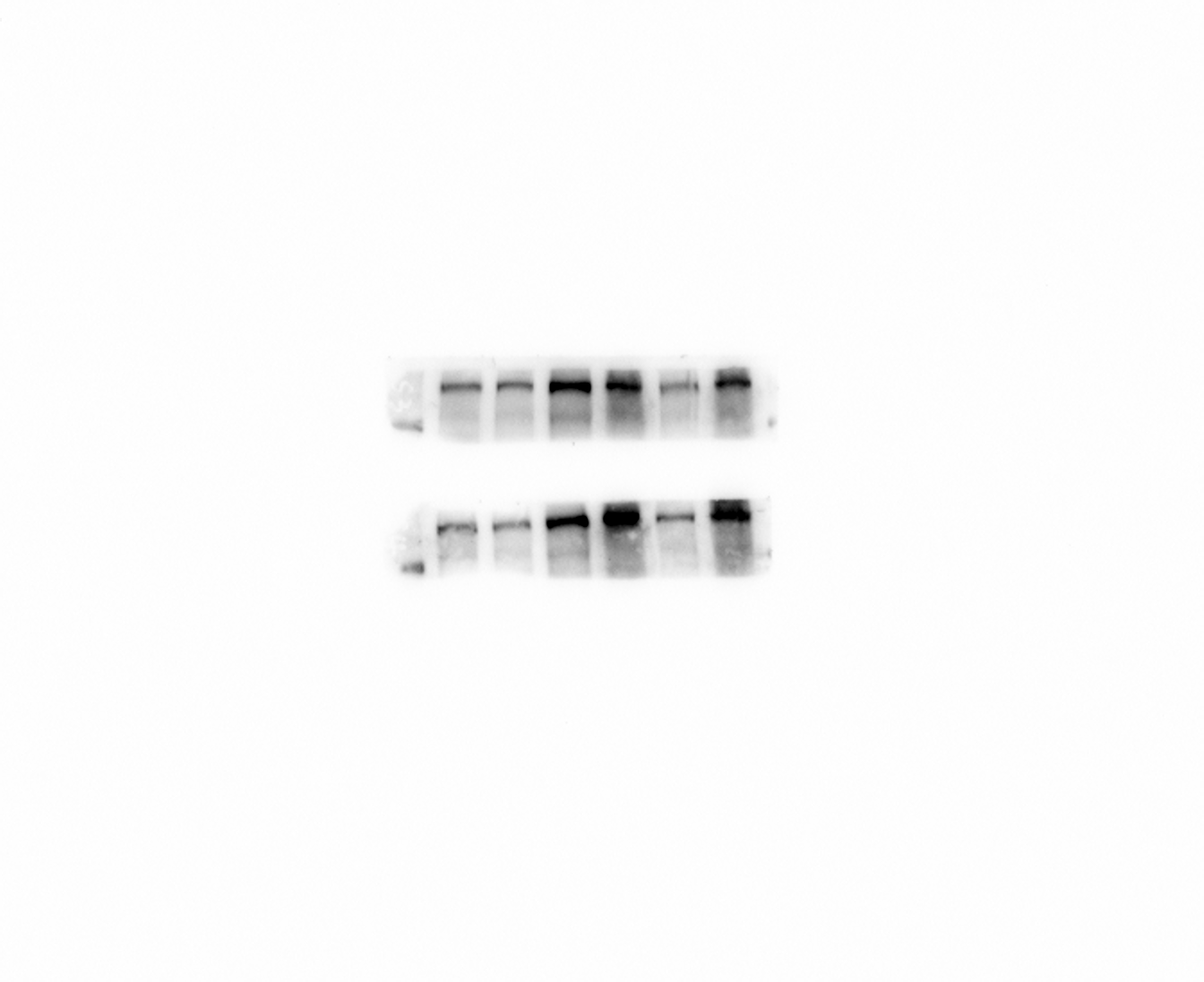

Supplement: Supplementary file 2 [file DataSheet2.ZIP › data1/2022.8.23/chop/10s.Tif]

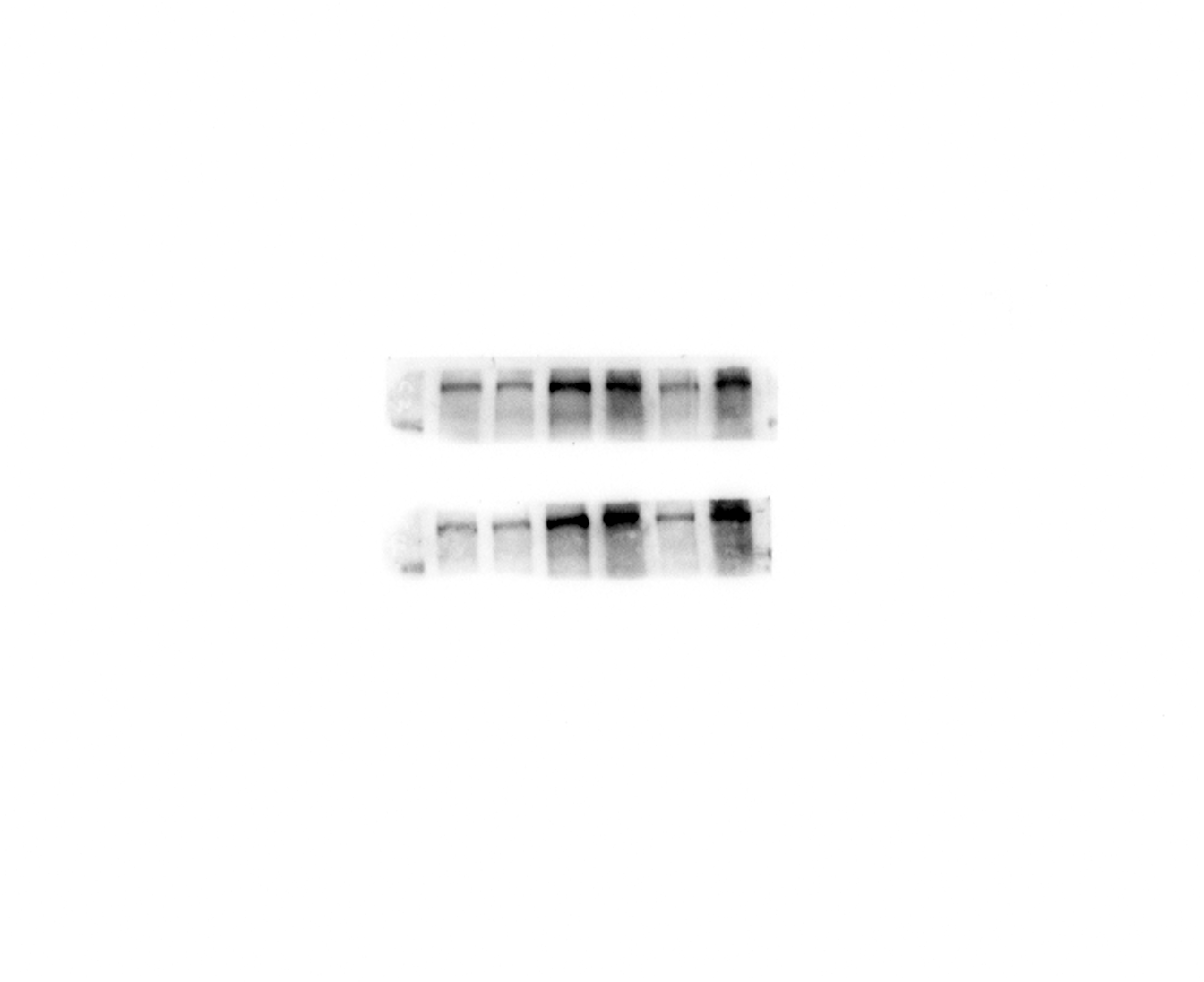

Supplement: Supplementary file 2 [file DataSheet2.ZIP › data1/2022.8.23/chop/5s.Tif]

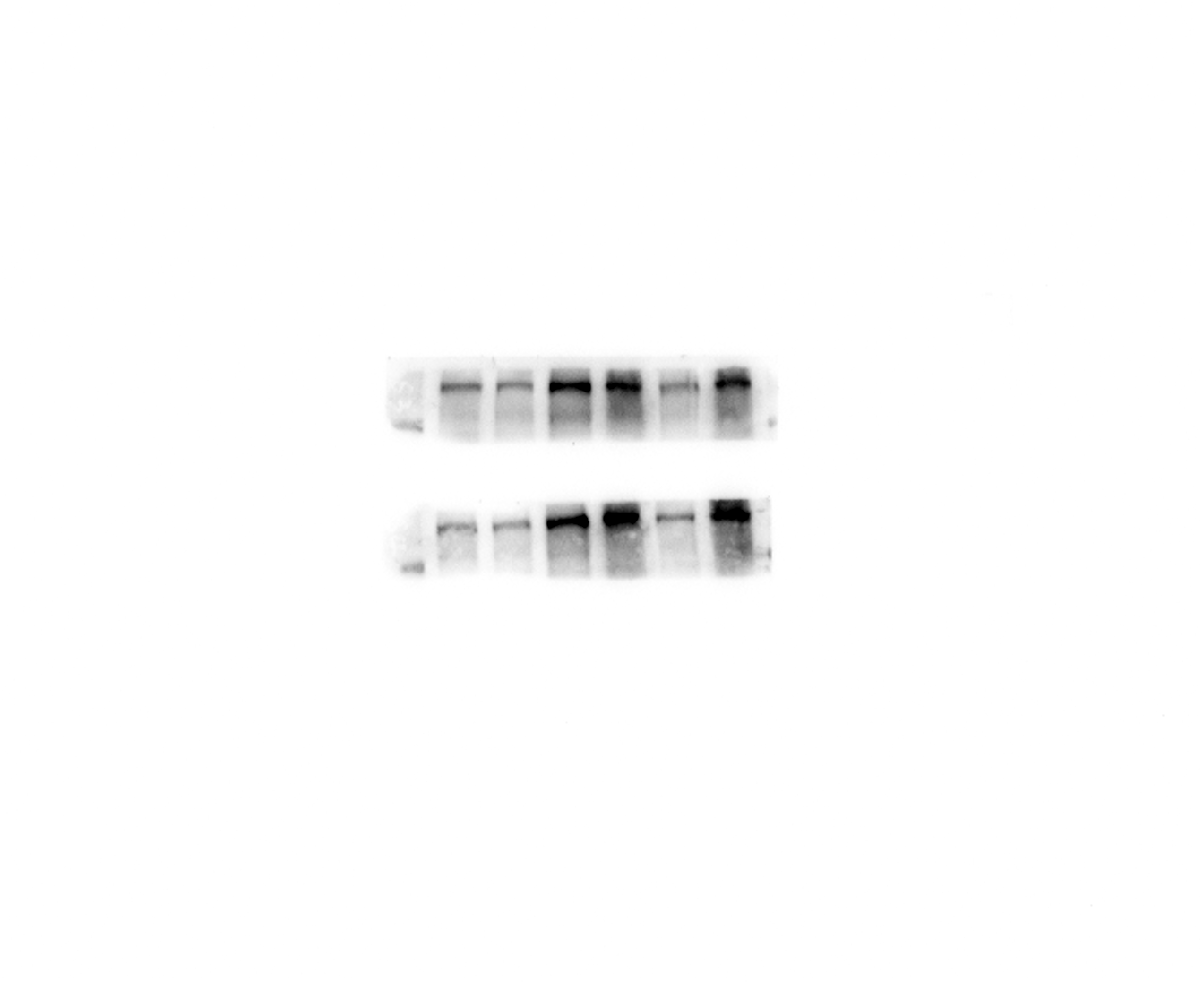

Supplement: Supplementary file 2 [file DataSheet2.ZIP › data1/2022.8.23/chop/7s.Tif]

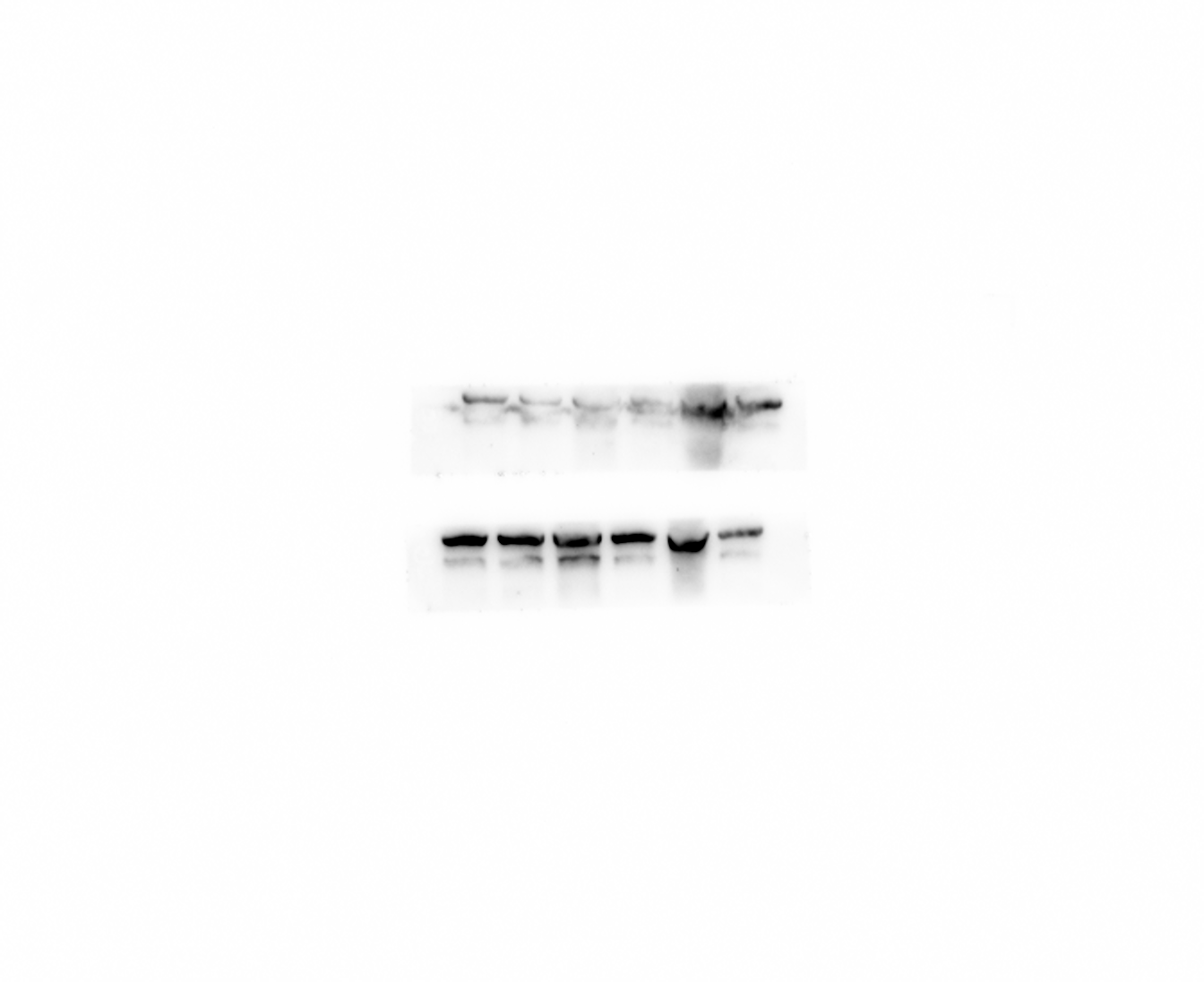

Supplement: Supplementary file 2 [file DataSheet2.ZIP › data1/2022.8.23/eif2a/10s.Tif]

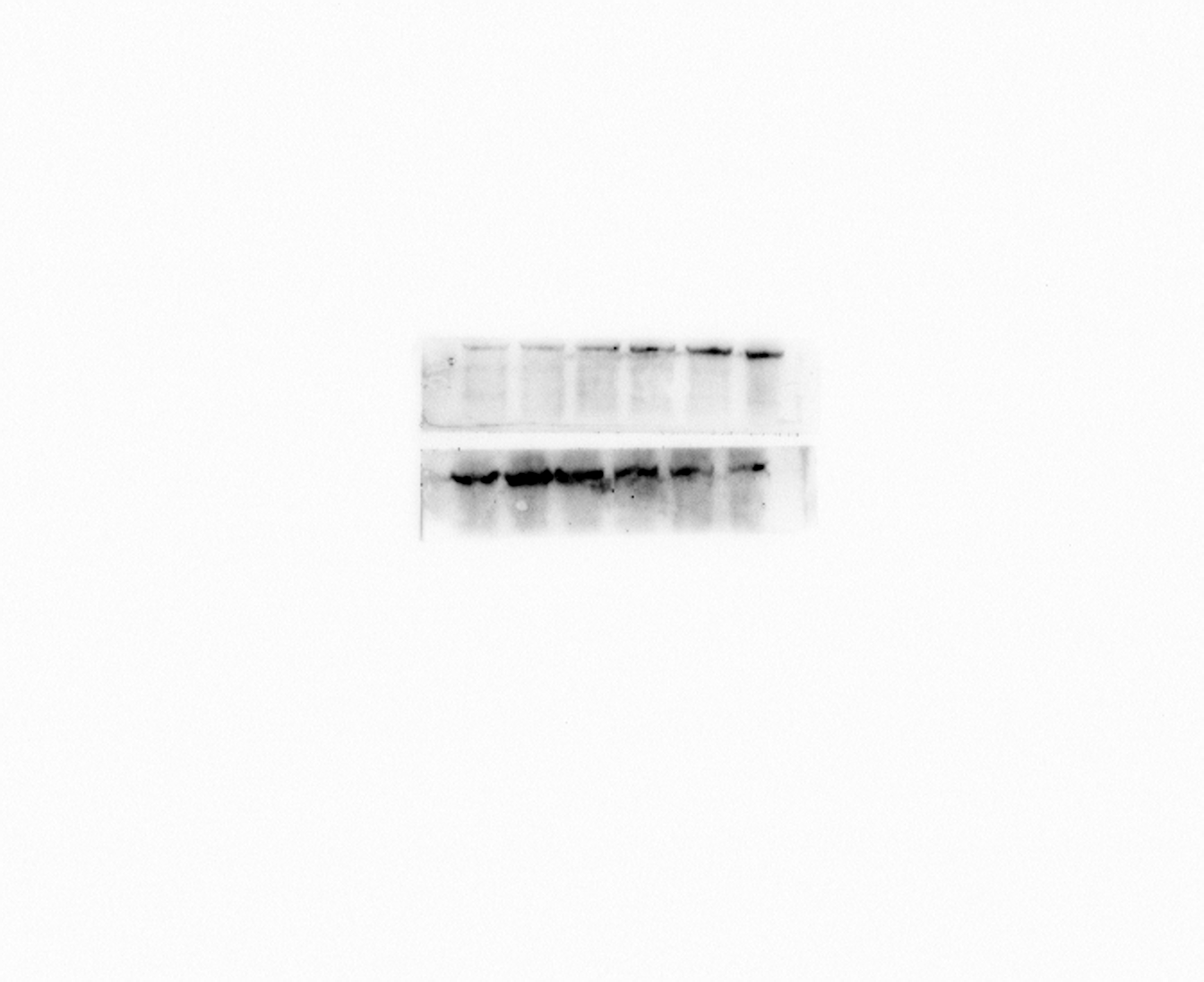

Supplement: Supplementary file 2 [file DataSheet2.ZIP › data1/2022.8.23/GAPDH/10s.Tif]

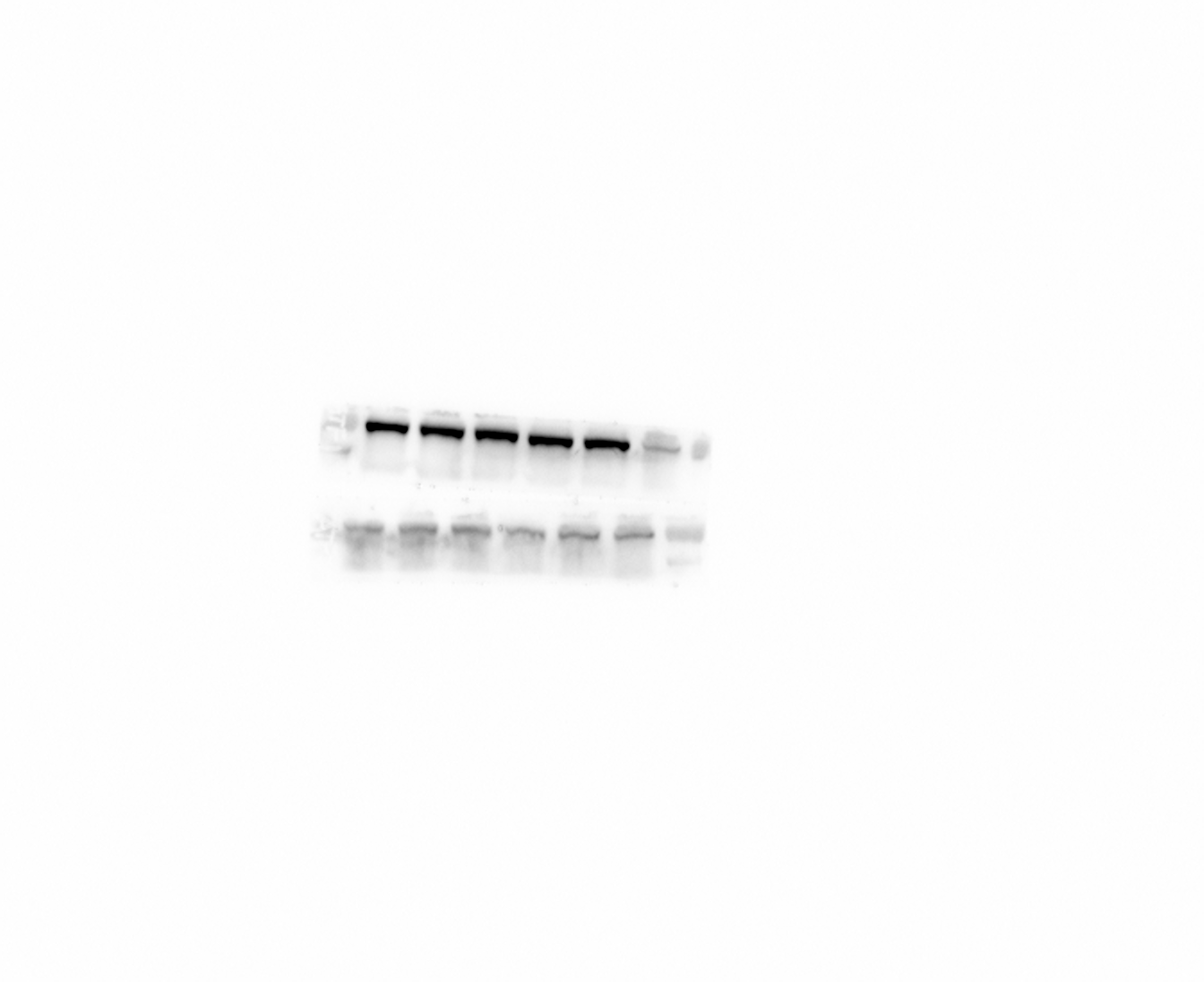

Supplement: Supplementary file 2 [file DataSheet2.ZIP › data1/2022.8.23/GAPDH/5s.Tif]

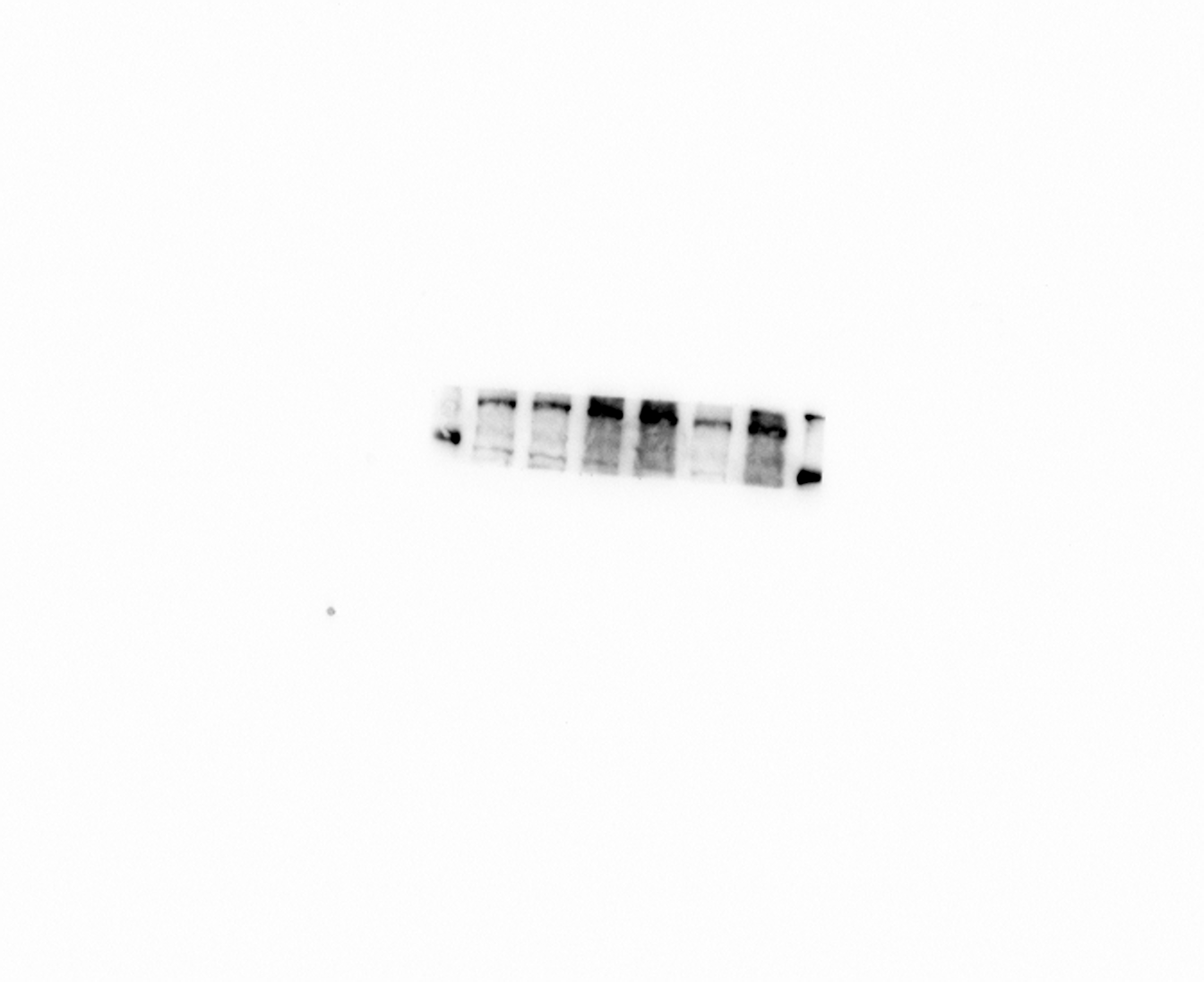

Supplement: Supplementary file 2 [file DataSheet2.ZIP › data1/2022.8.23/grp78/10s.Tif]

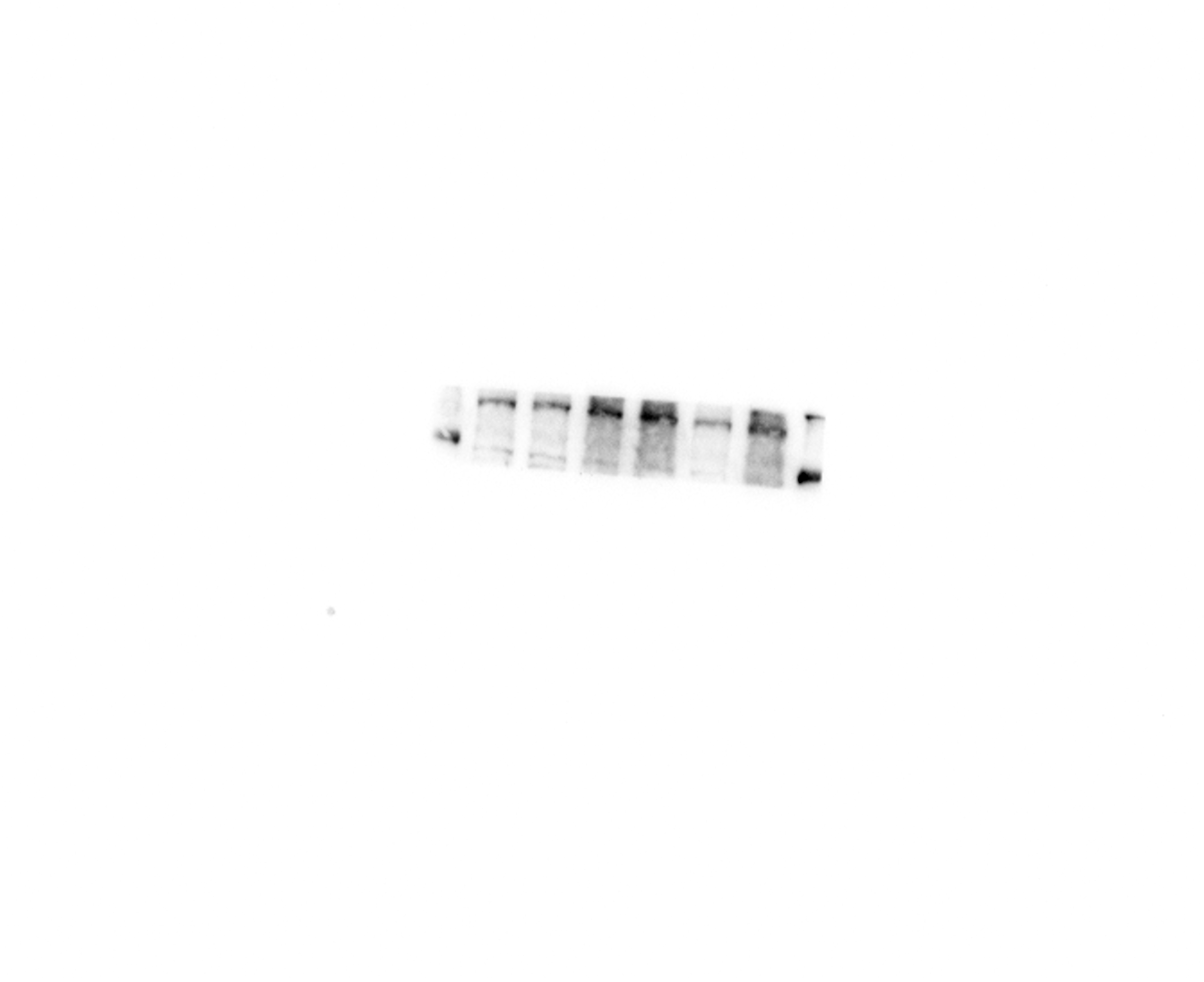

Supplement: Supplementary file 2 [file DataSheet2.ZIP › data1/2022.8.23/grp78/5s.Tif]

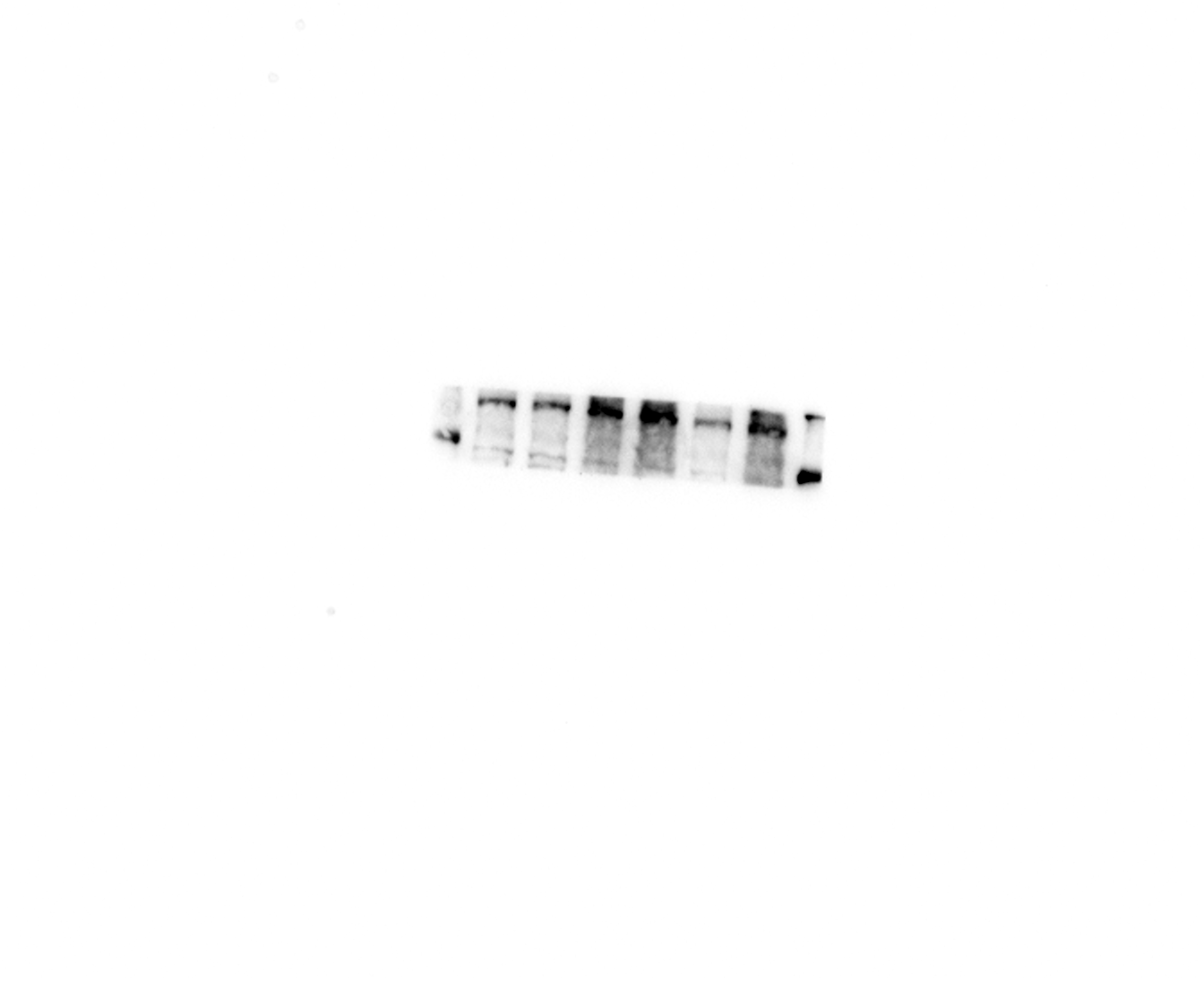

Supplement: Supplementary file 2 [file DataSheet2.ZIP › data1/2022.8.23/grp78/7s.Tif]

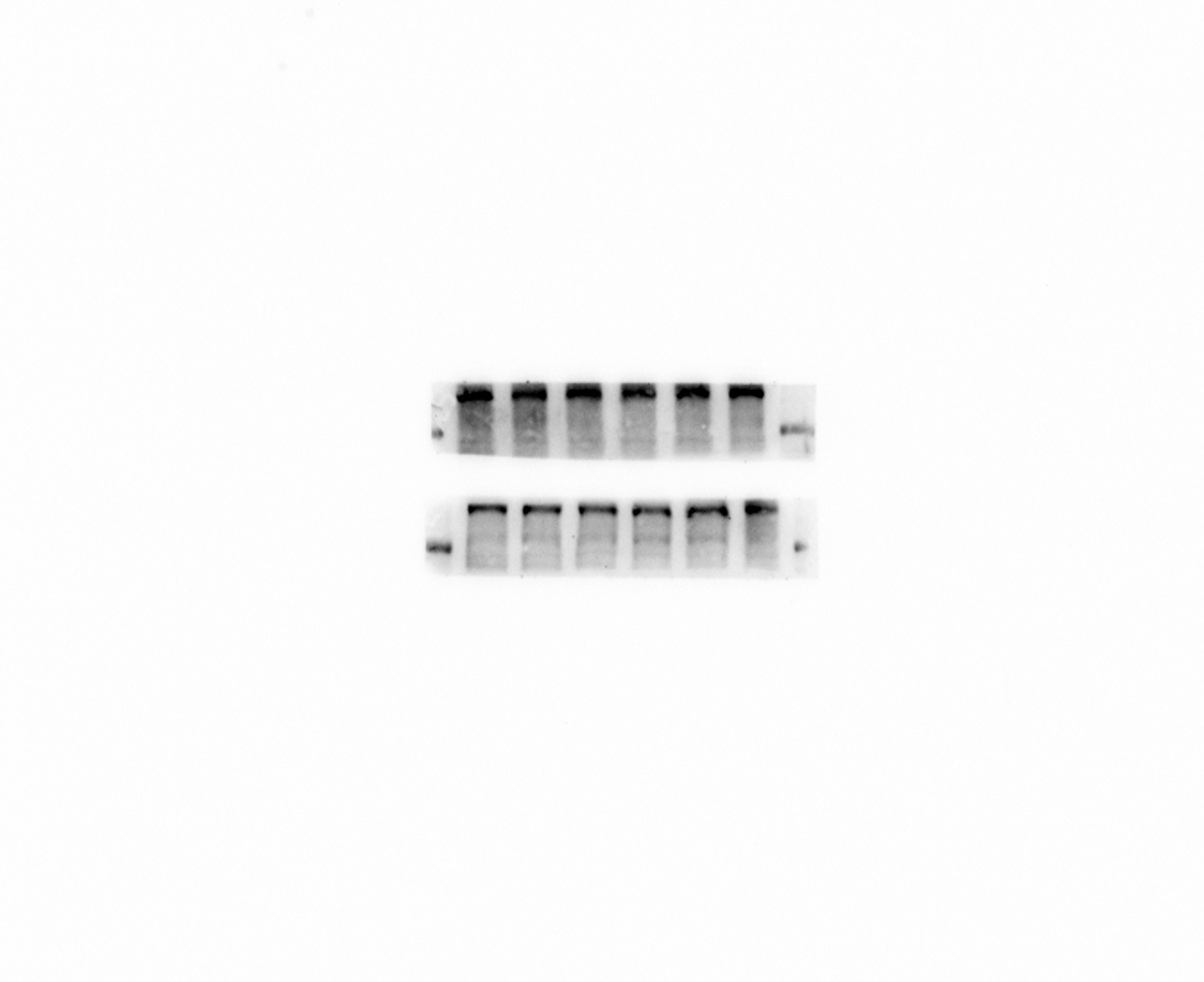

Supplement: Supplementary file 2 [file DataSheet2.ZIP › data1/2022.8.23/perk eif2a/10s.Tif]

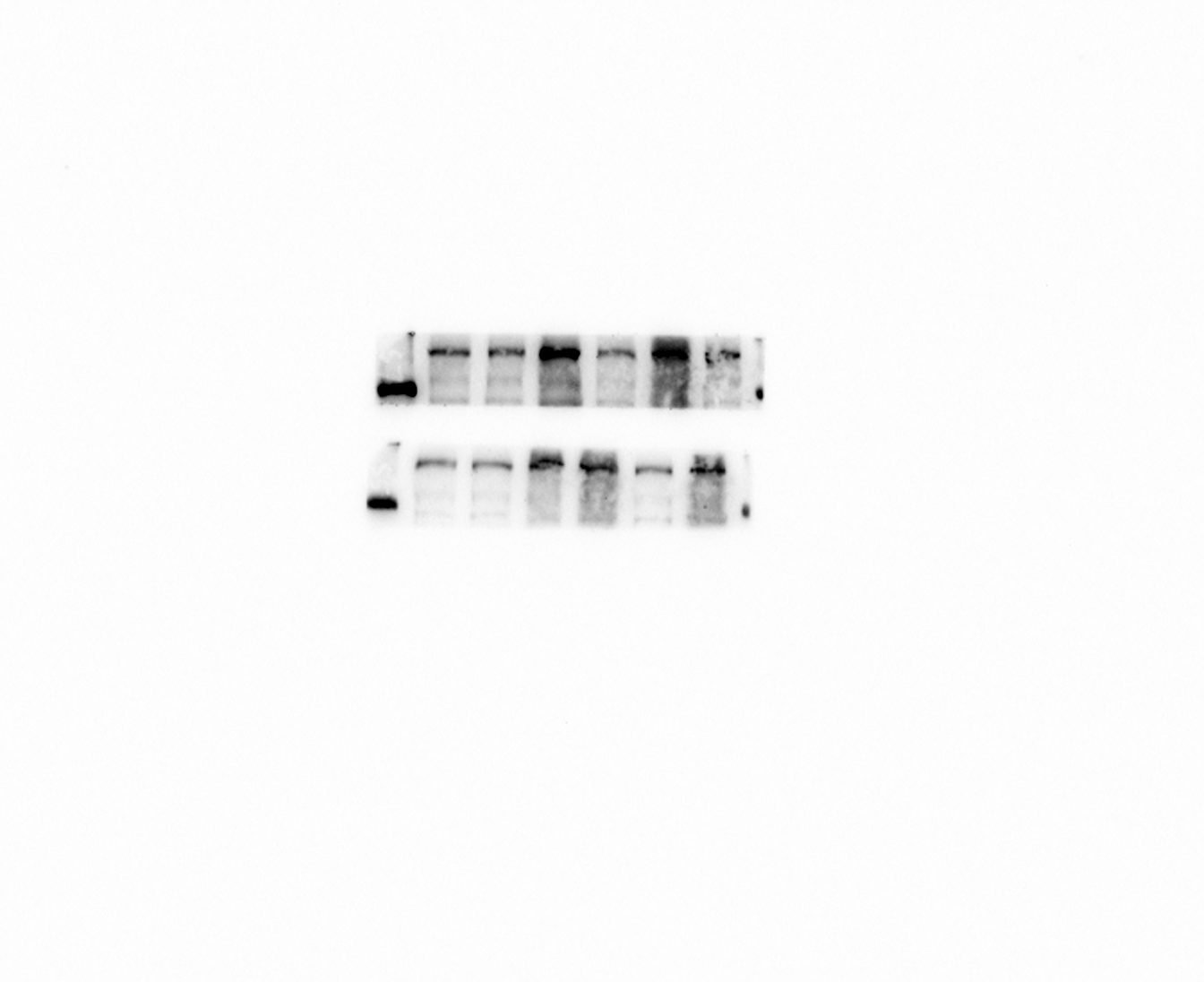

Supplement: Supplementary file 2 [file DataSheet2.ZIP › data1/2022.8.23/sirt1 atf4/10s.Tif]

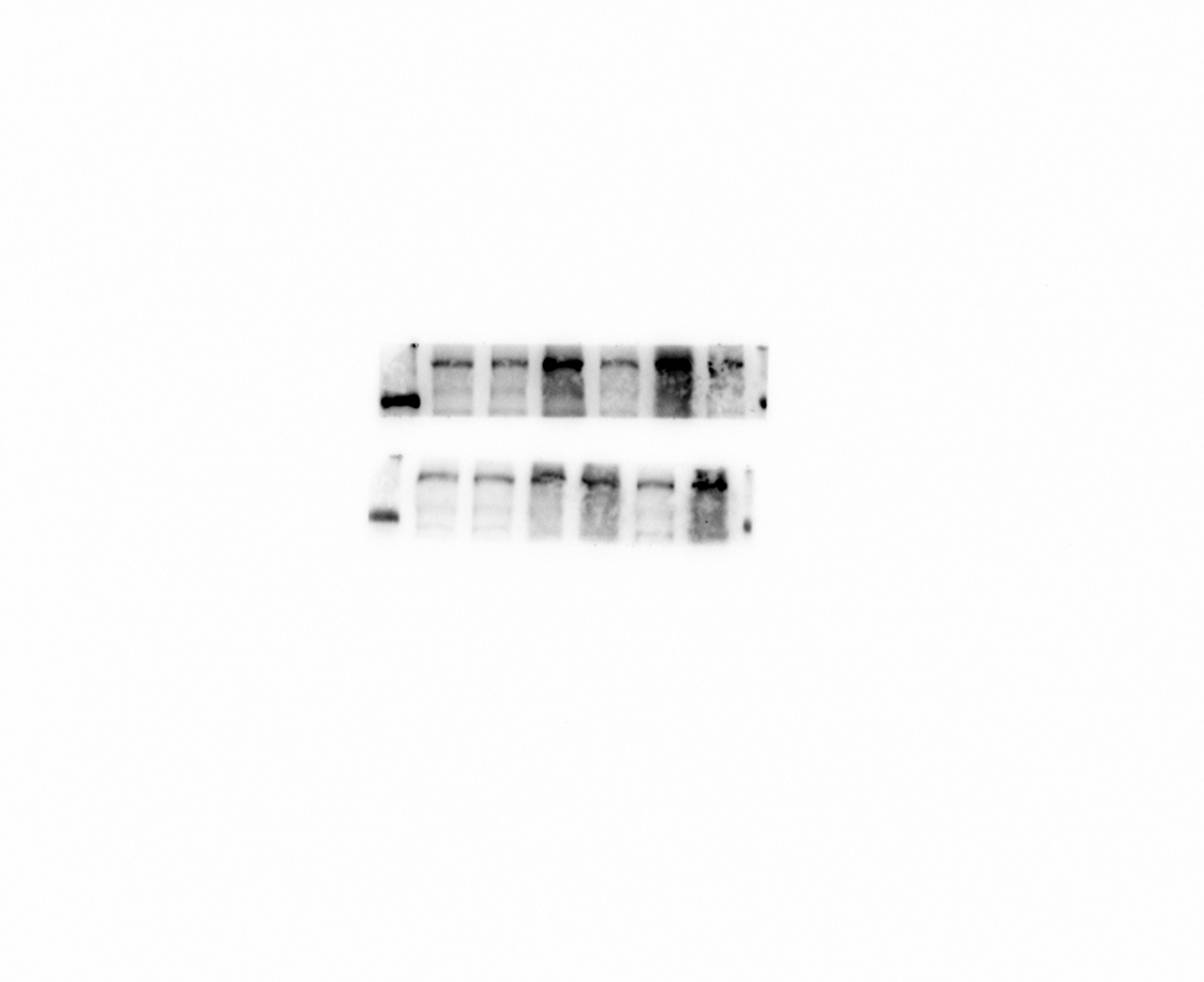

Supplement: Supplementary file 2 [file DataSheet2.ZIP › data1/2022.8.23/sirt1 atf4/12s.Tif]

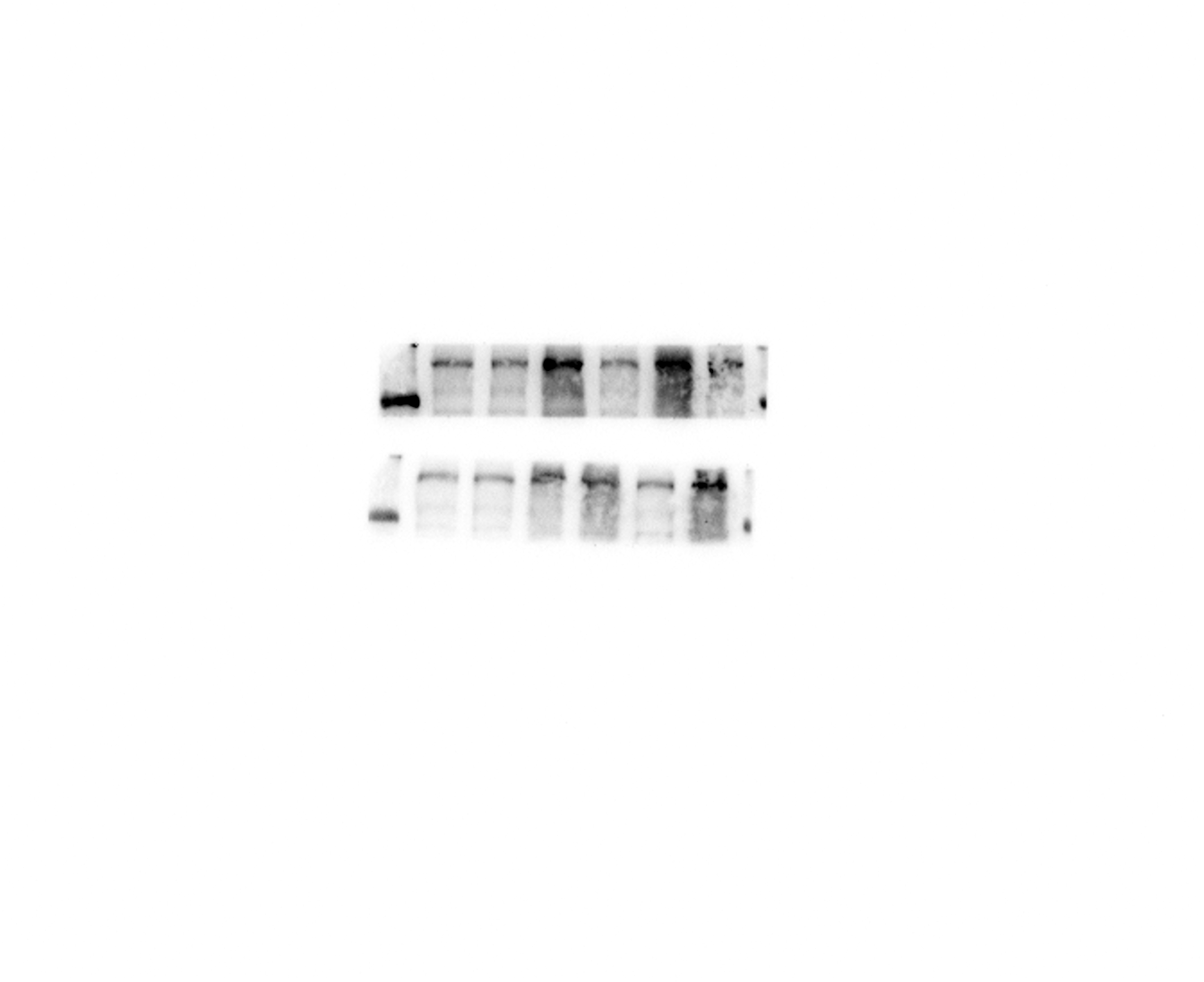

Supplement: Supplementary file 2 [file DataSheet2.ZIP › data1/2022.8.23/sirt1 atf4/5s.Tif]

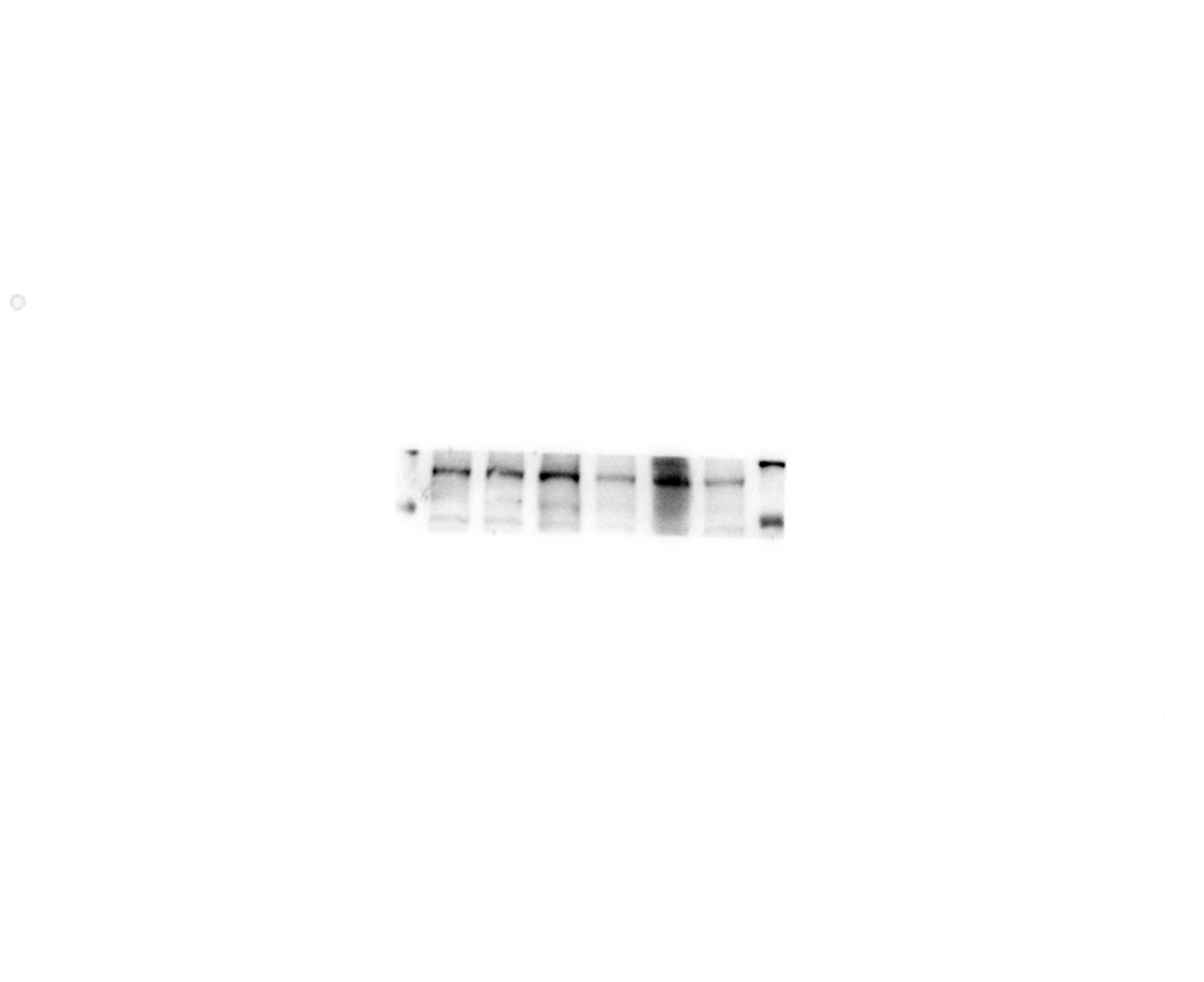

Supplement: Supplementary file 2 [file DataSheet2.ZIP › data1/2022.8.23/sirt1 atf4/sirt1 4s.Tif]
