# Supplementary figures and images for: Polydatin ameliorates early brain injury after subarachnoid hemorrhage through up-regulating SIRT1 to suppress endoplasmic reticulum stress (part 2 of 2)
Source: Front Pharmacol. 2024 Sep 4;15:1450238. doi: 10.3389/fphar.2024.1450238 (PMC11408241; doi:10.3389/fphar.2024.1450238)

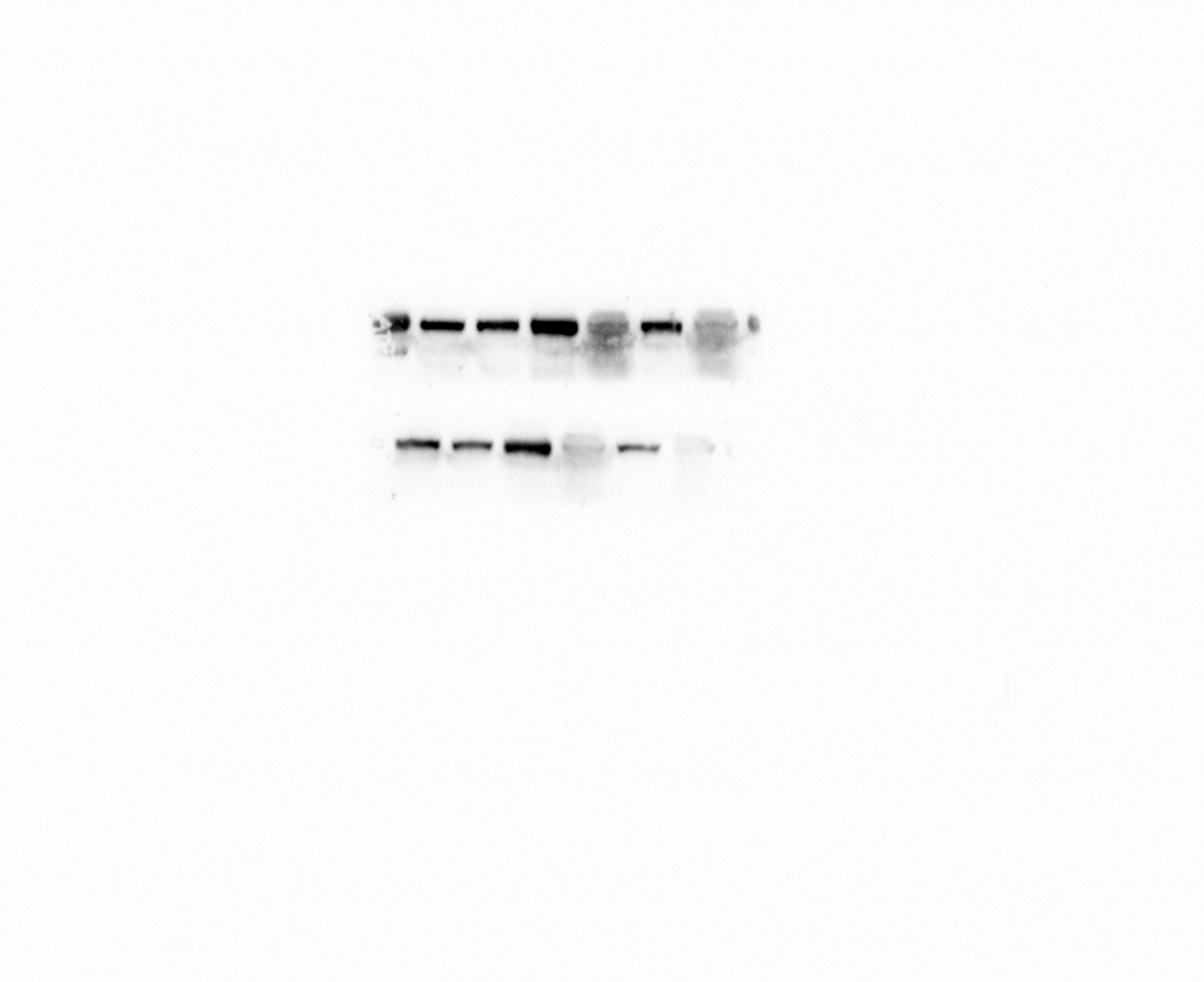

Supplement: Supplementary file 2 [file DataSheet2.ZIP › data1/2022.8.23/sirt1 atf4/sirt1.2.Tif]

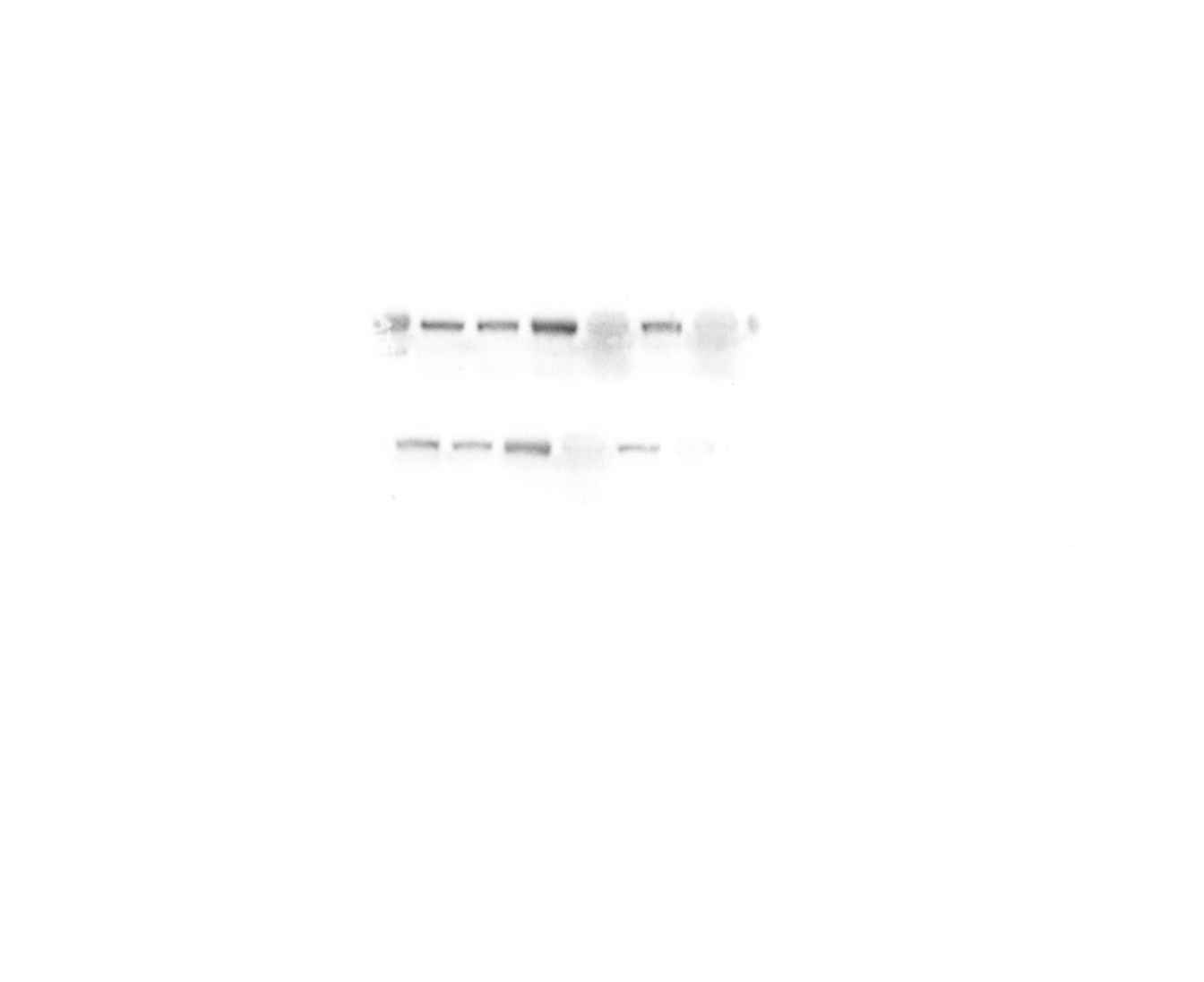

Supplement: Supplementary file 2 [file DataSheet2.ZIP › data1/2022.8.23/sirt1 atf4/sirt1.23s.Tif]

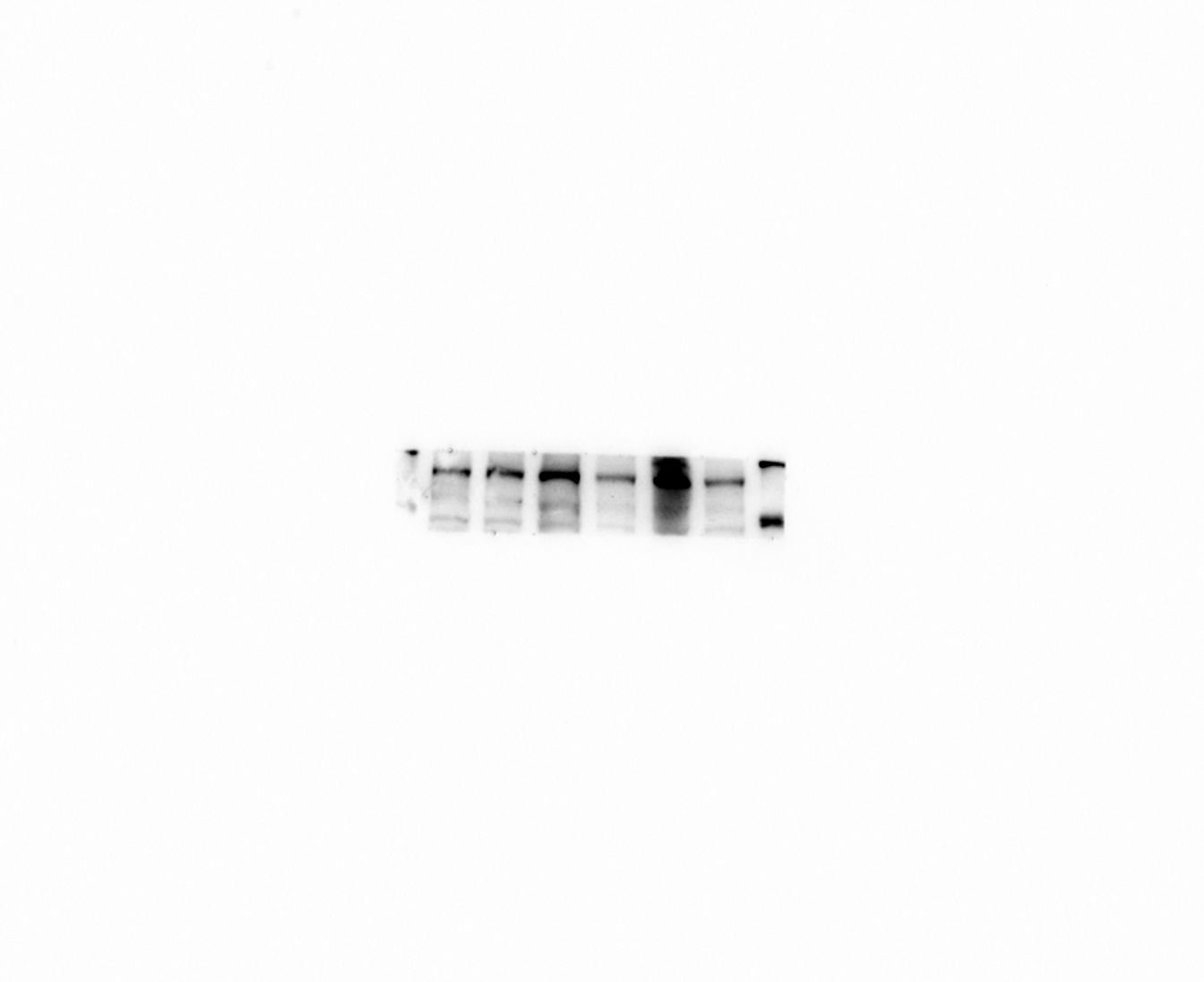

Supplement: Supplementary file 2 [file DataSheet2.ZIP › data1/2022.8.23/sirt1 atf4/sirt1.Tif]

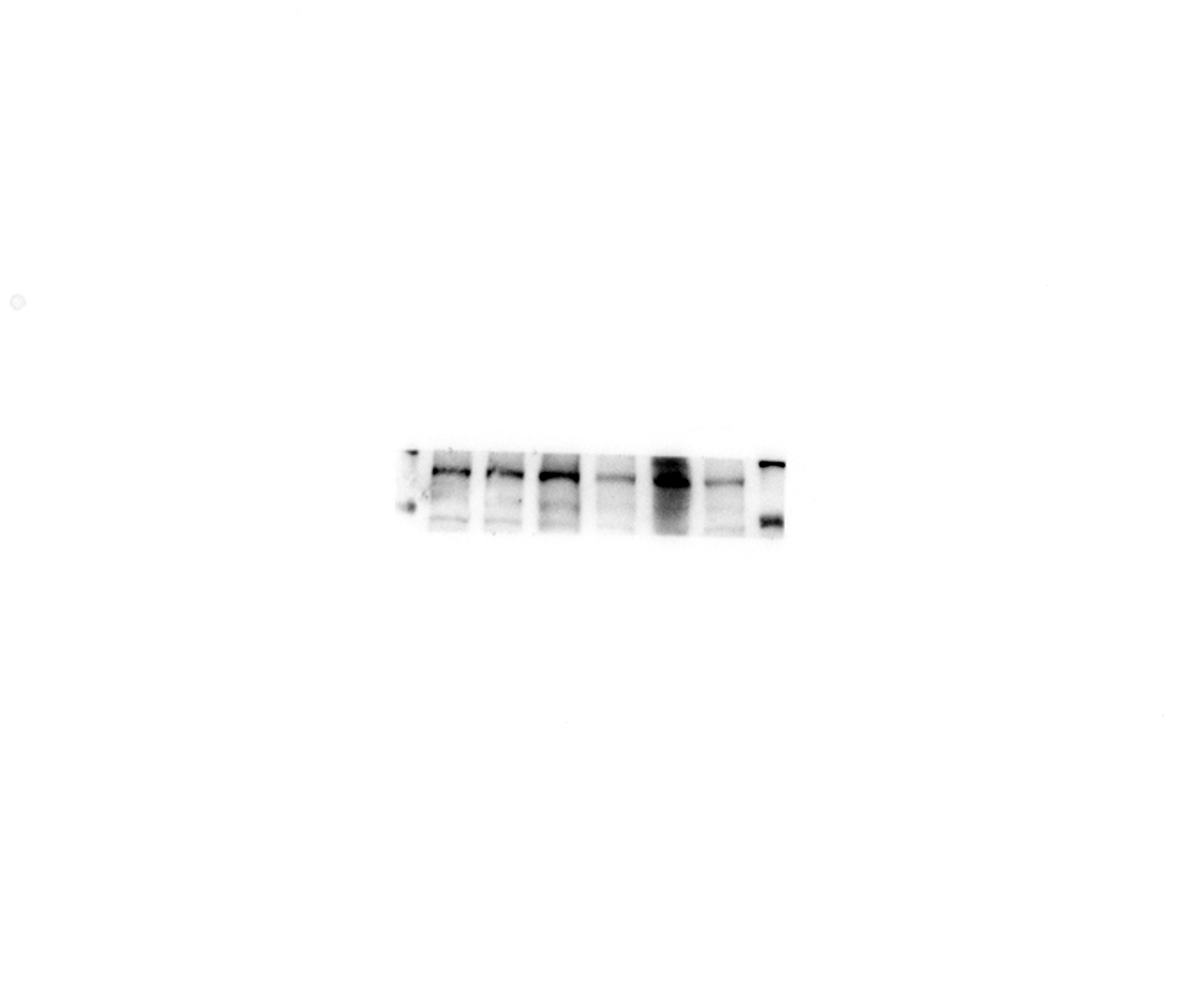

Supplement: Supplementary file 2 [file DataSheet2.ZIP › data1/2022.8.23/sirt1 atf4/sirt15s.Tif]

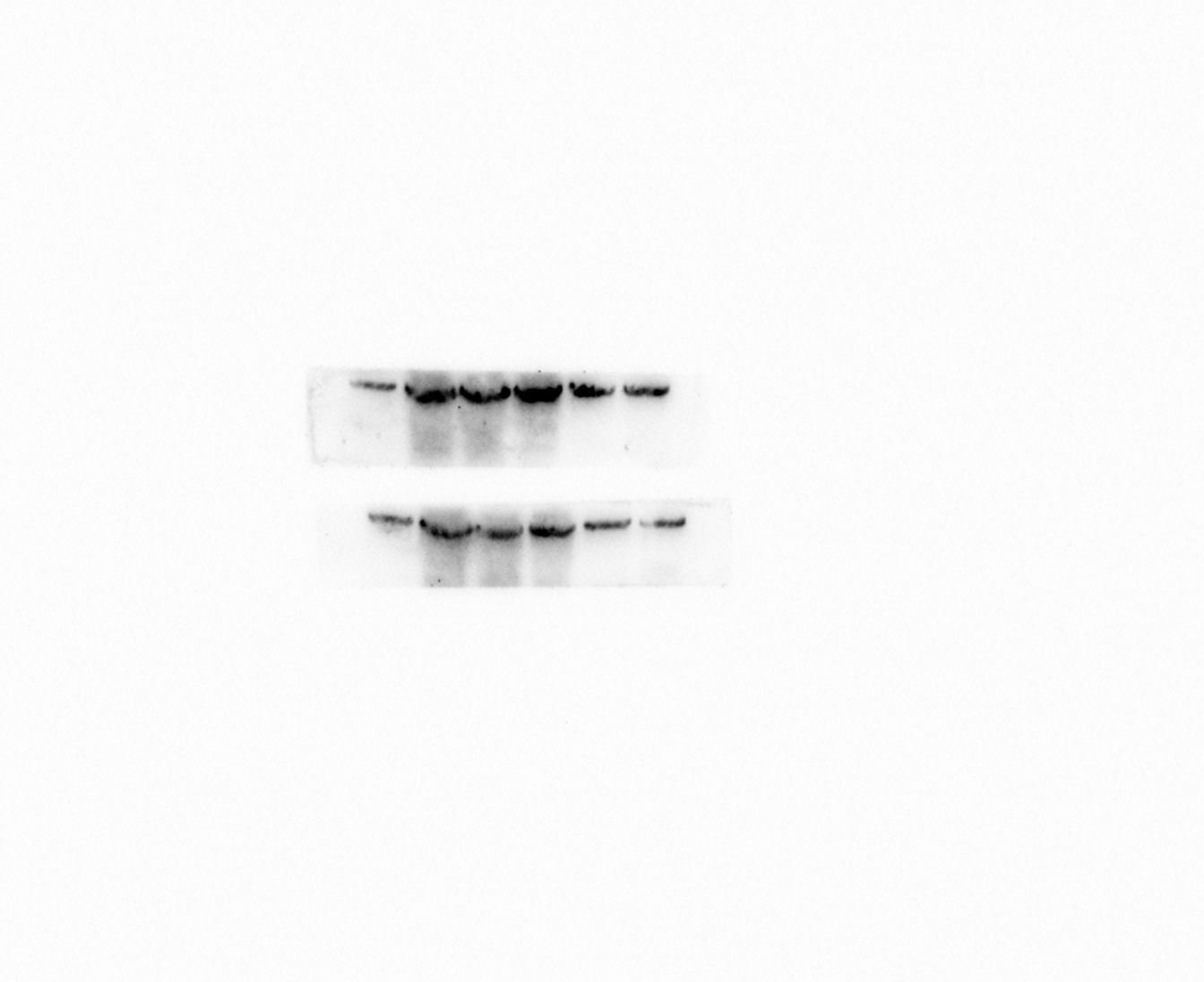

Supplement: Supplementary file 2 [file DataSheet2.ZIP › data1/2022.8.25/ATF4/10S.Tif]

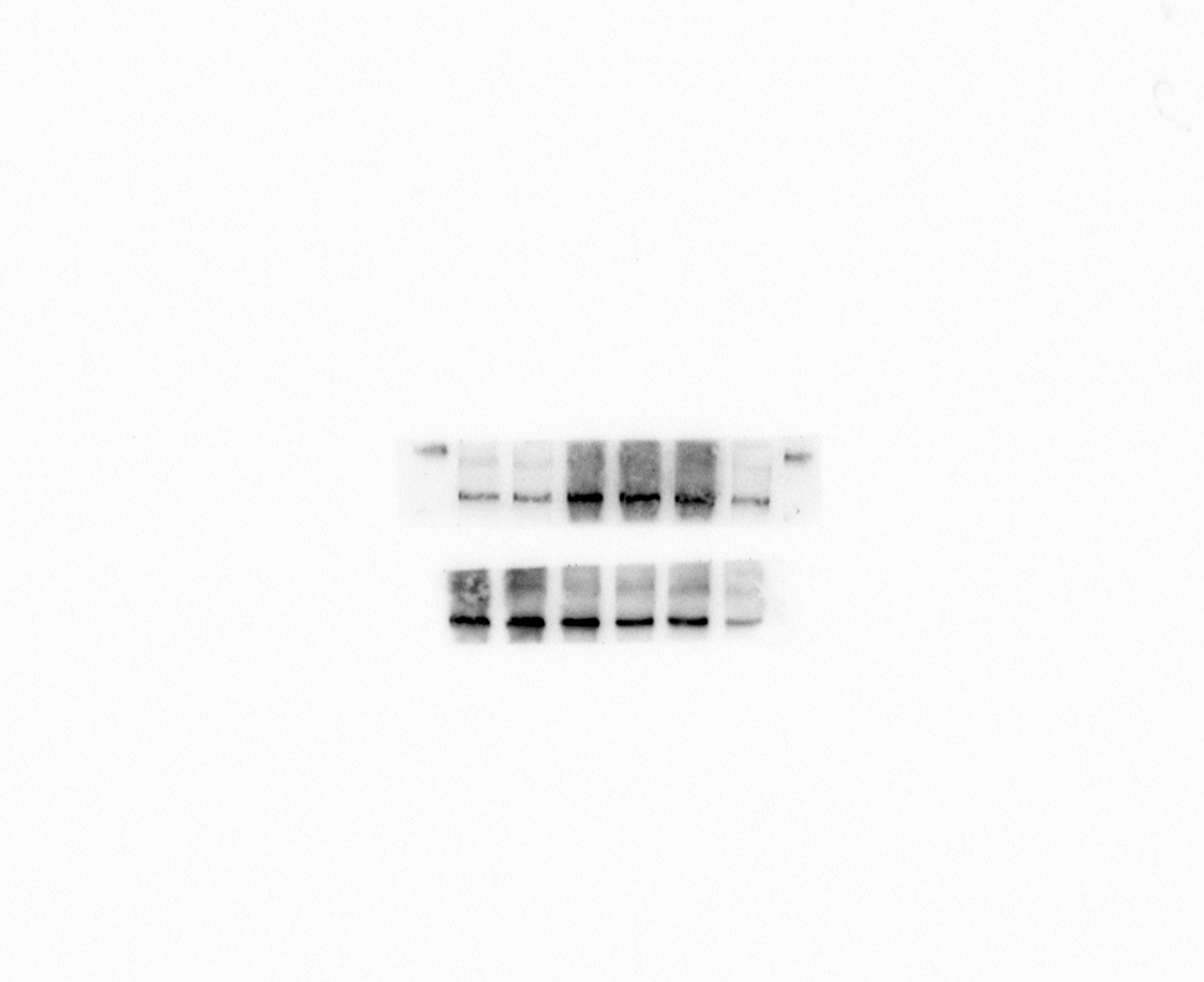

Supplement: Supplementary file 2 [file DataSheet2.ZIP › data1/2022.8.25/CHOP/10S.Tif]

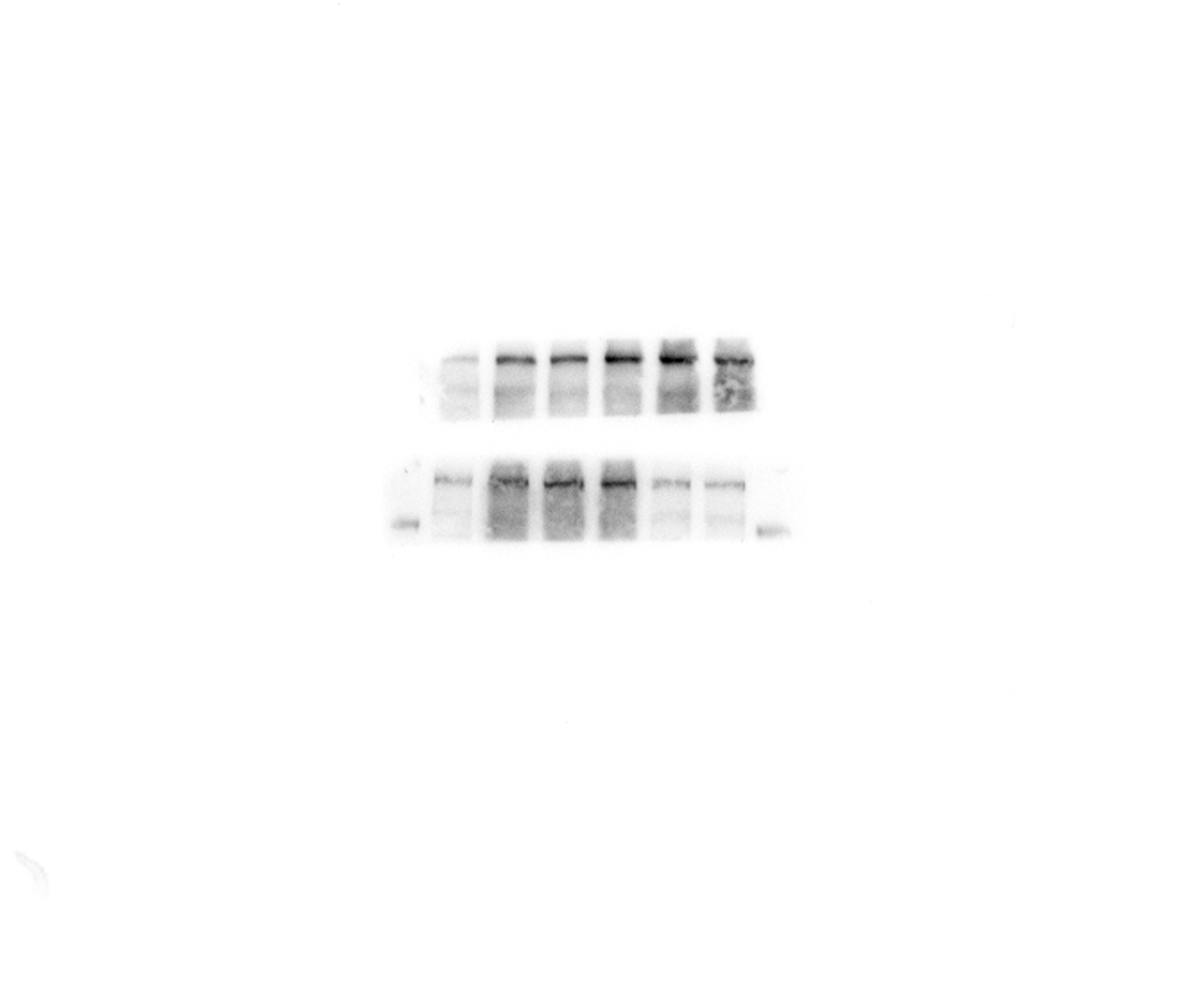

Supplement: Supplementary file 2 [file DataSheet2.ZIP › data1/2022.8.25/CHOP/7S.Tif]

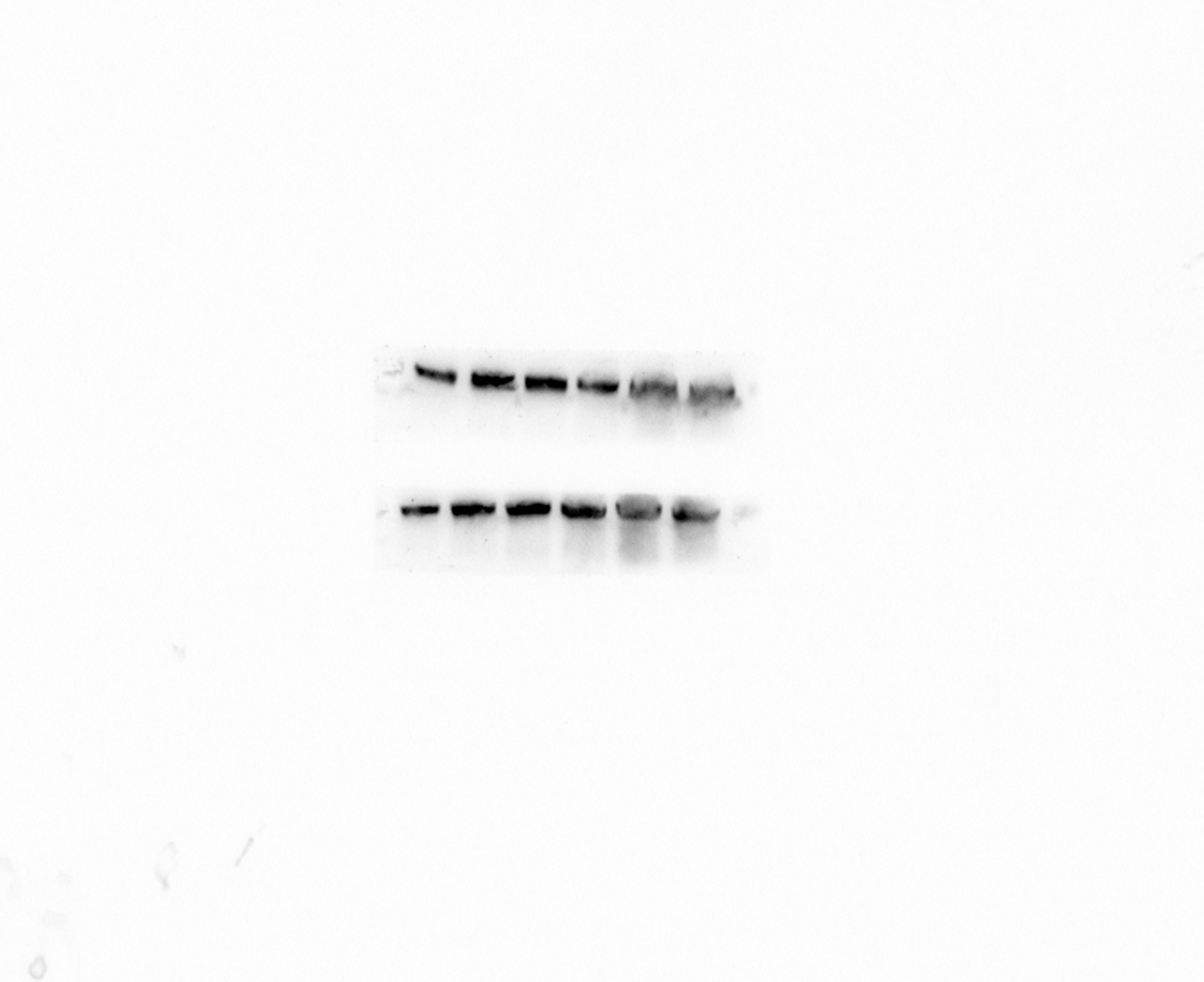

Supplement: Supplementary file 2 [file DataSheet2.ZIP › data1/2022.8.25/GAPDH/10S.Tif]

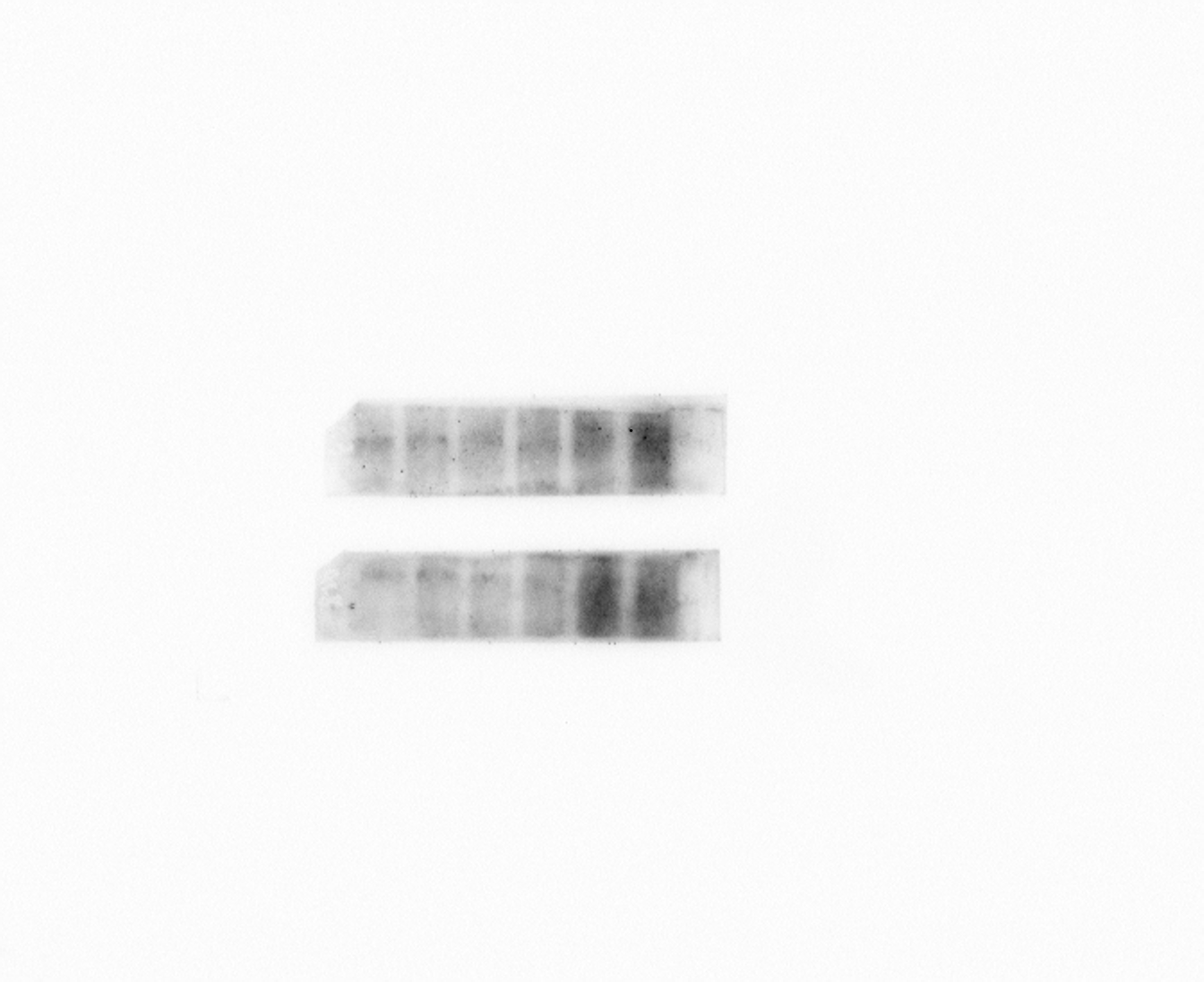

Supplement: Supplementary file 2 [file DataSheet2.ZIP › data1/2022.8.25/P-EIF2A/10S.Tif]

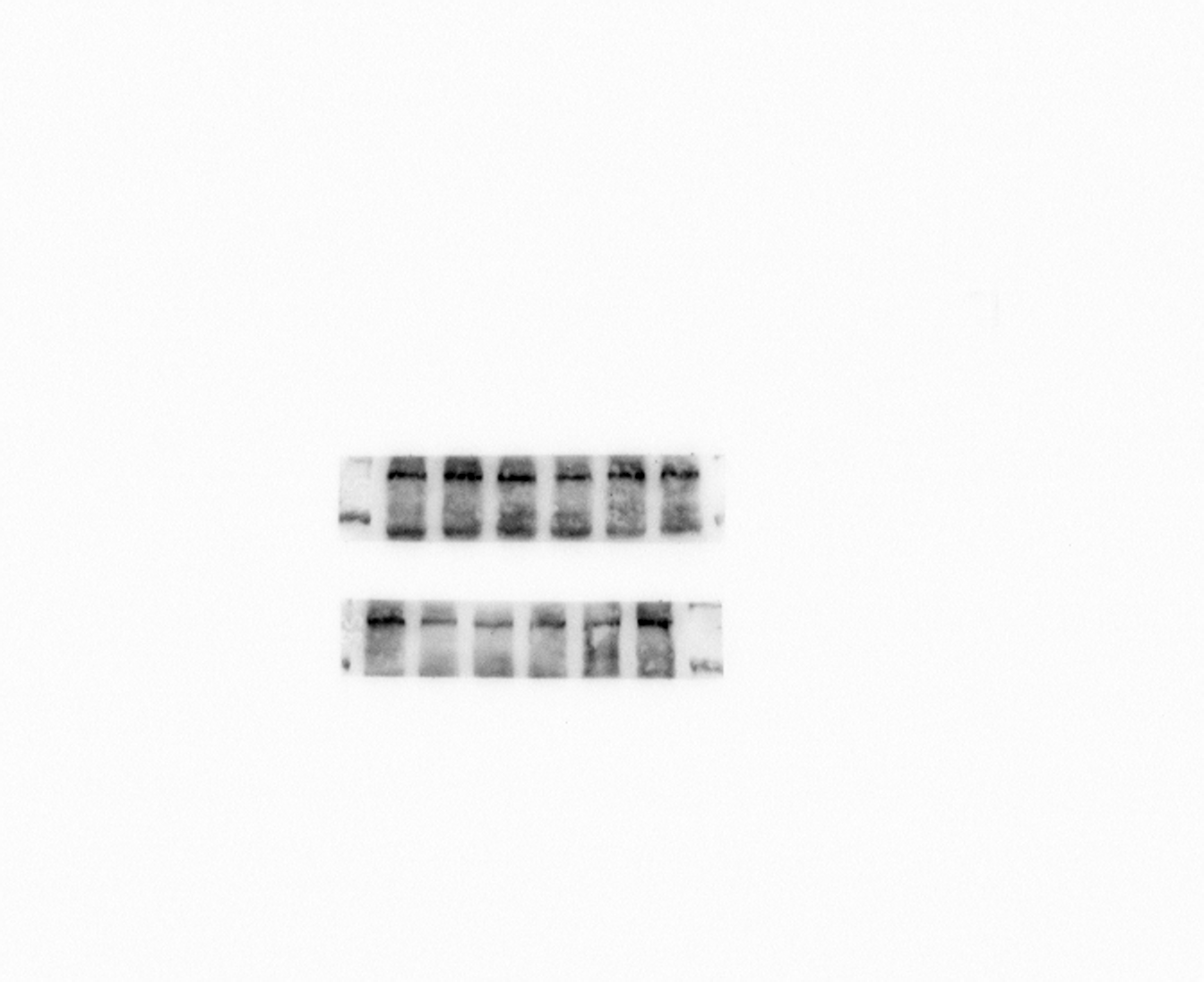

Supplement: Supplementary file 2 [file DataSheet2.ZIP › data1/2022.8.25/PERK/10S.Tif]

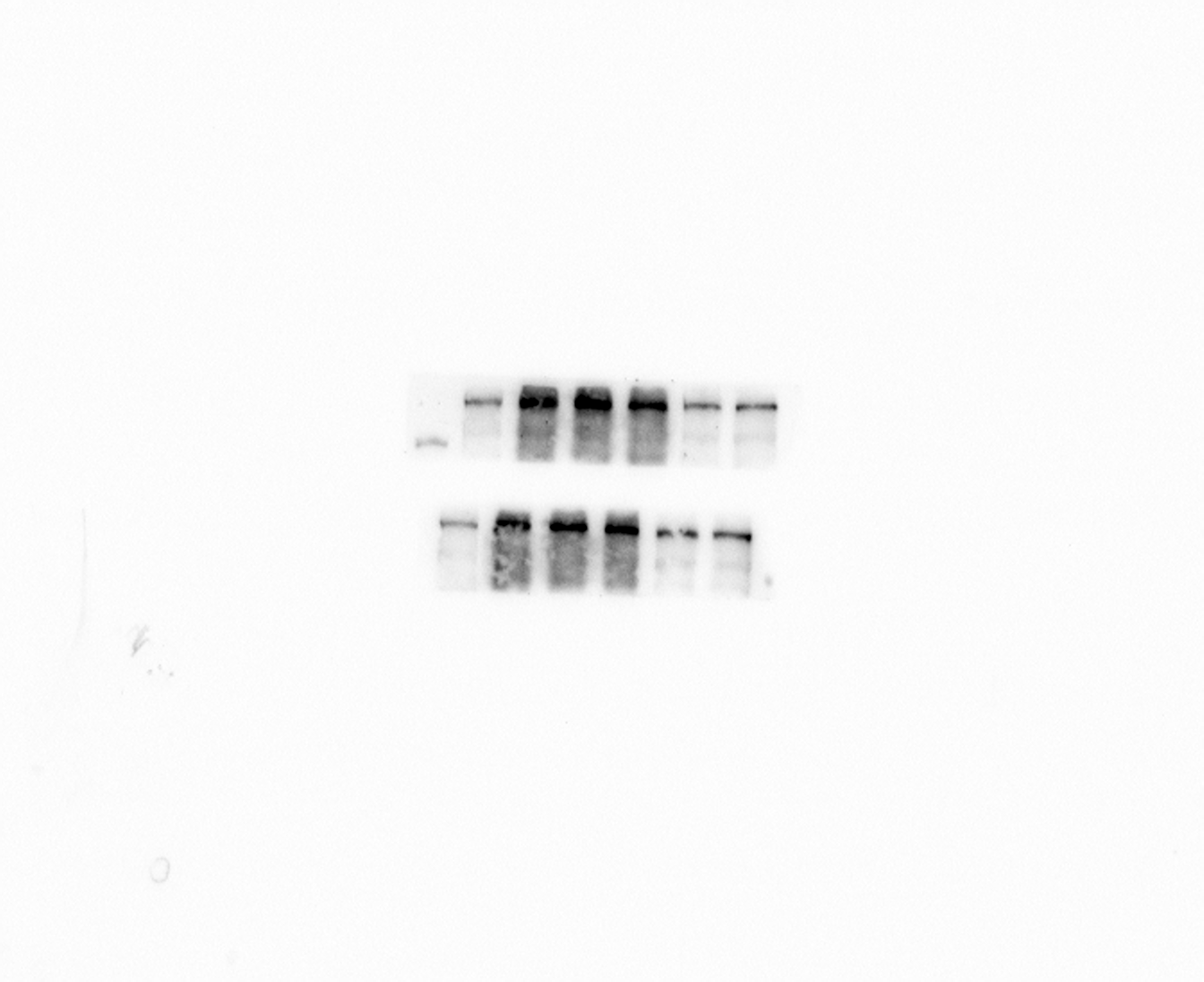

Supplement: Supplementary file 2 [file DataSheet2.ZIP › data1/2022.8.25/P-PERK/10S.Tif]

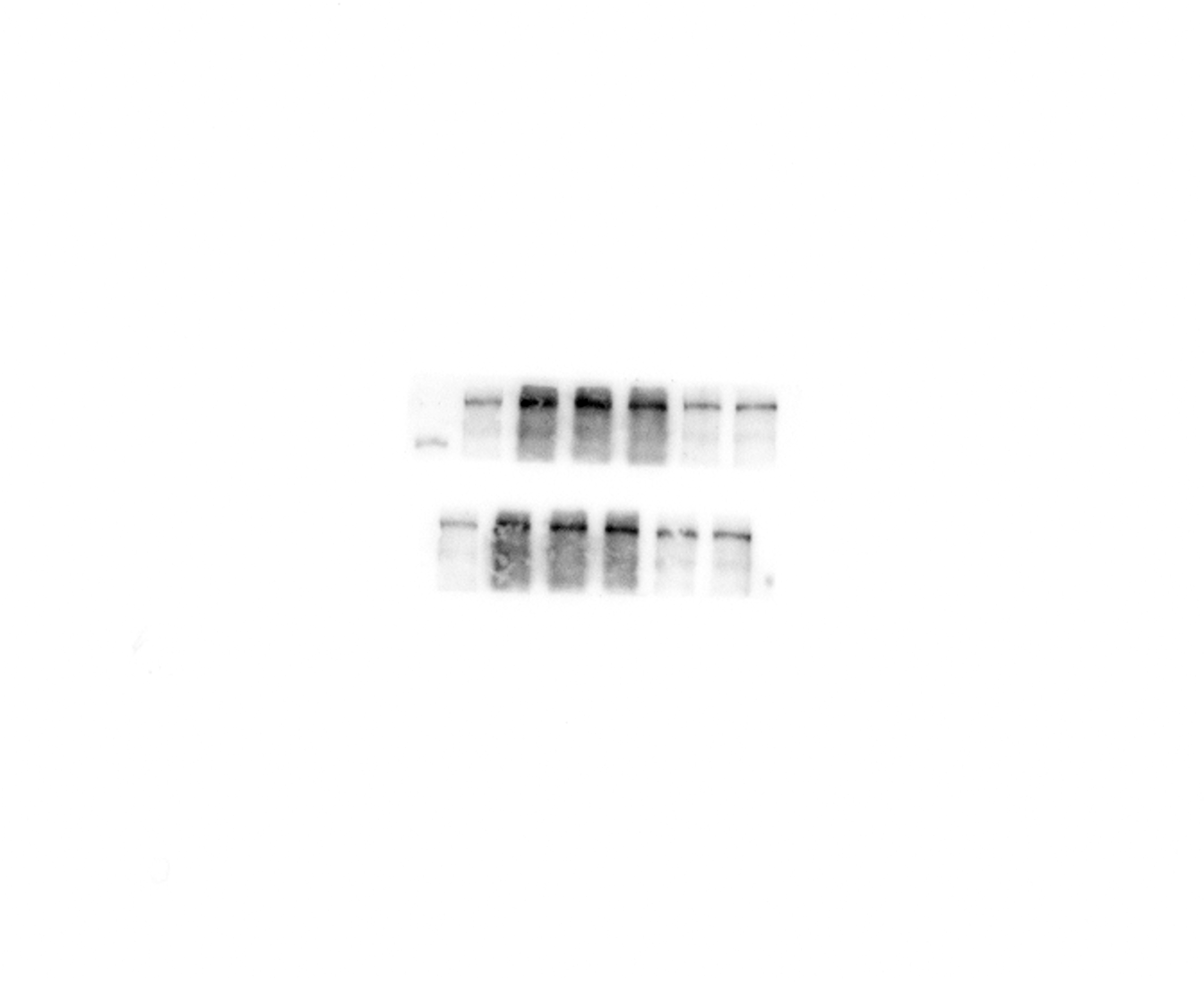

Supplement: Supplementary file 2 [file DataSheet2.ZIP › data1/2022.8.25/P-PERK/7S.Tif]

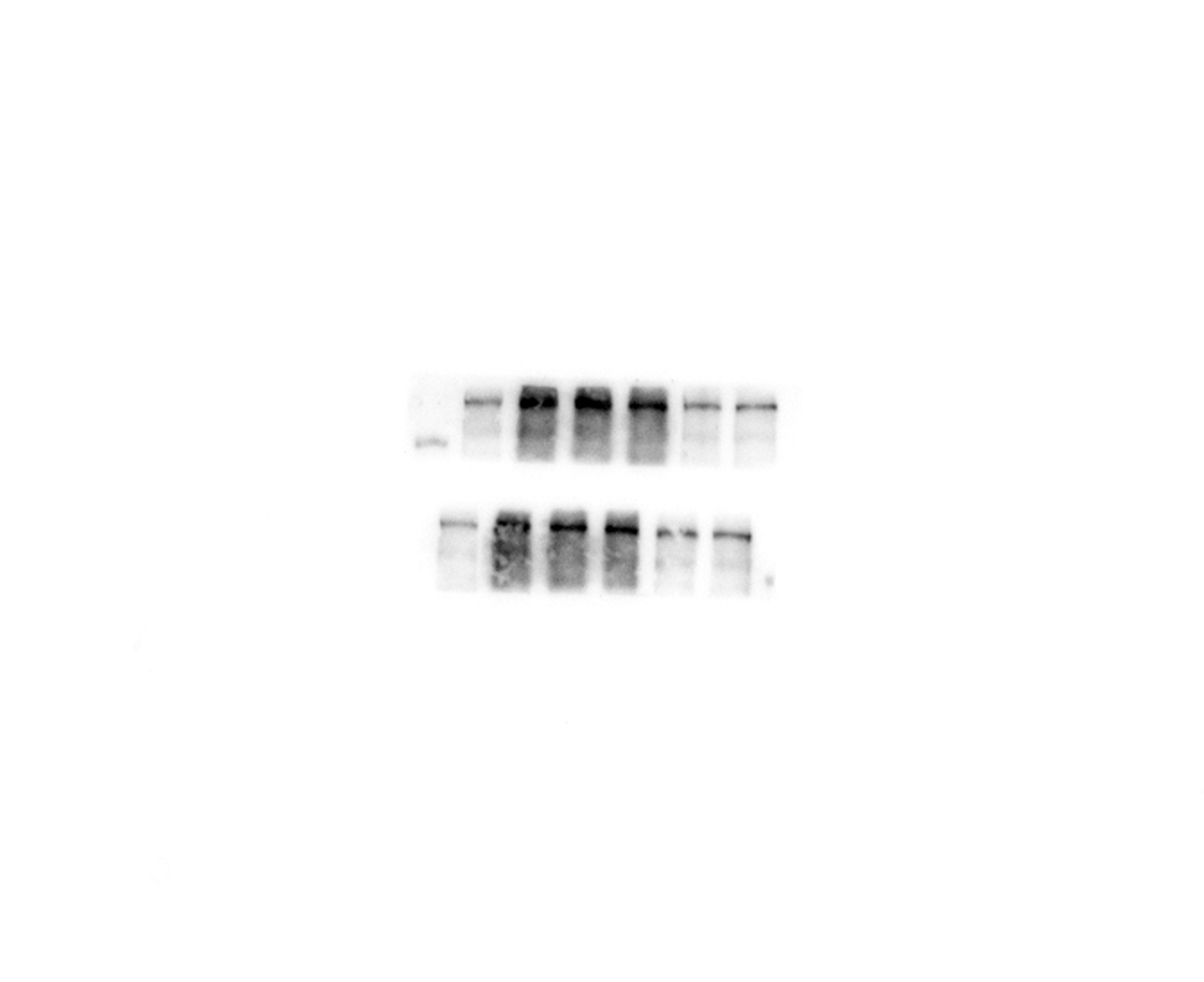

Supplement: Supplementary file 2 [file DataSheet2.ZIP › data1/2022.8.25/P-PERK/8S.Tif]

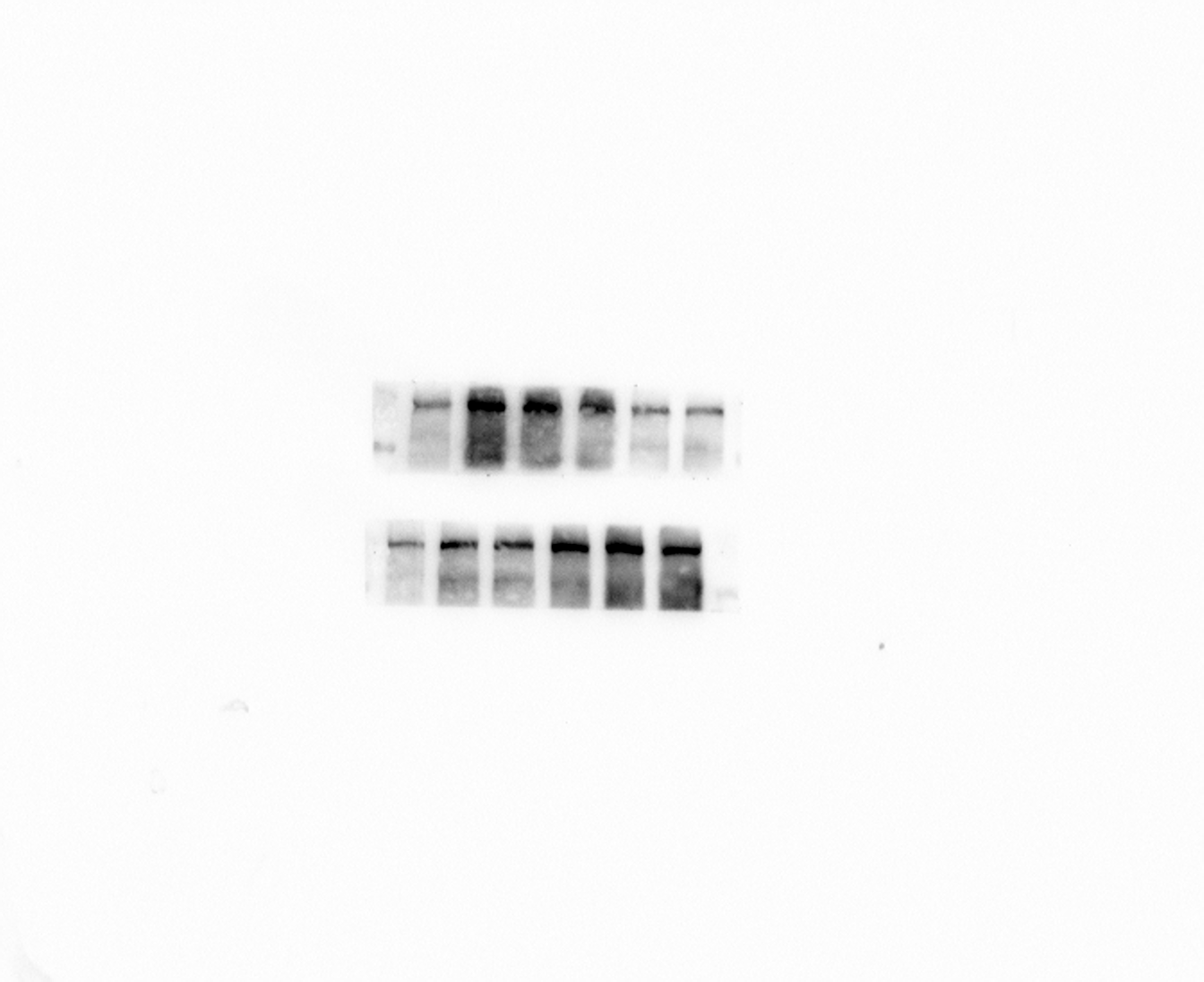

Supplement: Supplementary file 2 [file DataSheet2.ZIP › data1/2022.8.25/SIRT1/10S.Tif]

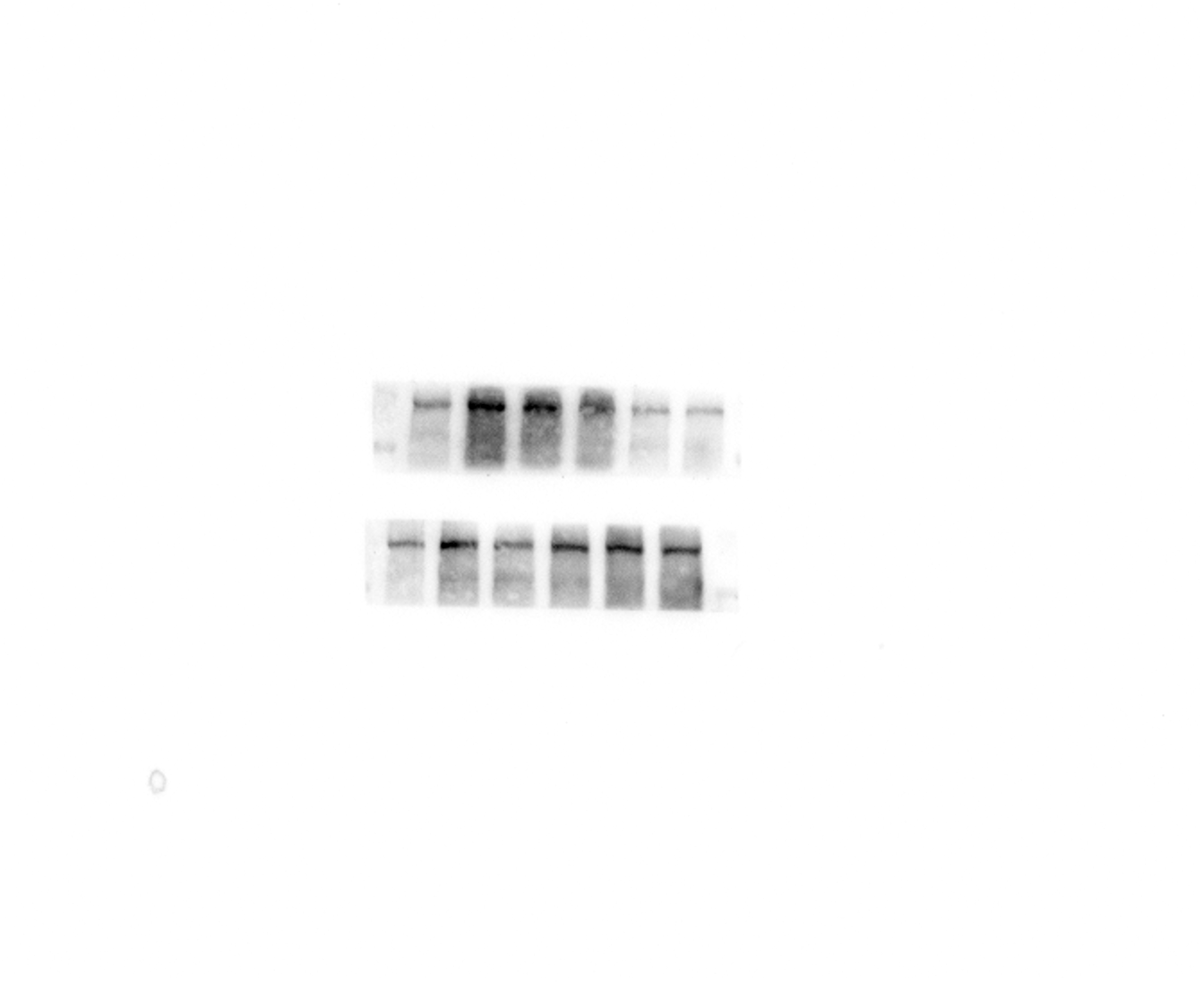

Supplement: Supplementary file 2 [file DataSheet2.ZIP › data1/2022.8.25/SIRT1/6S.Tif]

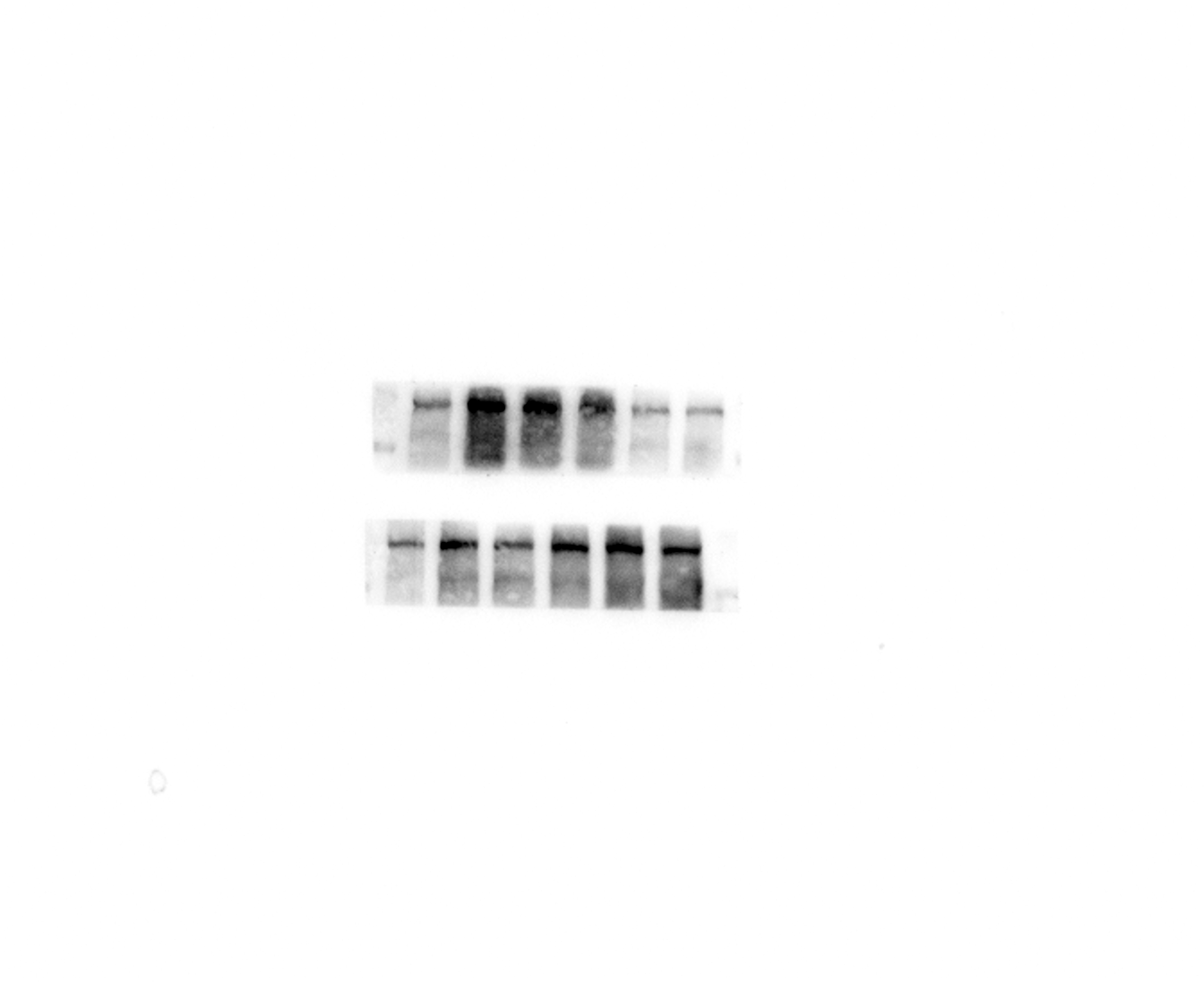

Supplement: Supplementary file 2 [file DataSheet2.ZIP › data1/2022.8.25/SIRT1/8S.Tif]

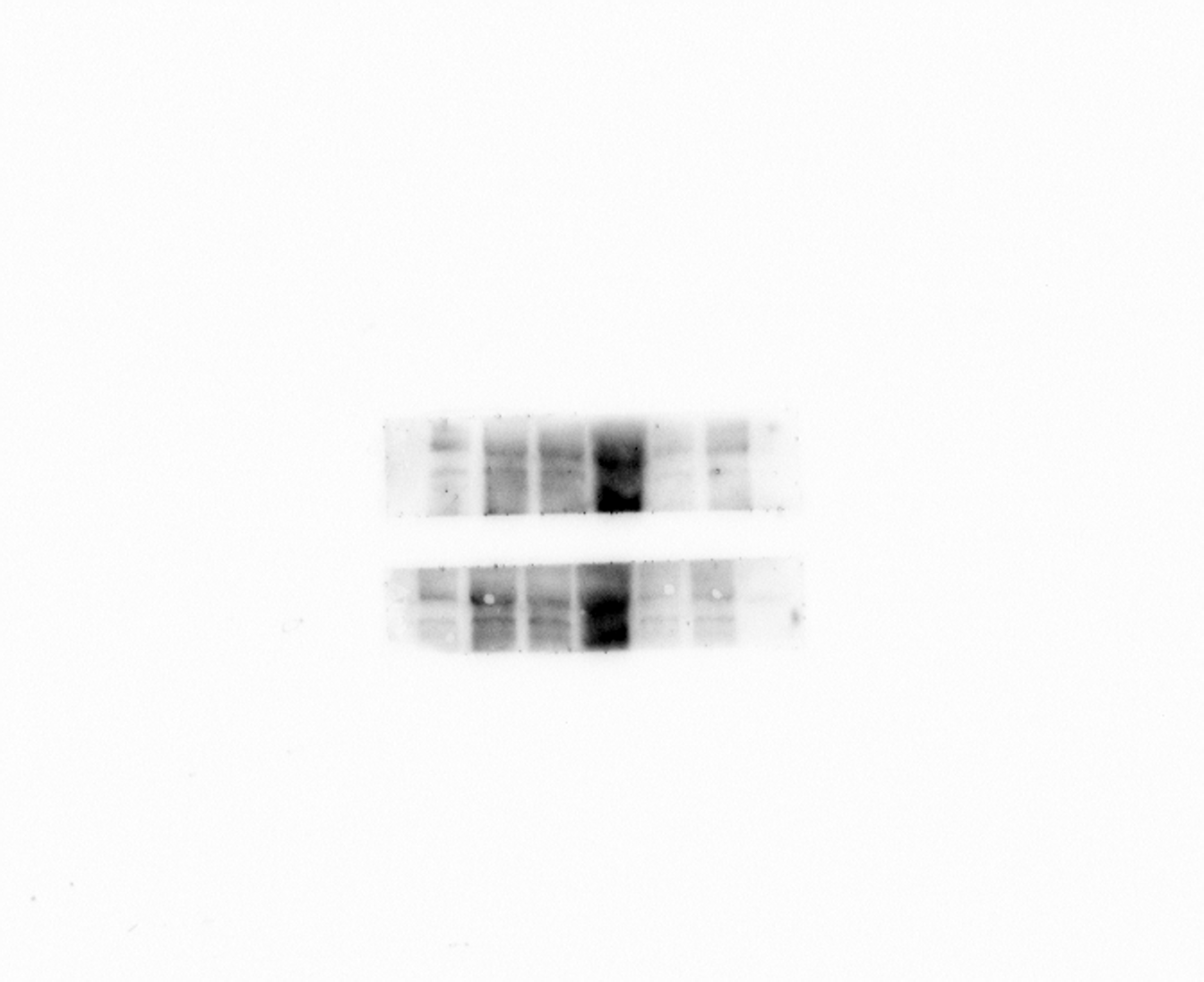

Supplement: Supplementary file 2 [file DataSheet2.ZIP › data1/2022.8.9/CHOP/10S.Tif]

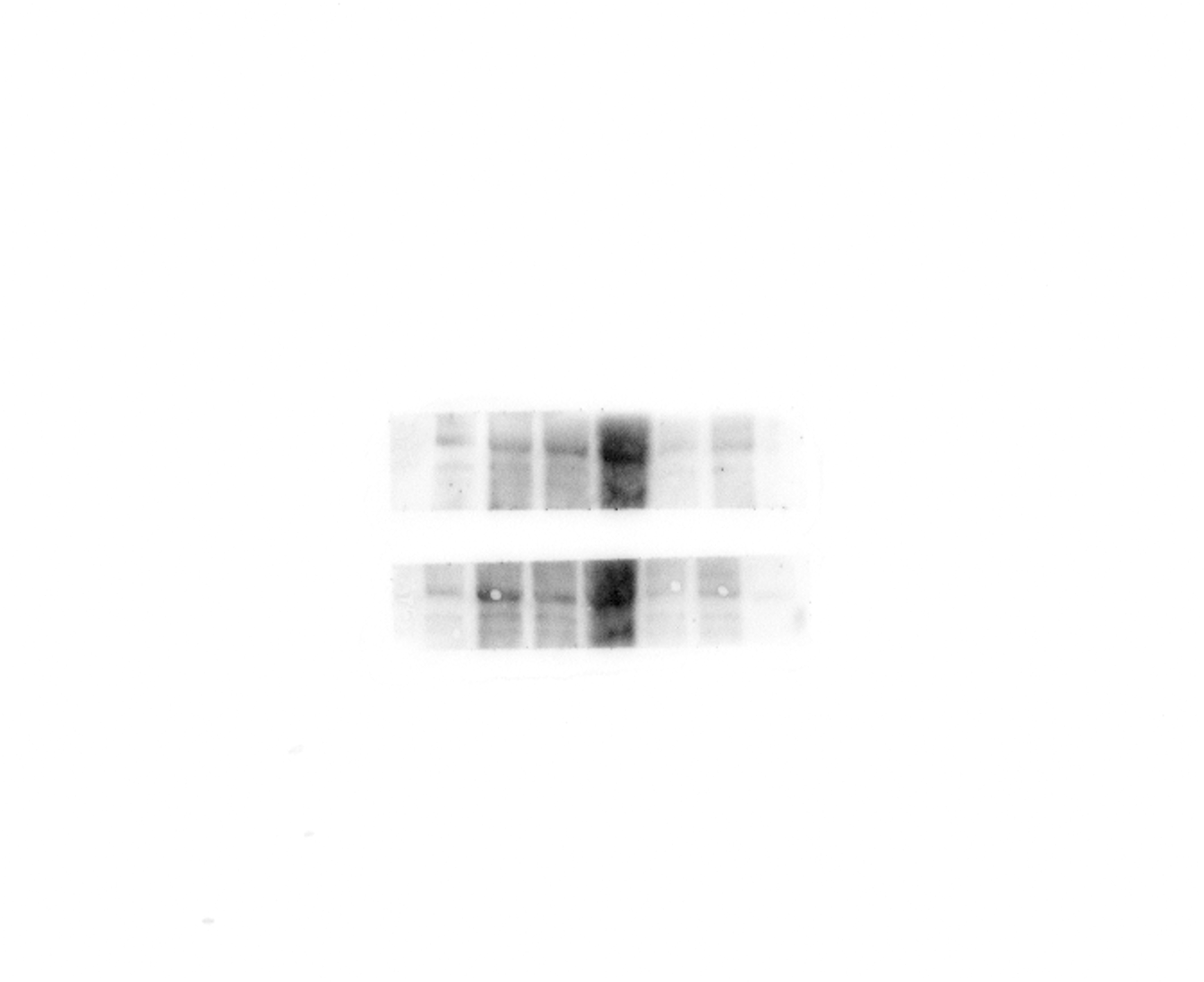

Supplement: Supplementary file 2 [file DataSheet2.ZIP › data1/2022.8.9/CHOP/5S.Tif]

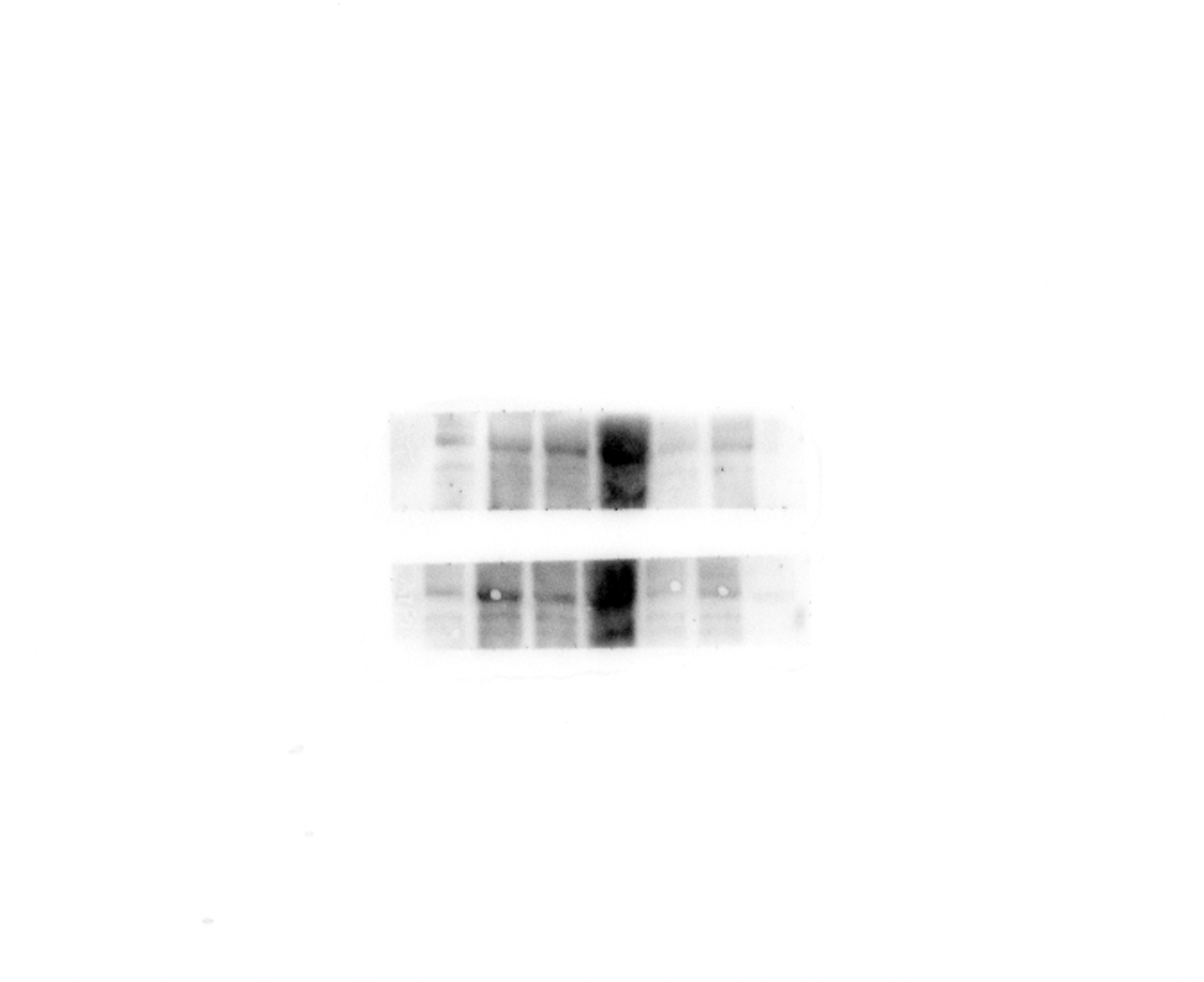

Supplement: Supplementary file 2 [file DataSheet2.ZIP › data1/2022.8.9/CHOP/6S.Tif]

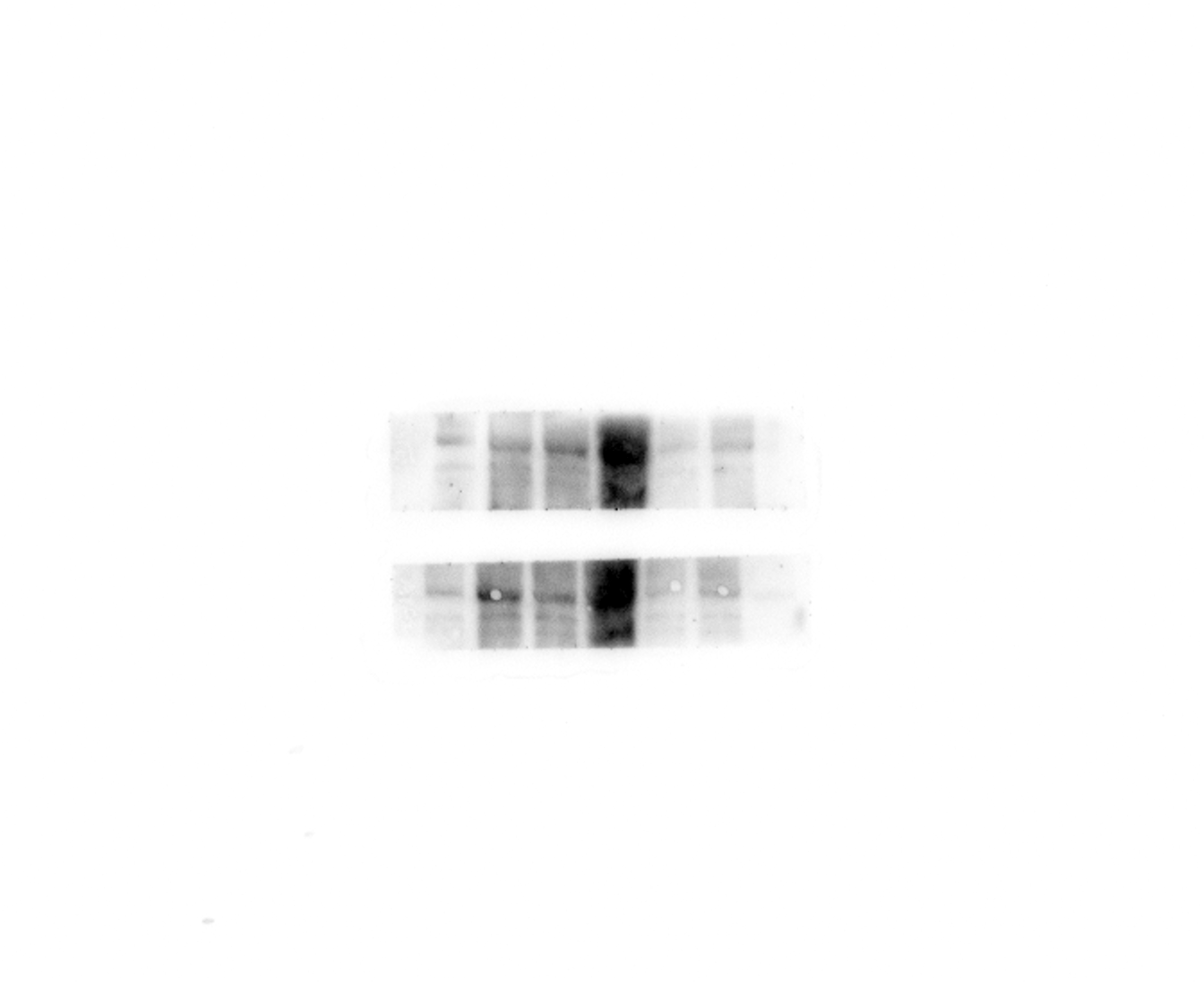

Supplement: Supplementary file 2 [file DataSheet2.ZIP › data1/2022.8.9/CHOP/7S.Tif]

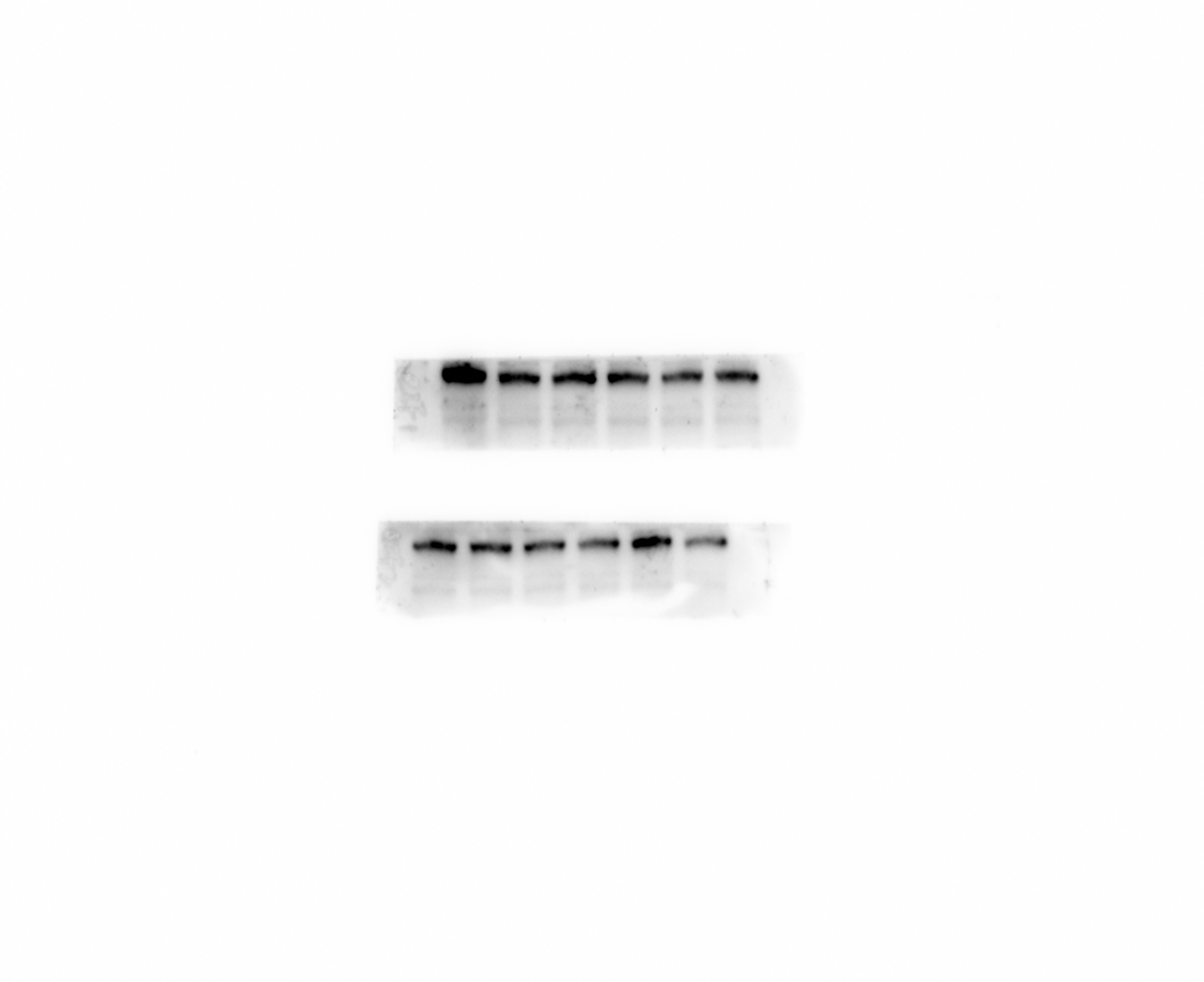

Supplement: Supplementary file 2 [file DataSheet2.ZIP › data1/2022.8.9/EIF2A/10S.Tif]

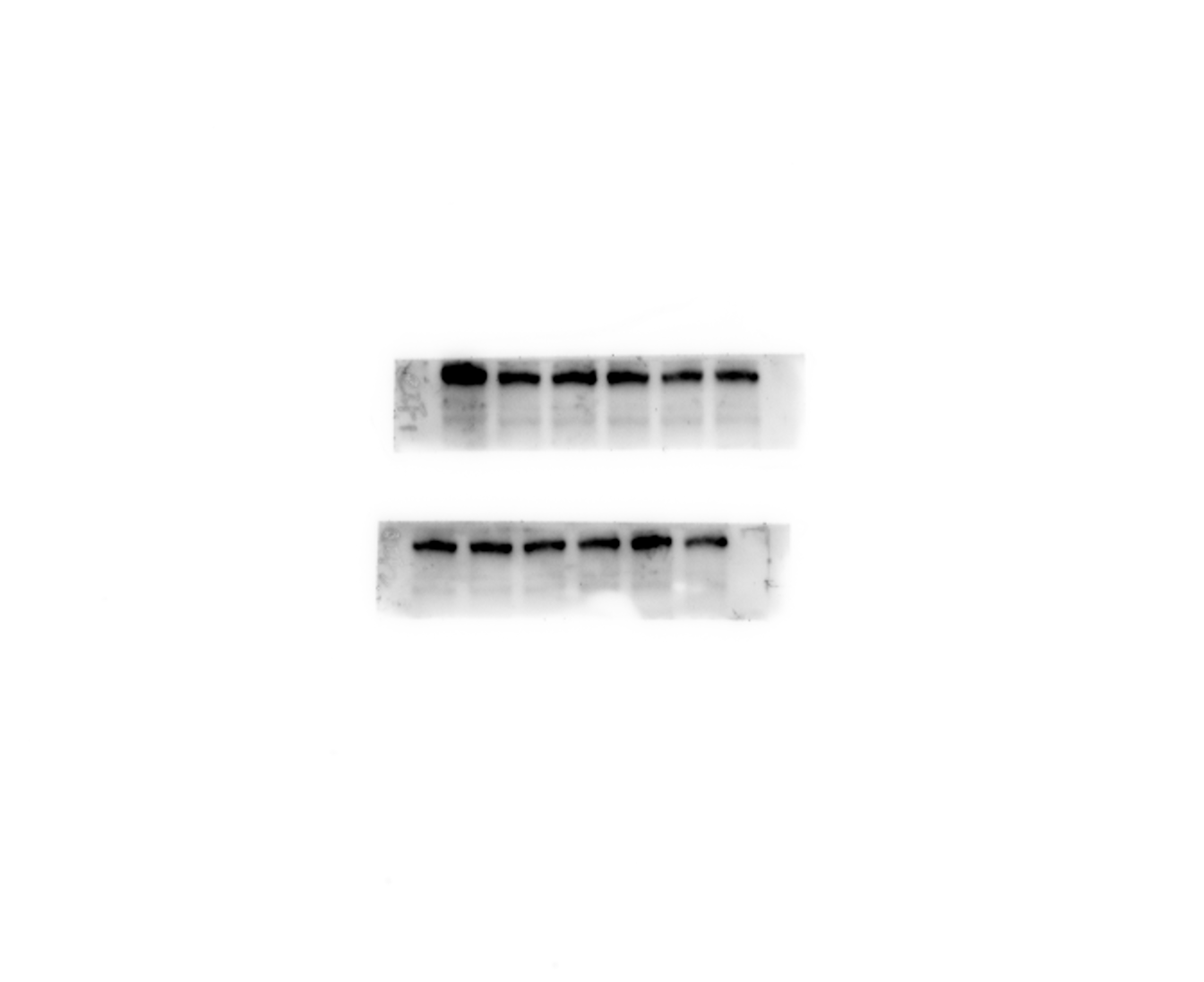

Supplement: Supplementary file 2 [file DataSheet2.ZIP › data1/2022.8.9/EIF2A/28S.Tif]

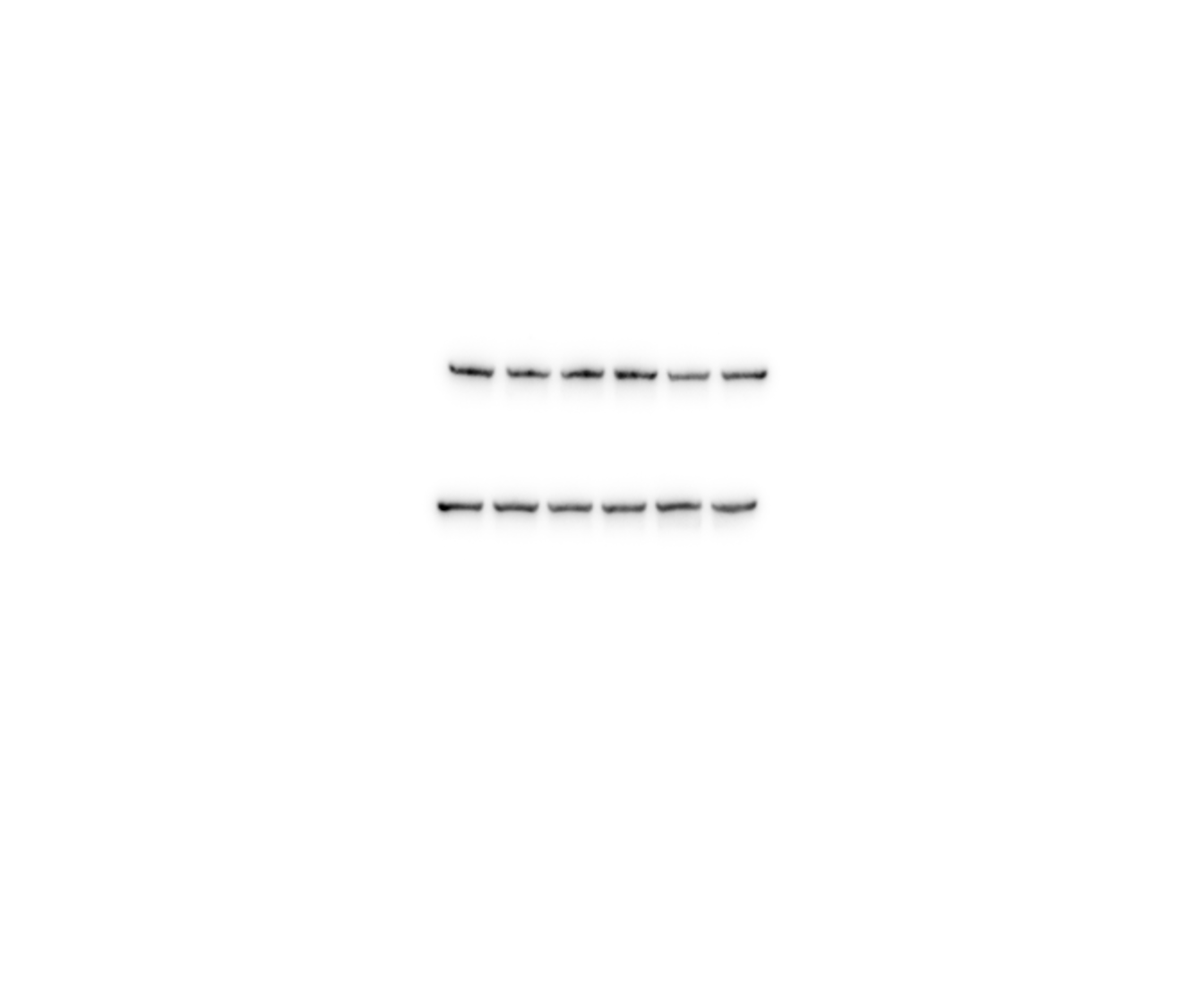

Supplement: Supplementary file 2 [file DataSheet2.ZIP › data1/2022.8.9/GAPDH/1s.Tif]

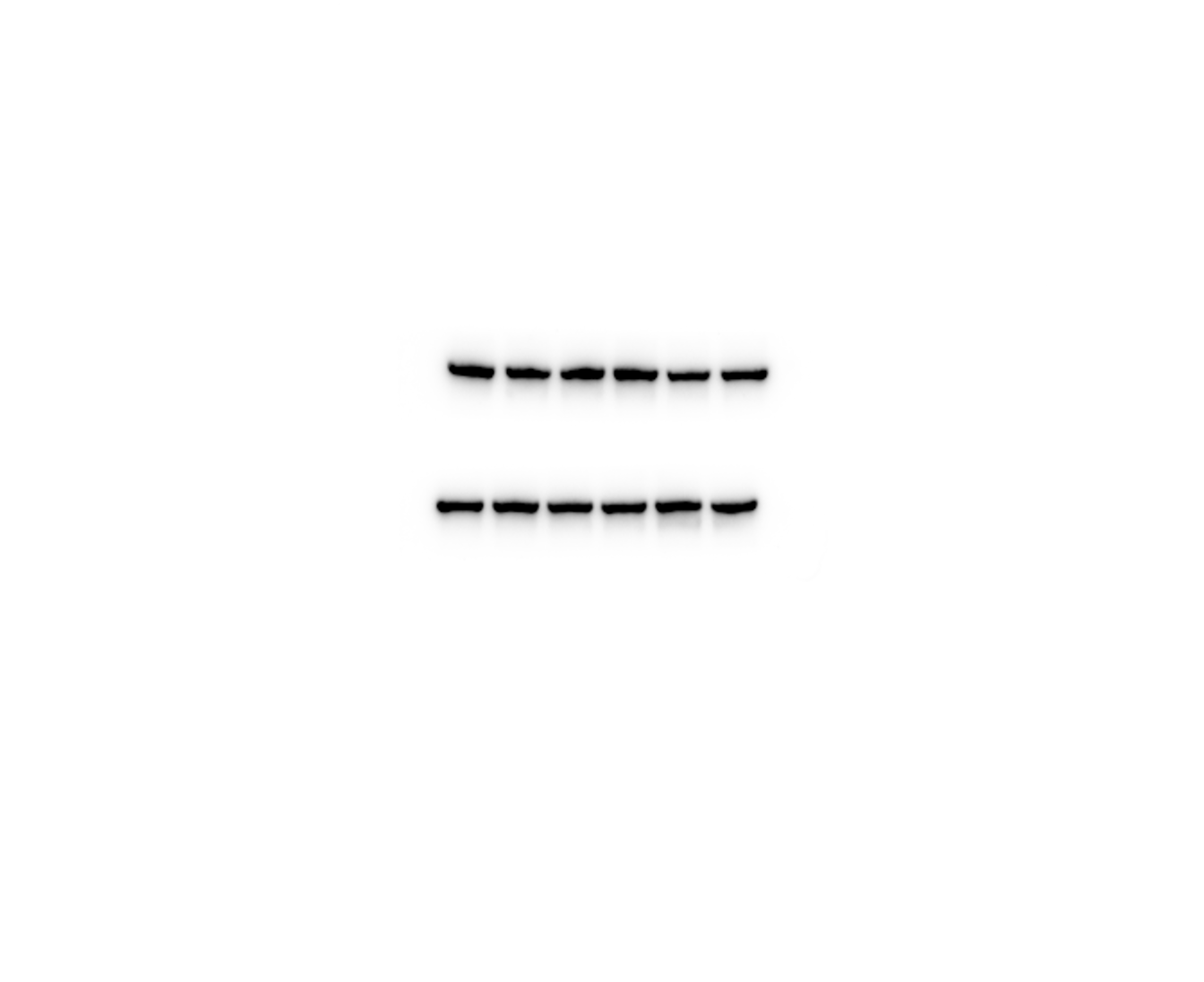

Supplement: Supplementary file 2 [file DataSheet2.ZIP › data1/2022.8.9/GAPDH/4s.Tif]

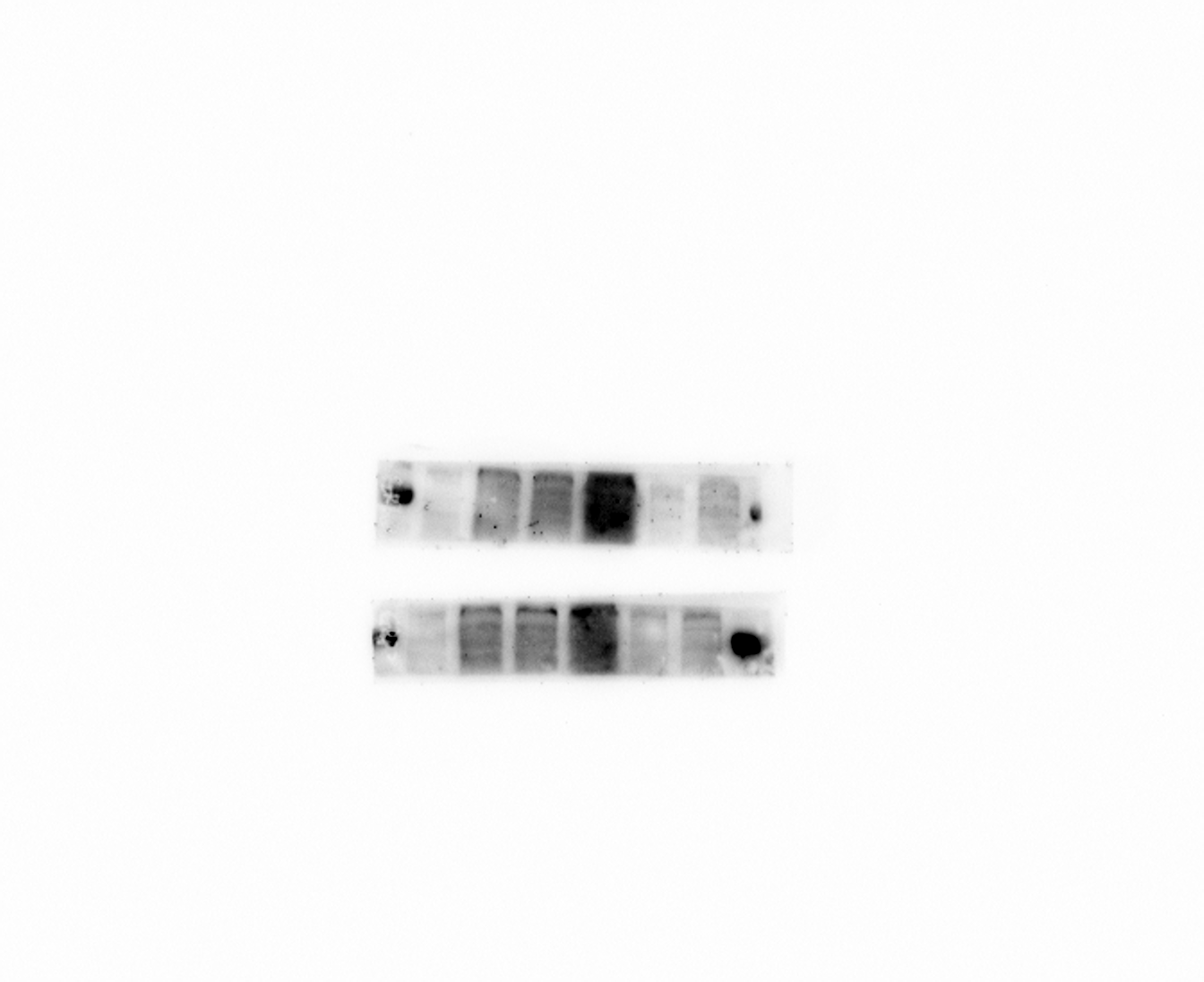

Supplement: Supplementary file 2 [file DataSheet2.ZIP › data1/2022.8.9/GRP78/10S.Tif]

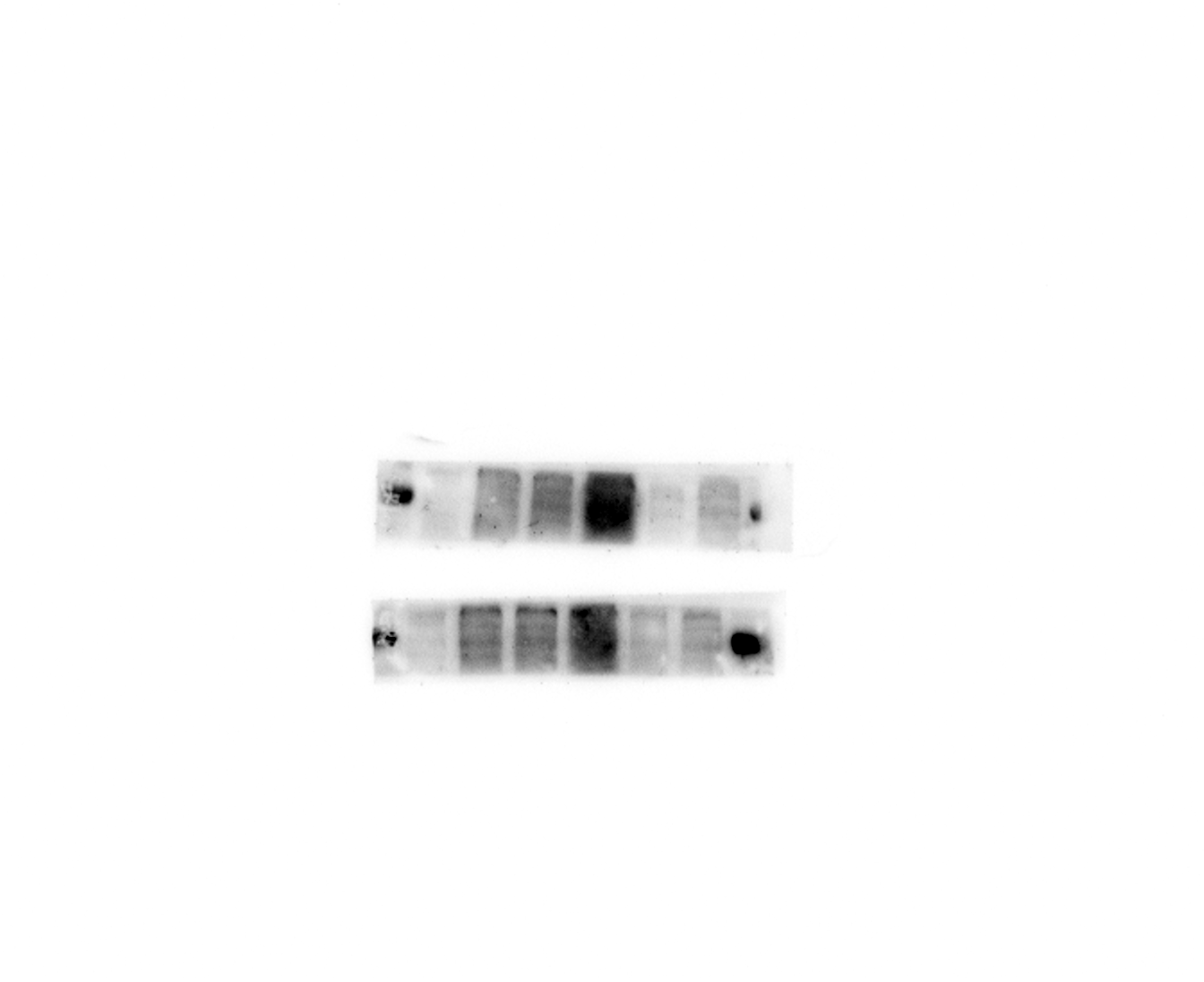

Supplement: Supplementary file 2 [file DataSheet2.ZIP › data1/2022.8.9/GRP78/6S.Tif]

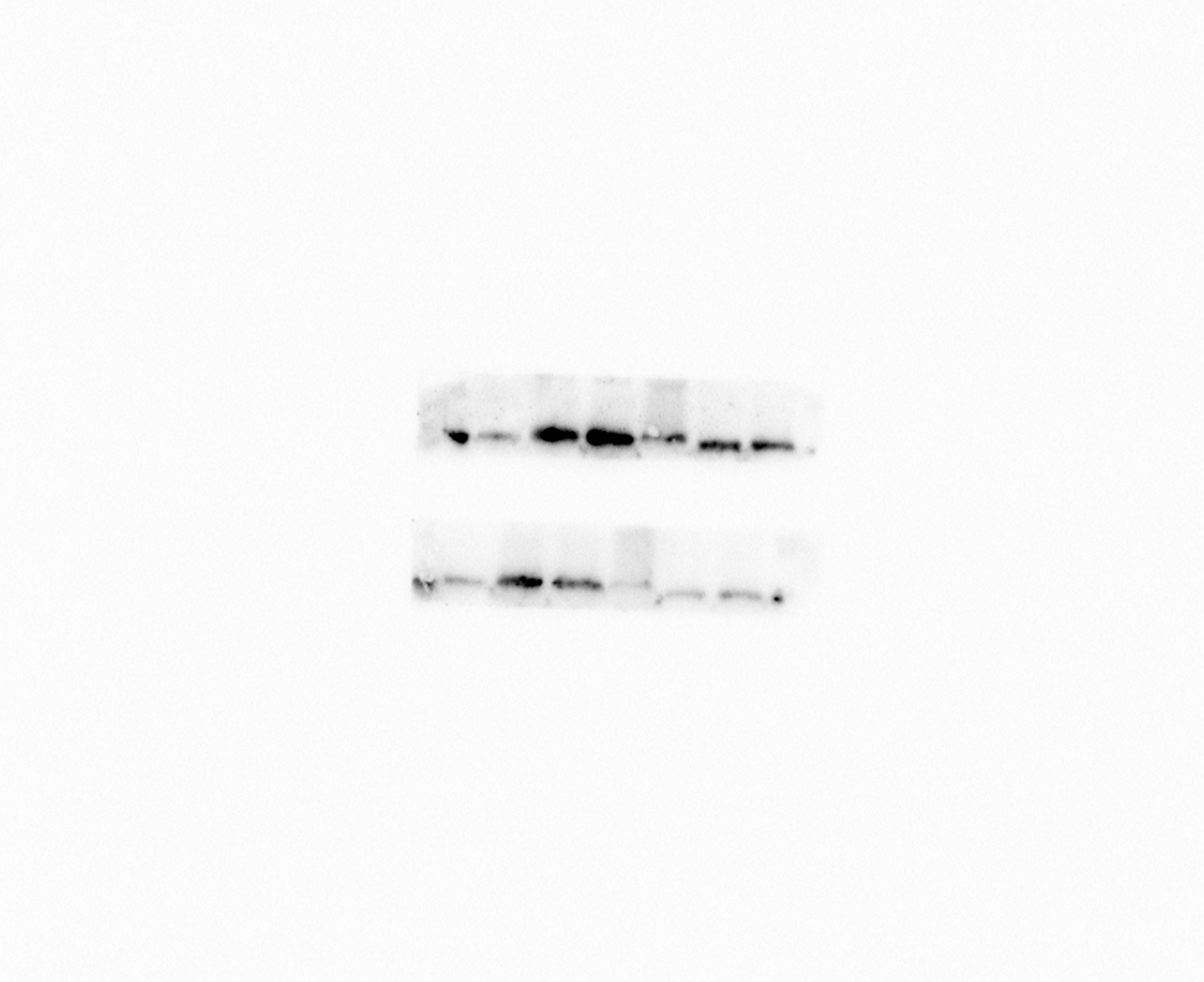

Supplement: Supplementary file 2 [file DataSheet2.ZIP › data1/2022.8.9/p-eIF2/5s.Tif]

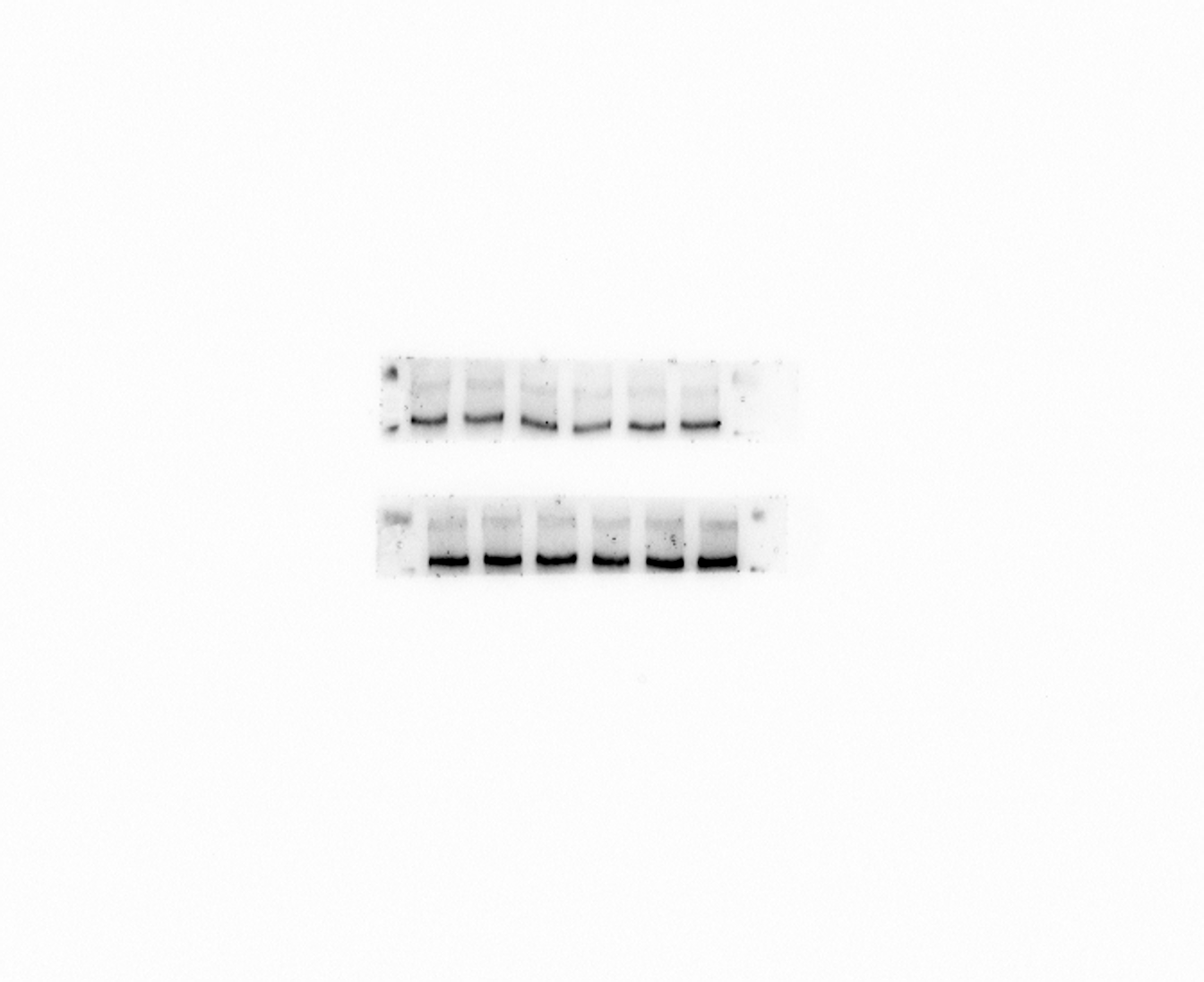

Supplement: Supplementary file 2 [file DataSheet2.ZIP › data1/2022.8.9/PERK/10S.Tif]

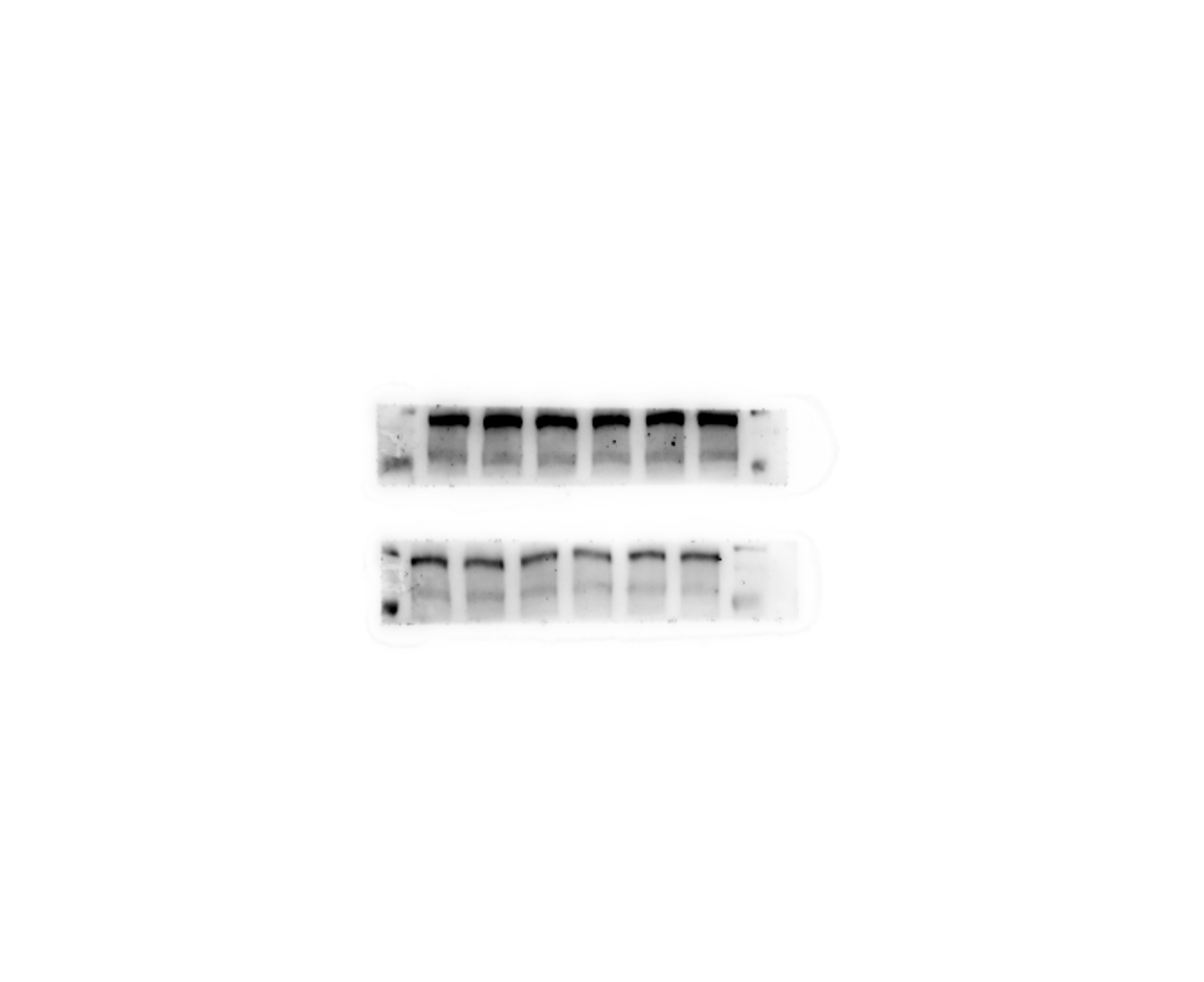

Supplement: Supplementary file 2 [file DataSheet2.ZIP › data1/2022.8.9/PERK/120S.Tif]

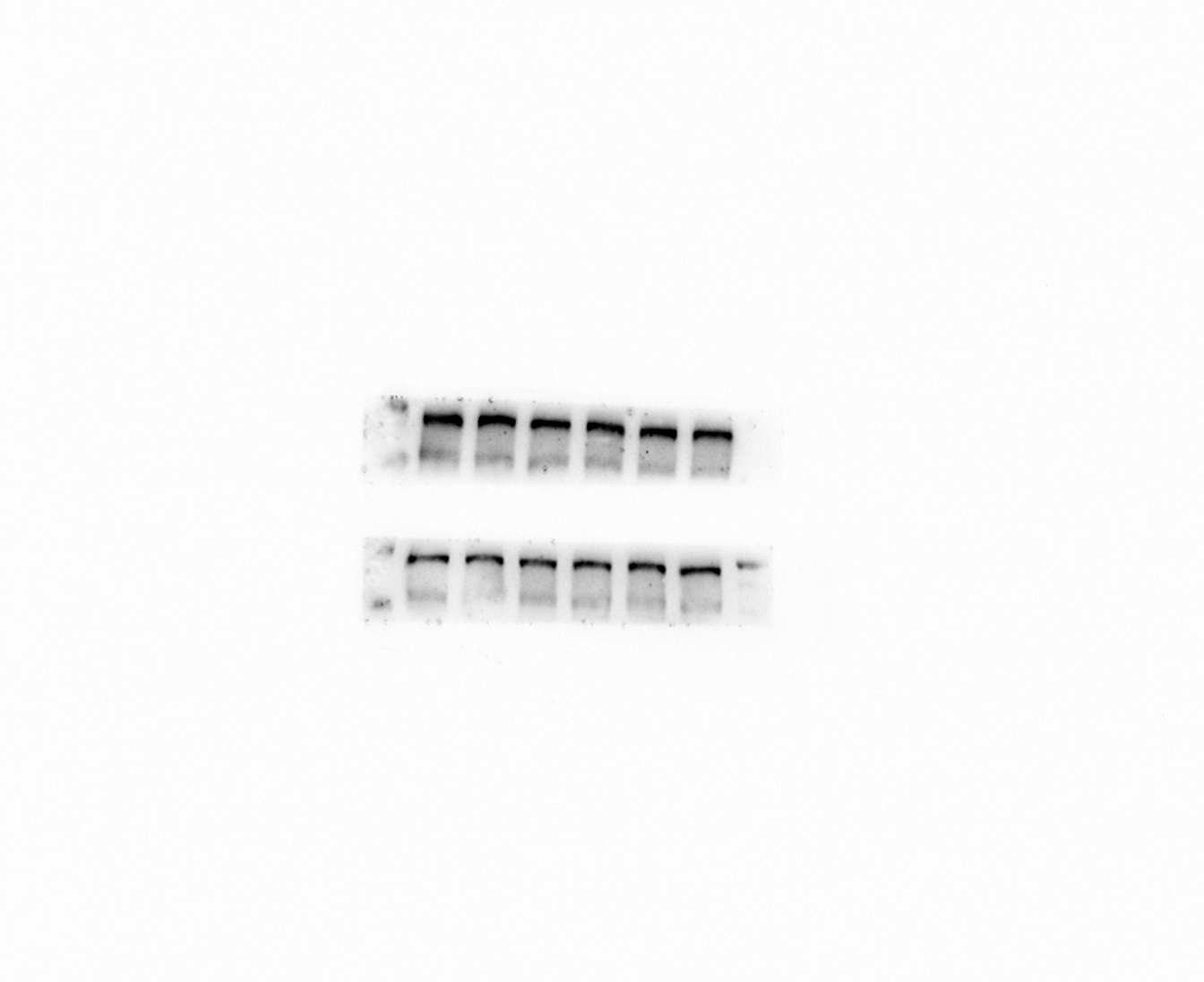

Supplement: Supplementary file 2 [file DataSheet2.ZIP › data1/2022.8.9/PERK2/10S.Tif]

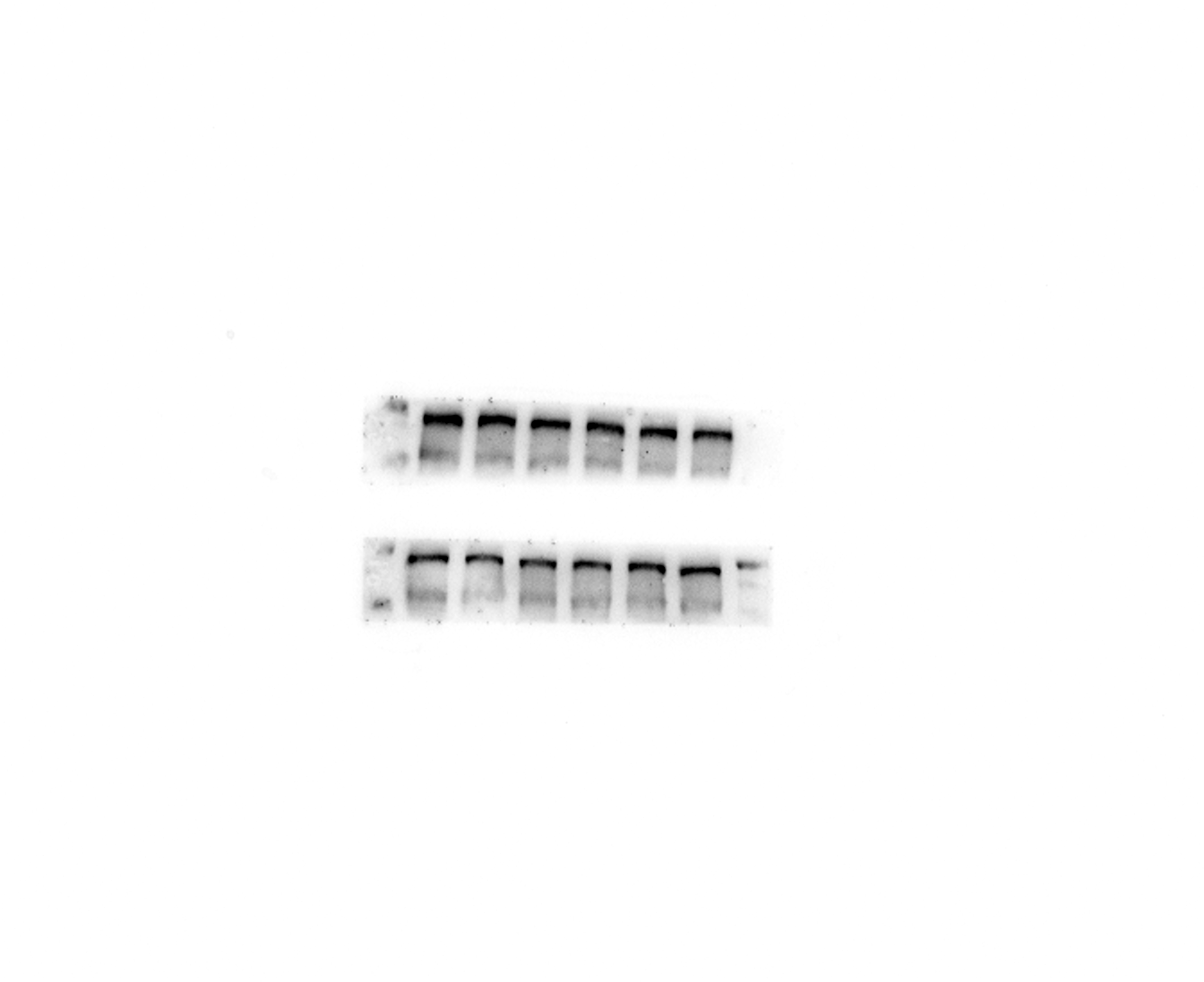

Supplement: Supplementary file 2 [file DataSheet2.ZIP › data1/2022.8.9/PERK2/8S.Tif]

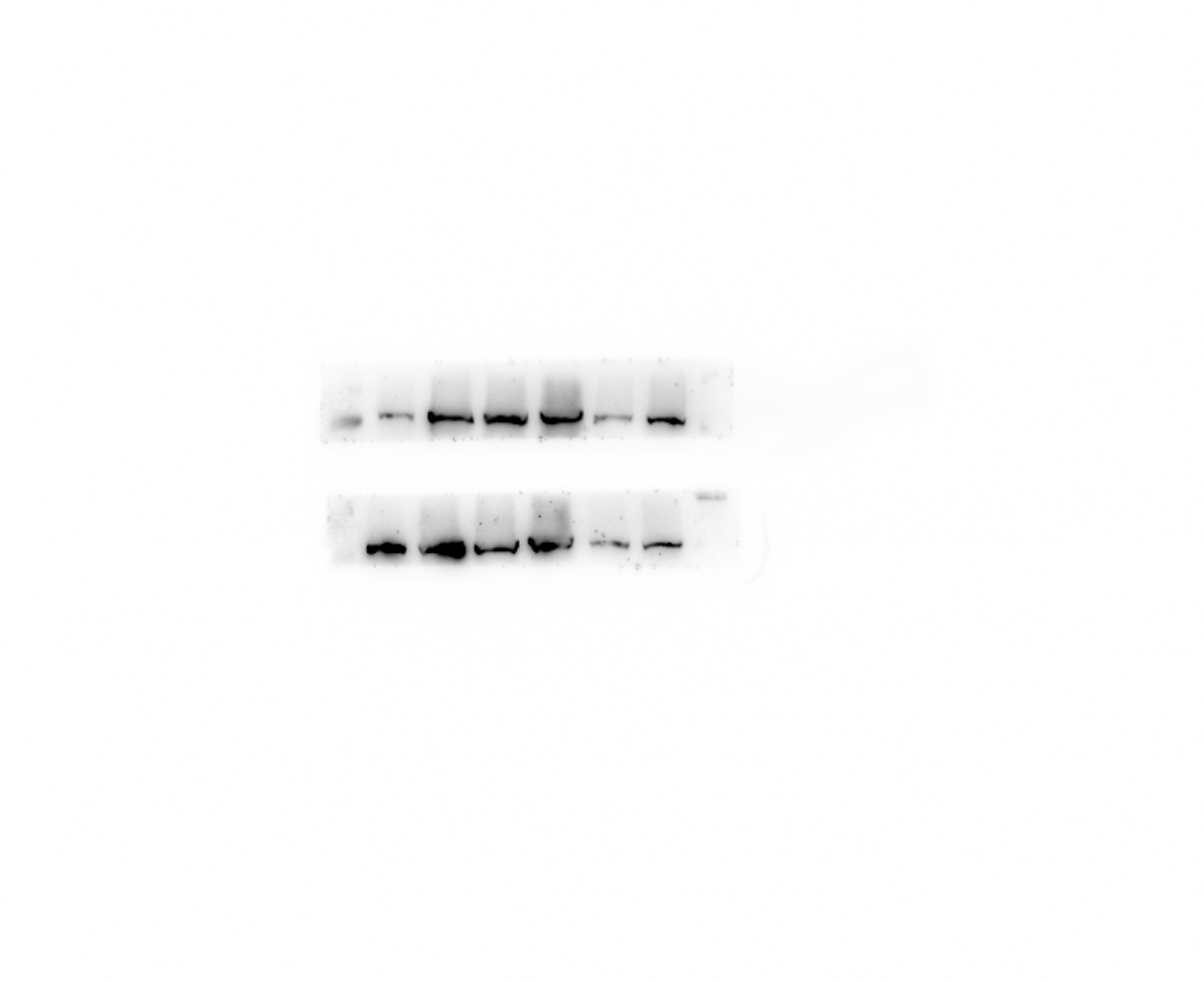

Supplement: Supplementary file 2 [file DataSheet2.ZIP › data1/2022.8.9/P-PERK/60S.Tif]

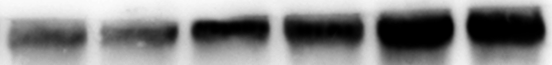

Supplement: Supplementary file 2 [file DataSheet2.ZIP › data1/2022.8.9/SIRT1/sirt1.tif]
